# Halobenzene Adducts of a Dysprosocenium Single-Molecule Magnet

**DOI:** 10.1021/acs.inorgchem.3c04105

**Published:** 2024-02-15

**Authors:** Sophie C. Corner, Gemma K. Gransbury, Iñigo J. Vitorica-Yrezabal, George F. S. Whitehead, Nicholas F. Chilton, David P. Mills

**Affiliations:** Department of Chemistry, The University of Manchester, Oxford Road, Manchester M13 9PL, U.K.

## Abstract

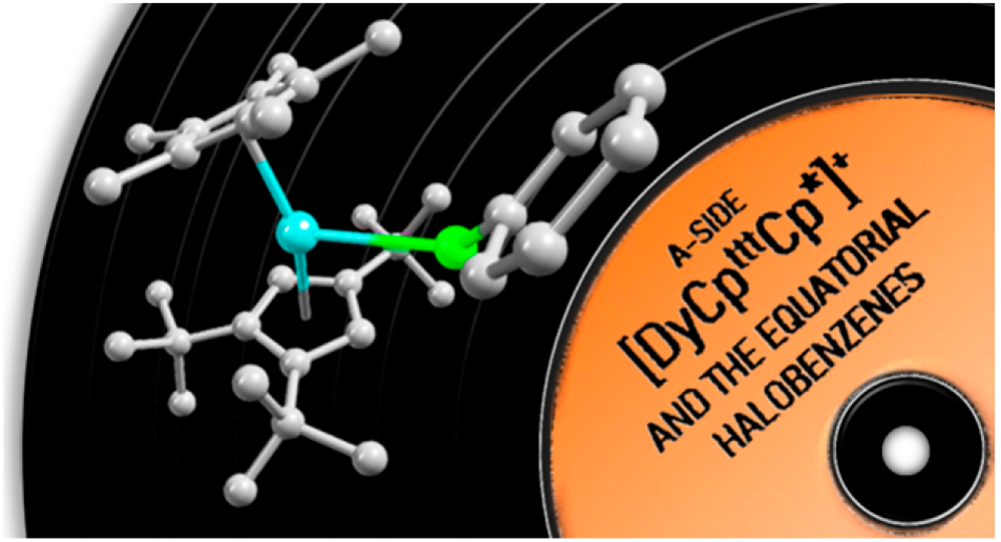

Dysprosium complexes with strong axial crystal fields are promising candidates for single-molecule magnets (SMMs), which could be used for high-density data storage. Isolated dysprosocenium cations, [Dy(Cp^R^)_2_]^+^ (Cp^R^ = substituted cyclopentadienyl), have recently shown magnetic hysteresis (a memory effect) above the temperature of liquid nitrogen. Synthetic efforts have focused on reducing strong transverse ligand fields in these systems as they are known to enhance magnetic relaxation by spin-phonon mechanisms. Here we show that equatorial coordination of the halobenzenes PhX (X = F, Cl, Br) and *o*-C_6_H_4_F_2_ to the cation of a recently reported dysprosocenium complex [Dy(Cp^ttt^)(Cp*)][Al{OC(CF_3_)_3_}_4_] (Cp^ttt^ = C_5_H_2_^t^Bu_3_-1,2,4; Cp* = C_5_Me_5_) reduces magnetic hysteresis temperatures compared to that of the parent cation. We find that this is due to increased effectiveness of both one- (Orbach) and two-phonon (Raman) relaxation mechanisms, which correlate with the electronegativity and number of interactions with the halide despite κ^1^-coordination of a single halobenzene having a minimal effect on the metrical parameters of [Dy(Cp^ttt^)(Cp*)(PhX-κ^1^-*X*)]^+^ cations vs the isolated [Dy(Cp^ttt^)(Cp*)]^+^ cation. We observe unusual divergent behavior of relaxation rates at low temperatures in [Dy(Cp^ttt^)(Cp*)(PhX)][Al{OC(CF_3_)_3_}_4_], which we attribute to a phonon bottleneck effect. We find that, despite the transverse fields introduced by the monohalobenzenes in these cations, the interactions are sufficiently weak that the effective barriers to magnetization reversal remain above 1000 cm^–1^, being only *ca*. 100 cm^–1^ lower than for the parent complex, [Dy(Cp^ttt^)(Cp*)][Al{OC(CF_3_)_3_}_4_].

## Abstract 2

We report the synthesis of monohalobenzene adducts of a heteroleptic dysprosocenium complex and probe the impact of these weak equatorial interactions on the resultant magnetic properties.

## Introduction

Single-molecule magnets (SMMs) have potential applications in high-density data storage as they can show magnetic memory effects, and lanthanide SMMs have shown the best performance to date.^1−3^ It is well established that Dy(III) complexes with highly axial crystal fields have favorable SMM properties, as these ligand arrangements stabilize the most magnetic *m*_*J*_ states while simultaneously destabilizing the least magnetic *m*_*J*_ states to give large energy barriers to magnetic reversal, *U*_eff_.^4−7^ To date, highly axial dysprosium complexes with no equatorial ligands have only been achieved using bulky substituted cyclopentadienyl ligands (Cp^R^)^8−15^ and related derivatives that contain charge-dense and rigid aromatic rings.^16−20^ This was first realized for dysprosocenium cations, [Dy(Cp^R^)_2_]^+^, where it was discovered that equatorial ligand interactions could be avoided by using bulky ring substituents and a charge-diffuse, weakly coordinating anion (WCA).^8−12^ The relatively high magnetic hysteresis temperatures (*T*_H_, the highest temperature for which hysteresis loops remain open for a sweep rate of ∼20 Oe s^–1^) and 100 s blocking temperatures (*T*_100_, the temperature for which the magnetic relaxation time is 100 s) of isolated dysprosocenium cations have been shown to be due to the combination of their highly axial ligand fields and the rigidity of multihapto Cp^R^ rings reducing the efficacy of Raman relaxation processes and quantum tunneling of the magnetization (QTM).^8,21^

It has previously been demonstrated that equatorial interactions present in Dy(III) metallocene complexes containing a {Dy(Cp*)_2_} (Cp* = {C_5_Me_5_})^7,22−40^ or {Dy(C_5_Me_4_H)_2_} core^41,42^ markedly diminish magnetic properties compared to those predicted for the idealized [Dy(Cp*)_2_]^+^ cation.^25,43^ However, a systematic study of the effect of weak equatorial ligand fields on previously isolated dysprosocenium cations has not yet been undertaken. We recently reported that a dysprosocenium cation with a Dy(III) center at the cusp of stability toward equatorial binding can exhibit both separated ion pair (SIP, [Dy(Cp^ttt^)(Cp*)][Al{OC(CF_3_)_3_}_4_]·C_6_H_6_, Cp^ttt^ = C_5_H_2_^t^Bu_3_-1,2,4) and contact ion pair (CIP, [{Dy(Cp^ttt^)(Cp*)}{Al[OC(CF_3_)_3_]_4_-κ-*F*}]) forms in the solid state, depending on crystallization conditions.^15^ Here we show that recrystallization of this complex from halobenzenes yields solvated adducts, allowing us to control the equatorial coordination and hence determine the influence of weakly coordinating solvents on the SMM properties of a dysprosocenium cation for the first time. These are also the first structurally authenticated haloarene-bound f-block complexes; only a handful of s-block^44−52^ and Sc^53^ haloarene complexes have been reported previously. We use a combination of magnetometry and complete active space self-consistent field spin–orbit (CASSCF-SO) calculations to disentangle the influence of equatorial ligand binding on the SMM behavior of the halobenzene-bound adducts compared to the parent CIP and SIP complexes.^15^ We find extremely slow and divergent relaxation times for the monohalobenzene adducts at low temperatures, and by comparison to a magnetically diluted sample of the fluorobenzene adduct, we identify unusual phonon bottleneck behavior in zero field. Surprisingly, we find that the coordination of a single halobenzene PhX (X = F, Cl, Br) in [Dy(Cp^ttt^)(Cp*)(PhX-κ^1^-*X*)]^+^ cations does not significantly change the Cp^R^···Dy···Cp^R^ angle and Dy···Cp^R^ distances vs the isolated cation, corresponding to only small differences in *U*_eff_ values observed, but the weak transverse field introduced by the haloarenes still greatly increases the magnetic relaxation rates in these systems.

## Results

### Synthesis and Characterization

**Scheme 1:**
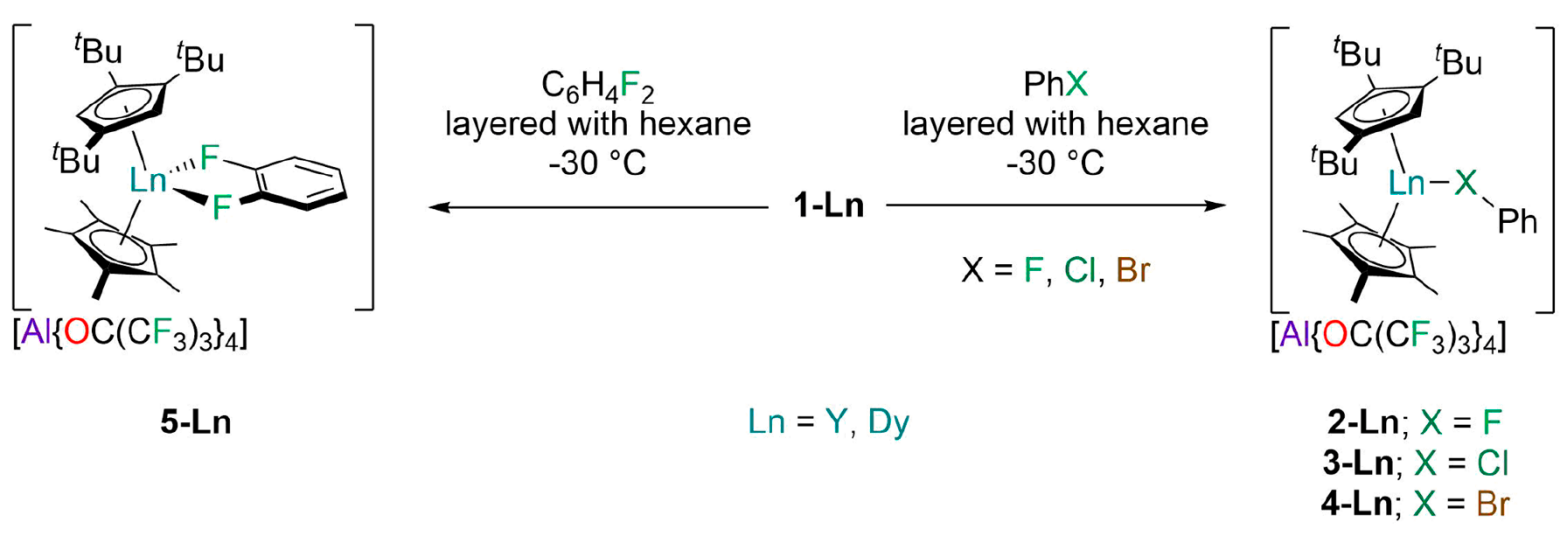
Synthesis of **2-Ln**, **3-Ln**, **4-Ln**, and **5-Ln** via the Recrystallization of **1-Ln**

The amorphous products [{Ln(Cp^ttt^)(Cp*)}{Al[OC(CF_3_)_3_]_4_}] (**1-Ln**; Ln = Y, Dy), previously determined to be a combination of the separated ion pair [Ln(Cp^ttt^)(Cp*)][Al{OC(CF_3_)_3_}_4_]·C_6_H_6_ (SIP) and the contact ion pair polymorph [Ln(Cp^ttt^)(Cp*){Al[OC(CF_3_)_3_]_4_-κ-*F*}] (CIP), were synthesized by the reported methodology.^15^ The halobenzene-bound complexes [Ln(Cp^ttt^)(Cp*)(PhX-κ-*X*)][Al{OC(CF_3_)_3_}_4_] (X = F, **2-Ln**; X = Cl, **3-Ln**; X = Br, **4-Ln**) and [Ln(Cp^ttt^)(Cp*)(C_6_H_4_F_2_-κ^2^-*F*,*F*)][Al{OC(CF_3_)_3_}_4_] (**5-Ln**) were obtained by separate recrystallizations of **1-Ln** in solutions of the parent halobenzene layered with *n*-hexane stored at −30 °C (Scheme 1); the crystalline yields of **2-Ln**, **3-Ln**, **4-Ln**, and **5-Ln** ranged from 79 to 94%. We also synthesized a doped sample, **5%Dy@2-Y**, by adding fluorobenzene to a 5:95 ratio of a mixture of **1-Dy** and **1-Y**, followed by recrystallization using procedures analogous to the synthesis of **2-Ln**.

Recrystallization of a fluorobenzene solution of **1-Y** layered with *n*-hexane at room temperature yielded [Y(Cp^ttt^)(Cp*)((PhF-κ-F)_2_][Al{OC(CF_3_)_3_}_4_] (**6-Y**). The replication of these results for the other halobenzenes investigated was not attempted at this time as the [Dy(Cp^ttt^)(Cp*)]^+^ cation has previously demonstrated a tendency to induce C–X bond cleavage of the [Al{OC(CF_3_)_3_}_4_]^−^ anion at room temperature.^15^ During one attempt to recrystallize **2-Dy**, several crystals of the mono-THF adduct [Dy(Cp^ttt^)(Cp*)(THF)][Al{OC(CF_3_)_3_}_4_] (**7-Dy**; see the SI for details) were identified by single crystal X-ray diffraction (SCXRD) analysis due to the unintentional introduction of trace amounts of THF. The preference of ethereal solvent coordination over a haloarene is well established for early metal ions^54,55^ and is consistent with results obtained for the related group 3 complex [Sc(Cp*)_2_(PhF-κ-F)_2_][BPh_4_]; NMR studies of this complex in THF revealed the formation of [Sc(Cp*)_2_(THF)_2_][BPh_4_], which was subsequently synthesized and structurally authenticated by SCXRD.^53^ Dissolving a small portion of **1-Dy** in THF and layering with *n*-hexane gave the bis-THF adduct [Dy(Cp^ttt^)(Cp*)(THF)_2_][Al{OC(CF_3_)_3_}_4_] (**8-Dy**; see the SI for details) as yellow crystals.

Various efforts to recrystallize **1-Dy** from α,α,α-trifluorotoluene or DCM layered with *n*-hexane consistently led to immediate decomposition of the product, even at low temperatures. The attempted direct synthesis of **5-Dy** via the reaction of [Dy(Cp^ttt^)(Cp*)(BH_4_)] with [CPh_3_][Al{OC(CF_3_)_3_}_4_] in ortho-difluorobenzene led to the cocrystallization of a significant amount of the dinuclear side product [{Dy(Cp^ttt^)(Cp*)}_2_(μ-BH_4_)][Al{OC(CF_3_)_3_}_4_]·0.5C_6_H_4_F_2_ (**9-Dy**; see the SI for details) with **5-Dy**, where only half an equivalent of borohydride has been abstracted. Thus, this methodology was not extended to other haloarenes.

### Solution-Phase Properties

Owing to the tendency of polar solvents to coordinate to rare earth metallocene complexes and displace the weakly coordinated halobenzenes, solution NMR studies were performed using the respective halobenzene as the solvent (see the SI for all NMR spectra collected for **2-Ln**, **3-Ln**, **4-Ln**, **5-Ln**, and **5%Dy@2-Y**). The resultant solution ^1^H NMR spectra for NMR spectra of **2-Y**, **3-Y**, **4-Y**, and **5-Y** were fully assigned, with four resonances corresponding to the four Cp^ttt^ and Cp* proton environments at the expected chemical shifts with integrals of 2:15:18:9. These peaks shift upfield with increasing halogen size, and the chemical shifts for **5-Y** are similar to those of **2-Y**. The ^13^C{^1^H} NMR spectra of **2-Y**, **3-Y**, **4-Y**, and **5-Y** could be confidently assigned following ^13^C DEPT 90 and HMBC experiments. For **4-Y**, the resonance corresponding to the ring carbon atoms of Cp* was obscured by a bromobenzene solvent signal, but all other carbon environments were observed at the expected field positions. In contrast to the ^1^H NMR spectra, halobenzene-dependent shifts were not observed in the ^13^C NMR experiments. The ^13^C{^1^H} NMR spectra of **2-Y**, **3-Y**, **4-Y**, and **5-Y** all showed coupling of ^13^C nuclei in Cp^R^ rings with 100% abundant *I* = 1/2 ^89^Y nuclei; in some cases, these coupling constants were resolved as doublets with ^1^*J*_CY_ values of 1.7–1.9 Hz. In the ^19^F NMR spectra of **2-Y**, **3-Y**, **4-Y**, and **5-Y**, the single [Al{OC(CF_3_)_3_}_4_]^−^ anion resonance was consistently seen at δ_F_ ≈ – 75 ppm. The fluorobenzene and *ortho*-difluorobenzene signals for **2-Y** and **5-Y**, at δ_F_ = −113.7 and −139.9 ppm, respectively, show chemical shifts and splitting patterns that are equivalent to those of the parent solvent; no other signals were observed.

As expected, the paramagnetism of **2-Dy**, **3-Dy**, **4-Dy**, and **5-Dy** precluded the interpretation of ^1^H and ^13^C{^1^H} NMR spectra for these complexes, as no signals could be reliably assigned. The ^19^F NMR spectra of **2-Dy** and **5-Dy** present paramagnetically broadened and shifted solvent signals at δ_F_ = −125.4 (υ_1/2_ ≈ 4200 Hz) and −148.8 ppm (υ_1/2_ ≈ 2550 Hz), respectively, which is indicative of fluxional coordination to the Dy^3+^ center in solution. Similar effects were seen for the sole [Al{OC(CF_3_)_3_}_4_]^−^ anion resonance, with an increasing impact on both the paramagnetic shift and broadening with halogen size (δ_F_, fwhm: **2-Dy**: −82.5 ppm, υ_1/2_ ≈ 180 Hz; **3-Dy**: −87.7 ppm, υ_1/2_ ≈ 340 Hz; **4-Dy**: −89.5 ppm, υ_1/2_ ≈ 380 Hz; **5-Dy**: −74.19 ppm, υ_1/2_ ≈ 140 Hz).

### Single-Crystal X-ray Diffraction

**Figure 1:**
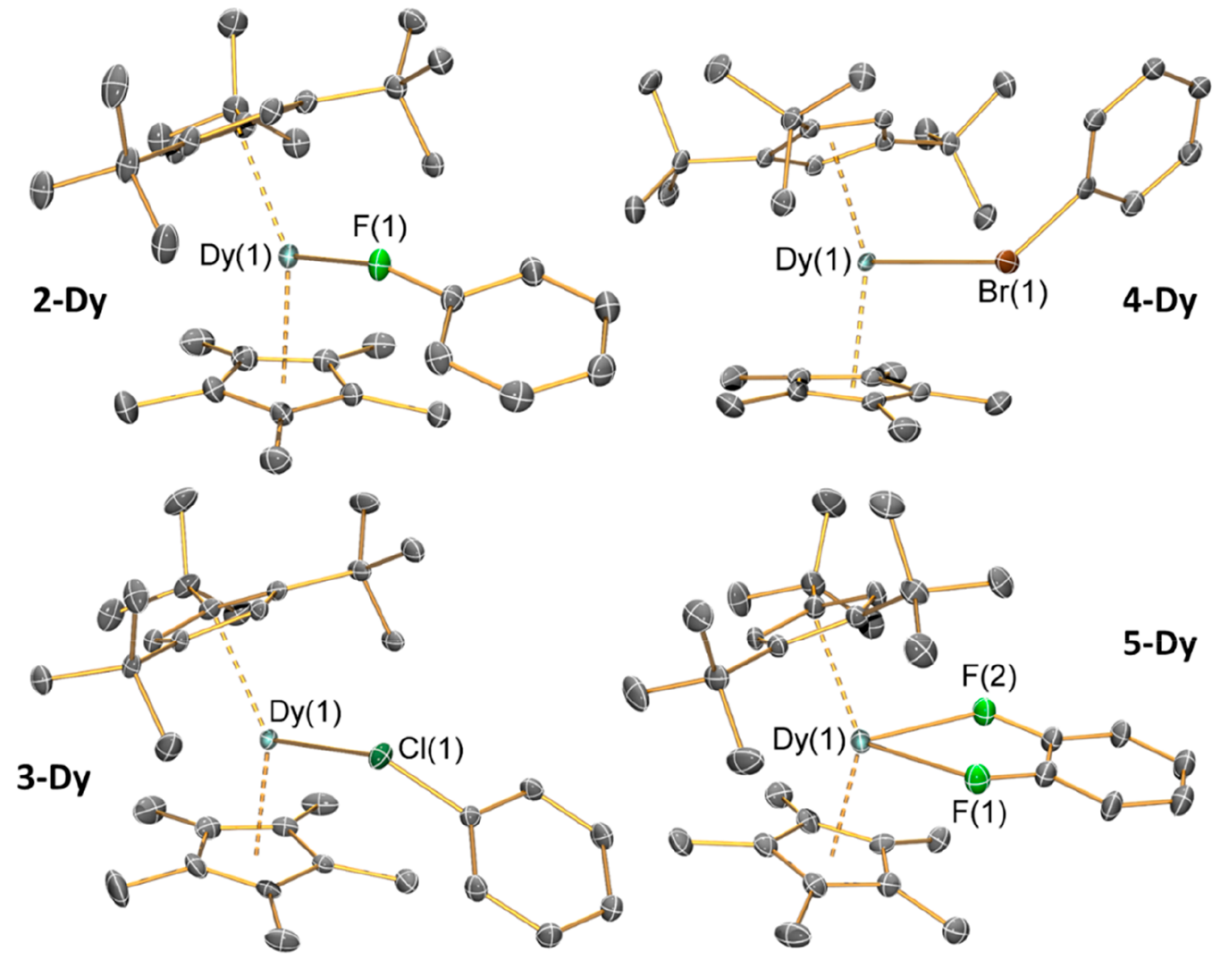
SCXRD structures of **2-Dy**, **3-Dy**, **4-Dy**, and **5-Dy** with selected atom labeling (Dy: cyan; C: gray; F: green; Cl: dark green; Br: brown). Displacement ellipsoids set at 30% probability levels; hydrogen atoms, lattice solvents, and the [Al{OC(CF_3_)_3_}_4_]^−^ anions are omitted for clarity.

The solid-state structures of **2-Ln**, **3-Ln**, **4-Ln**, **5-Ln**, **6-Y**, **7-Dy**, **8-Dy**, and **9-Dy** were determined by SCXRD; half a molecule of the respective parent halobenzene was found in the intermolecular spaces in the solid-state structures of **2-Ln**, **3-Ln**, **4-Y**, and **9-Dy**. (See Figure 1 for depictions of **2-Dy**, **3-Dy**, **4-Dy**, and **5-Dy** and Table 1 for selected parameters; see the SI for further details.) For **2-Dy**, **3-Dy**, and **4-Dy**, the equatorially coordinated PhX ligands exhibit a κ^1^-binding mode, while **5-Dy** has κ^2^-bound *o*-C_6_H_4_F_2_. The Dy–X distances increase with halogen size (**2-Dy**: 2.429(2) Å; **3-Dy**: 2.8868(7) Å; **4-Dy**: 3.035(8) Å); the difference between **3-Dy** and **4-Dy** is equivalent to that expected from the variation in halogen covalent radii upon the descent of group 17 (F: 0.64 Å; Cl: 0.99 Å; Br: 1.14 Å).^56^ Variations in Dy···Cp^R^_centroid_ distances for the halobenzene-bound complexes are insignificant (e.g., for **2-Dy**, Dy···Cp^ttt^_centroid_: 2.307(2) Å; Dy···Cp*_centroid_: 2.315(2) Å) and are in accord with the corresponding distances for the SIP and CIP.^15^ The Dy–X–C_ipso_ angles of **2-Dy**, **3-Dy**, and **4-Dy** decrease with increasing halogen size (**2-Dy**: 153.20(9)°; **3-Dy**: 126.74(3)°; **4-Dy**: 117.1(4)°). The bidentate *o*-C_6_H_4_F_2_ ligand in **5-Dy** requires a larger equatorial binding site than PhX (Table 1).^15^ Complex **5-Dy** has increased Dy–F (2.494(4) Å and 2.477(4) Å) and mean Dy···Cp^R^_centroid_ distances compared to **2-Dy** due to the increased denticity of *o*-difluorobenzene; this also leads to the Cp^ttt^_centroid_···Dy···Cp* angle of **5-Dy** (144.4(4)°) being smaller than those of the κ^1^-bound halobenzene complexes (**2-Dy**: 147.18(6)°; **3-Dy**: 147.33(5)°; **4-Dy**: 149.3(3)°), which are more similar to those seen for the SIP (149.15(9)°) and CIP (148.26(2)°).^15^

**Table 1:** Space Groups and Selected Interatomic Distances (Å) and Angles (deg) for **2-Dy**, **3-Dy**, **4-Dy**, and **5-Dy**

	**2-Dy**	**3-Dy**	**4-Dy**	**5-Dy**
Space Group	*P*2_1_/*c*	*P*2_1_/*c*	*P*2_1_/*n*	*Pc*
Dy(1)···Cp^ttt^_centroid_	2.307(2)	2.313(1)	2.317(2)	2.331(3)
Dy(1)···Cp*_centroid_	2.315(2)	2.315(2)	2.33(2)	2.345(2)
Closest Dy(1)···H	2.554	2.547	2.597	3.019
Closest Dy(1)···C	3.073(4)	3.051(2)	3.126(4)	3.621(8)
Dy(1)–X_1_	2.429(2)	2.8868(7)	3.035(8)	2.494(4)
Dy(1)–X_2_	-	-	-	2.477(4)
Corrected^56^ Dy–X_1_	1.789(2)	1.897(2)	1.895(8)	1.854(4)
Corrected^56^ Dy–X_2_	-	-	-	1.837(4)
X_1_–C	1.414(4)	1.769(2)	1.92(4)	1.38(3)
X_2_–C	-	-	-	1.39(4)
Cp^ttt^_centroid_···Dy···Cp*_centroid_	147.18(6)	147.33(5)	149.3(3)	144.4(4)
Cp^ttt^_centroid_···Dy–X_1_	106.54(6)	101.28(3)	110.7(2)	-
Cp*_centroid_···Dy–X_1_	105.78(6)	109.27(4)	99.8(2)	-
Cp^ttt^_centroid_···Dy–X_1/2_	-	-	-	108.88(11)
Cp*_centroid_···Dy–X_1/2_	-	-	-	106.0(4)
Dy–X_1_–C_ipso_	153.20(9)	126.74(3)	117.1(4)	-
Dy–X_1/2_–C_ipso_	-	-	-	169.0(5)
Largest Binding Site in {Dy(Cp^ttt^)(Cp*)} Core (% Solid Angle)	14.1	18.7	15.5	21.1

### Bulk Solid-State Characterization

To confirm the bulk composition of **2-Dy**, **3-Dy**, **4-Dy**, and **5-Dy**, powder X-ray diffraction (PXRD) with Pawley refinement (see the SI for full details)^57^ was applied to microcrystalline samples, showing excellent agreement with the expected patterns determined by SCXRD. (See below and the SI for full details.) We were unable to obtain good-quality PXRD from **5%Dy@2-Y**. The ATR-IR spectra of **2-Dy**, **3-Dy**, **4-Dy**, **5-Dy**, and **5%Dy@2-Y** presented overlapping spectra for most bands, with unique features facilitating assignment of the respective C–X stretches of halobenzenes; these vibrations were corroborated by density-functional theory (DFT) calculations on Y analogues of the cations of **2-Dy**, **3-Dy**, **4-Dy**, and **5-Dy** (see the SI for full details), with analogous calculations establishing the vibrational modes of the [Al{OC(CF_3_)_3_}_4_]^−^ anion. The calculated IR spectra were in good agreement with experimental data, allowing the confident assignment of halobenzene C–F stretches in **2-Dy** and **5%Dy@2-Y** (1112 and 770 cm^–1^) and **5-Dy** (1494 and 750 cm^–1^), the C–Cl stretch in **3-Dy** (684 cm^–1^), and the C–Br stretch in **4-Dy** (664 cm^–1^).

### Exchange Studies

Solvent exchange experiments were performed to investigate if differences in the relative binding strengths of halobenzenes to the [Dy(Cp^ttt^)(Cp*)]^+^ cation are large enough to influence the interconversion of **2-Dy**, **3-Dy**, and **4-Dy** in bulk halobenzene solutions. In separate sample vials in a glovebox, 0.5 mL halobenzene solutions of microcrystalline samples of ca. 10–20 mg of **2-Dy** (in either PhCl, PhBr, or *o*-C_6_H_4_F_2_), **3-Dy** (in PhF or PhBr), and **4-Dy** (in PhCl) were each layered with 3 mL of *n*-hexane and stored at −35 °C overnight to give yellow crystals; for **3-Dy**, we assumed that if PhF displaced PhCl then so would *o*-C_6_H_4_F_2_, and we followed the same logic for **4-Dy**. The supernatant solutions were decanted, the crystals were dried *in vacuo*, and the identities of the crystalline material formed were analyzed by a combination of SCXRD and ATR-IR spectroscopy by comparison with authentic samples (see the SI for full details). These experiments almost exclusively showed that the originally bound haloarenes were almost fully displaced by the bulk solvent, indicating that under these conditions the differences between the monohaloarene binding strengths are typically too small to affect the positions of dynamic equilibria. However, while **4-Dy** was identified by SCXRD from the sample of **2-Dy** recrystallized from PhBr/*n*-hexane, the ATR-IR spectrum exhibits absorptions corresponding to characteristic C–X stretches of both **2-Dy** and **4-Dy**. These data show that solvent exchange does not proceed to completion under the conditions employed when differences in PhX binding strength are sufficiently large and that, as expected from electrostatic considerations, PhF coordination to {Dy(Cp^ttt^)(Cp*)}^+^ is significantly stronger than PhBr binding, in accord with ^19^F NMR spectroscopy and magnetic data (see below).

### Magnetism

Microcrystalline samples of **2-Dy**, **3-Dy**, **4-Dy**, and **5-Dy** restrained in eicosane were studied with SQUID magnetometry. (See the SI for full details.) Magnetic susceptibility measurements were performed in a 0.1 T DC field at 300 K to give the products (*χT*) as 13.0 cm^3^ K mol^–1^ for **2-Dy**, 12.9 cm^3^ K mol^–1^ for **3-Dy**, 14.3 cm^3^ K mol^–1^ for **4-Dy**, and 13.1 cm^3^ K mol^–1^ for **5-Dy**. Comparison to the SIP (12.8 cm^3^ K mol^–1^) and CIP (13.1 cm^3^ K mol^–1^)^15^ values (see the SI, Figure S62) reveals **4-Dy** as an outlier that is closer to the Dy(III) free ion value of (^6^H_15/2_, *χT* = 14.17 cm^3^ K mol^–1^).^58^ The *χT* values of all of the complexes decrease slowly with temperature as the excited crystal field states are thermally depopulated. There is a marked decrease at around 16–18 K for **2-Dy**, **3-Dy**, and **4-Dy** and 6 K for **5-Dy**; this is indicative of slow magnetic dynamics on the time scale of the experiment.

Field-cooled (FC) and zero-field-cooled (ZFC) DC magnetic susceptibility measurements were performed on **2-Dy**, **3-Dy**, **4-Dy**, and **5-Dy**. This gave ZFC peaks (*T*_peak_) at 11 to 12 K for **2-Dy**, **3-Dy**, and **4-Dy** and 4 K for **5-Dy**. The irreversible points (*T*_irrev_), where the FC and ZFC measurements converge to within 1%, increase slightly according to halogen size (**2-Dy**: 23 K; **3-Dy**: 26 K; **4-Dy**: 27 K) and is reduced at 6 K for **5-Dy**. Magnetic hysteresis measurements were undertaken by using a sweep rate of 22 Oe s^–1^. The magnetization saturation (*M*_sat_) values of 4.82 Nμ_B_ for **2-Dy**, 4.78 Nμ_B_ for **3-Dy**, 5.19 Nμ_B_ for **4-Dy**, and 5.50 Nμ_B_ for **5-Dy** are all close to 5.00 Nμ_B_, which is the calculated value for a pure *m*_*J*_ = ±15/2 ground state.^59^ The hysteresis loops remained open (*T*_H_) to 22 K for **2-Dy**, 24 K for **3-Dy**, and 24 K for **4-Dy** (Table 2 and SI Figures S71–S76); for **5-Dy**, the loop is butterfly-shaped and closed around zero field even at 1.8 K. The steps observed at zero field are characteristic of QTM and are much sharper than the equivalent features observed for the SIP and CIP complexes.^15^ At 2 K, the remanent magnetization values of **2-Dy** and **3-Dy** are similar (79 and 73% of *M*_sat_, respectively; Figure S81) but lower for **4-Dy** (61%); these values are all higher than the SIP (44%) and CIP (43%).^15^ Additionally, the coercive field of the solvent adducts is increased at 1.8 K (**2-Dy**: 0.918 T; **3-Dy**: 0.986 T; **4-Dy**: 0.800 T; Figure S82) compared to those of the SIP (0.508 T) and CIP (0.335 T).^15^

Dynamic magnetic properties were investigated for **2-Dy**, **3-Dy**, **4-Dy**, **5-Dy**, and a doped sample of **2-Dy** in a diamagnetic matrix of **2-Y** (**5%Dy@2-Y**) using AC susceptibility measurements and DC magnetization decays (**2–4-Dy**, **5%Dy@2-Y**). Peaks in the out-of-phase AC susceptibilities (χ_M_″) were fitted with CC-FIT2 to the generalized Debye model to extract magnetic relaxation rates and their distributions between 44 and 86 K for **2-Dy**, 36 and 93 K for **3-Dy**, 46 and 97 K for **4-Dy**, and 2 and 73.5 K for **5-Dy**.^60,61^ Two overlapping peaks (A and B) and a shoulder (C) in χ_M_″ were observed for **5%Dy@2-Y** between 29 and 86 K (A), 12 and 68 K (B), and 8 and 53 K (C). These were fitted to the double- or triple-generalized Debye model using a customized script in CC-FIT2. Magnetization decays in a calibrated zero field were recorded for **2-Dy** (2–14 K), **3-Dy** (2–20 K), **4-Dy** (2–24 K), and **5%Dy@2-Y** (2–16 K) and fit to the stretched exponential model to extract magnetic relaxation rates as *e*^–⟨ln τ⟩^ and their distributions.^61^

**Figure 2:**
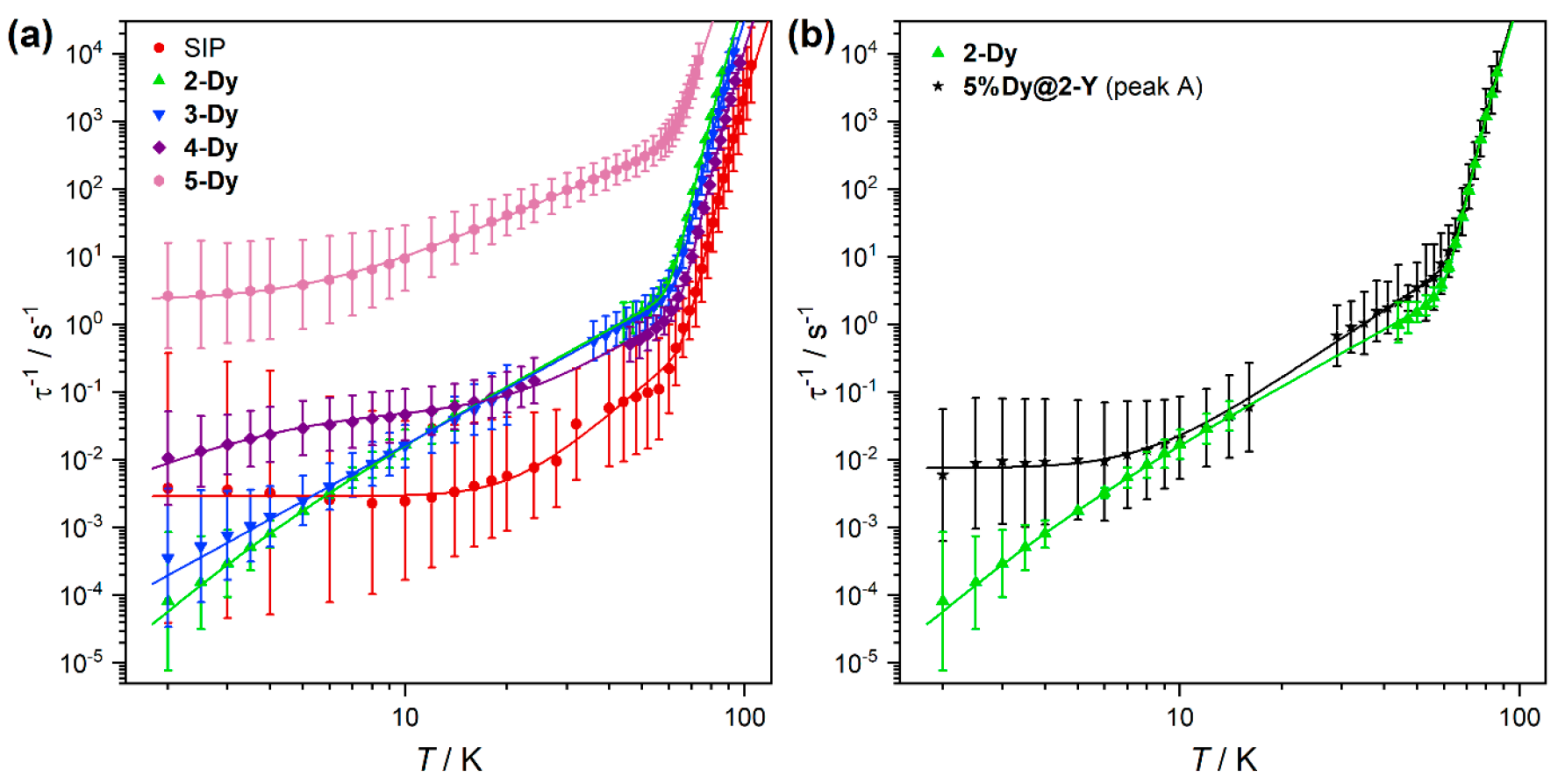
Relaxation profiles for (a) **2-Dy**, **3-Dy**, **4-Dy**, **5-Dy**, and SIP and (b) **2-Dy** and **5%Dy@2-Y** (ac peak A). Error bars represent 1 ESD in the distribution of rates.

The temperature dependence of the magnetic relaxation rates is shown in Figure 2a and can be fit by a combination of Orbach, Raman, QTM, and phonon bottleneck processes as discussed in detail below. At higher temperatures, the predominant Orbach relaxation rates follow the trend SIP ≈ CIP < **4-Dy** < **3-Dy** < **2-Dy** ≪ **5-Dy** (Figure S126);^15^ the effective barriers to magnetic reversal follow the same trend (*U*_eff_; **4-Dy**: 1182(9) cm^–1^; **3-Dy**: 1125(12) cm^–1^; **2-Dy**: 1100(9) cm^–1^; **5-Dy**: 850(13) cm^–1^). At midrange temperatures, the Raman relaxation mechanism dominates, with comparable temperature exponents for **2–4-Dy** (*n*; **2-Dy**: 2.8(1); **3-Dy**: 2.76(2); **4-Dy**: 3.1(5)) and slightly lower exponents for **5-Dy** (2.24(2)). A similar trend is seen for the Raman prefactors (log_10_[*C*/s^–1^ K^–*n*^]; **2-Dy**: −4.5(2); **3-Dy**: −4.53(3); **4-Dy**: −5.4(7); **5-Dy**: −1.34(3)). Only **5-Dy** shows the expected plateau of rates at low temperature due to QTM; fitting to a combination of Orbach, Raman, and QTM processes (Equation S7) reveals a τ_QTM_ of 10^–0.36(2)^ s, 3 orders of magnitude faster than the SIP (Table 2).

Compound **4-Dy** shows an initial plateau in rates at around 6–12 K, and then rates further decrease at lower temperatures; this unusual behavior can be explained by a phonon bottleneck effect where lattice phonons are out of equilibrium with the thermal bath (cryostat).^62,63^ The observed relaxation time is given as follows^63^
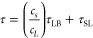
1where τ_SL_ is the spin–lattice relaxation time, τ_LB_ is the lattice-bath relaxation time, *c*_*s*_ is the specific heat of the spin system, *c*_*L*_ is the lattice specific heat, and the first term is presumed to have a power-law temperature dependence (*BT*^*–m*^). We fit the temperature dependence of relaxation rates for **4-Dy** to Equation S9 (derivation using Equation 1 based on Rousset et al.^64^) which considers Raman and QTM processes to be subject to a phonon bottleneck, while any effect of the phonon bottleneck on the Orbach process is absorbed into the value of τ_0_.^65^ This method gives a bottleneck temperature exponent of *m* = 2.0(8) and a τ_QTM_ of 10^1.3(2)^ s for **4-Dy**.

Relaxation rates for **2-Dy** and **3-Dy** do not plateau and instead reach rates slower than the SIP below 6 K. Complex **2-Dy** cannot be fit with a QTM term (τ_QTM_ > 10^4^ s; Figure S119), and a τ_QTM_ of 10^3.6(1)^ s for **3-Dy** (Figure S121) is unfeasibly slow compared to the SIP. Both **2-Dy** and **3-Dy** have increasing rate distributions at low temperature and nonmono-exponential decay at 2 K (β = 0.48; Figure S117), which is one of the hallmarks of a phonon-bottleneck effect.^66−68^ The log–log relaxation profile of **2-Dy** (Figure 2) has two linear regions that cross around 6 K, with a steeper slope at lower temperatures; fitting these rates to Equation S9 without the QTM term reveals a phonon bottleneck with a temperature exponent of *m* = 4.5(7). To further investigate the proposed bottleneck, we prepared a doped sample (**5%Dy@2-Y**) with the aim of reducing *c*_*s*_ and alleviating the bottleneck. Rates for **5%Dy@2-Y** are significantly faster than those for **2-Dy** at low temperature (Figure 2b), in accordance with the presence of a phonon bottleneck in **2-Dy**. There are reports of pure samples displaying slower zero-field QTM rates than diluted samples due to ferromagnetic dipolar interactions;^69^ however, this explanation cannot rationalize the downturn in **2-Dy** rates at low temperature. For AC measurements of **5%Dy@2-Y**, three peaks were observed; the slowest peak (A) has nearly identical Orbach rates to **2-Dy** and similar Raman rates above ca. 10 K (Figure S127), while the other peaks may correspond to other species such as the diluted Dy analog of **6-Y**. Fitting of the relaxation rates of **5%Dy@2-Y** to Equation S7 gives similar parameters to **2-Dy** but with observable QTM (τ_QTM_ = 10^2.12(5)^ s). The similarity of **3-Dy** to isomorphous **2-Dy** suggests that **3-Dy** also experiences a phonon bottleneck; however, as there is no significant downturn in rates at low temperatures, it appears that the bottleneck has similar parameters to the Raman relaxation in this sample, and the relaxation profile was fit with Orbach and Raman terms only (Equation S8).

**Table 2:** Selected SMM Parameters for **2-Dy**, **3-Dy**, **4-Dy**, **5-Dy**, and **5%Dy@2-Y** Compared with the SIP, [Dy(Cp^ttt^)(Cp*)][Al{OC(CF_3_)_3_}_4_]·C_6_H_6_, and CIP, [{Dy(Cp^ttt^)(Cp*)}{Al[OC(CF_3_)_3_]_4_-κ-F}]^15^

	SIP^15^	CIP^15^	**2-Dy**	**3-Dy**	**4-Dy**	**5-Dy**	**5%Dy@2-Y**^a^
*U*_eff_/cm^–1^	1221(25)	1265(15)	1100(9)	1125(12)	1182(9)	850(13)	1122(21)
τ_0_/s	10^–11.0(2)^	10^–11.4(1)^	10^–11.66(8)^	10^–11.53(9)^	10^–11.44(7)^	10^–11.1(1)^	10^–11.9(2)^
*C*/s^–1^ K^–*n*^	10^–8.3(5)^	10^–6.4(3)^	10^–4.5(2)^	10^–4.53(3)^	10^–5.4(7)^	10^–1.34(3)^	10^–5.2(1)^
*n*	4.3(3)	3.4(3)	2.8(1)	2.76(2)	3.1(5)	2.24(2)	3.35(7)
τ_QTM_/s	10^2.54(7)^	10^2.13(7)^	-	^–^	10^1.3(2)^	10^–0.36(2)^	10^2.12(5)^
*B*/s K^*m*^	-	-	10^5.5(3)^	-	10^2.6(3)^	-	-
*m*	-	-	4.5(7)	-	2.0(8)	-	-
*T*_H_/K	52	36	22	24	24	-	24^b^
*T*_100_/K	28	15	8	8	-	-	5

a Slowest component.

b Measurement performed with a higher density of points. At 24 K, hysteresis appears open on a similar scale to **2–5-Dy**, and above this, the coercive field is less than 50 Oe.

### Ab Initio Calculations

CASSCF-SO calculations were performed with OpenMOLCAS^70^ to investigate the electronic structures of [Dy(Cp^ttt^)(Cp*)(PhF-κ-*F*)]^+^ (**2′-Dy**), [Dy(Cp^ttt^)(Cp*)(PhCl-κ-*Cl*)]^+^ (**3′-Dy**), [Dy(Cp^ttt^)(Cp*)(PhBr-κ-*Br*)]^+^ (**4′-Dy**), and [Dy(Cp^ttt^)(Cp*)(C_6_H_4_F_2_-κ^2^-*F*,*F*)]^+^ (**5′-Dy**) using the unoptimized atomic coordinates from the SCXRD data sets. (See the SI for full details.) The ground-state Kramers doublets of **2′-Dy**, **3′-Dy**, **4′-Dy**, and **5′-Dy** have *g*_*x*_ and *g*_*y*_ ≈ 0 while *g*_*z*_ is 19.8 or 19.9, corresponding to *m*_*J*_ = ± 15/2 doublets (Tables S28–S31), with their easy axes crossing through the Cp^R^ ligands in all cases (Figures S128–S131). Predicted *U*_eff_ values are inferred from the point at which this axiality is lost, and the doublets are more highly mixed, corresponding to the fourth excited state in all cases, suggesting *U*_calc_ values of 925, 954, 960, and 849 cm^–1^ for **2′-Dy**, **3′-Dy**, **4′-Dy**, and **5′-Dy**, respectively. The trend in calculated *U*_eff_ values matches the experiment, with good absolute agreement to *U*_eff_ for **5-Dy**, while the predicted values underestimate the experimental *U*_eff_ for **2-Dy**, **3-Dy**, and **4-Dy**. Better agreement with experiment would be provided by Orbach relaxation proceeding via the sixth excited state (**2-Dy**: 1140 cm^–1^; **3-Dy**: 1159 cm^–1^; **4-Dy**: 1180 cm^–1^; see SI Tables S28–S30).

## Discussion

Halobenzenes, being weakly coordinating solvents, have previously been used to crystallize both solvent-free dysprosocenium and *bis*-phospholyl dysprosium cations.^16,71^ The solvent adducts reported herein further demonstrate the tendency of the [Dy(Cp^ttt^)(Cp*)]^+^ cation to form atypical equatorial interactions.^15^ The flexibility of this motif is reflected in the accommodation of 0, 1, or 2 equatorially coordinated atoms, as predicted previously,^15^ and the extreme variation of largest coordination site size in the {Dy(Cp^ttt^)(Cp*)}^+^ fragment: from a 12.2% solid angle in the SIP to 29.3% for **8-Dy** (Table S8).^15^ To the best of our knowledge, complexes **2-Dy**, **3-Dy**, **4-Dy**, and **5-Dy** are the first structurally characterized haloarene-bound f-block complexes; there are two previous rare earth examples for more charge-dense Sc(III) ions, [Sc(Cp*)_2_(PhF-κ-*F*)_2_][BPh_4_] and [Sc(Cp*)_2_(C_6_H_4_F_2_-κ^2^-*F*,*F*)][BPh_4_].^53^ The inclusion of the halobenzenes within the coordination spheres of **2-Ln**, **3-Ln**, **4-Ln**, and **5-Ln** displaces the WCA, which is to be expected when considering the inherent charge-diffuse nature of the WCA.^72^

For the diamagnetic Y complexes **2-Y**, **3-Y**, **4-Y**, and **5-Y**, the variation in chemical shifts observed in ^1^H NMR spectra appear to correlate with the electronegativity of the bound halogen rather than just being a consequence of the variation in dielectric constants; this demonstrates that the halobenzene solvent used coordinates to the Y^3+^ center in solution. Further evidence of halobenzene solvent binding is presented by the paramagnetic broadening and shifting of the solvent signals in the ^19^F NMR spectra of compounds **2-Dy** and **5-Dy**. However, in the ^19^F NMR spectra of **2-Y** and **5-Y**, the resonances observed are essentially identical to parent halobenzene solvent signals, indicating that these equatorial interactions are fluxional in solution at room temperature; in all cases for **2-Y**, **3-Y**, **4-Y**, and **5-Y**, a ^1^*J*_YF_ coupling constant could not be determined. Paramagnetic broadening of the ^19^F NMR signals corresponding to the WCA, which is substantial for **3-Dy** and **4-Dy**, could result from competitive binding of the halobenzene and WCA in solution; this indicates that PhCl and PhBr coordination to the {Dy(Cp^ttt^)(Cp*)}^+^ fragment is weaker than that for PhF or bidentate *o*-C_6_H_4_F_2_, in agreement with solvent exchange studies. Finally, the ^1^*J*_CY_ coupling constants for Cp^R^ ring carbon atoms extracted from the ^13^C{^1^H} NMR spectra of **2-Y**, **3-Y**, **4-Y**, and **5-Y** are comparable to those previously reported for other Y Cp^R^ complexes (e.g., [Y(Cp^ttt^)_2_][B(C_6_F_5_)_4_] (range ^1^*J*_CY_: 1.0–1.5 Hz)).^8^

Halobenzene coordination does not impact the structural parameters of the {Dy(Cp^ttt^)(Cp*)}^+^ motif in the solid state to a large extent for **2-Dy**, **3-Dy**, and **4-Dy** when compared to the SIP and CIP;^15^ this is likely a result of the point-like nature of the κ^1^ interaction, which minimizes the steric strain imposed on the system. The mean Dy···Cp^R^_centroid_ distances of **2-Dy**, **3-Dy**, and **4-Dy** are statistically similar, and the range in their Cp^ttt^_centroid_···Dy···Cp*_centroid_ angles (147.18(6)°–149.3(3)°) covers the range of respective features for the solvent-free SIP and CIP.^15^ We therefore posit that differences in SMM properties between these complexes are mainly a consequence of transverse interactions. The structural differences observed in the {Dy(Cp^ttt^)(Cp*)}^+^ core for complex **5-Dy** have been attributed to the more sterically demanding, bidentate κ^2^ interaction of *o*-difluorobenzene (the binding site comprises a 21.1% solid angle),^15^ leading to increased Dy···Cp^R^_centroid_ distances and a more acute Cp^ttt^_centroid_···Dy···Cp*_centroid_ angle. Due to the reduced axiality of the {Dy(Cp^ttt^)(Cp*)}^+^ ligand field and additional equatorial interactions, **5-Dy** exhibits faster magnetic relaxation than **2-Dy**, **3-Dy**, and **4-Dy** (see below).

The variation in Ln–X–C_ipso_ angles in the solid-state structures of **2-Ln**, **3-Ln**, and **4-Ln** can be attributed to the anisotropic electrostatic potential distribution of the halogen atoms, which has recently been postulated to dictate Mg–X–C_ipso_ angles in an analogous series of halobenzene-bound complexes, [Mg(^tBu^BDI)(PhX-κ-*X*)][B(C_6_F_5_)_4_] (^tBu^BDI = {HC[C(^t^Bu)N(Dipp)]_2_}; Dipp = 2,6-diisopropylphenyl; X = F, Cl, Br, I).^50^ The staggered arrangement of the Cp^R^ rings for **2-Dy**, **3-Dy**, and **4-Dy** is consistent with the solid-state structures of the SIP and CIP.^15^ For **5-Dy**, the higher-energy, more-eclipsed conformation adopted by the Cp^R^ rings is further evidence of the steric impact of the bidentate interaction, despite the Dy–*X*_1/2_–C_ipso_ angle being closer to linearity than the corresponding angle in **2-Dy**, **3-Dy**, and **4-Dy**. Finally, the variation in the space group of **2-Dy** and **3-Dy** (*P2*_1_/*c*) when compared to that of **4-Dy** (*P2*_1_/*n*) can be attributed to the absence of uncoordinated lattice solvent in the latter complex.

With respect to magnetic relaxation profiles, the Orbach relaxation rates follow the trend SIP ≈ CIP < **4-Dy** < **3-Dy** < **2-Dy** ≪ **5-Dy**.^15^ The much faster rates for **5-Dy** reflect the reduced axiality, and the calculated *U*_eff_ value is in good agreement with experiment, indicating relaxation via the fourth excited state. The experimental *U*_eff_ values for **2-Dy**, **3-Dy**, and **4-Dy** are only slightly lower than those for the solvent-free adducts (SIP: 1221(25) cm^–1^; CIP: 1265(15) cm^–1^; see Table 2 and Figure 2)^15^ and are much higher than previous examples of equatorially coordinated dysprosocenium complexes.^22−42^ Faster Orbach rates correlate with harder donor atoms in halobenzenes, with the more electronegative halides providing stronger transverse crystal fields. The *T*_irrev_ values in ZFC/FC measurements follow the same trend as the Orbach rates, increasing with halogen size. The Raman relaxation rates are increased for **2-Dy**, **3-Dy**, and **4-Dy** compared to the SIP and CIP^15^ due to the increase in labile degrees of freedom associated with the weak equatorial interactions. Complexes **2-Dy** and **3-Dy** are isomorphous and are expected to have similar spectral densities in the acoustic phonon region, consistent with nearly identical Raman parameters. The more bent geometry and bidentate equatorial binding for **5-Dy** result in significantly increased Raman rates, which correlate with the number of halogen atoms coordinated. The *T*_H_ values for all complexes lie within the Raman-dominated temperature range and follow the same trend: SIP > CIP > **2-Dy** ≈ **3-Dy** ≈ **4-Dy** > **5-Dy**.^15^ The coercivity values of the solvated adducts **2-Dy**, **3-Dy**, and **4-Dy** at 1.8 K are all greater than those seen for the SIP and CIP,^15^ indicating that **2-Dy**, **3-Dy**, and **4-Dy** have an increased resistance to demagnetization at low temperatures.^39^

The τ_QTM_ values follow the trend SIP > CIP ≈ **5%Dy@2-Y** > **4-Dy** > **5-Dy**, confirming that increasing the strength and number of equatorial interactions reduces axiality and facilitates QTM. The exception is **5%Dy@2-Y**; reduced dipolar fields from the dilution of spins slows QTM to be closer to the CIP than **4-Dy**. When comparing τ_QTM_ for **5%Dy@2-Y** to the 5% doped CIP,^15^ the CIP tunneling time is slower (10^2.54(4)^ s), confirming that stronger transverse crystal fields are generated by fluorobenzene than by the WCA. The presence of a phonon bottleneck has prevented the observation of the QTM region for **2-Dy** and **3-Dy**.

It is unusual for a phonon bottleneck to be observed in zero field where direct single-phonon relaxation within the ground doublet does not occur, but this appears to be the case for **2-Dy**, **3-Dy**, and **4-Dy**, leading to extremely slow relaxation at low temperature for **2-Dy** and **3-Dy**. Phonon bottlenecks for direct single-phonon relaxation processes can have temperature exponents of *m* = 1–6 depending on heat conduction.^73^ A value of *m* = 6 is expected for a spectral bottleneck with anharmonic effects,^63^ while a value of *m* = 2 is characteristic of either a spatial bottleneck (mean free paths of phonons are much smaller than crystal dimensions) or a two-phonon spectral bottleneck.^62,63^ The temperature exponents of 2.0(8) for **4-Dy** and *m* = 4.5(7) for **2-Dy** are within the expected range for phonon bottlenecks and are consistent with these rates being highly sensitive to polymorphism, even in the presence of similar coordination environments.^74^

## Conclusions

Monohalobenzene adducts of the [Dy(Cp^ttt^)(Cp*)]^+^ cation have provided an opportunity to quantify the effect of weak transverse interactions on the SMM properties of dysprosocenium complexes. At high temperatures, the coordination of a single haloarene results in accelerated Orbach relaxation rates, with smaller halides providing a larger transverse crystal field. We additionally find Raman rates to correlate with the number of coordinated halogen atoms. The bromobenzene, *o*-difluorobenzene, and yttrium-doped fluorobenzene adducts show increased QTM rates compared to the parent solvent-free complexes, once accounting for the dilution. Finally, we observe unusual phonon bottleneck regimes on the coordination of monohalobenzenes, despite the absence of direct single-phonon relaxation processes. The introduction of weak equatorial interactions promotes magnetic relaxation, although the effect is modest for the monohalobenzene adducts compared to previous examples of strong equatorial coordination; this correlates with the observation of only small changes in the Cp^R^···Dy···Cp^R^ angle and Dy···Cp^R^ distances vs the isolated [Dy(Cp^ttt^)(Cp*)]^+^ cation when only a single halobenzene is κ^1^-bound.

## Data Availability

Research data files supporting this publication are available from FigShare at 10.6084/m9.figshare.24582861.

## Supporting Information Available

Additional experimental details, materials, methods, and data associated with this article (PDF)

## Accession Codes

CDDC 2303528–2303539 contain the supplementary crystallographic data for this paper. These data can be obtained free of charge via www.ccdc.cam.ac.uk/data_request/cif, or by emailing data_request@ccdc.cam.ac.uk, or by contacting The Cambridge Crystallographic Data Centre, 12 Union Road, Cambridge, CB2 1EZ, UK; fax: + 44 1223 336033.
