# Supplementary material for: Halobenzene Adducts of a Dysprosocenium Single-Molecule Magnet
Source: Inorg Chem. 2024 Feb 15;63(21):9552–61. doi: 10.1021/acs.inorgchem.3c04105 (PMC11134494; doi:10.1021/acs.inorgchem.3c04105)
Supplement: Supplementary file 1 — ic3c04105_si_001.pdf [file ic3c04105_si_001.pdf]

Supplementary Information for:

Halobenzene Adducts of a Dysprosocenium Single-Molecule Magnet

Sophie C. Corner,[‡] Gemma K. Gransbury,[‡] Iñigo J. Vitorica-Yrezabal, George F. S. Whitehead, Nicholas F. Chilton and David P. Mills**

Department of Chemistry, The University of Manchester, Oxford Road, Manchester, M13 9PL, U.K.

Contents

1. **General Methods** S2
2. **Synthesis** S3
3. **NMR Spectroscopy** S9
4. **Powder X-ray Diffraction** S21
5. **Single crystal X-ray diffraction** S26
6. **Infrared Spectroscopy** S43
7. **DFT calculations** S49
8. **Magnetic Measurements** S58
9. **CASSCF-SO Calculations** S118
10. **Exchange studies** S127
11. **References** S134

1. General Methods

All manipulations were performed in an inert argon atmosphere with rigorous exclusion of oxygen and water using Schlenk line and glovebox techniques. The solvent *n*-hexane was dried by refluxing over potassium and stored over a potassium mirror. Fluorobenzene, *ortho*-difluorobenzene, chlorobenzene and bromobenzene were dried by stirring with CaH_2 overnight and were stored over 4 Å molecular sieves. Toluene was dried over a column charged with alumina and stored over potassium mirrors. Tetrahydrofuran (THF) was dried over a column charged with alumina and stored over 4 Å molecular sieves. All solvents were degassed before use. The reagents $[Dy(Cp^{ttt})(Cp^*)(BH_4)]$ and $[CPh_3][Al\{OC(CF_3)_3\}_4]$, and the amorphous compound "$[\{Ln(Cp^{ttt})(Cp^*)\}\{Al[OC(CF_3)_3]_4\}]$" (**1-Ln**; Ln = Y, Dy) (which was previously determined to comprise of a mixture of $[Ln(Cp^{ttt})(Cp^*)][Al\{OC(CF_3)_3\}_4]$ and $[Ln(Cp^{ttt})(Cp^*)\{Al[OC(CF_3)_3]_4\text{-}\kappa\text{-}F\}]$) were prepared according to literature methods.[1,2] ^{1}H (400 and 500 MHz), ^{13}C (126 MHz) and ^{19}F (376 MHz) NMR spectra were obtained on a Bruker Avance III 400 or 500 MHz spectrometer at 298 K and were referenced to the solvent used, to external $SiMe_4$ (^{1}H, ^{13}C) or $C_7H_5F_3/CDCl_3$ (^{19}F). ATR-IR spectra were recorded on a Bruker Alpha spectrometer with Platinum-ATR module. Elemental analysis was carried out by Mr Martin Jennings and Mrs Anne Davies at the Microanalytical service, Department of Chemistry, the University of Manchester. Elemental analysis results for complexes **2-Dy**, **3-Dy**, **4-Dy** and **5-Dy** showed lower carbon values than expected; this was ascribed to a combination of the experimental conditions employed[3] and carbide formation, which is particularly common for fluorine-rich complexes.[4]

2. **Synthesis**

[Y(Cp^{ttt})(Cp*)(PhF-κ-*F*)][Al{OC(CF$_3$)$_3$}$_4$] (2-Y). Fluorobenzene (2 mL) was used to extract **1-Y** (253 mg, 0.17 mmol), the resulting yellow solution was layered with *n*-hexane (10 mL) and stored at –30 °C. The product **2-Y** was obtained as light yellow crystals containing 0.5 eq. lattice halobenzene (228 mg, 0.15 mmol, 84%). Anal. Calcd for $C_{104}H_{103}Al_2F_{75}O_8Y_2$: C, 39.81; H, 3.31. Found: C, 38.79; H, 3.32. ^{1}H NMR (400.13 MHz, C_6H_5F with a D_2O insert, 298 K): δ = 6.31 (s, 2H, Cp-*H*), 1.77 (s, 15H, Cp-C*H*$_3$), 1.21 (s, 18H, C(C*H*$_3$)$_3$), 1.09 (s, 9H, C(C*H*$_3$)$_3$). ^{13}C{^{1}H} NMR (100.60 MHz, C_6H_5F with a D_2O insert, 298 K): δ = 139.8 (Cp-*C*C(CH$_3$)$_3$), 139.7 (d, $^1J_{CY}$ = 1.9 Hz, Cp-*C*C(CH$_3$)$_3$), 125.3 (d, $^1J_{CY}$ = 1.7 Hz, Cp-*C*(CH$_3$)), 114.5 (Cp-*C*H), 33.9 (*C*(CH$_3$)$_3$), 32.5 (*C*(CH$_3$)$_3$), 32.0 (C(*C*H$_3$)$_3$), 30.5 (C(*C*H$_3$)$_3$), 11.3 (Cp-C(*C*H$_3$)). ^{19}F NMR (376.46 MHz, C_6H_5F with a D_2O insert, 298 K): δ = –75.1 ([Al{OC(C*F*$_3$)$_3$}$_4$]$^-$), –113.5 (C$_6$H$_5$*F*). FTIR (ATR, microcrystalline): $\tilde{\nu}$ = 2966 (m, C–H stretch), 2910 (w, C–H stretch), 2869 (w, C–H stretch), 1580 (w, C=C stretch), 1484 (s, C=C stretch), 1352 (s, C–O stretch), 1297 (s), 1274 (s, C–F stretch), 1237 (s, C=C stretch), 1211 (s), 1165 (s), 1114 (s, C–F stretch), 1067 (w), 1019 (w), 970 (s), 830 (s, Al–O stretch), 775 (s, C–F stretch), 746 (s, C–H bend), 725 (s), 678 (m), 561 (s), 536 (s), 482 (w), 444 (s) cm^{-1}.

[Dy(Cp^{ttt})(Cp*)(PhF-κ-*F*)][Al{OC(CF$_3$)$_3$}$_4$] (2-Dy). Fluorobenzene (3 mL) was used to extract **1-Dy** (400 mg, 0.27 mmol), the resulting yellow solution was layered with *n*-hexane (15 mL) and stored at –30 °C. The product **2-Dy** was obtained as yellow crystals containing 0.5 eq. lattice halobenzene (411 mg, 0.25 mmol, 94%). Anal. Calcd for $C_{104}H_{103}Al_2Dy_2F_{75}O_8$: C, 38.03; H, 3.16. Found: C, 36.30; H, 3.07. The paramagnetism of **2-Dy** precluded the assignment of its ^{1}H, and ^{13}C{^{1}H} NMR spectra. ^{19}F NMR (376.46 MHz, C_6H_5F, 298 K): δ = –82.5 ($\upsilon_{1/2}$ ~ 180 Hz, [Al{OC(C*F*$_3$)$_3$}$_4$]$^-$), –125.4 ($\upsilon_{1/2}$ ~ 4200 Hz, C$_6$H$_5$*F*). FTIR (ATR, microcrystalline): $\tilde{\nu}$ = 2963 (m, C–H stretch), 2914 (w, C–H stretch), 2871 (w, C–H stretch), 1578 (w, C=C stretch), 1486 (s, C=C stretch), 1350 (s, C–O stretch), 1296 (s), 1274 (s, C–F

stretch), 1239 (s, C=C stretch), 1211 (s), 1165 (s), 1112 (s, C–F stretch), 1067 (w), 1021 (w), 970 (s), 832 (s, Al–O stretch), 806 (m, C–H bend), 770 (s, C–F stretch), 746 (s, C–H bend), 725 (s), 676 (m), 561 (s), 536 (s), 485 (w), 442 (s) cm^{-1}.

[5%Dy@Y(Cp^{ttt})(Cp*)(PhF-κ-*F*)][Al{OC(CF$_3$)$_3$}$_4$] (5%Dy@2-Y). Fluorobenzene (1 mL) was used to extract a mix of **1-Y** (0.136 g, 0.095 mmol) and **1-Dy** (0.007 g, 0.005 mmol), the resulting yellow solution was layered with *n*-hexane (10 mL) and stored at –30 °C. The product **5%Dy@2-Y** was obtained as light yellow microcrystals (248 mg, 0.08 mmol, 79%). Anal. Calcd for $C_{49}H_{49}AlDy_{0.05}F_{37}O_4Y_{0.95}$: C, 38.61; H, 3.24. Found: C, 38.59; H, 3.26. ^{1}H NMR (400.13 MHz, C_6H_5F with a D_2O insert, 298 K): δ = 6.52 (s, 2H, Cp-*H*), 1.97 (s, 15H, Cp-C*H*$_3$), 1.41 (s, 18H, C(C*H*$_3$)$_3$), 1.29 (s, 9H, C(C*H*$_3$)$_3$). ^{13}C{^{1}H} NMR (100.60 MHz, C_6H_5F with a D_2O insert, 298 K): δ = 148.2 (Cp-*C*C(CH$_3$)$_3$), 140.0 (Cp-*C*C(CH$_3$)$_3$), 125.7 (Cp-*C*(CH$_3$)), 34.2 (*C*(CH$_3$)$_3$), 32.3 (C(*C*H$_3$)$_3$), 30.8 (C(*C*H$_3$)$_3$), 11.6 (Cp-C(*C*H$_3$)). ^{19}F NMR (376.46 MHz, C_6H_5F with a D_2O insert, 298 K): δ = –75.8 ([Al{OC(C*F*$_3$)$_3$}$_4$]$^-$), –113.7 (C$_6$H$_5$*F*). FTIR (ATR, microcrystalline): $\tilde{\nu}$ = 2967 (m, C–H stretch), 2916 (w, C–H stretch), 2869 (w, C–H stretch), 1580 (w, C=C stretch), 1484 (s, C=C stretch), 1352 (s, C–O stretch), 1297 (s), 1274 (s, C–F stretch), 1237 (s, C=C stretch), 1211 (s), 1165 (s), 1116 (s, C–F stretch), 1068 (w), 1019 (w), 970 (s), 830 (s, Al–O stretch), 775 (s, C–F stretch), 746 (s, C–H bend), 725 (s), 678 (m), 560 (s), 536 (s), 483 (w), 444 (s) cm^{-1}.

[Y(Cp^{ttt})(Cp*)(PhCl-κ-*Cl*)][Al{OC(CF$_3$)$_3$}$_4$] (3-Y). Chlorobenzene (2 mL) was used to extract **1-Y** (251 mg, 0.17 mmol), the resulting yellow solution was layered with *n*-hexane (10 mL) and stored at –30 °C. The product **3-Y** was obtained as light yellow crystals containing 0.5 eq. lattice halobenzene (185 mg, 0.12 mmol, 68%). Anal. Calcd for $C_{104}H_{103}Al_2Cl_3F_{72}O_8Y_2$: C, 39.20; H, 3.26. Found: C, 37.47; H, 3.36. ^{1}H NMR (400.13 MHz, C_6H_5Cl with a D_2O insert, 298 K): δ = 5.88 (s, 2H, Cp-*H*), 1.60 (s, 15H, Cp-C*H*$_3$), 0.98 (s, 18H, C(C*H*$_3$)$_3$), 0.80 (s, 9H, C(C*H*$_3$)$_3$). ^{13}C{^{1}H} NMR (100.60 MHz, C_6H_5Cl with a D_2O insert, 298 K): δ = 139.6 (Cp-

$CC(CH_3)_3$), 137.9 (Cp-$CC(CH_3)_3$), 126.4 (d, $^1J_{CY}$ = 1.8 Hz, Cp-$C(CH_3)$), 114.0 (d, $^1J_{CY}$ = 1.7 Hz, Cp-CH), 34.0 ($C(CH_3)_3$), 31.8 ($C(CH_3)_3$), 31.5 ($C(CH_3)_3$), 29.7 ($C(CH_3)_3$), 11.5 (Cp-$C(CH_3)$). ^{19}F NMR (376.46 MHz, C_6H_5Cl with a D_2O insert, 298 K): δ = –75.2 ($[Al\{OC(CF_3)_3\}_4]^-$). FTIR (ATR, microcrystalline): $\tilde{\nu}$ = 2965 (m, C–H stretch), 2914 (w, C–H stretch), 2869 (w, C–H stretch), 1478 (m, C=C stretch), 1350 (s, C–O stretch), 1297 (s), 1272 (s, C–F stretch), 1239 (s, C=C stretch), 1211 (s), 1161 (s), 1087 (w), 1060 (w), 1019 (w), 970 (s), 830 (s, Al–O stretch), 742 (s, C–H bend), 725 (s), 684 (s, C–Cl stretch), 561 (s), 536 (s), 442 (s) cm^{-1}.

$[Dy(Cp^{ttt})(Cp^*)(PhCl\text{-}\kappa\text{-}Cl)][Al\{OC(CF_3)_3\}_4]$ (3-Dy). Chlorobenzene (3 mL) was used to extract **1-Dy** (400 mg, 0.27 mmol), the resulting yellow solution was layered with *n*-hexane (15 mL) and stored at –30 °C. The product **3-Dy** was obtained as yellow crystals containing 0.5 eq. lattice halobenzene (356 mg, 0.21 mmol, 79%). Anal. Calcd. for $C_{104}H_{103}Al_2Cl_3Dy_2F_{72}O_8$: C. 37.47; H, 3.11. Found: C, 35.59; H, 2.92. The paramagnetism of **3-Dy** precluded the assignment of its ^{1}H and $^{13}C\{^1H\}$ NMR spectra. ^{19}F NMR (376.46 MHz, C_6H_5Cl, 298 K): δ = –87.7 ($\upsilon_{1/2}$ ~ 340 Hz, $[Al\{OC(CF_3)_3\}_4]^-$). FTIR (ATR, microcrystalline): $\tilde{\nu}$ = 2965 (m, C–H stretch), 2912 (w, C–H stretch), 2871 (w, C–H stretch), 1478 (m, C=C stretch), 1350 (s, C–O stretch), 1297 (s), 1272 (s, C–F stretch), 1239 (s, C=C stretch), 1211 (s), 1163 (s), 1085 (w), 1060 (w), 1019 (w), 968 (s), 832 (s, Al–O stretch), 742 (s, C–H bend), 725 (s), 686 (s, C–Cl stretch), 559 (s), 536 (s), 442 (s) cm^{-1}.

$[Y(Cp^{ttt})(Cp^*)(PhBr\text{-}\kappa\text{-}Br)][Al\{OC(CF_3)_3\}_4]$ (4-Y). Bromobenzene (2 mL) was used to extract **1-Y** (249 mg, 0.17 mmol), the resulting yellow solution was layered with *n*-hexane (10 mL) and stored at –30 °C. The product **4-Y** was obtained as light yellow crystals containing 0.5 eq. lattice halobenzene (159 mg, 0.10 mmol, 56%). Anal. Calcd for $C_{104}H_{103}Al_2Br_3F_{72}O_8Y_2$: C, 37.62; H, 3.13. Found: C, 36.35; H, 3.20. ^{1}H NMR (400.13 MHz, C_6H_5Br with a D_2O insert, 298 K): δ = 5.67 (s, 2H, Cp-H), 1.40 (s, 15H, Cp-CH_3), 0.80 (s, 18H, $C(CH_3)_3$), 0.56 (s, 9H,

C(CH_3)$_3$). $^{13}C\{^1H\}$ NMR (100.60 MHz, C_6H_5Br with a D_2O insert, 298 K): δ = 139.2 (Cp-*C*C(CH$_3$)$_3$), 137.7 (Cp-*C*C(CH$_3$)$_3$), 113.6 (Cp-*C*H), 33.8 (*C*(CH$_3$)$_3$), 31.6 (*C*(CH$_3$)$_3$), 31.5 (C(*C*H$_3$)$_3$), 29.6 (C(*C*H$_3$)$_3$), 11.7 (Cp-C(*C*H$_3$)). Cp-*C*(CH$_3$) not observed. ^{19}F NMR (376.46 MHz, C_6H_5Br with a D_2O insert, 298 K): δ = –75.4 ([Al{OC(C*F*$_3$)$_3$}$_4$]$^-$). FTIR (ATR, microcrystalline): $\tilde{\nu}$ = 2966 (m, C–H stretch), 2920 (w, C–H stretch), 2871 (w, C–H stretch), 1474 (m, C=C stretch), 1350 (s, C–O stretch), 1295 (s), 1274 (s, C–F stretch), 1237 (s, C=C stretch), 1211 (s), 1163 (s), 1070 (w), 1021 (w), 970 (s), 832 (s, Al–O stretch), 740 (s, C–H bend), 725 (s), 678 (m), 664 (m, C–Br stretch), 561 (s), 536 (s), 442 (s) cm^{-1}.

[Dy(Cp^{ttt})(Cp*)(PhBr-κ-*Br*)][Al{OC(CF$_3$)$_3$}$_4$] (4-Dy). Bromobenzene (3 mL) was used to extract **1-Dy** (400 mg, 0.27 mmol), the resulting yellow solution was layered with *n*-hexane (15 mL) and stored at –30 °C. The product **4-Dy** was obtained as yellow crystals (351 mg, 0.21 mmol, 79%). Anal. Calcd. for $C_{49}H_{49}AlBrDyF_{36}O_4$: C, 35.56; H, 2.98. Found: C, 33.43; H, 2.78. The paramagnetism of **4-Dy** precluded the assignment of its ^1H and $^{13}C\{^1H\}$ NMR spectra. ^{19}F NMR (376.46 MHz, C_6H_5Br, 298 K): δ = –89.0 ($\upsilon_{1/2}$ ~ 380 Hz, [Al{OC(C*F*$_3$)$_3$}$_4$]$^-$). FTIR (ATR, microcrystalline): $\tilde{\nu}$ = 2966 (m, C–H stretch), 2918 (w, C–H stretch), 2873 (w, C–H stretch), 1474 (m, C=C stretch), 1350 (s, C–O stretch), 1296 (s), 1274 (s, C–F stretch), 1239 (s, C=C stretch), 1211 (s), 1165 (s), 1058 (w), 1021 (w), 968 (s), 832 (s, Al–O stretch), 742 (s, C–H bend), 725 (s), 680 (m), 664 (m, C–Br stretch), 561 (s), 536 (s), 442 (s) cm^{-1}.

[Y(Cp^{ttt})(Cp*)(C$_6$H$_4$F$_2$-κ^2-*F,F*)][Al{OC(CF$_3$)$_3$}$_4$] (5-Y). *Ortho*-difluorobenzene (2 mL) was used to extract **1-Y** (252 mg, 0.17 mmol), the resulting yellow solution was layered with *n*-hexane (10 mL) and stored at –30 °C. The product **5-Y** was obtained as light yellow crystals (196 mg, 0.13 mmol, 74%). Anal. Calcd for $C_{49}H_{48}AlF_{38}O_4Y$: C, 38.25; H, 3.14. Found: C, 36.35; H, 3.11. ^1H NMR (400.13 MHz, $C_6H_4F_2$ with a D_2O insert, 298 K): δ = 6.30 (s, 2H, Cp-*H*), 1.72 (s, 15H, Cp-C*H*$_3$), 1.15 (s, 18H, C(C*H*$_3$)$_3$), 1.11 (s, 9H, C(C*H*$_3$)$_3$). $^{13}C\{^1H\}$ NMR (100.60 MHz, $C_6H_4F_2$ with a D_2O insert, 298 K): δ = 140.7 (d, $^1J_{CY}$ = 1.7 Hz,

Cp-*C*C(CH_3)$_3$), 140.2 (Cp-*C*C(CH_3)$_3$), 125.6 (d, $^1J_{CY}$ = 1.7 Hz, Cp-*C*(CH_3)), 115.6 (Cp-*C*H), 33.8 (*C*(CH_3)$_3$), 32.5 (*C*(CH_3)$_3$), 31.5 (C(*C*H_3)$_3$), 30.2 (C(*C*H_3)$_3$), 10.4 (Cp-C(*C*H_3)). ^{19}F NMR (376.46 MHz, $C_6H_4F_2$ with a D_2O insert, 298 K): δ = –75.8 ([Al{OC(C*F*$_3$)$_3$}$_4$]$^-$), –139.9 (C_6H_4*F*$_2$). FTIR (ATR, microcrystalline): $\tilde{\nu}$ = 2962 (m, C–H stretch), 2912 (w, C–H stretch), 2869 (w, C–H stretch), 1492 (s, C–F stretch, C=C stretch), 1352 (s, C–O stretch), 1297 (s), 1274 (s, C–F stretch), 1239 (s, C=C stretch), 1211 (s), 1165 (s), 1079 (w), 1021 (w), 970 (s), 832 (s, Al–O stretch), 750 (s, C–F stretch), 725 (s), 676 (w), 561 (s), 536 (s), 439 (s) cm^{-1}.

[Dy(Cp^{ttt})(Cp*)($C_6H_4F_2$-κ^2-*F,F*)][Al{OC(CF_3)$_3$}$_4$] (5-Dy). *Ortho*-difluorobenzene (3 mL) was used to extract **1-Dy** (400 mg, 0.27 mmol), the resulting yellow solution was layered with *n*-hexane (15 mL) and stored at –30 °C. The product **5-Dy** was obtained as yellow crystals (375 mg, 0.23 mmol, 86%). Anal. Calcd for $C_{49}H_{48}AlDyF_{38}O_4$: C, 36.50; H, 3.00. Found: C, 34.23; H, 2.84. The paramagnetism of product **5-Dy** precluded the assignment of its ^{1}H, and ^{13}C{^{1}H} NMR spectra. ^{19}F NMR (376.46 MHz, $C_6H_4F_2$, 298 K): δ = –74.2 ($\upsilon_{1/2}$ ~ 140 Hz, [Al{OC(C*F*$_3$)$_3$}$_4$]$^-$), –148.8 ($\upsilon_{1/2}$ ~ 2550 Hz, C_6H_4*F*$_2$). FTIR (ATR, microcrystalline): $\tilde{\nu}$ = 2962 (m, C–H stretch), 2910 (w, C–H stretch), 2869 (w, C–H stretch), 1494 (s, C–F stretch, C=C stretch), 1350 (s, C–O stretch), 1296 (s), 1274 (s, C–F stretch), 1239 (s, C=C stretch), 1211 (s), 1165 (s), 1081 (w), 1021 (w), 970 (s), 832 (s, Al–O stretch), 806 (m, C–H bend), 750 (s, C–F stretch), 725 (s), 674 (w), 561 (s), 536 (s), 442 (s) cm^{-1}.

[Y(Cp^{ttt})(Cp*)(PhF-κ-*F*)$_2$][Al{OC(CF_3)$_3$}$_4$] (6-Y). Fluorobenzene (2 mL) was used to extract **1-Y** (154 mg, 0.11 mmol), the resulting yellow solution was layered with *n*-hexane (10 mL) and stored at room temperature. The product **6-Y** was obtained as light yellow crystals (131 mg, 0.08 mmol, 77%). FTIR (ATR, microcrystalline): $\tilde{\nu}$ = 2966 (m, C–H stretch), 2914 (w, C–H stretch), 2869 (w, C–H stretch), 1485 (s, C=C stretch), 1352 (s, C–O stretch), 1299 (s), 1272 (s, C–F stretch), 1237 (s, C=C stretch), 1211 (s), 1165 (s), 1114 (s, C–F stretch), 1021

(w), 1019 (w), 970 (s), 832 (s, Al–O stretch), 787 (br. s, C–F stretch), 756 (s, C–H bend), 725 (s), 678 (m), 559 (s), 536 (s), 439 (s) cm^{-1}.

[Dy(Cp^{ttt})(Cp*)(THF)][Al{OC(CF$_3$)$_3$}$_4$] (7-Dy). Crystals of **7-Dy** formed concurrently with those of **2-Dy** following a preliminary recrystallization attempt. It was found that a trace amount of THF was present in the *n*-hexane used to layer the fluorobenzene.

[Dy(Cp^{ttt})(Cp*)(THF)$_2$][Al{OC(CF$_3$)$_3$}$_4$] (8-Dy). THF (2 mL) was used to extract a small portion of **1-Dy** (< 50 mg), the resulting yellow solution was layered with *n*-hexane (10 mL) and stored at –30 °C. A small amount of yellow crystals of **8-Dy** were afforded.

[{Dy(Cp^{ttt})(Cp*)}$_2$(*μ*-BH$_4$)][Al{OC(CF$_3$)$_3$}$_4$] (9-Dy). Following a small scale reaction of [Dy(Cp^{ttt})(Cp*)(BH$_4$)] (55 mg, 0.10 mmol) and [CPh$_3$][Al{OC(CF$_3$)$_3$}$_4$] (121 mg, 0.10 mmol) in *ortho*-difluorobenzene (2 mL), layering with *n*-hexane (5 mL) led to the co-crystallization of **5-Dy**, and **9-Dy** containing 0.5 eq. lattice halobenzene, thus this methodology was not repeated for further haloarenes.

3. NMR Spectroscopy

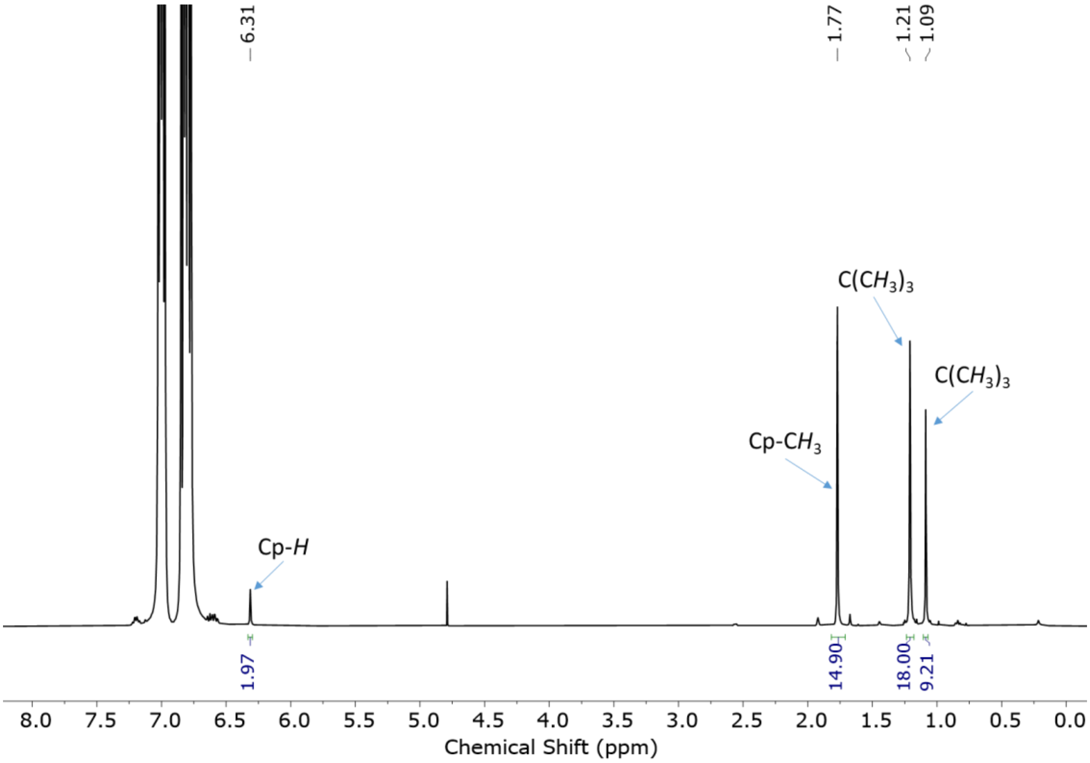

Figure S1. ^{1}H NMR spectrum of **2-Y** (400 MHz) in C_6H_5F with a D_2O insert.

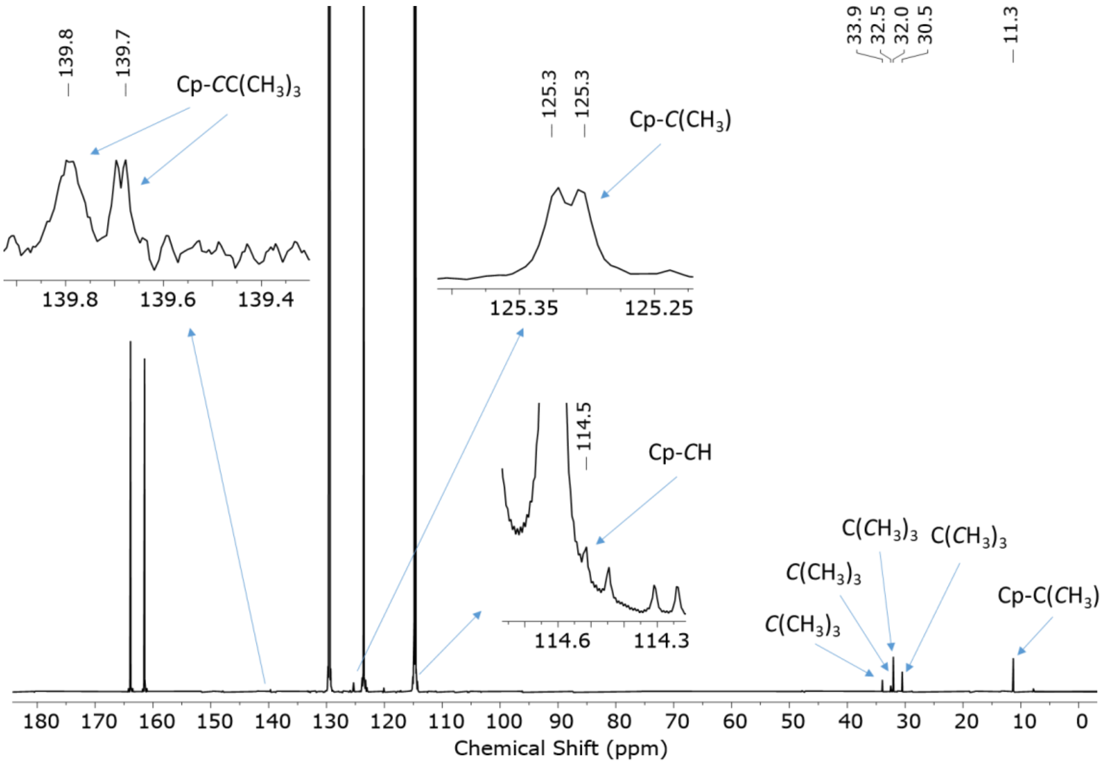

Figure S2. ^{13}C{^{1}H} NMR spectrum of **2-Y** (100 MHz) in C_6H_5F with a D_2O insert.

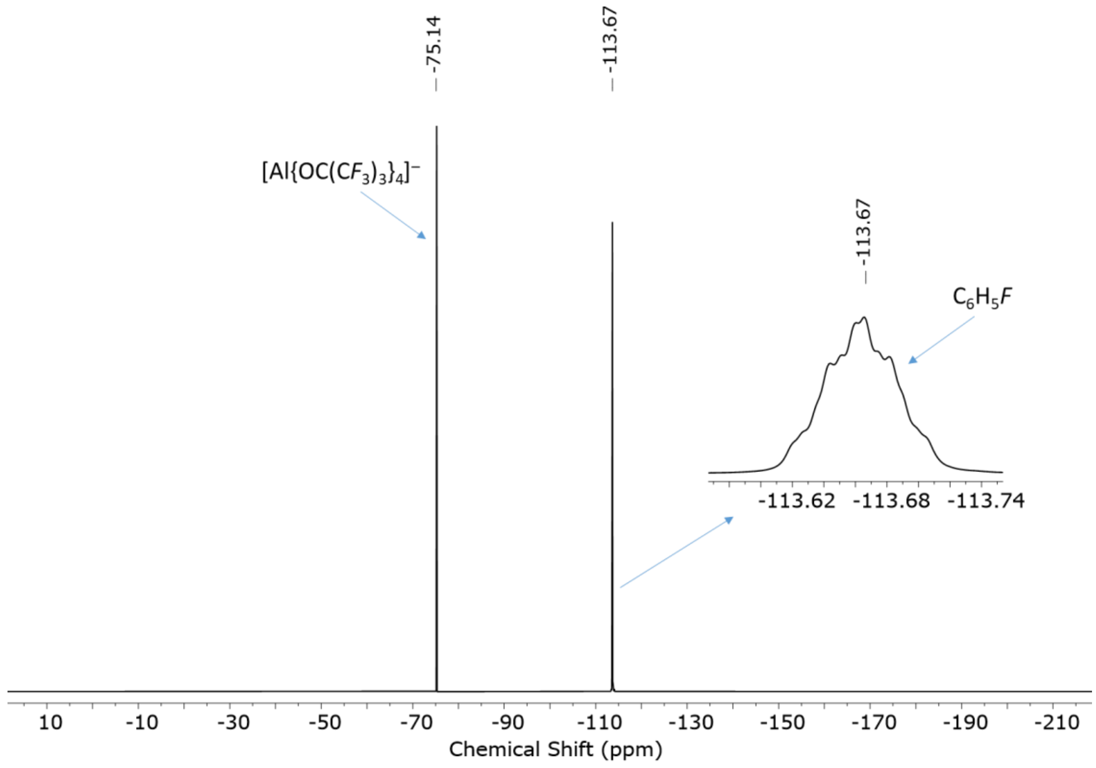

Figure S3. ^{19}F NMR spectrum of **2-Y** (376 MHz) in C_6H_5F with a D_2O insert.

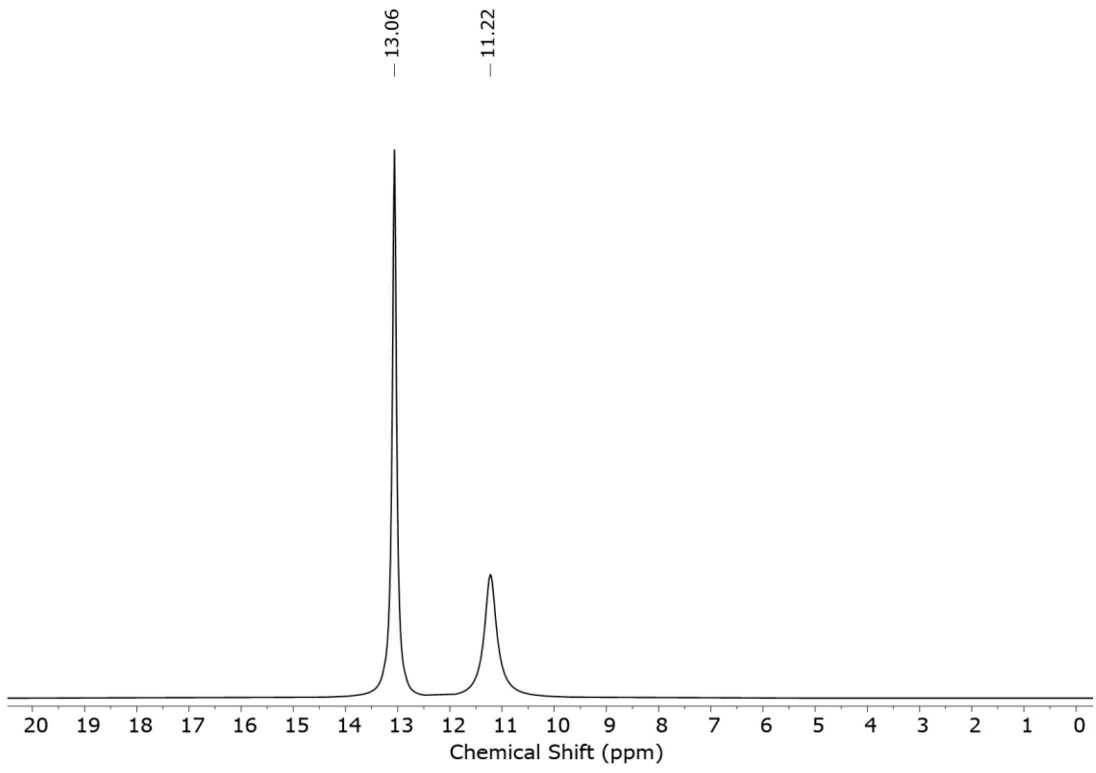

Figure S4. ^{1}H NMR spectrum of **2-Dy** (400 MHz) in C_6H_5F; full spectral range 200 to –200 ppm.

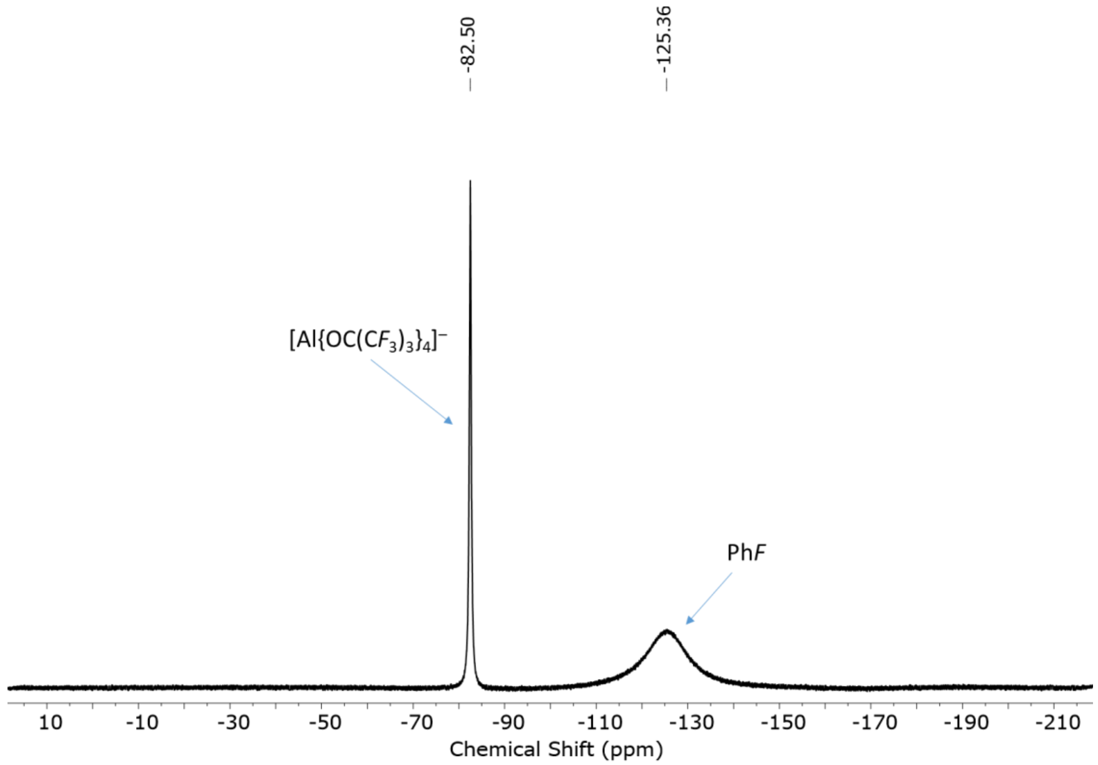

Figure S5. ^{19}F NMR spectrum of **2-Dy** (376 MHz) in C_6H_5F.

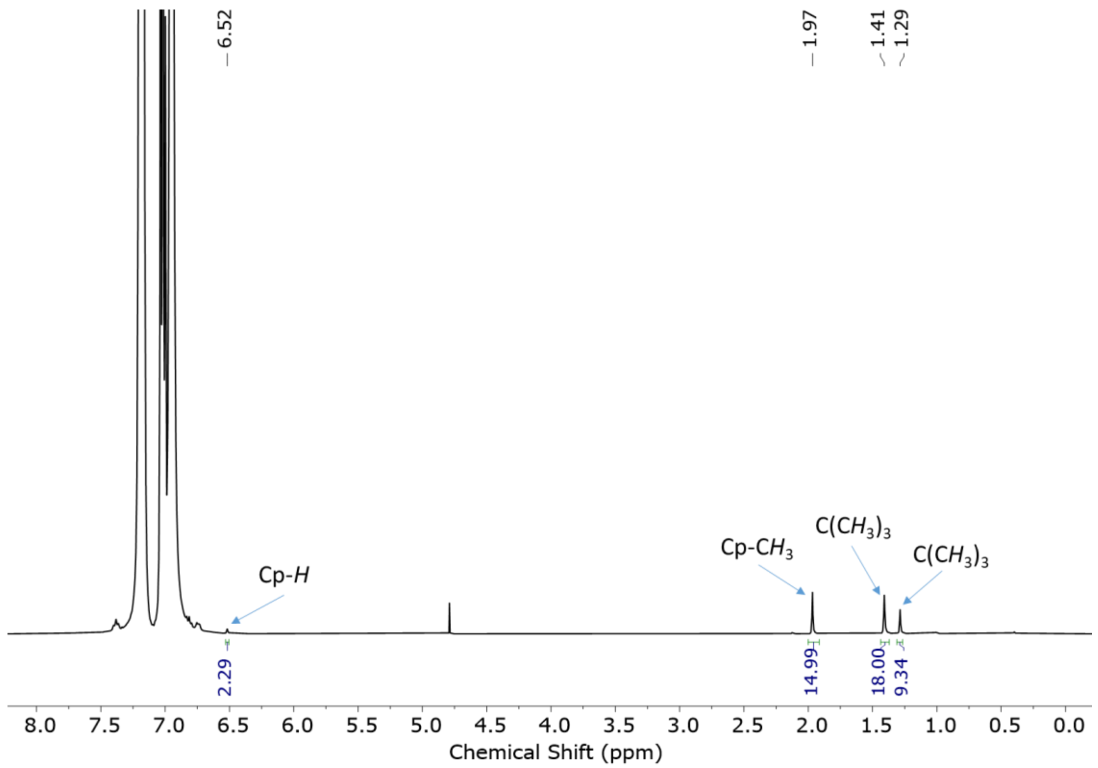

Figure S6. ^{1}H NMR spectrum of **5%Dy@2-Y** (400 MHz) in C_6H_5F with a D_2O insert.

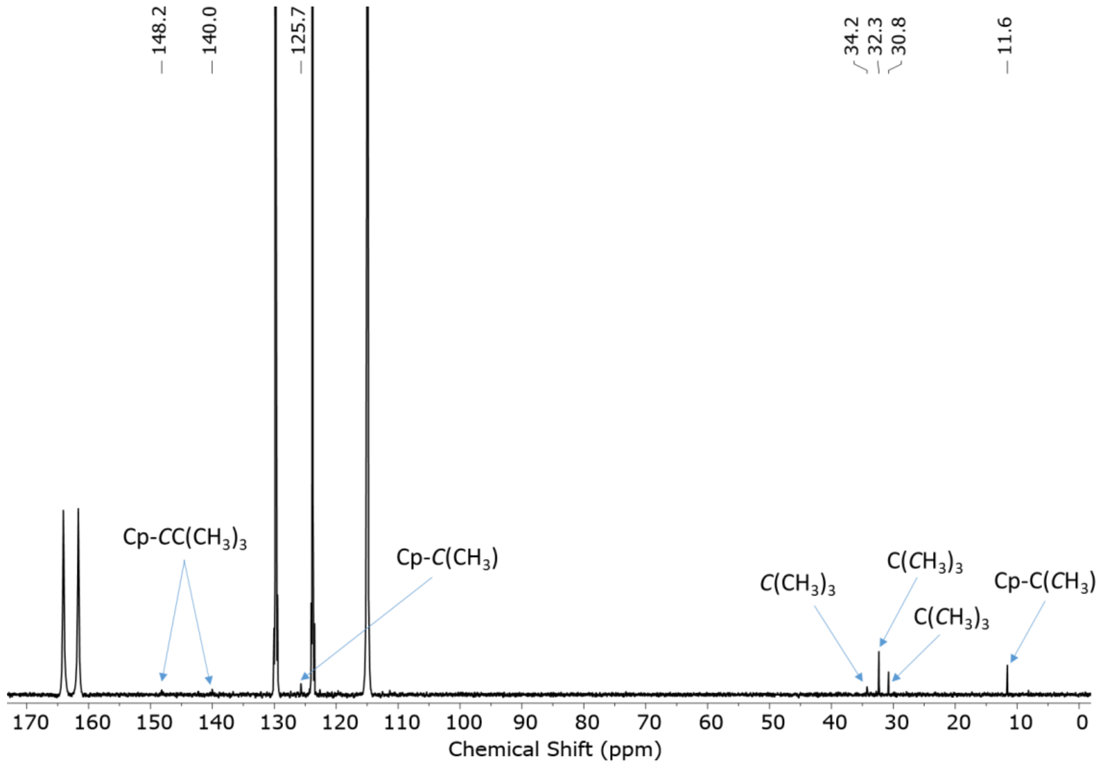

Figure S7. $^{13}C\{^{1}H\}$ NMR spectrum of **5%Dy@2-Y** (100 MHz) in C_6H_5F with a D_2O insert.

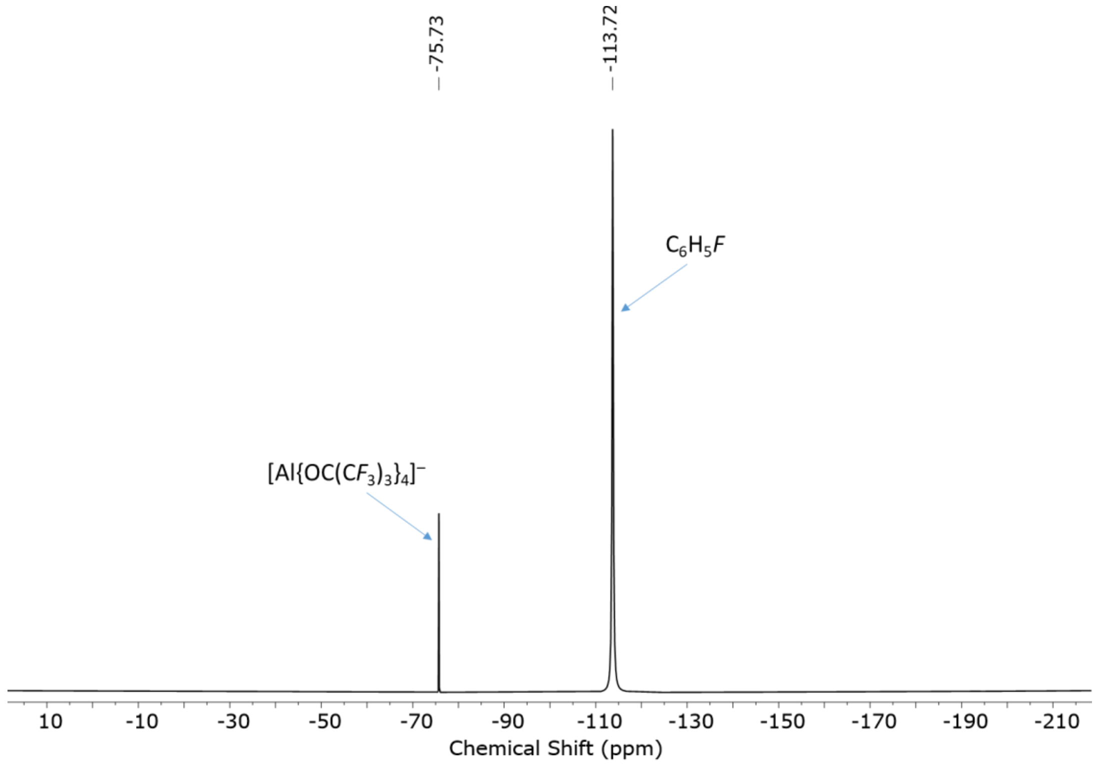

Figure S8. ^{19}F NMR spectrum of **5%Dy@2-Y** (376 MHz) in C_6H_5F with a D_2O insert.

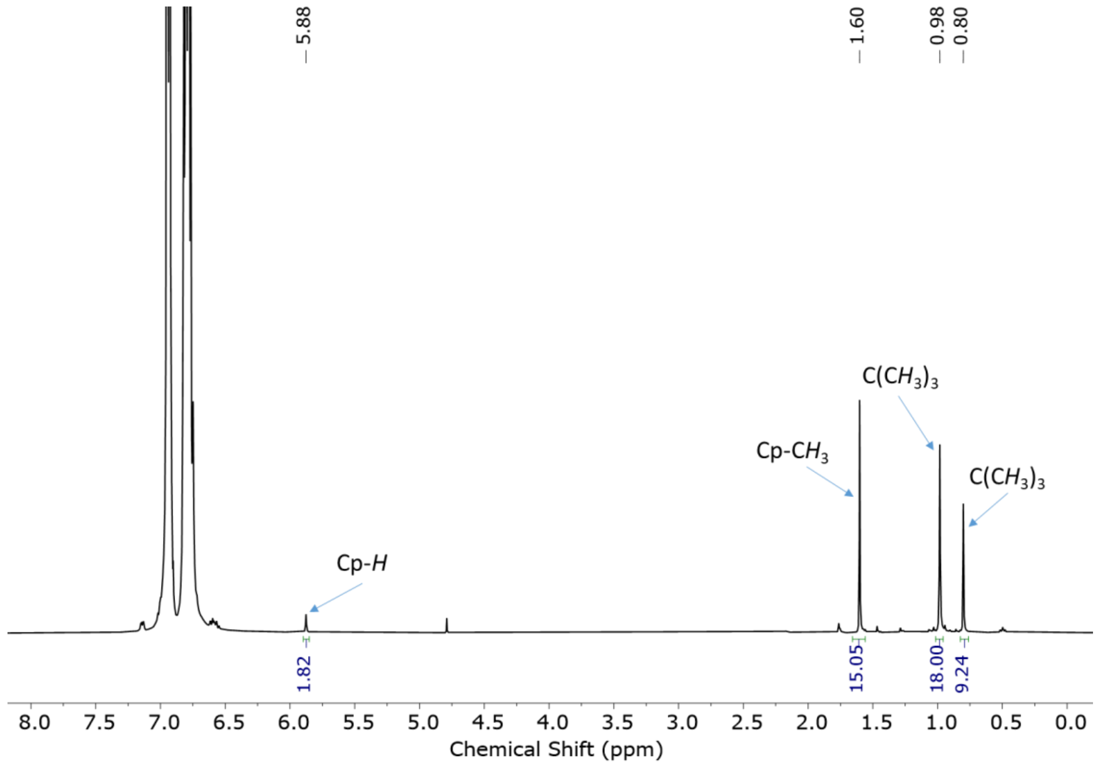

Figure S9. ^{1}H NMR spectrum of **3-Y** (400 MHz) in C_6H_5Cl with a D_2O insert.

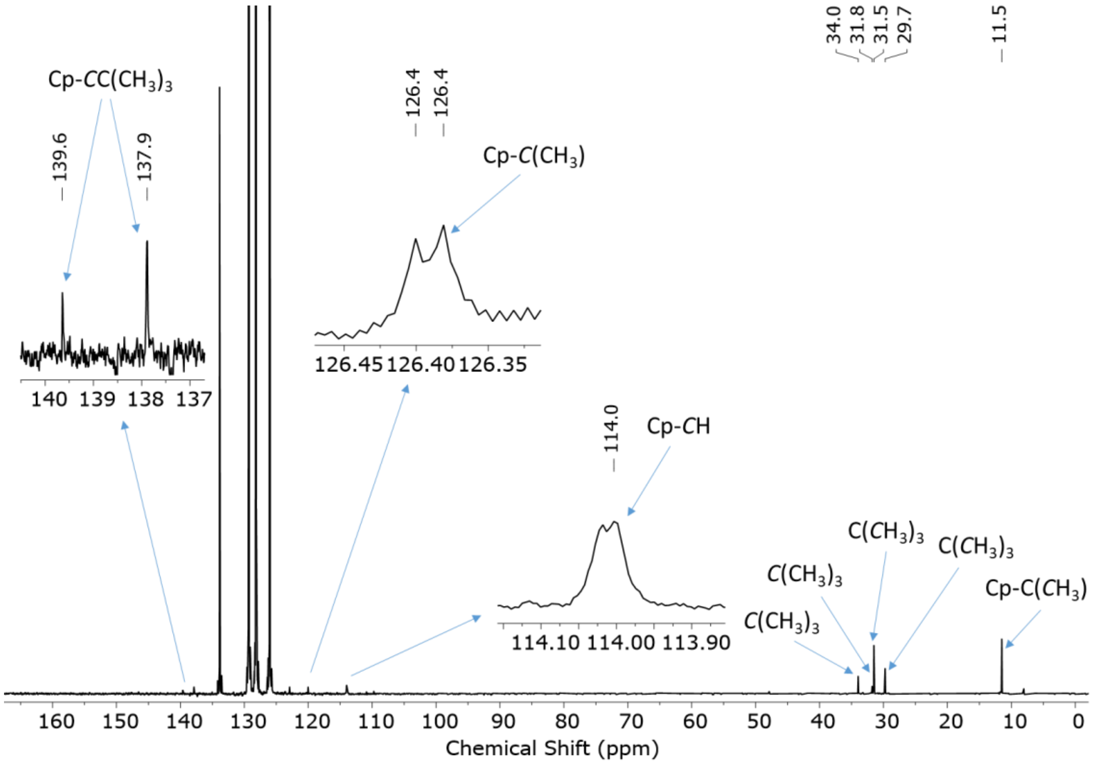

Figure S10. $^{13}C\{^{1}H\}$ NMR spectrum of **3-Y** (100 MHz) in C_6H_5Cl with a D_2O insert.

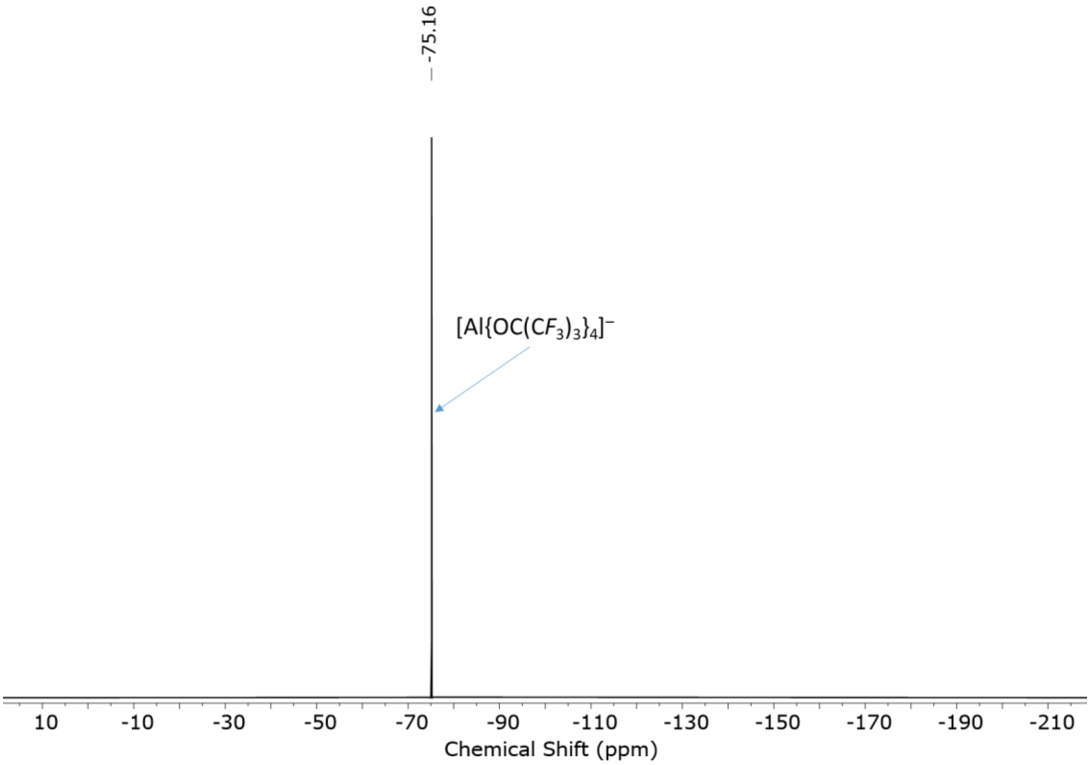

Figure S11. ^{19}F NMR spectrum of **3-Y** (376 MHz) in C_6H_5Cl with a D_2O insert.

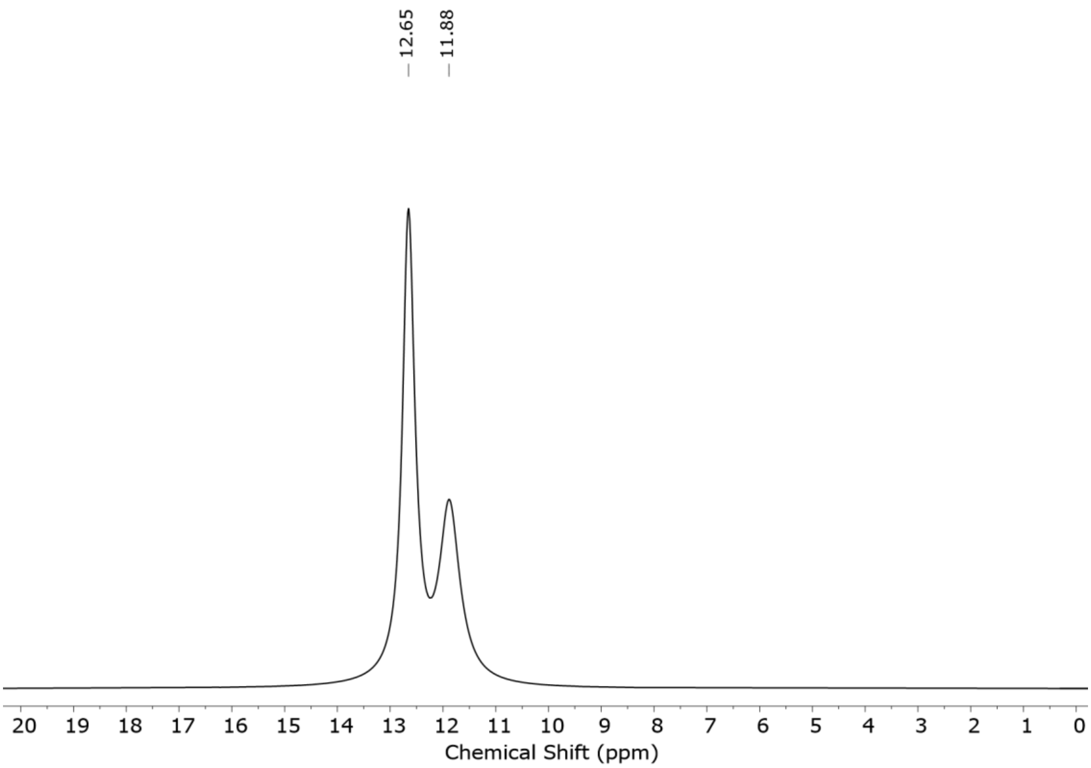

Figure S12. ^{1}H NMR spectrum of **3-Dy** (400 MHz) in C_6H_5Cl; full spectral range 200 to –200 ppm.

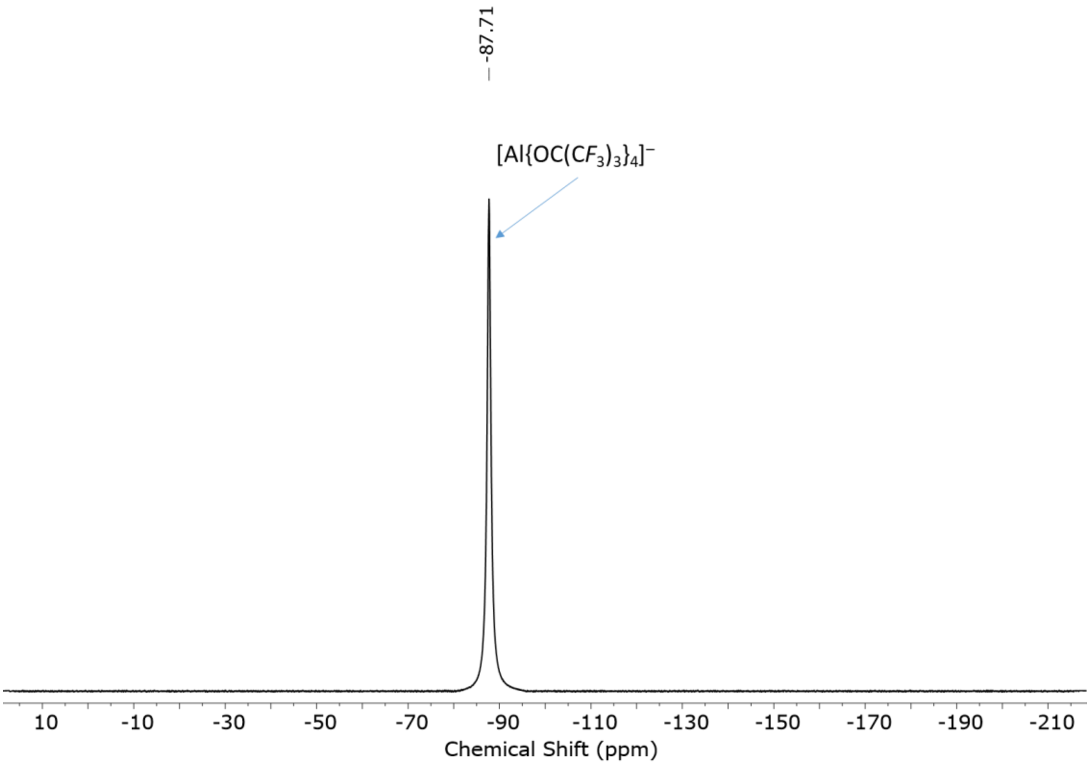

Figure S13. ^{19}F NMR spectrum of **3-Dy** (376 MHz) in C_6H_5Cl.

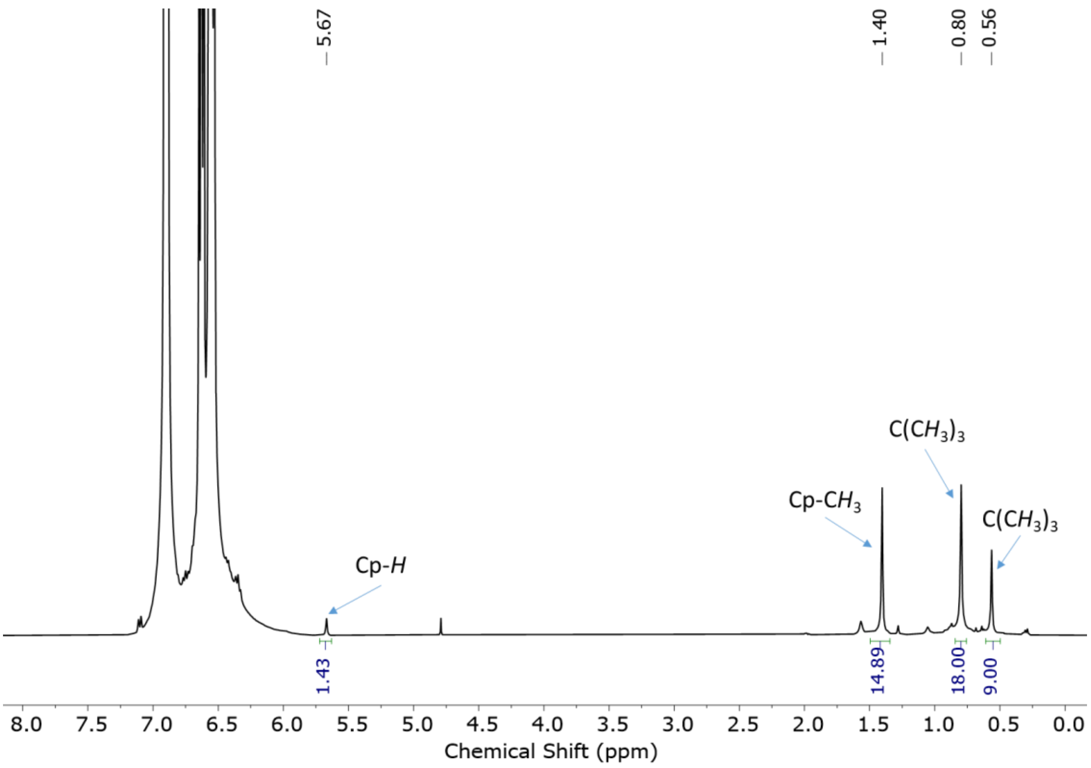

Figure S14. ^{1}H NMR spectrum of **4-Y** (400 MHz) in C_6H_5Br with a D_2O insert.

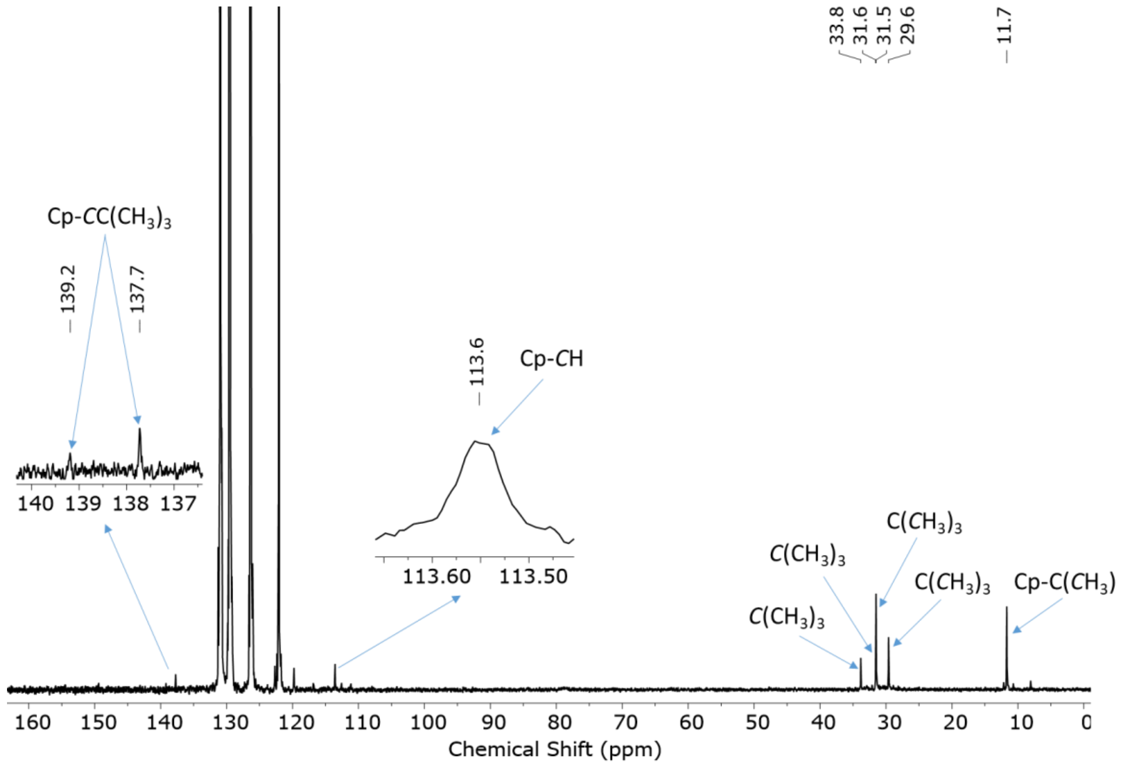

Figure S15. $^{13}C\{^{1}H\}$ NMR spectrum of **4-Y** (100 MHz) in C_6H_5Br with a D_2O insert.

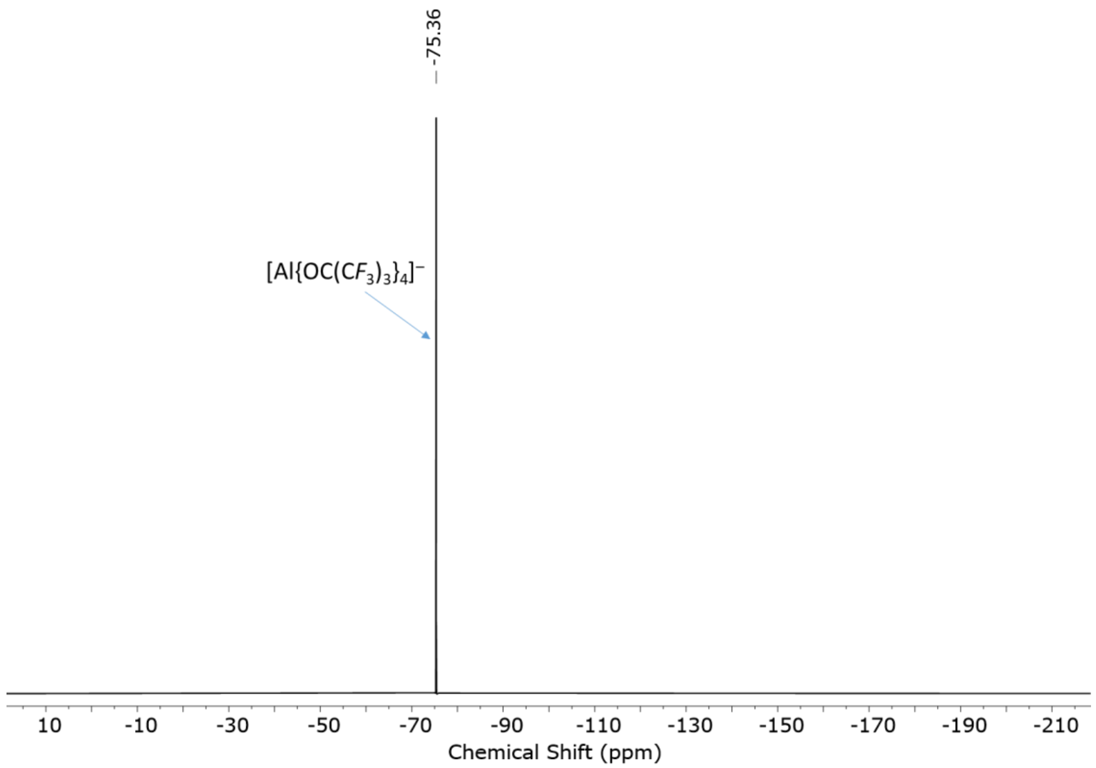

Figure S16. ^{19}F NMR spectrum of **4-Y** (376 MHz) in C_6H_5Br with a D_2O insert.

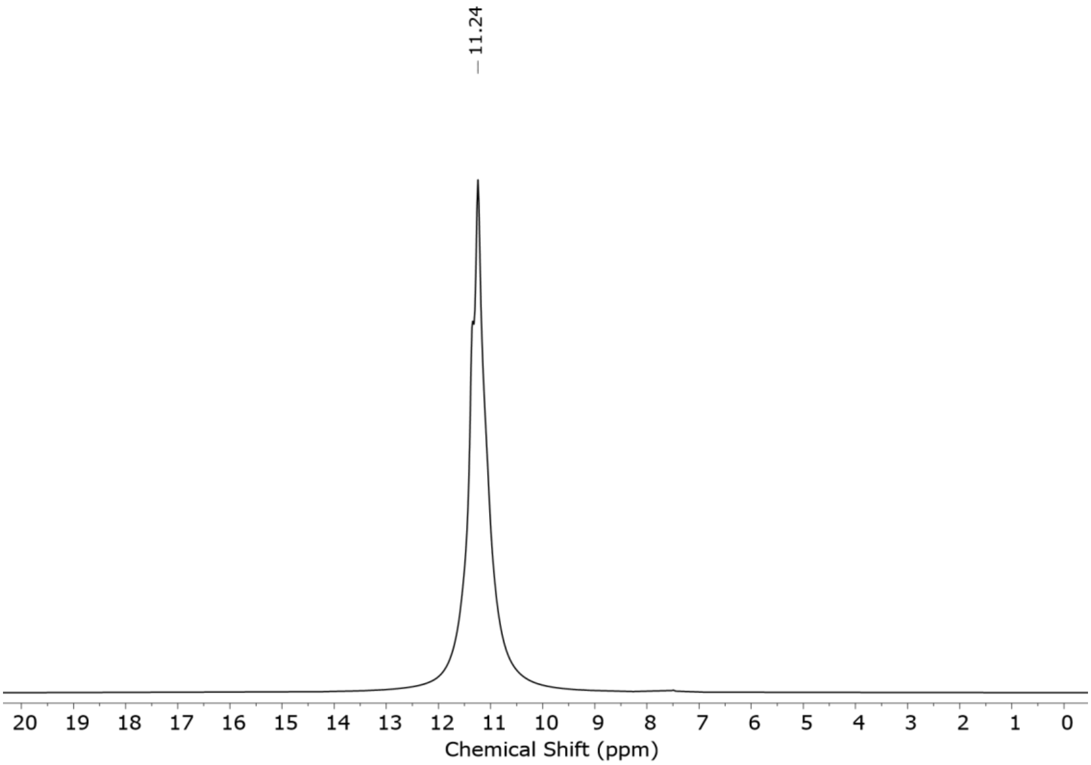

Figure S17. ^{1}H NMR spectrum of **4-Dy** (400 MHz) in C_6H_5Br; full spectral range 200 to –200 ppm.

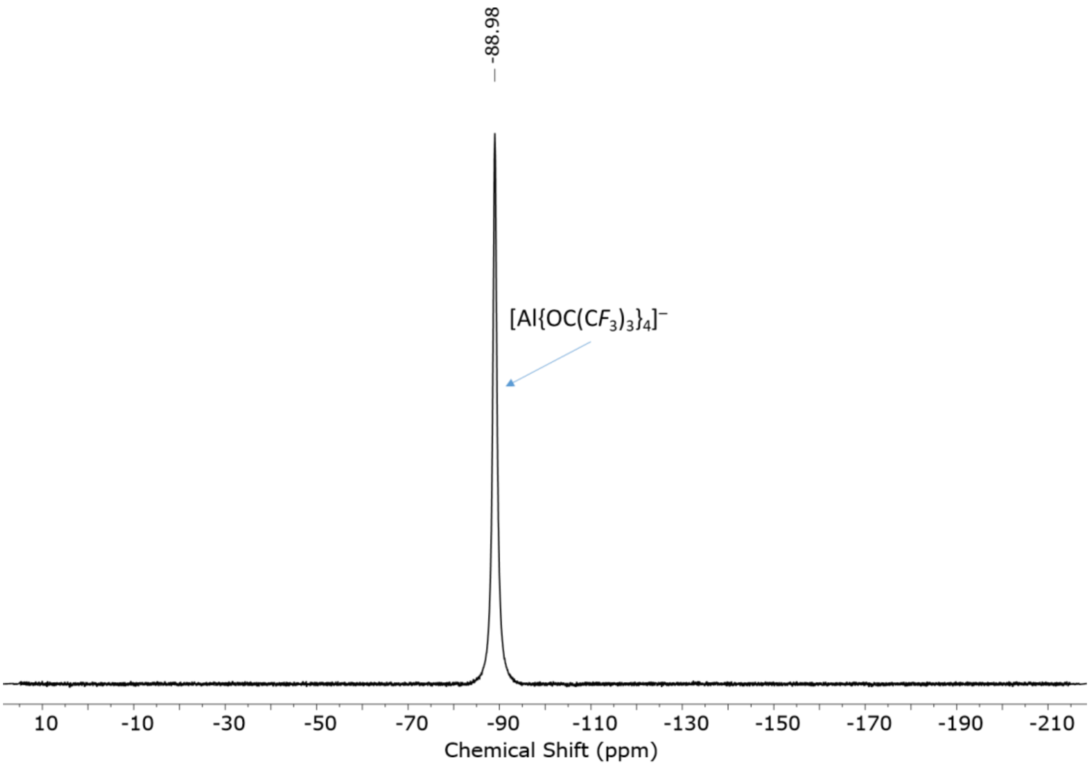

Figure S18. ^{19}F NMR spectrum of **4-Dy** (376 MHz) in C_6H_5Br.

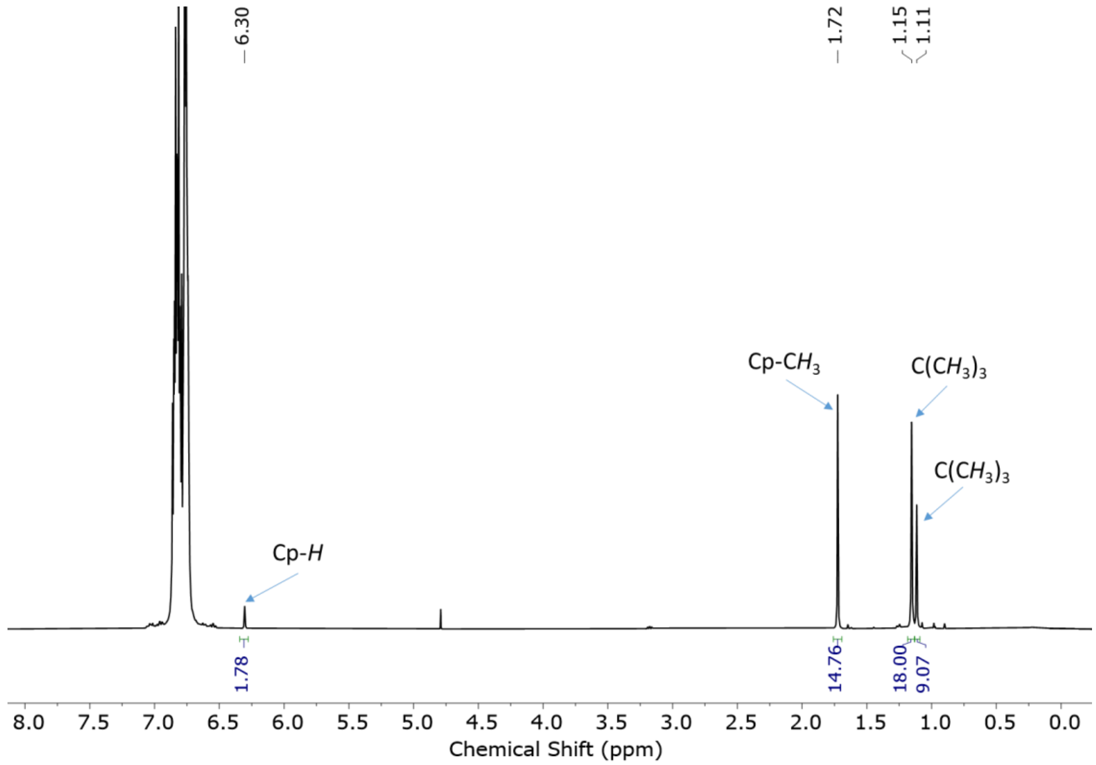

Figure S19. ^{1}H NMR spectrum of **5-Y** (400 MHz) in $C_6H_4F_2$ with a D_2O insert.

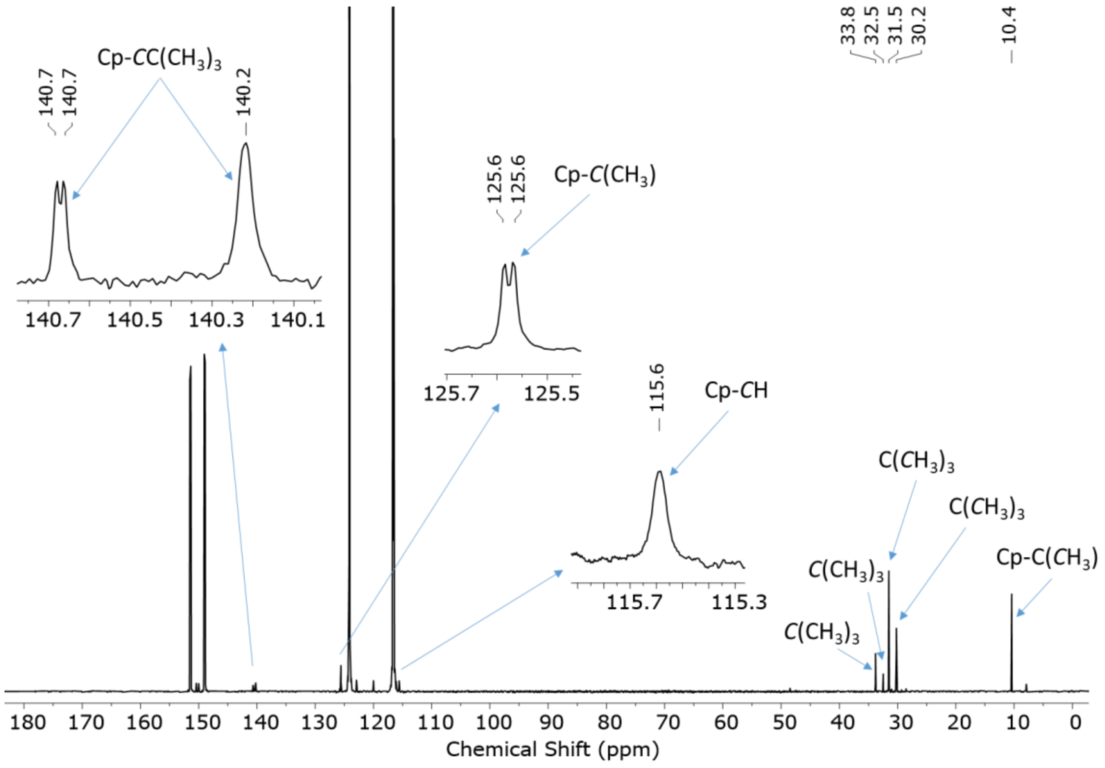

Figure S20. $^{13}C\{^{1}H\}$ NMR spectrum of **5-Y** (100 MHz) in $C_6H_4F_2$ with a D_2O insert.

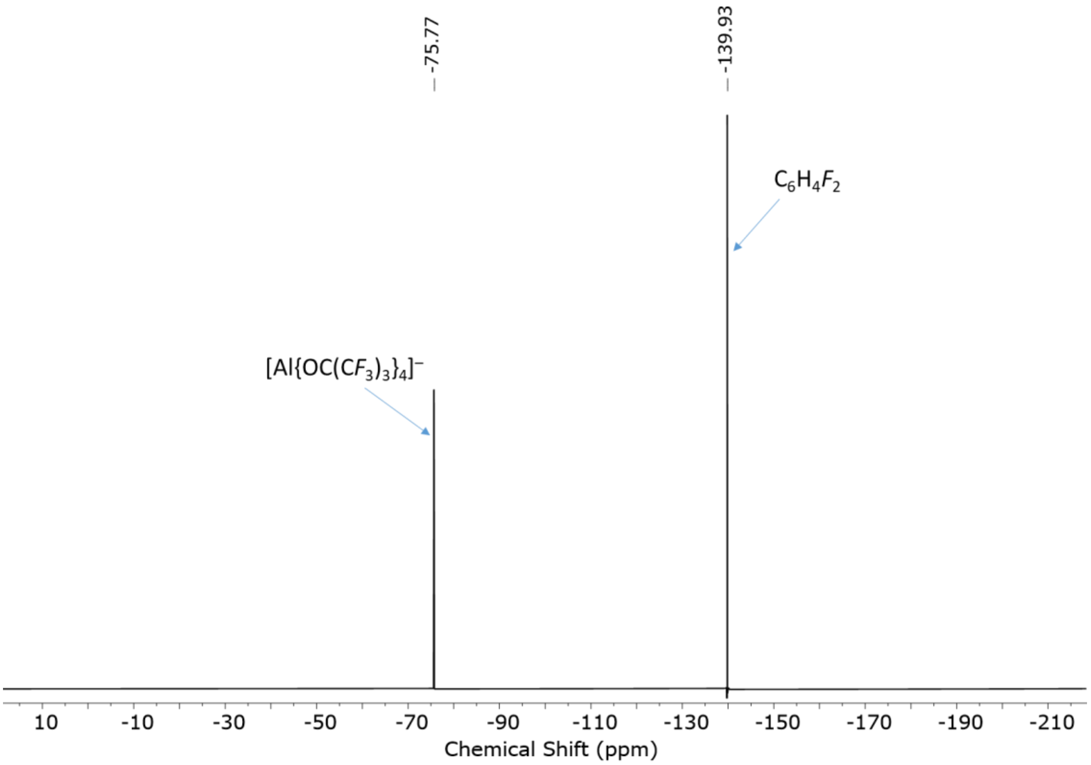

Figure S21. ^{19}F NMR spectrum of **5-Y** (376 MHz) in $C_6H_4F_2$ with a D_2O insert.

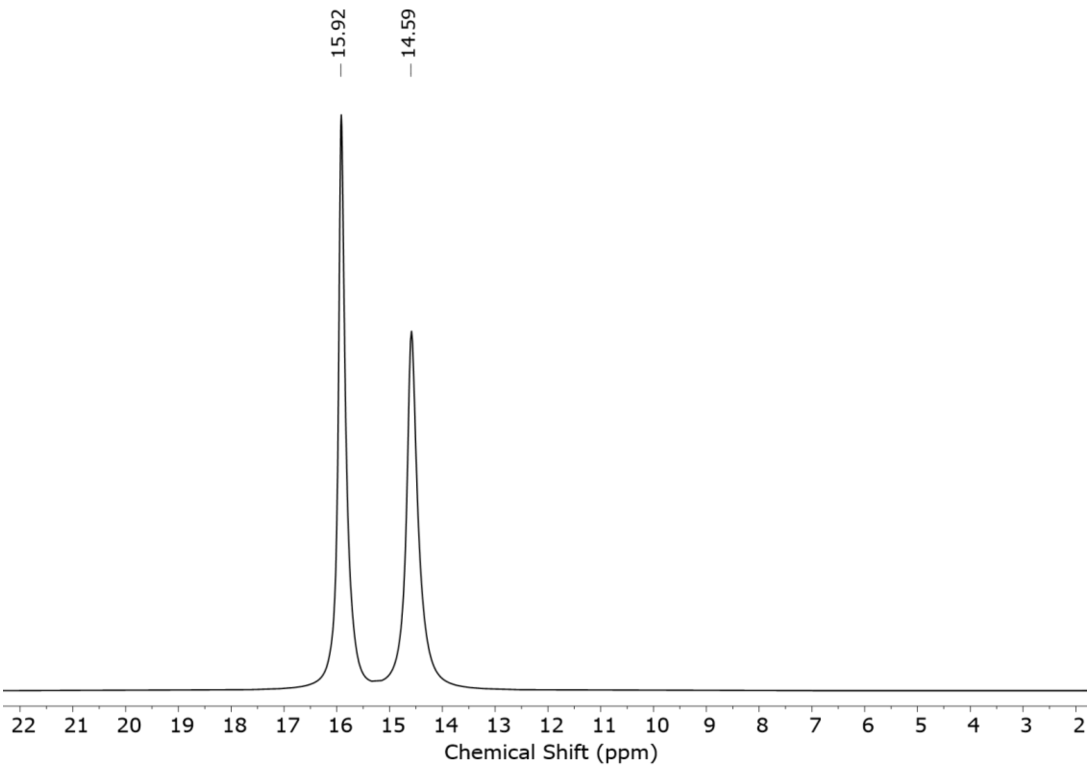

Figure S22. ^{1}H NMR spectrum of **5-Dy** (400 MHz) in $C_6H_4F_2$; full spectral range 200 to –200 ppm.

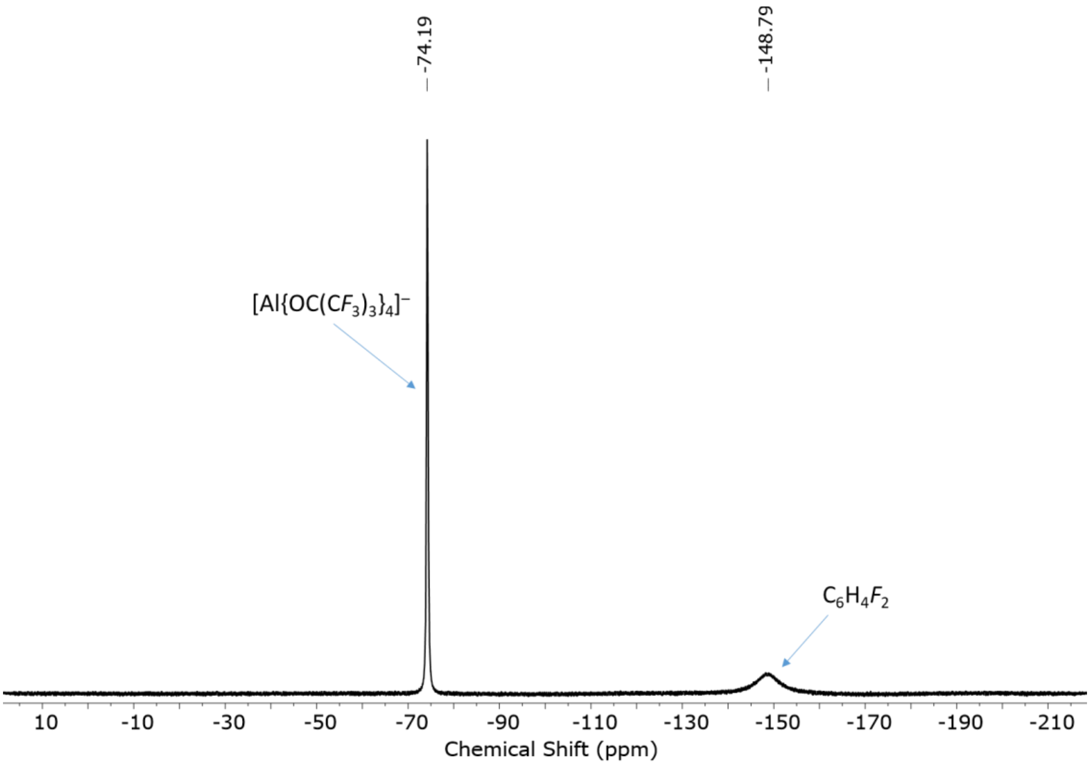

Figure S23. ^{19}F NMR spectrum of **5-Dy** (376 MHz) in $C_6H_4F_2$.

4. Powder X-ray Diffraction

Data collection. X-ray diffraction data of microcrystalline samples of **2-Dy**, **3-Dy**, **4-Dy** and **5-Dy** mounted with a minimum amount of fomblin were collected at 100 K using a Rigaku FR-x rotating anode single crystal X-ray diffractometer using Cu Kα radiation (λ = 1.5418 Å) with a Hypix-6000HE detector and an Oxford Cryosystems nitrogen flow gas system. Data were collected between 3–70 °2θ, with a detector distance of 150 mm and a beam divergence of 1.5 mRad.[5] X-ray data were collected using CrysAlisPro software.[6]

Data processing. The instrument was calibrated using silver behenate as standard. Then, X-ray data were reduced and integrated using CrysAlisPro software.[6] Pawley refinement with the unit cells obtained from the crystal structures were performed using TOPAS software.[7,8]

Data analysis. The data are presented in Figures S22–S29 and Table S1. The peaks of the diffractograms are generally in excellent agreement with the peak positions calculated; the only two significant unfitted peaks are at 2θ = 9.4° for **3-Dy** and 9.3° for **5-Dy**. The low number and intensity of unfitted peaks provides confidence that **2-Dy**, **3-Dy**, **4-Dy** and **5-Dy** show high phase purities. The Pawley refinements qualitatively provide an excellent match with the experimental data.

Table S1. Unit cell values obtained from Pawley refinement results.

Compound	a	b	c	α	β	γ
**2-Dy**	9.541(5)	26.84(1)	24.17(4)	90	95.03(1)	90
**3-Dy**	9.681(3)	26.52(1)	24.391(8)	90	91.95(1)	90
**4-Dy**	11.375(3)	15.178(4)	35.592(8)	90	90.993(5)	90
**5-Dy**	13.516(3)	10.323(2)	21.369(4)	90	94.566(5)	90

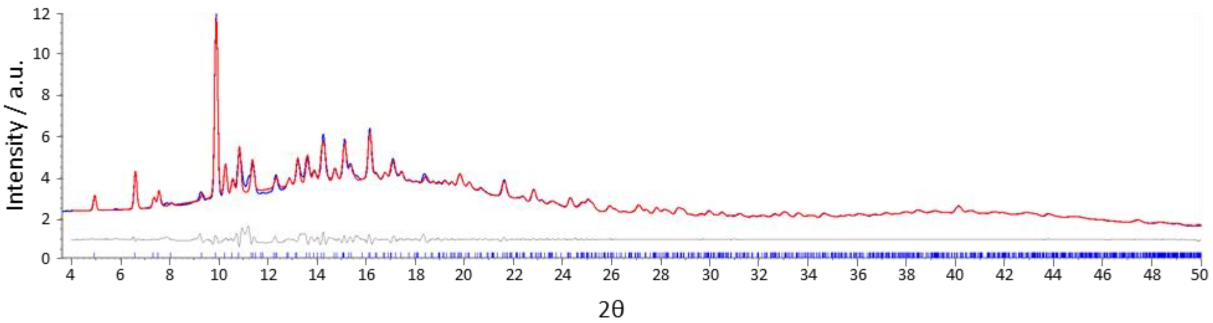

Figure S24. Pawley refinement analysis of **2-Dy**; experimental data (blue), calculated model from crystallographic parameters (red) and the difference (gray). Pawley refinement Rwp = 2.693; Rwp′ = 7.993. Pawley refinement was determined using the unit cell values from the crystal structure.

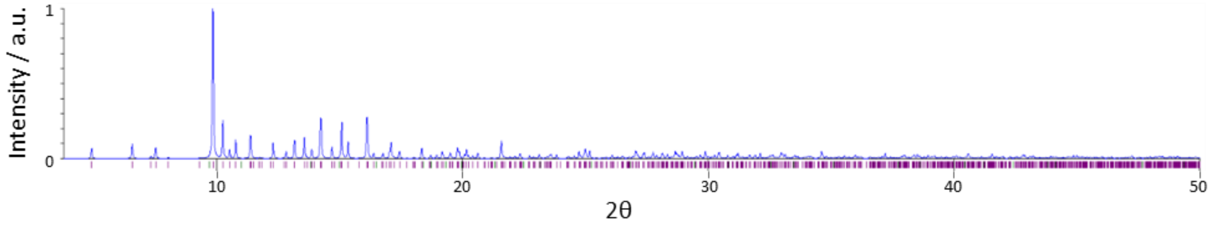

Figure S25. Theoretical powder X-ray diffraction pattern of **2-Dy** derived from crystallographic parameters.

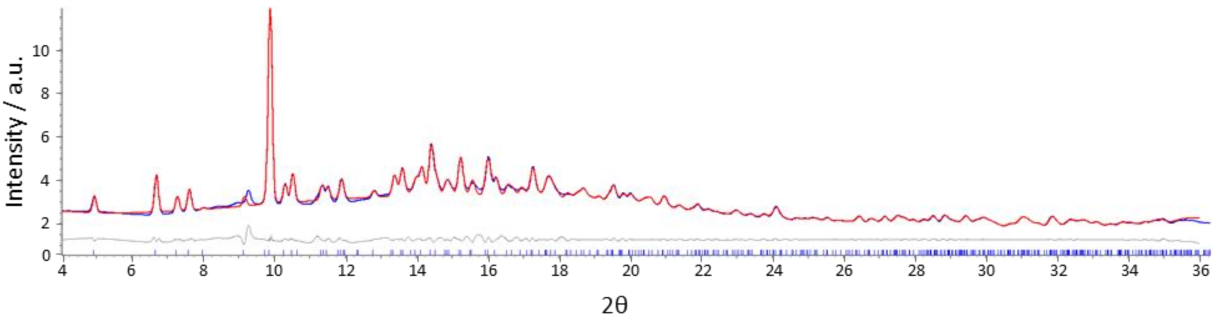

Figure S26. Pawley refinement analysis of **3-Dy**; experimental data (blue), calculated model from crystallographic parameters (red) and the difference (gray). Pawley refinement Rwp = 2.487; Rwp′ = 9.687. Pawley refinement was determined using the unit cell values from the crystal structure.

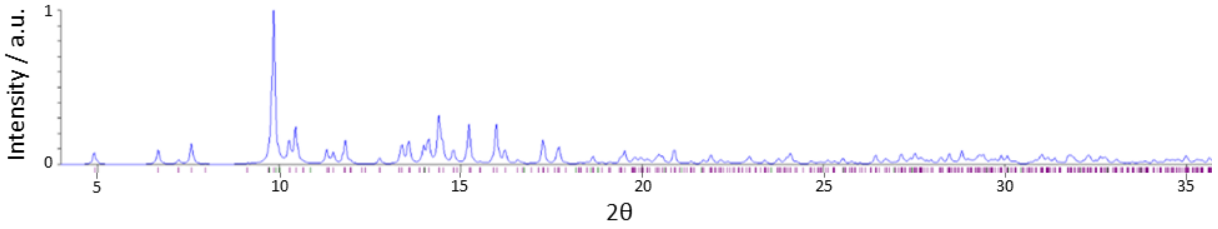

Figure S27. Theoretical powder X-ray diffraction pattern of **3-Dy** derived from crystallographic parameters.

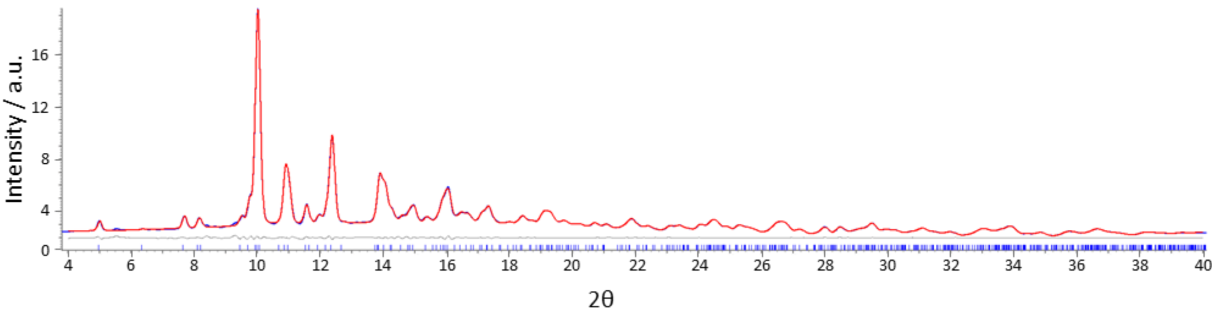

Figure S28. Pawley refinement analysis of **4-Dy**; experimental data (blue), calculated model from crystallographic parameters (red) and the difference (gray). Pawley refinement Rwp = 0.992; Rwp′ = 3.036. Pawley refinement was determined using the unit cell values from the crystal structure.

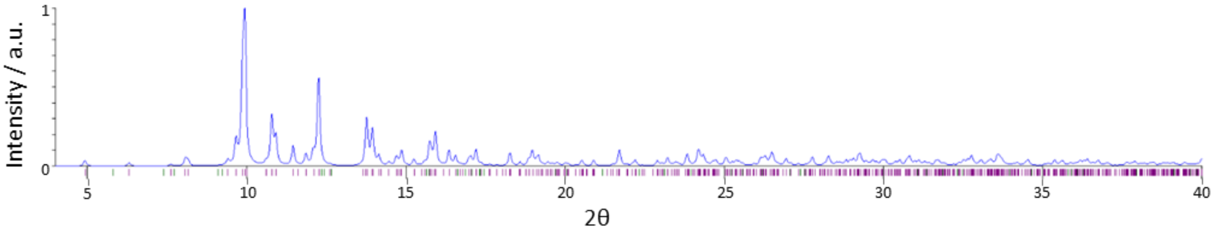

Figure S29. Theoretical powder X-ray diffraction pattern of **4-Dy** derived from crystallographic parameters.

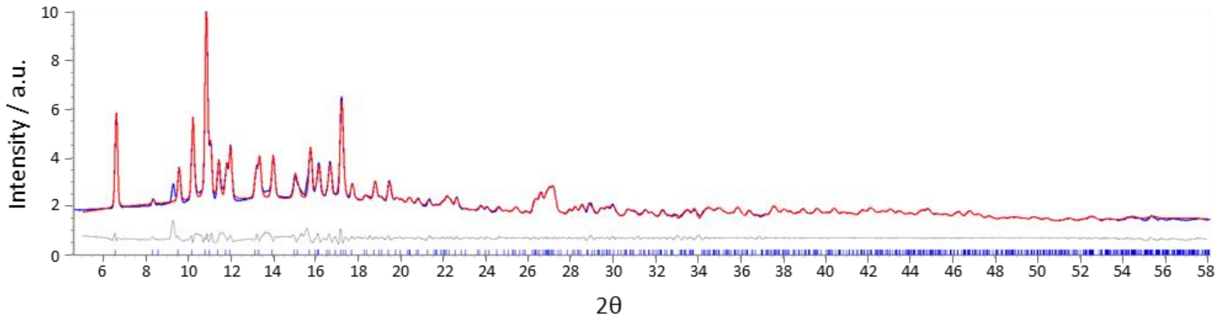

Figure S30. Pawley refinement analysis of **5-Dy**; experimental data (blue), calculated model from crystallographic parameters (red) and the difference (gray). Pawley refinement Rwp = 3.323; Rwp′ = 10.235. Pawley refinement was determined using the unit cell values from the crystal structure.

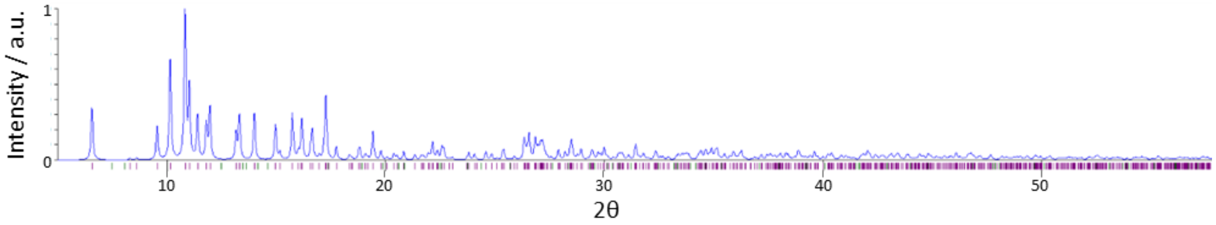

Figure S31. Theoretical powder X-ray diffraction pattern of **5-Dy** derived from crystallographic parameters.

5. Single crystal X-ray diffraction

X-ray diffraction data for compounds **2-Ln**, **3-Ln**, **4-Ln**, **5-Dy**, **6-Y** and **7-9-Dy** were collected using a dual wavelength Rigaku FR-X rotating anode diffractometer using CuKα (λ = 1.54146 Å) radiation, equipped with an AFC-11 4-circle goniometer, VariMAX^{TM} microfocus optics, a Hypix-6000HE detector and an Oxford Cryosystems 800 plus nitrogen flow gas system, at a temperature of 100, 150 or 200 K. X-Ray Data for **5-Y** were collected at a temperature of 293 K with Zr-L radiation (λ = 0.6889 Å) using the synchrotron X-Ray source at single crystal X-Ray diffraction beamline I19 in Diamond light Source,[9] equipped with an Pilatus 2M detector and an Oxford Cryosystems Cryostream 800 nitrogen flow gas system using GDA suite of programs. Data were collected and reduced using CrysAlisPro v42.[6] Absorption correction was performed using empirical methods (SCALE3 ABSPACK) based upon symmetry-equivalent reflections combined with measurements at different azimuthal angles. The structures were solved and refined against all F^2 values using Shelx-2018/3 implemented through Olex2 v1.5.[10,11] ORTEP-3[12] and POV-Ray[13] were employed for molecular graphics.

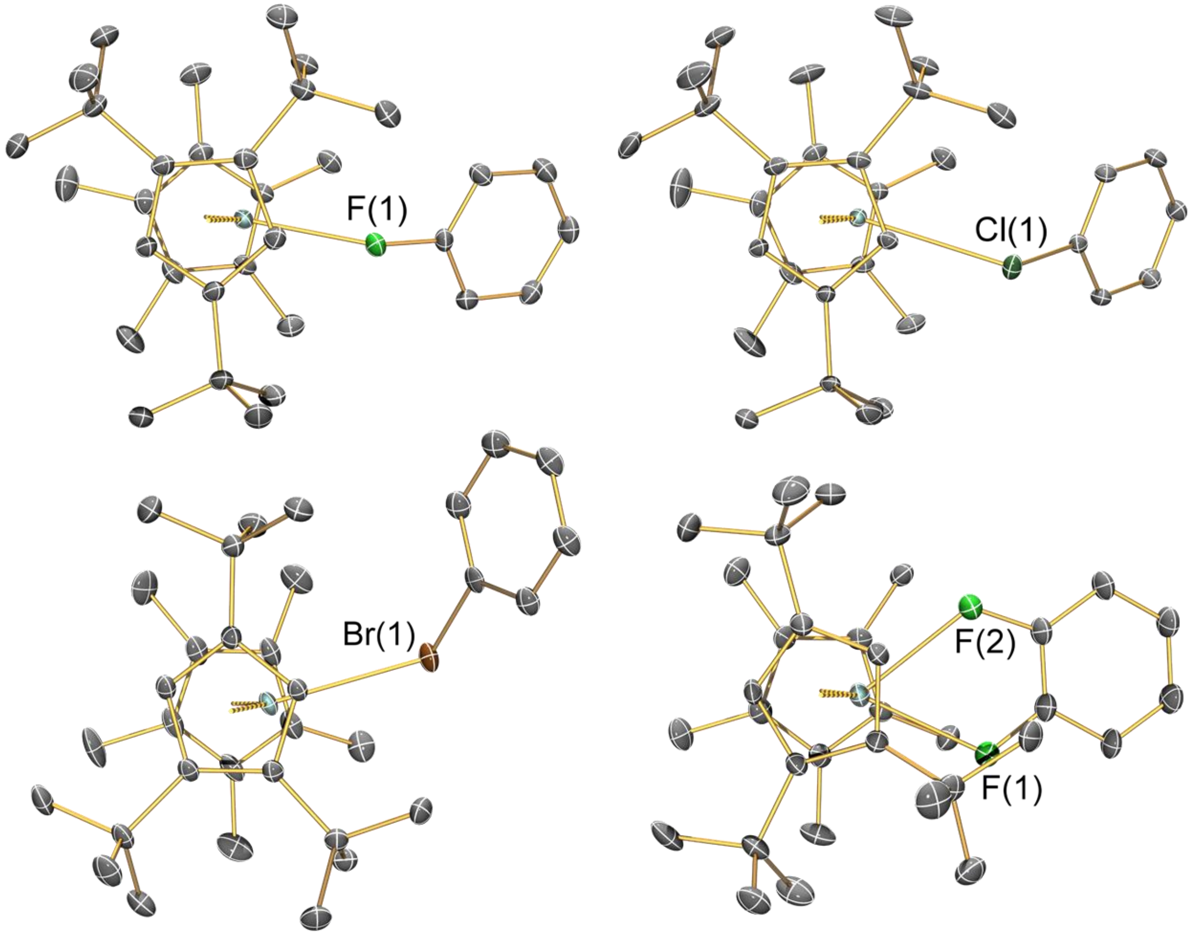

Figure S32. Top view of the SCXRD structures of **2-Dy**, **3-Dy**, **4-Dy** and **5-Dy** with selected atom labelling (Dy: cyan, C: gray, F: green, Cl: dark green, Br: brown). Displacement ellipsoids set at 30% probability levels; hydrogen atoms, lattice solvents and the $[Al\{OC(CF_3)_3\}_4]^-$ anions are omitted for clarity.

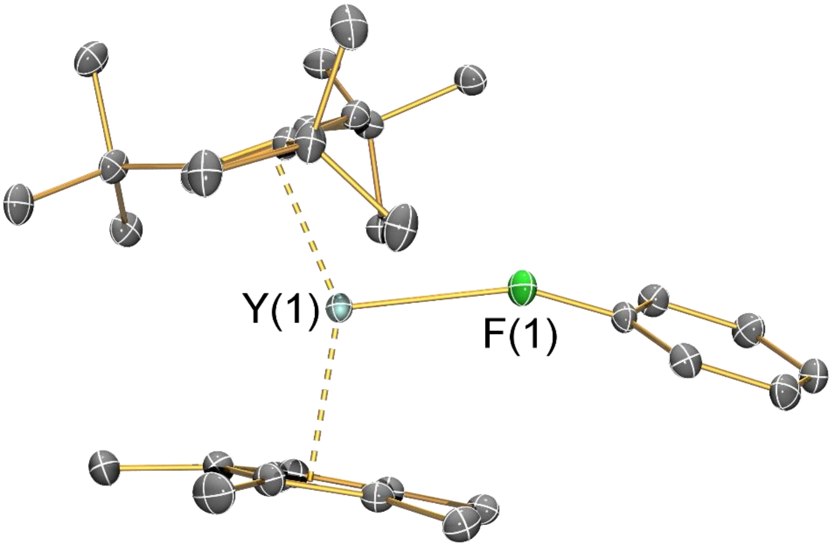

Figure S33. SCXRD structure of **2-Y** with selected atom labelling (Y: cyan, C: gray, F: green). Displacement ellipsoids set at 30% probability levels; hydrogen atoms, the lattice solvent and the $[Al\{OC(CF_3)_3\}_4]^-$ anion have been omitted for clarity. Selected bond lengths and angles: Y(1)···Cp$^{ttt}_{centroid}$ 2.297(2) Å, Y(1)···Cp*$_{centroid}$ 2.303(2) Å, Y(1)–F(1) 2.394(2) Å, Cp$^{ttt}_{centroid}$···Y(1)···Cp*$_{centroid}$ 146.97(6)°, Y(1)–F(1)–C$_{ipso}$ 153.41(9)°, Cp$^{ttt}_{centroid}$···Y(1)–F(1) 106.44(6)°, Cp*$_{centroid}$···Y(1)–F(1) 105.94(6)°.

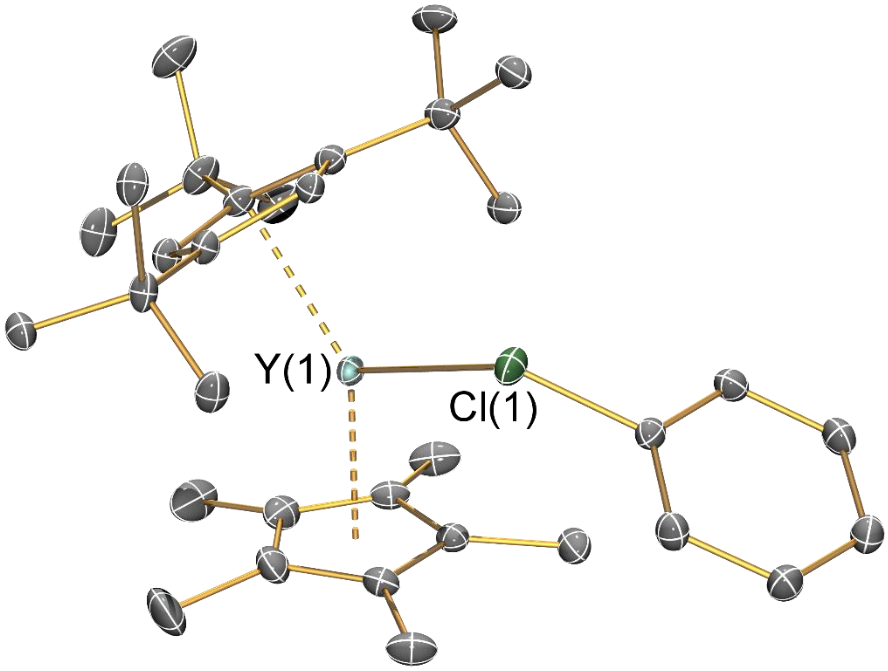

Figure S34. SCXRD structure of **3-Y** with selected atom labelling (Y: cyan, C: gray, Cl: dark green). Displacement ellipsoids set at 30% probability levels; hydrogen atoms, the lattice solvent and the $[Al\{OC(CF_3)_3\}_4]^-$ anion have been omitted for clarity. Selected bond lengths and angles: Y(1)···$Cp^{ttt}_{centroid}$ 2.303(2) Å, Y(1)···$Cp^*_{centroid}$ 2.305(2) Å, Y(1)–Cl(1) 2.8722(7) Å, $Cp^{ttt}_{centroid}$···Y(1)···$Cp^*_{centroid}$ 147.02(5)°, Y(1)–Cl(1)–C_{ipso} 126.49(3), $Cp^{ttt}_{centroid}$···Y(1)–Cl(1) 101.23(3)°, $Cp^*_{centroid}$···Y(1)–Cl(1) 109.29(4)°.

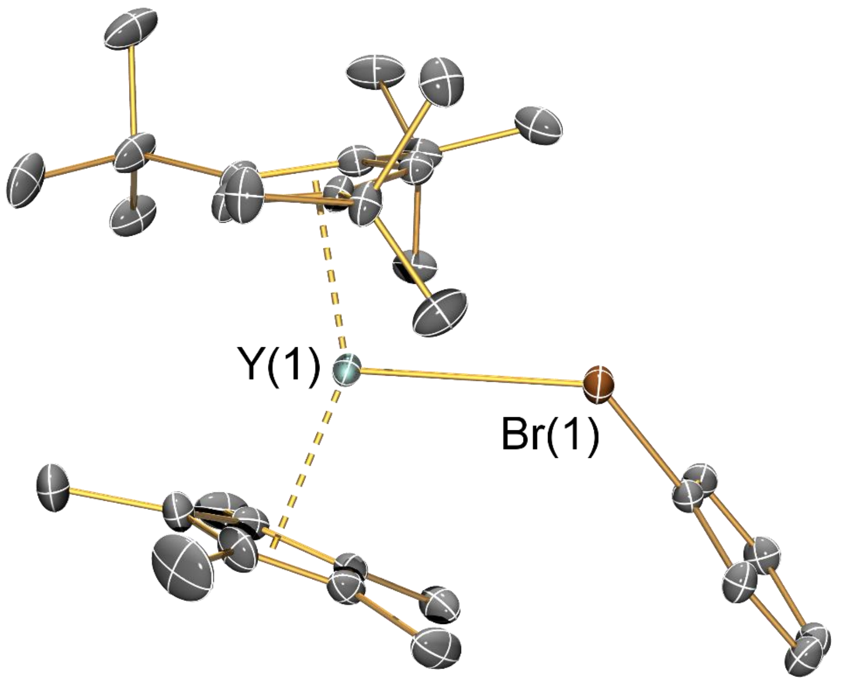

Figure S35. SCXRD structure of **4-Y** with selected atom labelling (Y: cyan, C: gray, Br: brown). Displacement ellipsoids set at 30% probability levels; hydrogen atoms, the lattice solvent and the $[Al\{OC(CF_3)_3\}_4]^-$ anion have been omitted for clarity. Selected bond lengths and angles: Y(1)···$Cp^{ttt}_{centroid}$ 2.305(2) Å, Y(1)···$Cp^*_{centroid}$ 2.309(2) Å, Y(1)–Br(1) 2.9915(7) Å, $Cp^{ttt}_{centroid}$···Y(1)···$Cp^*_{centroid}$ 147.52(5)°, Y(1)–Br(1)–C_{ipso} 123.18(3), $Cp^{ttt}_{centroid}$···Y(1)–Br(1) 99.85(3)°, $Cp^*_{centroid}$···Y(1)–Br(1) 109.84(4)°.

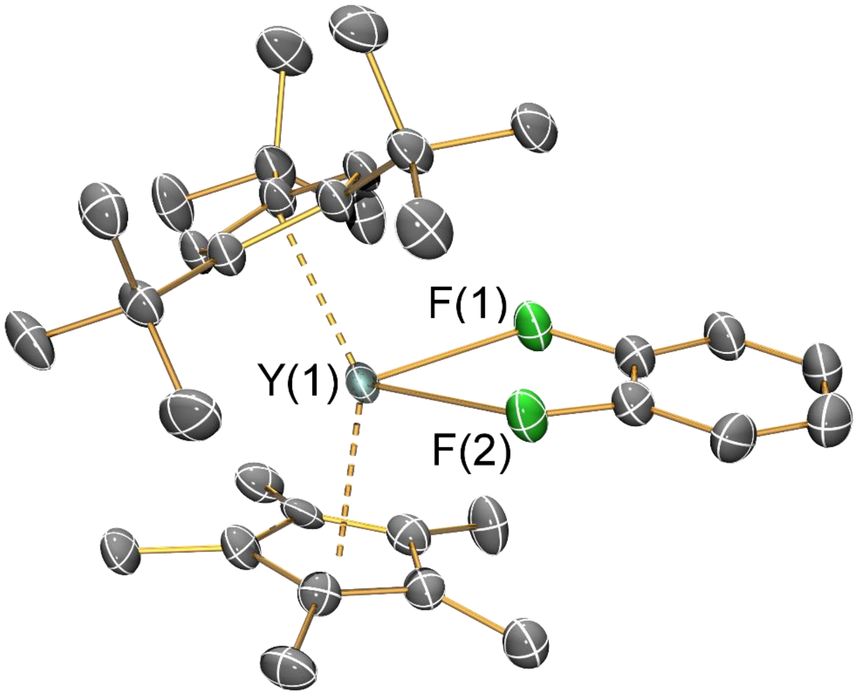

Figure S36. SCXRD structure of **5-Y** with selected atom labelling (Y: cyan, C: gray, F: green). Displacement ellipsoids set at 30% probability levels; hydrogen atoms and the $[Al\{OC(CF_3)_3\}_4]^-$ anion have been omitted for clarity. Selected bond lengths and angles: Y(1)···Cp$^{ttt}_{centroid}$ 2.322(2) Å, Y(1)···Cp*$_{centroid}$ 2.342(2) Å, Y(1)–F(1) 2.438(5) Å, Y(1)–F(2) 2.431(5) Å, Cp$^{ttt}_{centroid}$···Y(1)···Cp*$_{centroid}$ 144.39(3)°, Y(1)–F$_{2centroid}$–C$_{6centroid}$ 169.82(2), Cp$^{ttt}_{centroid}$···Y(1)–F$_{2centroid}$109.39(3)°, Cp*$_{centroid}$···Y(1)–F$_{2centroid}$ 105.06(3)°.

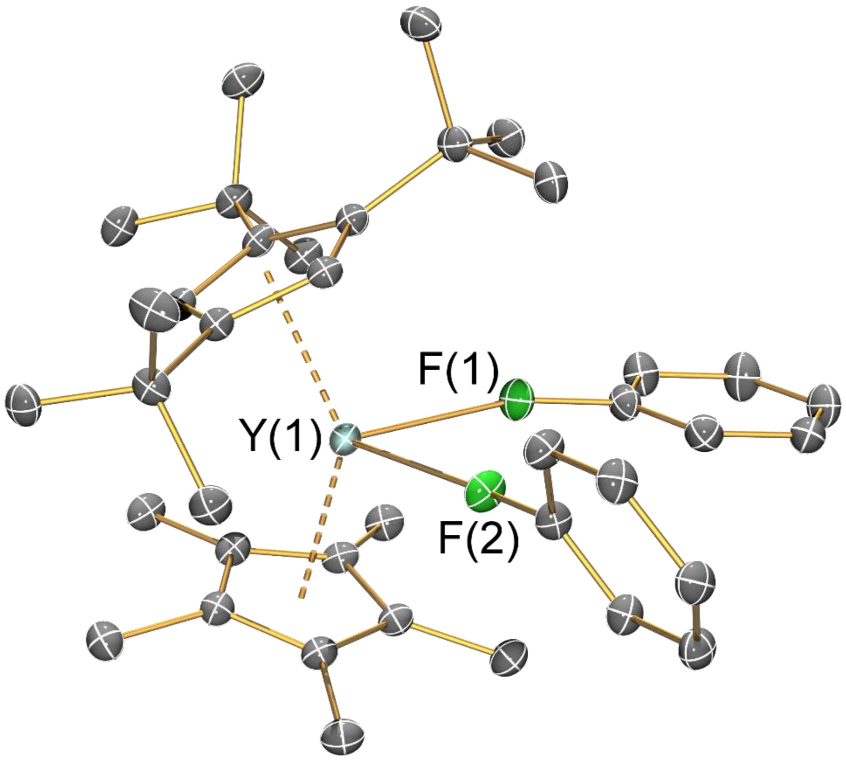

Figure S37. SCXRD structure of **6-Y** with selected atom labelling (Y: cyan, C: gray, F: green). Displacement ellipsoids set at 30% probability levels; hydrogen atoms and the $[Al\{OC(CF_3)_3\}_4]^-$ anion have been omitted for clarity. Selected bond lengths and angles: Y(1)···$Cp^{ttt}_{centroid}$ 2.353(2) Å, Y(1)···$Cp^*_{centroid}$ 2.347(2) Å, Y(1)–F(1) 2.390(2) Å, Y(1)–F(2) 2.380(2) Å, $Cp^{ttt}_{centroid}$···Y(1)···$Cp^*_{centroid}$ 142.21(6)°, F(1)–Y(1)–F(2) 73.02(7)°, Y(1)–F(1)–C_{ipso} 164.08(10), Y(1)–F(2)–C_{ipso} 166.89(10), $Cp^{ttt}_{centroid}$···Y(1)–F(1) 108.49(7)°, $Cp^{ttt}_{centroid}$···Y(1)–F(2) 105.08(7)°, $Cp^*_{centroid}$···Y(1)–F(1) 97.96(4)°, $Cp^*_{centroid}$···Y(1)–F(2) 105.72(7)°.

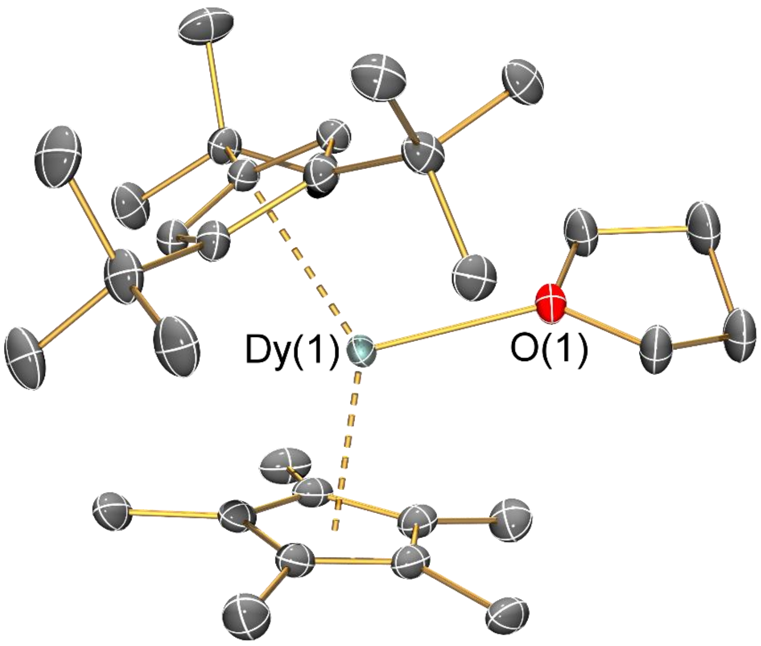

Figure S38. SCXRD structure of **7-Dy** with selected atom labelling (Dy: cyan, C: gray, O: red). Displacement ellipsoids set at 30% probability levels; hydrogen atoms and the $[Al\{OC(CF_3)_3\}_4]^-$ anion have been omitted for clarity. Selected bond lengths and angles: Dy(1)···$Cp^{ttt}_{centroid}$ 2.323(5) Å, Dy(1)···$Cp^{*}_{centroid}$ 2.324(5) Å, Dy(1)–O(1) 2.394(7) Å, $Cp^{ttt}_{centroid}$···Dy(1)···$Cp^{*}_{centroid}$ 140.2(2)°, $Cp^{ttt}_{centroid}$···Dy(1)–O(1) 111.6(2)°, $Cp^{*}_{centroid}$···Dy(1)–O(1) 107.1(2)°.

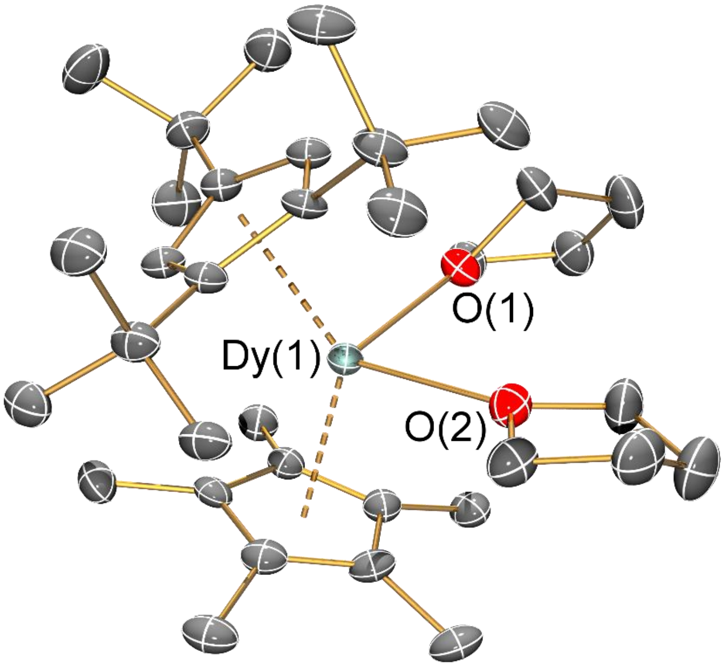

Figure S39. SCXRD structure of **8-Dy** with selected atom labelling (Dy: cyan, C: gray, O: red). Displacement ellipsoids set at 30% probability levels; hydrogen atoms and the $[Al\{OC(CF_3)_3\}_4]^-$ anion have been omitted for clarity. Selected bond lengths and angles: Dy(1)···$Cp^{ttt}_{centroid}$ 2.423(4) Å, Dy(1)···$Cp^*_{centroid}$ 2.399(4) Å, Dy(1)–O(1) 2.400(6) Å, Dy(1)–O(2) 2.386(7) Å, $Cp^{ttt}_{centroid}$···Dy(1)···$Cp^*_{centroid}$ 135.46(14)°, $Cp^{ttt}_{centroid}$···Dy(1)–O(1) 104.7(2)°, $Cp^{ttt}_{centroid}$···Dy(1)–O(2) 111.8(2)°, $Cp^*_{centroid}$···Dy(1)–O(1) 105.6(2)°, $Cp^*_{centroid}$···Dy(1)–O(2) 103.3(2)°, O(1)–Dy(1)–O(2) 83.9(2)°.

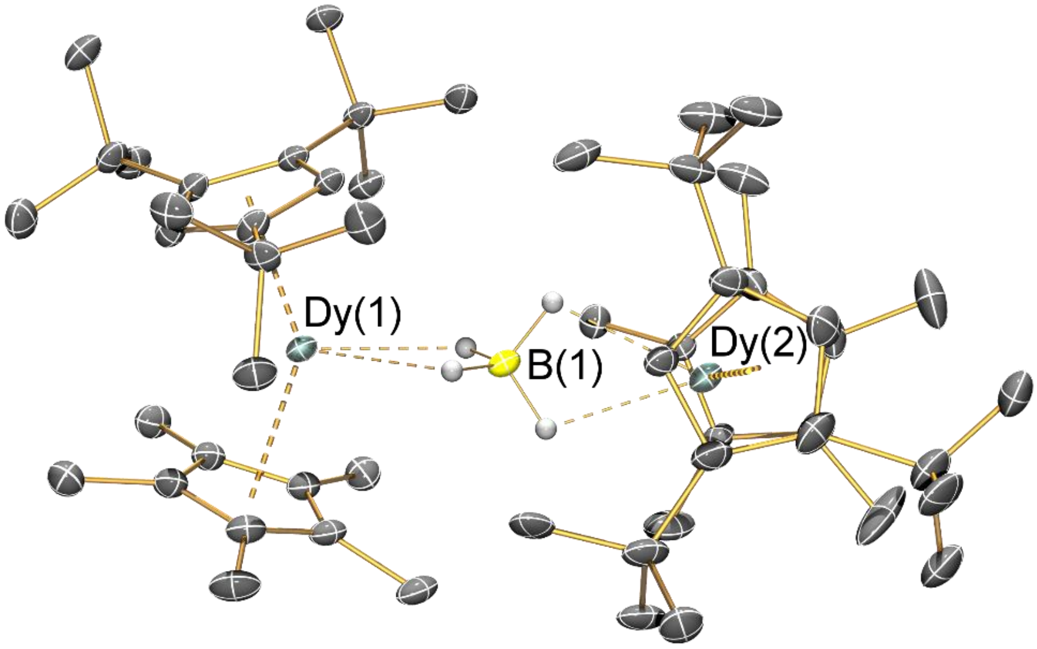

Figure S40. SCXRD structure of **9-Dy** with selected atom labelling (Dy: cyan, C: gray, B: yellow, H: white). Displacement ellipsoids set at 30% probability levels; hydrogen atoms, with the exception of those belonging to the BH_4^- group, the lattice solvent and the $[Al\{OC(CF_3)_3\}_4]^-$ anion have been omitted for clarity. Selected mean bond lengths and angles: Dy···$Cp^{ttt}_{centroid}$ 2.335(8) Å, Dy···$Cp^{*}_{centroid}$ 2.332(3) Å, Dy–B(1) 2.803(8) Å, $Cp^{ttt}_{centroid}$···Dy···$Cp^{*}_{centroid}$ 143.1(5)°, $Cp^{ttt}_{centroid}$···Dy–B(1) 109.0(3)°, $Cp^{*}_{centroid}$···Dy–B(1) 106.8(5)°.

Table S2. Crystallographic data for **2-Ln**.

	**2-Y**	**2-Dy**
Formula	$C_{104}H_{103}Al_2F_{75}O_8Y_2$	$C_{104}H_{103}Al_2Dy_2F_{75}O_8$
molecular mass, g mol^{-1}	3137.64	3284.82
cryst size, mm	0.095 × 0.074 × 0.055	0.339 × 0.168 × 0.146
cryst syst	monoclinic	monoclinic
space group	$P2_1/c$	$P2_1/c$
collection temperature, K	100(2)	100(2)
a, Å	9.52840(10)	9.52972(10)
b, Å	26.8687(3)	26.8408(3)
c, Å	24.1008(2)	24.1446(2)
α, °	90	90
β, °	94.5680(10)	94.5931(9)
γ, °	90	90
V, Å^3	6150.58(11)	6156.00(11)
Z	2	2
ρ_{calcd}, g cm^{-3}	1.694	1.772
μ, mm^{-1}	2.934	8.091
no. of reflections made	37922	69188
no. of unique reflns, R_{int}	12465, 0.0423	12658, 0.0414
no. of reflns with $F^2 > 2\sigma(F^2)$	10542	11316
transmn coeff range	0.51091–1.00000	0.753–1.000
R, R_w[a] ($F^2 > 2\sigma(F^2)$)	0.0511, 0.1353	0.0426, 0.1120
R, R_w[a] (all data)	0.0600, 0.1416	0.0474, 0.1150
S[a]	1.036	1.038
parameters, restraints	894, 977	894, 1187
max., min. diff map, e Å^{-3}	1.072, –0.936	1.059, –1.489

[a] Conventional $R = \Sigma||F_o| - |F_c||/\Sigma|F_o|$; $R_w = [\Sigma w(F_o^2 - F_c^2)^2/\Sigma w(F_o^2)^2]^{1/2}$; $S = [\Sigma w(F_o^2 - F_c^2)^2/\text{no. data} - \text{no. params})]^{1/2}$ for all data.

Table S3. Crystallographic data for **3-Ln**.

	**3-Y**	**3-Dy**
Formula	$C_{104}H_{103}Al_2Cl_3F_{72}O_8Y_2$	$C_{104}H_{103}Al_2Cl_3Dy_2F_{72}O_8$
molecular mass, g mol^{-1}	3186.99	3334.17
cryst size, mm	0.254 × 0.168 × 0.098	0.402 × 0.280 × 0.209
cryst syst	monoclinic	monoclinic
space group	$P2_1/c$	$P2_1/c$
collection temperature, K	100(2)	100(2)
a, Å	9.67510(10)	9.67800(10)
b, Å	26.4156(3)	26.4270(2)
c, Å	24.3682(3)	24.39180(10)
α, °	90	90
β, °	91.5310(10)	91.6530(10)
γ, °	90	90
V, Å^3	6225.64(12)	6235.86(8)
Z	2	2
ρ_{calcd}, g cm^{-3}	1.700	1.776
μ, mm^{-1}	3.452	8.540
no. of reflections made	76785	36686
no. of unique reflns, R_{int}	12749, 0.0415	12569, 0.0256
no. of reflns with $F^2 > 2\sigma(F^2)$	11556	11890
transmn coeff range	0.700–1.000	0.525–1.000
R, R_w[a] ($F^2 > 2\sigma(F^2)$)	0.0391, 0.1057	0.0306, 0.0809
R, R_w[a] (all data)	0.0425, 0.1098	0.0323, 0.0818
S[a]	1.048	0.980
parameters, restraints	1051, 1911	1033, 2219
max., min. diff map, e Å^{-3}	0.543, –1.002	0.991, –1.203

[a] Conventional $R = \Sigma||F_o| - |F_c||/\Sigma|F_o|$; $R_w = [\Sigma w(F_o^2 - F_c^2)^2/\Sigma w(F_o^2)^2]^{1/2}$; $S = [\Sigma w(F_o^2 - F_c^2)^2/\text{no. data} - \text{no. params})]^{1/2}$ for all data.

Table S4. Crystallographic data for **4-Ln**.

	**4-Y**	**4-Dy**
Formula	$C_{104}H_{103}Al_2Br_3F_{72}O_8Y_2$	$C_{49}H_{49}AlBrDyF_{36}O_4$
molecular mass, g mol^{-1}	3320.37	1655.27
cryst size, mm	0.399 × 0.125 × 0.074	0.392 × 0.291 × 0.202
cryst syst	monoclinic	monoclinic
space group	$P2_1/c$	$P2_1/n$
collection temperature, K	100(2)	200(2)
a, Å	9.70670(10)	11.42580(10)
b, Å	26.4870(3)	15.2258(2)
c, Å	24.4366(3)	35.8030(3)
α, °	90	90
β, °	90.6400(10)	91.0190(10)
γ, °	90	90
V, Å^3	6282.29(12)	6227.55(11)
Z	2	4
ρ_{calcd}, g cm^{-3}	1.755	1.765
μ, mm^{-1}	3.952	8.690
no. of reflections made	80414	37708
no. of unique reflns, R_{int}	12729, 0.0546	12540, 0.0302
no. of reflns with $F^2 > 2\sigma(F^2)$	11395	11492
transmn coeff range	0.675–1.000	0.526–1.000
R, R_w[a] ($F^2 > 2\sigma(F^2)$)	0.0434, 0.1152	0.0485, 0.1403
R, R_w[a] (all data)	0.0477, 0.1180	0.0517, 0.1432
S[a]	0.992	1.175
parameters, restraints	1051, 1918	1479, 4918
max., min. diff map, e Å^{-3}	1.194, –1.089	0.746, –1.145

[a] Conventional $R = \Sigma||F_o| - |F_c||/\Sigma|F_o|$; $R_w = [\Sigma w(F_o^2 - F_c^2)^2/\Sigma w(F_o^2)^2]^{1/2}$; $S = [\Sigma w(F_o^2 - F_c^2)^2$/no. data – no. params$)]^{1/2}$ for all data.

Table S5. Crystallographic data for **5-Ln**.

	**5-Y**	**5-Dy**
Formula	$C_{49}H_{48}AlF_{38}O_4Y$	$C_{49}H_{48}AlDyF_{38}O_4$
molecular mass, g mol^{-1}	1538.76	1612.35
cryst size, mm	0.250 × 0.225 × 0.189	0.189 × 0.104 × 0.061
cryst syst	Monoclinic	monoclinic
space group	*Pc*	*Pc*
collection temperature, K	293(2)	100(2)
a, Å	13.4660(4)	13.4963(2)
b, Å	10.2689(3)	10.2755(2)
c, Å	21.3655(6)	21.3597(4)
α, °	90	90
β, °	95.091(3)	94.878(2)
γ, °	90	90
V, Å^3	2942.79(15)	2951.46(9)
Z	2	2
ρ_{calcd}, g cm^{-3}	1.737	1.814
μ, mm^{-1}	1.091	8.436
no. of reflections made	48903	37174
no. of unique reflns, R_{int}	25234, 0.1333	10876, 0.0562
no. of reflns with $F^2 > 2\sigma(F^2)$	10021	10100
transmn coeff range	0.31702–1.00000	0.868–1.000
R, R_w[a] ($F^2 > 2\sigma(F^2)$)	0.0960, 0.2549	0.0426, 0.1160
R, R_w[a] (all data)	0.1947, 0.3144	0.0457, 0.1178
S[a]	0.888	0.993
parameters, restraints	1066, 2452	1434, 4800
max., min. diff map, e Å^{-3}	1.583, –1.692	0.729, –0.752

[a] Conventional $R = \Sigma||F_o| - |F_c||/\Sigma|F_o|$; $R_w = [\Sigma w(F_o^2 - F_c^2)^2/\Sigma w(F_o^2)^2]^{1/2}$; $S = [\Sigma w(F_o^2 - F_c^2)^2/\text{no. data} - \text{no. params})]^{1/2}$ for all data.

Table S6. Crystallographic data for **6-Y** and **7-Dy**.

	**6-Y**	**7-Dy**
Formula	$C_{55}H_{54}AlF_{38}O_4Y$	$C_{47}H_{52}AlDyF_{36}O_5$
molecular mass, g mol^{-1}	1616.87	1570.36
cryst size, mm	0.158 × 0.081 × 0.050	0.256 × 0.152 × 0.145
cryst syst	monoclinic	monoclinic
space group	$P2_1/c$	$P2_1/c$
collection temperature, K	100(2)	150(2)
a, Å	12.7619(5)	10.4411(3)
b, Å	16.6264(6)	33.5035(9)
c, Å	30.3895(7)	16.8103(4)
α, °	90	90
β, °	92.416(3)	91.470(3)
γ, °	90	90
V, Å^3	6442.4(4)	5878.6(3)
Z	4	4
ρ_{calcd}, g cm^{-3}	1.667	1.774
μ, mm^{-1}	2.834	8.412
no. of reflections made	34931	11885
no. of unique reflns, R_{int}	11670, 0.0587	11885
no. of reflns with $F^2 > 2\sigma(F^2)$	8856	9952
transmn coeff range	0.855–1.000	0.553–1.000
R, R_w[a] ($F^2 > 2\sigma(F^2)$)	0.0506, 0.1275	0.0897, 0.2561
R, R_w[a] (all data)	0.0713, 0.1406	0.0998, 0.2618
S[a]	0.963	1.081
parameters, restraints	1411, 4393	1331, 4268
max., min. diff map, e Å^{-3}	0.845, –0.817	2.118, –1.073

[a] Conventional $R = \Sigma||F_o| - |F_c||/\Sigma|F_o|$; $R_w = [\Sigma w(F_o^2 - F_c^2)^2/\Sigma w(F_o^2)^2]^{1/2}$; $S = [\Sigma w(F_o^2 - F_c^2)^2/\text{no. data} - \text{no. params})]^{1/2}$ for all data.

Table S7. Crystallographic data for **8-Dy** and **9-Dy**.

	**8-Dy**	**9-Dy**
Formula	$C_{51}H_{60}AlDyF_{36}O_6$	$C_{76}H_{96}AlBDy_2F_{38}O_4$
molecular mass, g mol^{-1}	1642.47	2158.31
cryst size, mm	0.286 × 0.054 × 0.028	0.222 × 0.118 × 0.044
cryst syst	monoclinic	triclinic
space group	$P2_1/c$	$P\bar{1}$
collection temperature, K	100(2)	100(2)
a, Å	18.2139(8)	16.1953(4)
b, Å	16.9517(8)	17.6714(5)
c, Å	20.4215(7)	19.4227(5)
α, °	90	106.544(2)
β, °	90.356(4)	110.819(2)
γ, °	90	103.507(2)
V, Å^3	6305.1(5)	4619.9(2)
Z	4	2
ρ_{calcd}, g cm^{-3}	1.730	1.552
μ, mm^{-1}	7.885	9.727
no. of reflections made	34068	64119
no. of unique reflns, R_{int}	11228, 0.1012	18403, 0.0583
no. of reflns with $F^2 > 2\sigma(F^2)$	7589	13699
transmn coeff range	0.894–1.000	0.805–1.000
R, R_w[a] ($F^2 > 2\sigma(F^2)$)	0.0840, 0.2256	0.0521, 0.1406
R, R_w[a] (all data)	0.1166, 0.2546	0.0709, 0.1528
S[a]	1.021	0.983
parameters, restraints	1249, 3582	1865, 5630
max., min. diff map, e Å^{-3}	1.306, –2.653	1.288, –1.325

[a] Conventional $R = \Sigma||F_o| - |F_c||/\Sigma|F_o|$; $R_w = [\Sigma w(F_o^2 - F_c^2)^2/\Sigma w(F_o^2)^2]^{1/2}$; $S = [\Sigma w(F_o^2 - F_c^2)^2/\text{no. data} - \text{no. params})]^{1/2}$ for all data.

Table S8. *AtomAccess* results on {Dy(Cp^{ttt})(Cp*)} SCXRD fragments.[1]

Parent complex	Equatorial binding mode	Largest cluster (% solid angle)
SIP [1]	n/a	12.2
CIP [1]	Al[OC(CF_3)$_3$]$_4$-κ^1-*F*	13.2
**2-Dy**	PhF-κ^1-*F*	14.1
**3-Dy**	PhCl-κ^1-*Cl*	15.5
**4-Dy**	PhBr-κ^1-*Br*	18.7
**5-Dy**	$C_6H_4F_2$-κ^2-*F*,*F*	21.1
**6-Y**	(PhF-κ^1-*F*)$_2$	21.8
**7-Dy**	THF-κ^1-*O*	20.5
**8-Dy**	(THF-κ^1-*O*)$_2$	29.3
**9-Dy**	$\mu_2,\eta^2:\eta^2$-BH_4	19.2–23.3
[Dy(Cp^{ttt})(Cp*)(BH_4)][1]	η^2-BH_4	20.0
[{Dy(Cp^{ttt})(Cp*)}$_2$(μ-F)] [Al{OC(CF_3)$_3$}$_4$][1]	μ_2-F	20.2–23.1

6. Infrared Spectroscopy

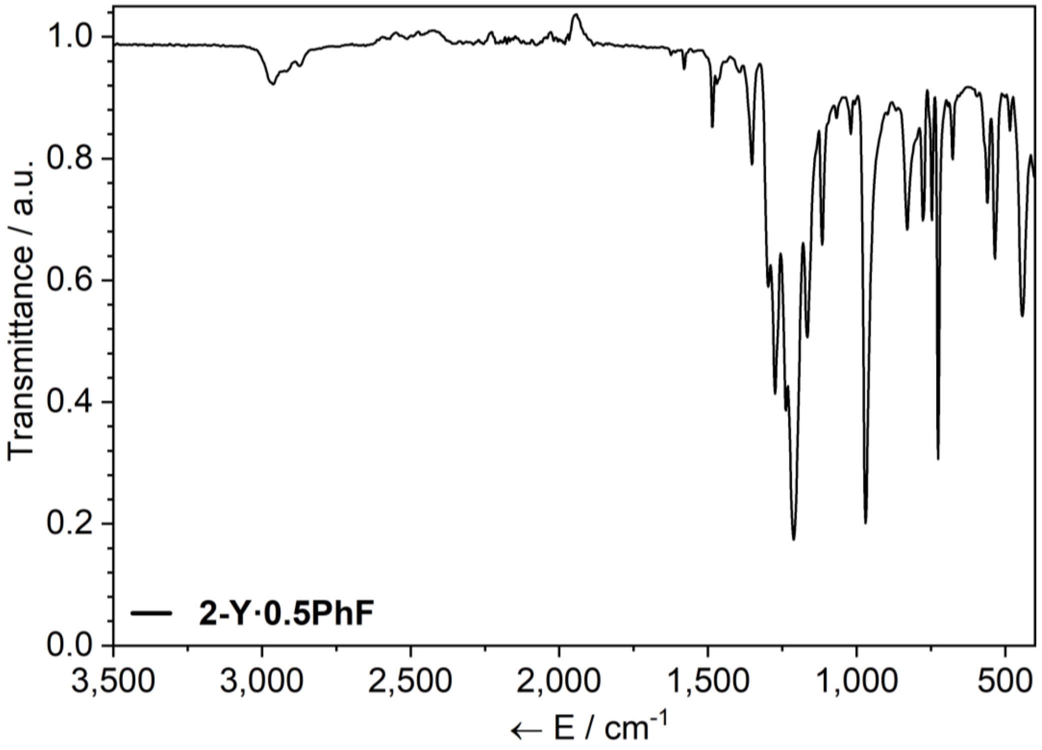

Figure S41. ATR-IR spectrum of **2-Y**, recorded as a microcrystalline powder.

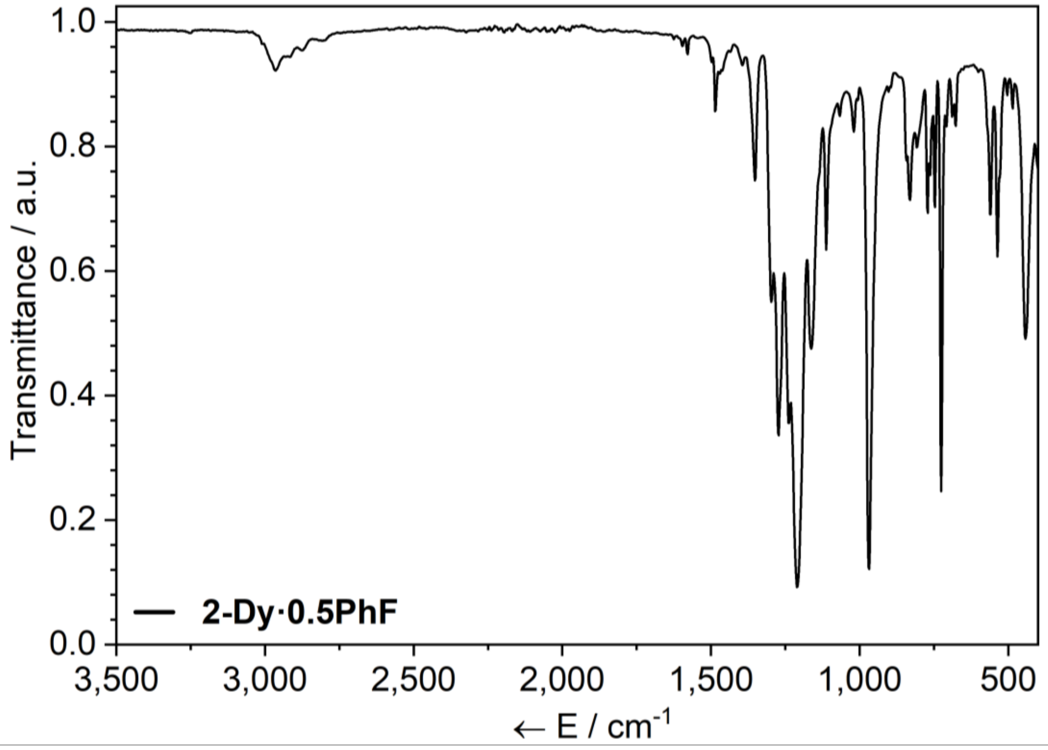

Figure S42. ATR-IR spectrum of **2-Dy**, recorded as a microcrystalline powder.

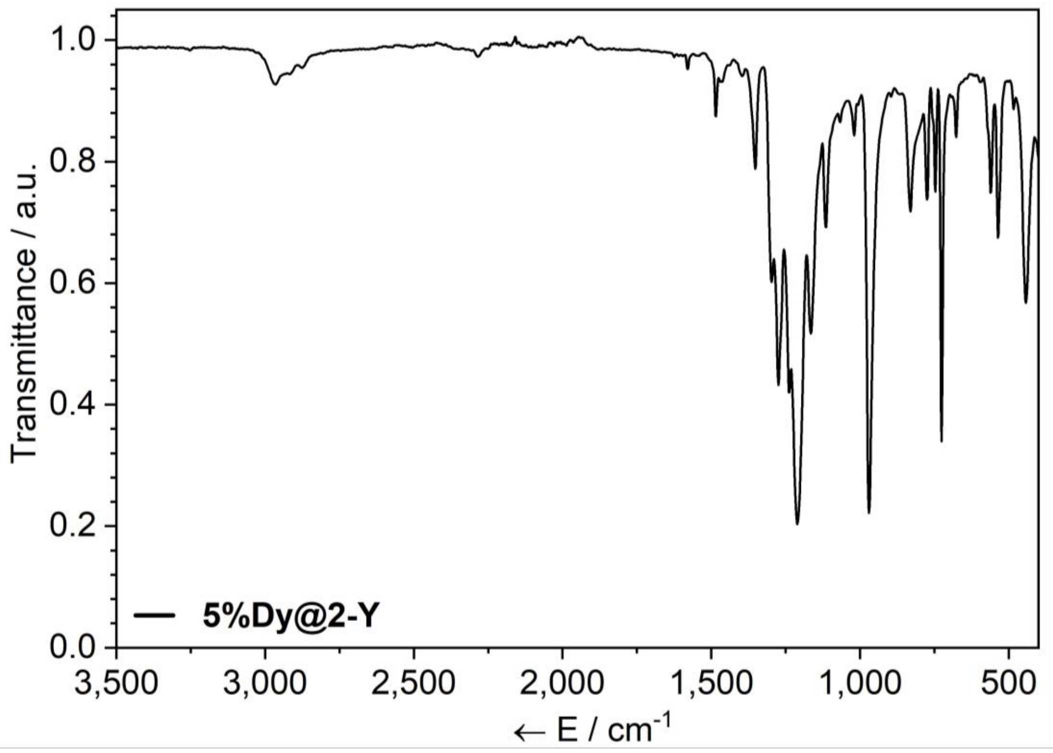

Figure S43. ATR-IR spectrum of **5%Dy@2-Y**, recorded as a microcrystalline powder.

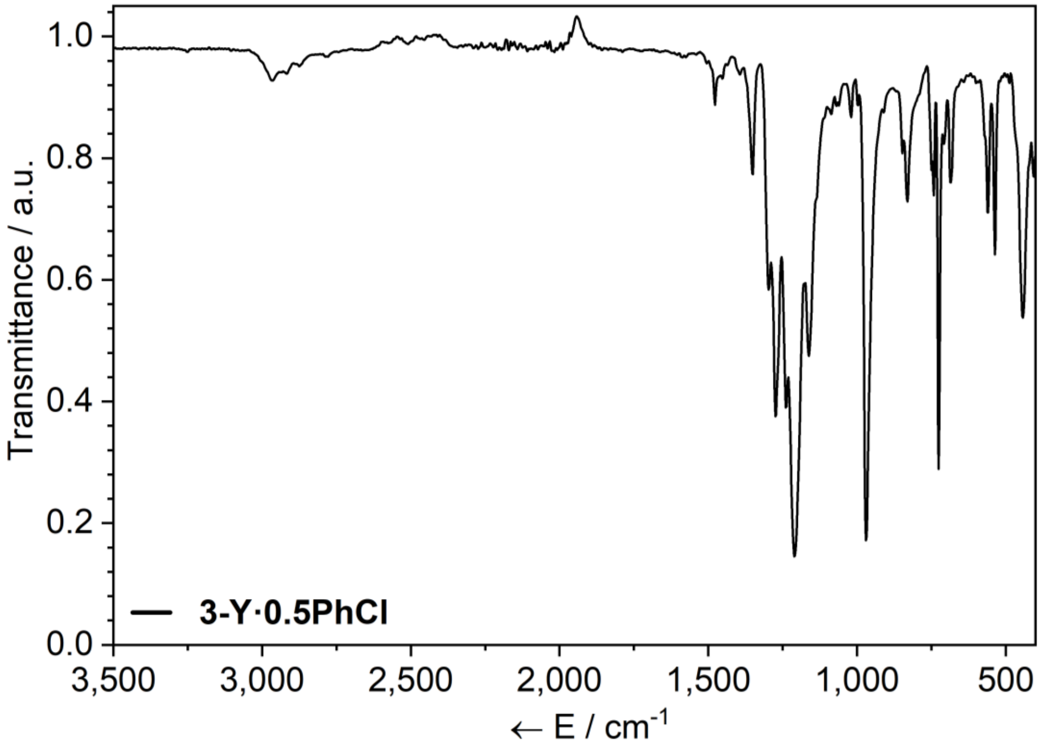

Figure S44. ATR-IR spectrum of **3-Y**, recorded as a microcrystalline powder.

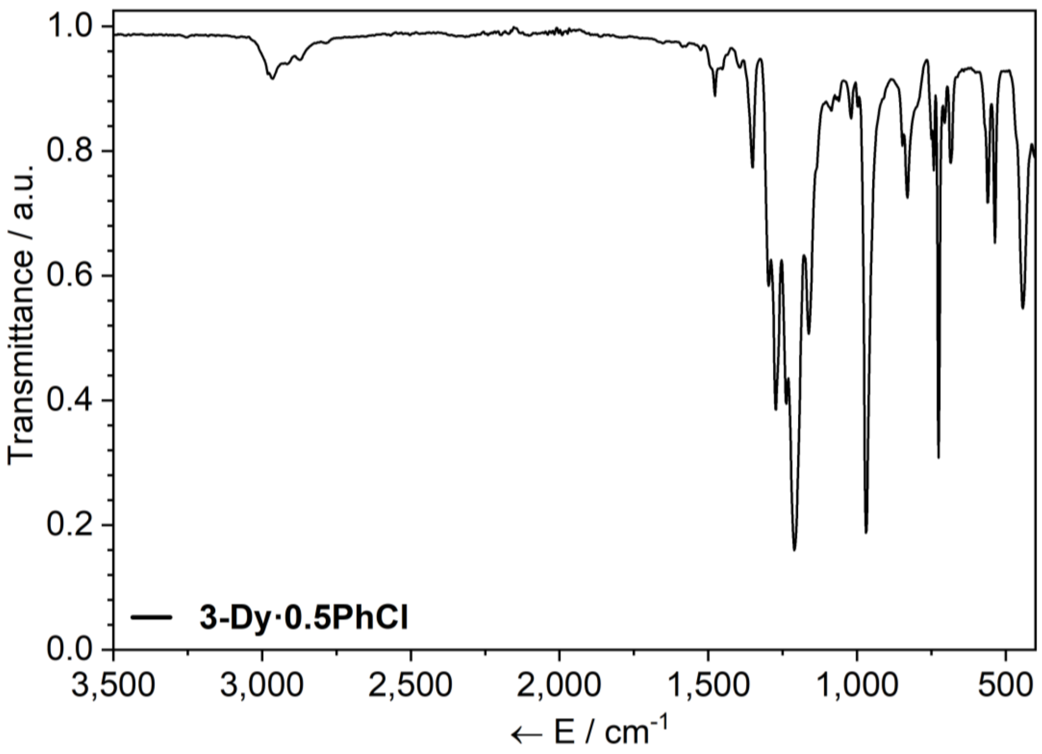

Figure S45. ATR-IR spectrum of **3-Dy**, recorded as a microcrystalline powder.

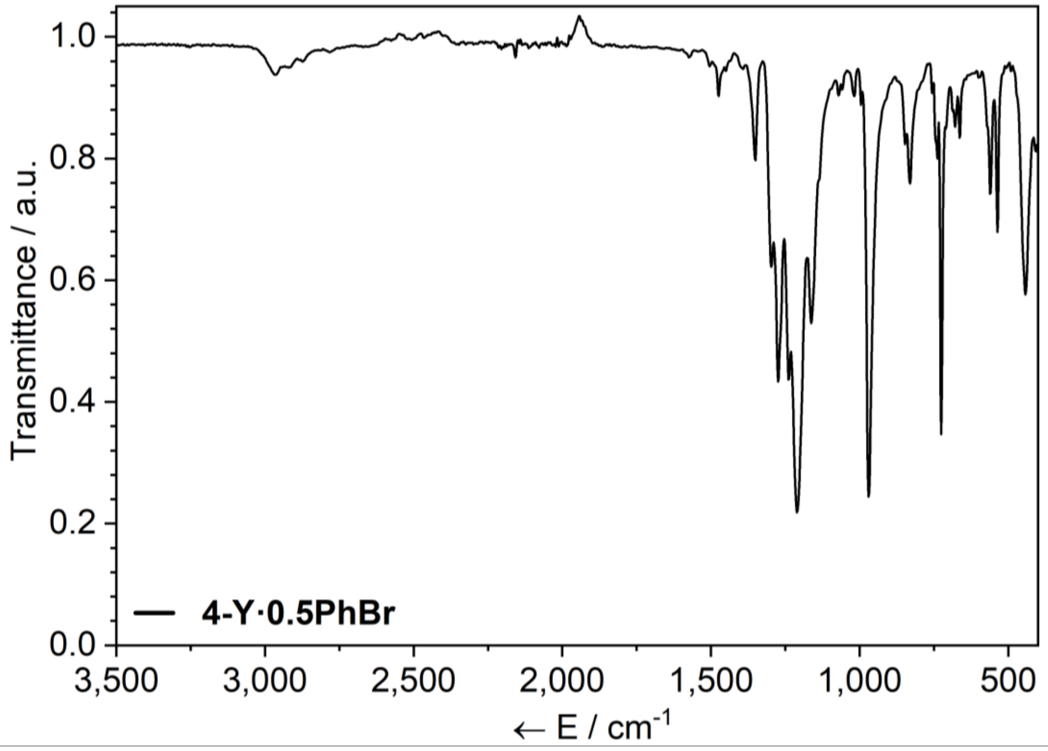

Figure S46. ATR-IR spectrum of **4-Y**, recorded as a microcrystalline powder.

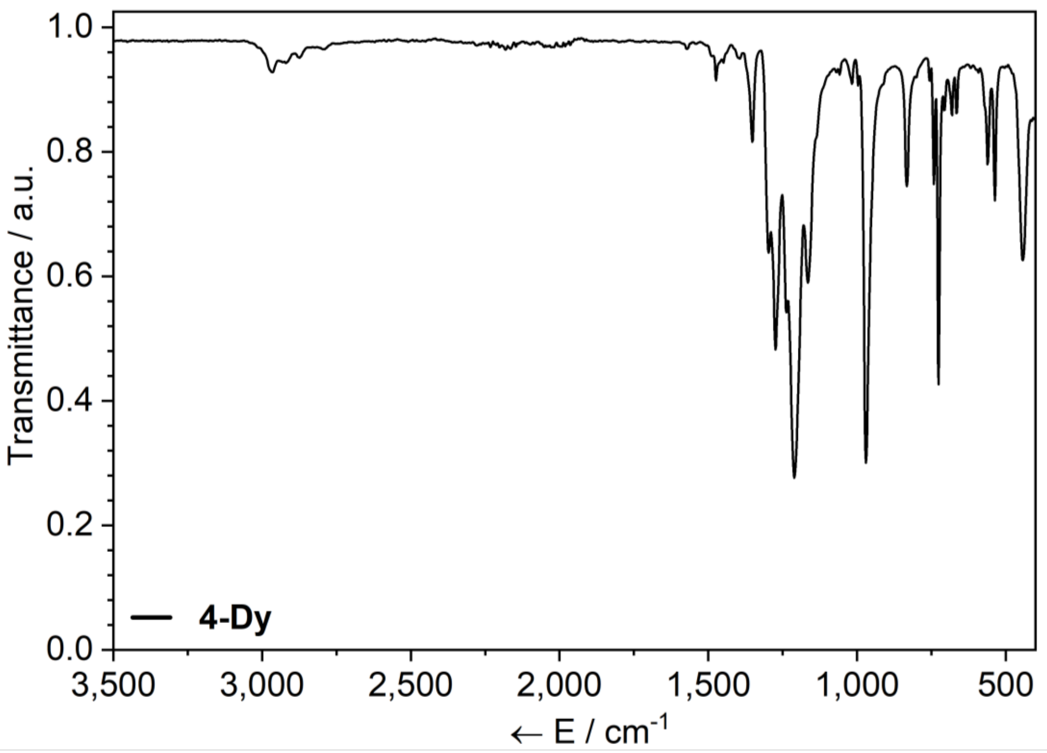

Figure S47. ATR-IR spectrum of **4-Dy**, recorded as a microcrystalline powder.

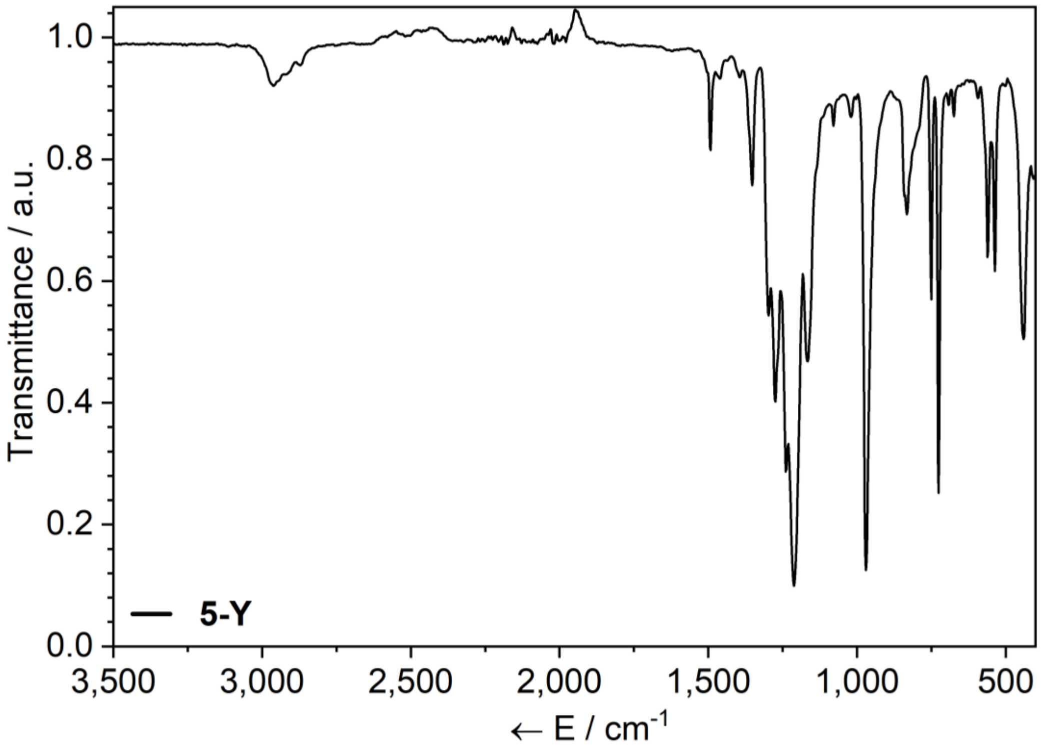

Figure S48. ATR-IR spectrum of **5-Y**, recorded as a microcrystalline powder.

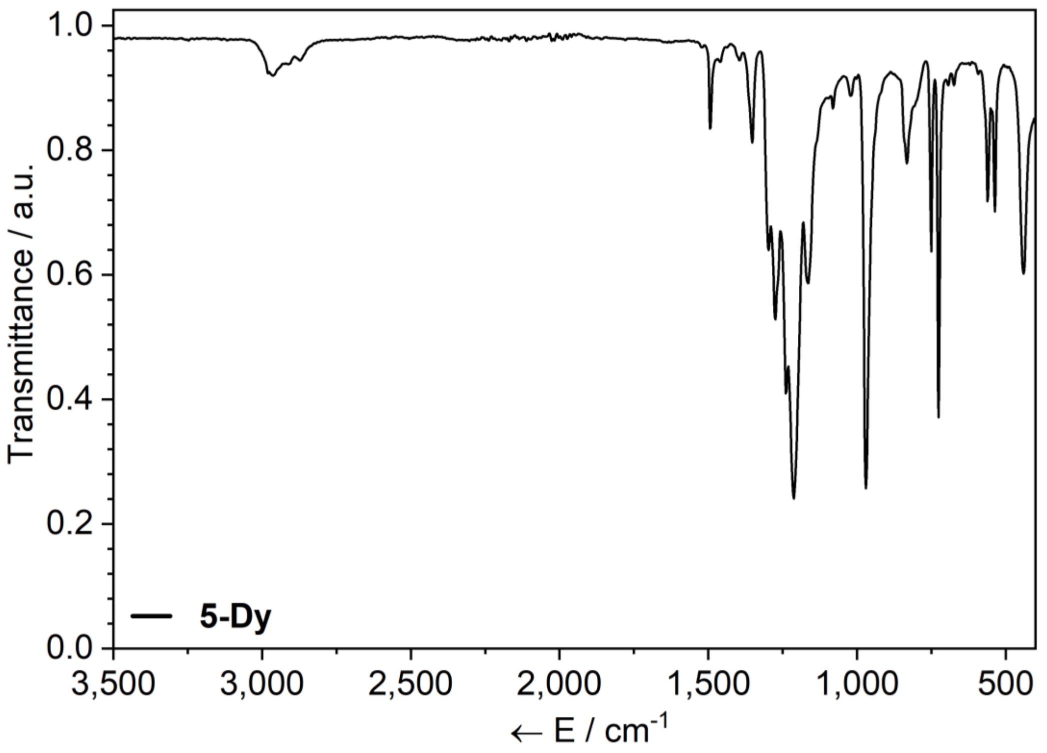

Figure S49. ATR-IR spectrum of **5-Dy**, recorded as a microcrystalline powder.

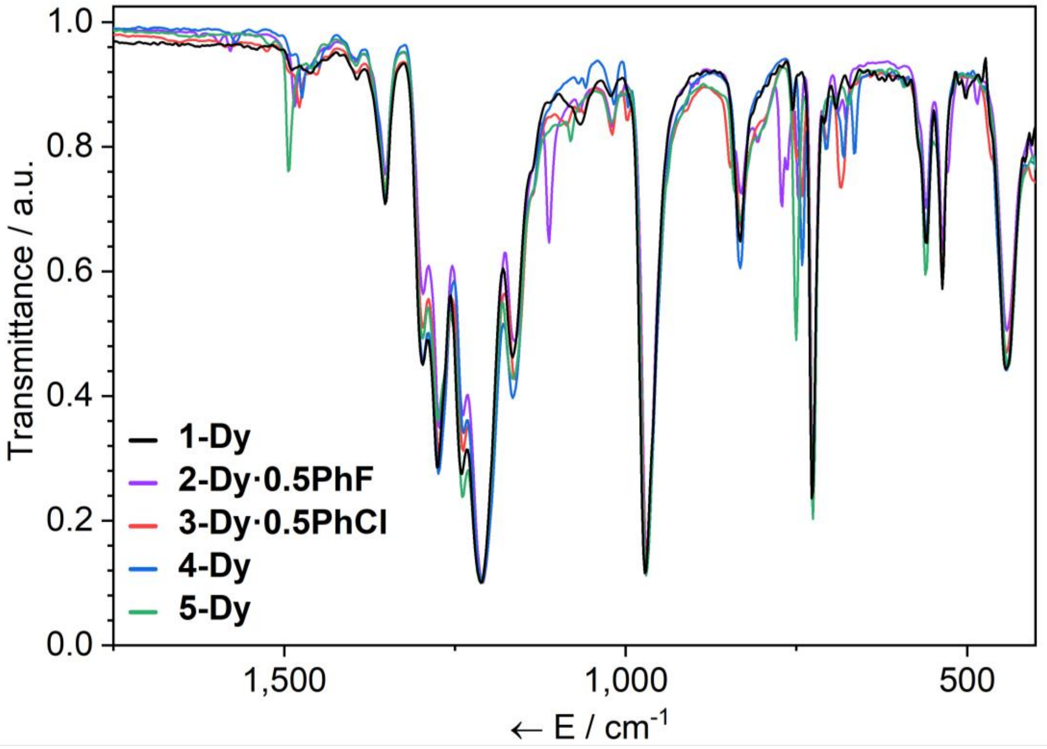

Figure S50. Overlay of the ATR-IR spectra recorded for **1-5-Dy**. The data are normalized.

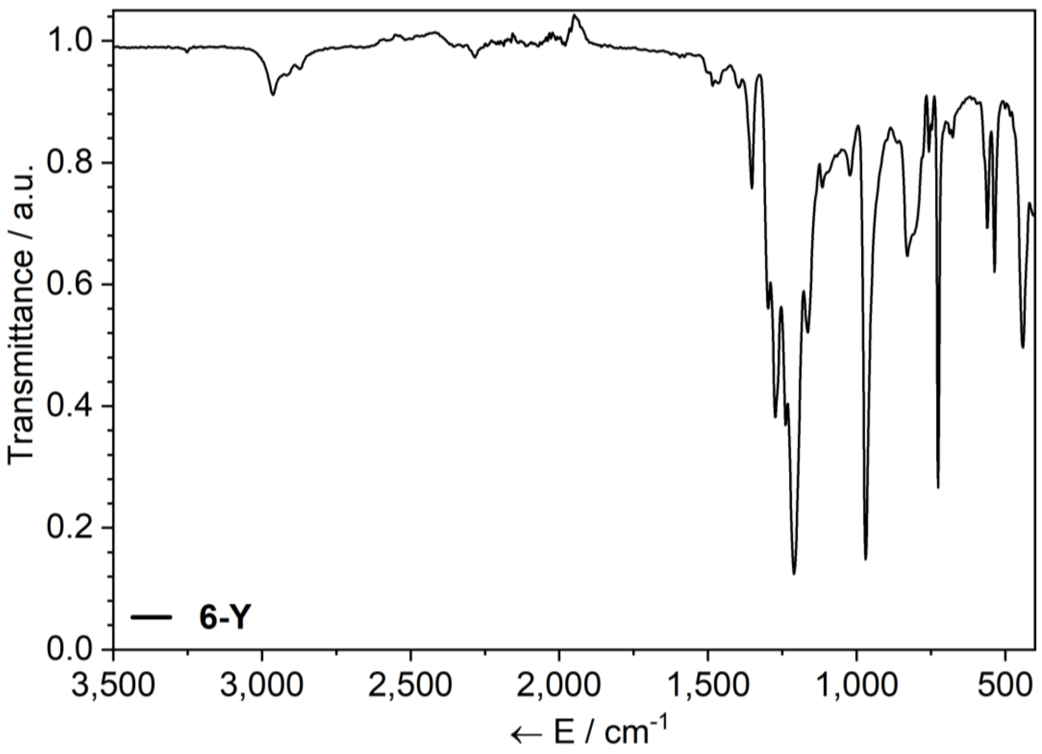

Figure S51. ATR-IR spectrum of **6-Y**, recorded as a microcrystalline powder.

7. DFT calculations

All calculations were performed using the Orca 4.2.1 or 5.0.3 software package.[14–16] The PBE0 hybrid functional, augmented by Stefan Grimme's D4 dispersion correction,[17,18] the Def2-TZVP basis set,[19–21] and the RIJCOSX approximation were employed throughout. The initial coordinates were obtained through unoptimized XRD data and manually altered to make the Y analogues of the Dy cations. Atom coordinates were verified as minima on the potential energy surface by the absence of imaginary vibrational modes.

Table S9. Calculated atomic coordinates for $[Al\{OC(CF_3)_3\}_4]^-$.

Atom	x	y	z	Atom	x	y	Z
Al	4.532058	9.405431	3.764383	F	6.066174	6.248042	6.525827
F	7.970596	12.568006	4.881661	F	8.464619	8.021809	3.792304
F	1.085118	8.211057	6.814503	F	0.923441	10.997481	7.103628
O	3.651449	9.998709	5.131879	F	2.847619	11.970111	7.084584
F	8.088318	12.767564	2.051493	F	4.643532	10.009945	7.565546
F	7.348838	10.862466	1.380734	F	4.391924	5.689994	4.52103
F	6.152924	12.618367	1.104752	F	3.831876	8.036921	7.335051
F	6.227639	4.641875	4.118137	F	4.718337	8.109374	0.276369
F	5.214991	12.987953	5.053285	F	1.12984	7.663508	2.283073
F	6.253564	14.15462	3.561375	F	0.899751	8.165129	0.204788
F	8.077818	6.117517	2.885823	F	2.425569	6.767646	0.825827
F	8.051136	6.110993	5.695077	F	0.913062	10.306146	2.096629
F	1.702842	10.836054	0.164856	C	2.04956	11.151163	6.396992
F	5.268398	5.791976	2.561715	C	1.707085	8.706085	5.737681
F	8.369074	10.698803	3.909998	C	3.516145	9.330619	7.408289
O	5.511103	8.097896	4.316891	C	7.50505	7.265459	3.263111
F	7.060218	7.878504	2.174214	C	2.770917	9.792936	6.112518
F	0.772089	9.203506	4.92941	C	7.394838	11.45811	4.40684
F	2.774038	9.505766	8.508559	C	3.819845	9.076997	0.149789
F	6.850922	10.814803	5.431748	C	6.30069	11.755709	3.322434
F	4.39963	13.177527	3.078408	C	2.038691	10.414453	1.388947
F	2.77754	11.355827	1.95221	C	6.330032	7.049426	4.281353
O	3.484087	8.837529	2.501198	C	5.528886	13.045911	3.758001
F	1.738186	11.758276	5.259535	C	6.992145	12.008566	1.943082
F	7.233295	8.023316	6.253299	C	1.797106	7.895784	1.161255
F	2.293633	7.695856	5.107725	C	2.823474	9.058168	1.359235
F	3.206025	8.91012	–1.028189	C	5.54258	5.764812	3.866924
F	4.479082	10.233325	0.098359	C	6.935222	6.850162	5.714002
O	5.454945	10.736952	3.163852				

Table S10. Calculated atomic coordinates for [Y(Cp^{ttt})(Cp*)(PhF-κ-*F*)]$^+$ (**2′-Y**).

Atom	x	y	z	Atom	x	y	z
Y	–2.30152	12.86343	6.007704	H	–1.82475	15.35793	3.436664
F	–1.90884	10.44787	6.522516	C	1.225825	16.29711	4.808377
C	–4.68483	13.73581	6.546105	H	1.570506	16.52127	5.82005
C	–4.81715	12.34216	6.306628	H	1.33708	17.20292	4.206778
C	–4.52429	12.10227	4.939747	H	1.882131	15.53681	4.395669
C	–4.23287	13.35231	4.327017	C	0.700198	12.48559	4.567128
C	–4.34379	14.36212	5.315697	C	1.775267	13.13245	3.696767
C	–5.07835	14.4467	7.79974	H	2.121778	12.41179	2.952069
H	–4.94024	13.82937	8.688903	H	2.634804	13.42317	4.303014
H	–6.13857	14.719	7.766223	H	1.424662	14.00976	3.162027
H	–4.51813	15.37144	7.945047	C	–0.51845	12.12282	3.701752
C	–5.33256	11.33991	7.286561	H	–1.19692	11.42441	4.224713
H	–5.04378	10.32135	7.027062	H	–0.22874	11.57524	2.800733
H	–6.42661	11.36536	7.309612	H	–1.07723	13.00382	3.372056
H	–4.99524	11.5373	8.306559	C	1.29952	11.17431	5.082494
C	–4.66657	10.80537	4.212279	H	0.576632	10.56449	5.62746
H	–3.92081	10.68542	3.421982	H	2.151798	11.36123	5.738042
H	–5.64538	10.74897	3.724649	H	1.655004	10.58148	4.237047
H	–4.59133	9.94439	4.876673	C	–1.06452	13.4459	9.298725
C	–4.06295	13.5593	2.857074	C	0.186924	13.10044	10.1073
H	–3.63405	14.53318	2.622282	H	0.904984	13.92198	10.07544
H	–5.0346	13.5107	2.354528	H	0.679829	12.20736	9.717954
H	–3.43386	12.796	2.392786	H	–0.07204	12.91441	11.15257
C	–4.40596	15.83129	5.068393	C	–2.04482	12.27143	9.365044
H	–4.11405	16.40908	5.945623	H	–2.38008	12.09007	10.38958
H	–5.43561	16.11786	4.828966	H	–1.58703	11.35027	8.996641
H	–3.78524	16.14936	4.231196	H	–2.95261	12.47806	8.778088
C	–0.39115	14.66322	5.765622	C	–1.72614	14.68236	9.895185
C	0.199634	13.34939	5.732494	H	–1.05145	15.54048	9.873469
C	0.022014	12.78765	7.023816	H	–1.99719	14.49798	10.93687
H	0.3673	11.80952	7.323101	H	–2.63865	14.95243	9.358686
C	–0.67928	13.6842	7.855461	C	–2.34979	9.136689	6.46021
C	–0.94436	14.81712	7.069315	C	–2.95679	8.613771	7.575447
H	–1.43044	15.71115	7.425241	H	–3.07917	9.20928	8.470796
C	–0.24852	15.87067	4.835119	C	–3.40284	7.301255	7.498552
C	–1.01569	17.07205	5.394547	H	–3.88903	6.854939	8.3571
H	–2.07642	16.86438	5.523033	C	–3.22725	6.567109	6.334827
H	–0.92355	17.90873	4.699345	H	–3.58009	5.544829	6.283717
H	–0.60748	17.40106	6.351574	C	–2.59758	7.135895	5.2377
C	–0.76864	15.6373	3.417544	H	–2.45633	6.560808	4.330968
H	–0.22581	14.86617	2.875192	C	–2.14314	8.4463	5.291284
H	–0.68291	16.55691	2.834662	H	–1.64745	8.912423	4.449464

Table S11. Calculated atomic coordinates for [Y(Cp^{ttt})(Cp*)(PhCl-κ-*Cl*)]$^+$ (**3′-Y**).

Atom	x	y	z	Atom	x	y	z
Y	10.92509	12.65932	18.22324	H	8.449165	13.36486	13.05487
Cl	10.32321	9.975897	17.27458	H	7.679048	14.31274	14.3353
C	9.107417	14.53134	18.48651	C	10.44375	12.25732	14.56903
C	8.447736	13.25151	18.45935	H	11.4031	12.26766	15.10208
C	8.609137	12.73548	17.14471	H	10.67293	12.18686	13.50288
H	8.176645	11.81117	16.79121	H	9.901947	11.35094	14.84723
C	9.357158	13.63263	16.35332	C	13.32286	13.38899	17.57667
C	9.676181	14.71152	17.19359	C	13.39105	11.99022	17.80696
H	10.20371	15.59619	16.8758	C	13.14659	11.75915	19.18577
C	9.052665	15.6932	19.48154	C	12.94973	13.02055	19.81183
C	9.589494	15.34085	20.86785	C	13.07475	14.02808	18.82271
H	9.009233	14.5744	21.37744	C	13.69899	14.07399	16.30377
H	9.583744	16.22464	21.50996	H	13.42904	13.49032	15.42192
H	10.62462	14.99399	20.79972	H	14.78213	14.23062	16.26112
C	7.611007	16.21231	19.5648	H	13.23189	15.05521	16.21046
H	7.263723	16.51098	18.57345	C	13.80078	10.97483	16.79102
H	7.572069	17.0918	20.21282	H	13.6845	9.960471	17.16983
H	6.912007	15.47814	19.95264	H	14.85616	11.10478	16.53288
C	9.884593	16.87322	18.97267	H	13.23795	11.04993	15.85678
H	10.92817	16.60897	18.81255	C	13.2517	10.46173	19.91916
H	9.855147	17.6745	19.71348	H	12.43596	10.30889	20.63097
H	9.483065	17.27834	18.04217	H	14.1795	10.4303	20.49927
C	7.881176	12.37055	19.58192	H	13.26292	9.606394	19.2454
C	6.86595	13.05438	20.49453	C	12.84713	13.22278	21.28907
H	6.470008	12.32491	21.20526	H	12.53184	14.23346	21.54643
H	6.027204	13.43747	19.91079	H	13.82189	13.06335	21.76163
H	7.284084	13.87476	21.06982	H	12.1537	12.52427	21.76502
C	9.068192	11.86555	20.42104	C	13.22841	15.48962	19.07379
H	9.68961	11.1446	19.85942	H	12.92494	16.08936	18.21529
H	8.735687	11.29833	21.29465	H	14.28233	15.71852	19.26395
H	9.693036	12.68255	20.79968	H	12.66662	15.83188	19.94273
C	7.166937	11.14541	19.00413	C	11.01765	8.558548	18.03945
H	7.82973	10.50215	18.42464	C	11.9653	7.836256	17.34032
H	6.333923	11.44265	18.36476	H	12.27278	8.142125	16.3491
H	6.76014	10.5451	19.82075	C	12.50219	6.707839	17.94203
C	9.628854	13.51579	14.86861	H	13.24704	6.127124	17.41179
C	10.38857	14.73627	14.36145	C	12.08368	6.321156	19.20649
H	9.813376	15.65191	14.51333	H	12.50651	5.437759	19.66865
H	10.57869	14.63863	13.29066	C	11.11419	7.054135	19.87442
H	11.35308	14.85088	14.86032	H	10.77422	6.743488	20.8548
C	8.288042	13.42926	14.1339	C	10.56505	8.185341	19.29019
H	7.717416	12.55036	14.44146	H	9.796097	8.754834	19.79581

Table S12. Calculated atomic coordinates for [Y(Cp^{ttt})(Cp*)(PhBr-κ-*Br*)]$^+$ (**4′-Y**).

Atom	x	y	z	Atom	x	y	z
Y	1.744194	5.003137	13.12254	H	3.225713	3.686364	15.16594
Br	0.018709	6.708144	15.00358	C	1.1541	2.690013	12.00011
C	3.059527	6.938131	11.99303	C	–0.52338	3.73718	13.16755
C	3.683776	5.753208	11.47382	C	0.058858	2.948811	15.57196
C	4.281319	5.092999	12.58852	H	0.980276	2.841423	16.14753
H	4.862182	4.187203	12.52068	H	–0.54744	2.060996	15.77759
C	3.33559	6.969709	13.38904	H	–0.48635	3.80564	15.9711
H	3.022917	7.750319	14.06541	C	2.82933	5.214328	9.107564
C	2.104284	7.953063	11.35817	H	2.07521	4.533092	9.507817
C	4.099496	5.84588	13.7589	H	3.149594	4.818734	8.141137
C	1.456547	8.85244	16.22578	H	2.347438	6.169687	8.914121
H	1.268665	9.392369	15.3077	C	5.07558	6.281325	9.482278
C	1.007652	7.557949	16.40206	H	4.70351	7.297714	9.382574
C	4.735809	6.680403	16.02834	H	5.398623	5.941051	8.494821
H	5.527923	7.304162	15.61002	H	5.953188	6.309484	10.13164
H	5.03496	6.383356	17.0361	C	1.893359	7.46765	18.6044
H	3.835771	7.290427	16.11919	H	2.053093	6.931256	19.53188
C	4.027914	5.312556	10.04948	C	1.360758	2.442289	13.3848
C	4.695068	3.934071	10.06683	C	–1.84209	4.382859	13.44958
H	5.663792	3.960651	10.56847	H	–1.97176	4.612358	14.50737
H	4.872964	3.607272	9.040663	H	–2.65834	3.70879	13.17084
H	4.074008	3.182373	10.55206	H	–1.99797	5.304392	12.88156
C	2.63638	8.624217	10.09394	C	2.148084	9.450508	17.27003
H	3.582739	9.127968	10.30057	H	2.50864	10.46523	17.15333
H	1.923867	9.380651	9.75581	C	2.370253	1.490436	13.94246
H	2.793947	7.936803	9.268881	H	3.36457	1.629644	13.51155
C	1.200462	6.850507	17.57357	H	2.076672	0.456426	13.73518
H	0.814756	5.84569	17.68768	H	2.459696	1.579596	15.02575
C	1.796861	9.087557	12.33796	C	5.777363	4.601819	15.1361
H	1.292676	8.722255	13.23316	H	5.640264	3.677293	14.57084
H	1.130712	9.808717	11.8605	H	6.067407	4.325514	16.15254
H	2.705179	9.614646	12.63598	H	6.603492	5.155172	14.68585
C	0.778318	7.235898	11.06817	C	–0.69369	3.85455	10.59257
H	0.900937	6.375262	10.41076	H	–0.00891	3.902724	9.745388
H	0.04765	7.905622	10.60603	H	–1.21705	4.810788	10.65489
H	0.290939	6.909809	12.0055	H	–1.44709	3.097821	10.34959
C	0.314589	3.0758	14.10552	C	2.370228	8.76071	18.45204
C	4.507094	5.44674	15.16085	H	2.909334	9.236545	19.26184
C	0.000128	3.502615	11.86776	C	1.815954	1.929237	10.90099
C	3.366027	4.608026	15.74391	H	1.238095	1.021483	10.69485
H	2.424931	5.176558	15.77523	H	2.82148	1.606734	11.17061
H	3.558834	4.303115	16.77624	H	1.876483	2.484825	9.966039

Table S13. Calculated atomic coordinates for [Y(Cp^{ttt})(Cp*)($C_6H_4F_2$-κ^2-*F*,*F*)]$^+$ (**5′-Y**).

Atom	x	y	z	Atom	X	y	z
Y	9.582510	–4.488920	11.206720	C	9.327772	–5.949746	13.330701
C	10.108933	–4.320346	8.556010	C	8.381398	–4.904631	13.455837
F	7.246539	–3.531424	10.944901	C	9.316908	–6.463900	7.308973
F	7.849711	–6.091061	10.531485	H	10.262894	–6.998719	7.315866
C	9.087686	–0.460378	10.213114	H	8.785526	–6.775984	6.407089
H	8.729618	–0.443745	9.181420	H	8.730801	–6.803694	8.166215
H	9.054397	0.564080	10.590322	C	10.623315	–5.384578	13.439648
H	8.384585	–1.051813	10.806068	C	8.103166	–4.349896	7.002850
C	10.553472	–2.462249	9.890104	H	7.399149	–4.499867	7.820431
C	9.654817	–3.048591	8.985940	H	7.689110	–4.839194	6.118991
H	8.773816	–2.556888	8.607104	H	8.153109	–3.283292	6.780147
C	11.421511	–0.228432	9.358288	C	11.460663	–6.987522	9.895307
H	11.092498	–0.343061	8.323081	H	12.076940	–7.890784	9.904966
H	12.450929	–0.586736	9.426526	H	10.556499	–7.209782	9.331980
H	11.414175	0.836486	9.604947	H	11.184951	–6.803499	10.946078
C	13.434747	–5.555155	10.240555	C	11.335555	–4.566082	9.273469
H	13.128898	–5.336121	11.265291	C	11.002125	–0.827084	11.739071
H	14.059182	–4.734630	9.881549	H	10.940772	0.222900	12.035761
H	14.055573	–6.452928	10.269597	H	12.038728	–1.149564	11.854143
C	11.601987	–3.107296	14.095665	H	10.389299	–1.405832	12.428570
H	12.432482	–3.084432	13.387617	C	12.239471	–5.798914	9.319601
H	12.000969	–3.480471	15.043328	C	10.510594	–1.008654	10.312298
H	11.283972	–2.079948	14.260185	C	6.690456	–5.602910	9.986027
C	11.585302	–3.414085	10.072894	C	9.089169	–3.685551	13.649365
H	12.457935	–3.277081	10.693706	C	12.819839	–6.168531	7.952949
C	8.442765	–2.365084	13.941255	H	13.346949	–5.317469	7.516796
H	7.753908	–2.450884	14.785911	H	12.067980	–6.505927	7.245581
H	7.859907	–1.970894	13.102808	H	13.541217	–6.980881	8.071357
H	9.178384	–1.606368	14.203031	C	5.889034	–6.405211	9.212441
C	9.487568	–4.945511	7.295617	H	6.153396	–7.441471	9.045343
C	10.369299	–4.541750	6.104653	C	4.765379	–5.830687	8.632560
H	10.373051	–3.456177	5.988464	H	4.125087	–6.439527	8.007159
H	9.971042	–4.981156	5.185904	C	4.461658	–4.494032	8.840481
H	11.399920	–4.867738	6.215706	H	3.587931	–4.057878	8.373722
C	6.387045	–4.275247	10.198382	C	9.018807	–7.408202	13.267848
C	5.273866	–3.699221	9.638542	H	8.979825	–7.830063	14.277290
H	5.060456	–2.653165	9.820102	H	9.776374	–7.972089	12.720300
C	6.898505	–5.071893	13.535214	H	8.050927	–7.608812	12.804588

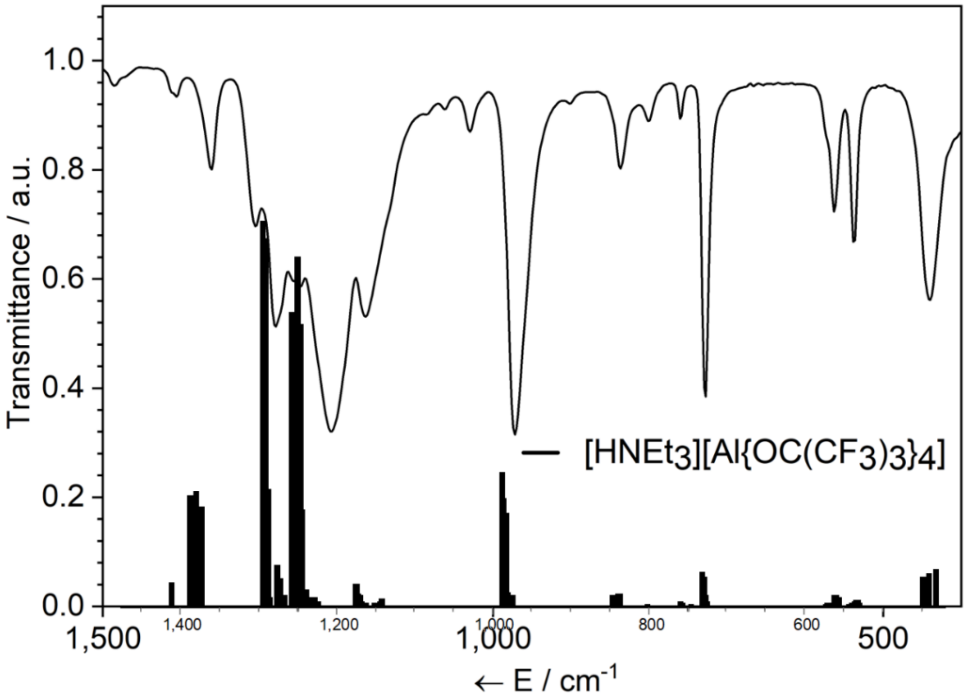

Figure S52. Overlay of the simulated IR spectrum of $[Al\{OC(CF_3)_3\}_4]^-$ (0.99 scale) and the measured ATR-IR spectrum of $[HNEt_3][Al\{OC(CF_3)_3\}_4]$.

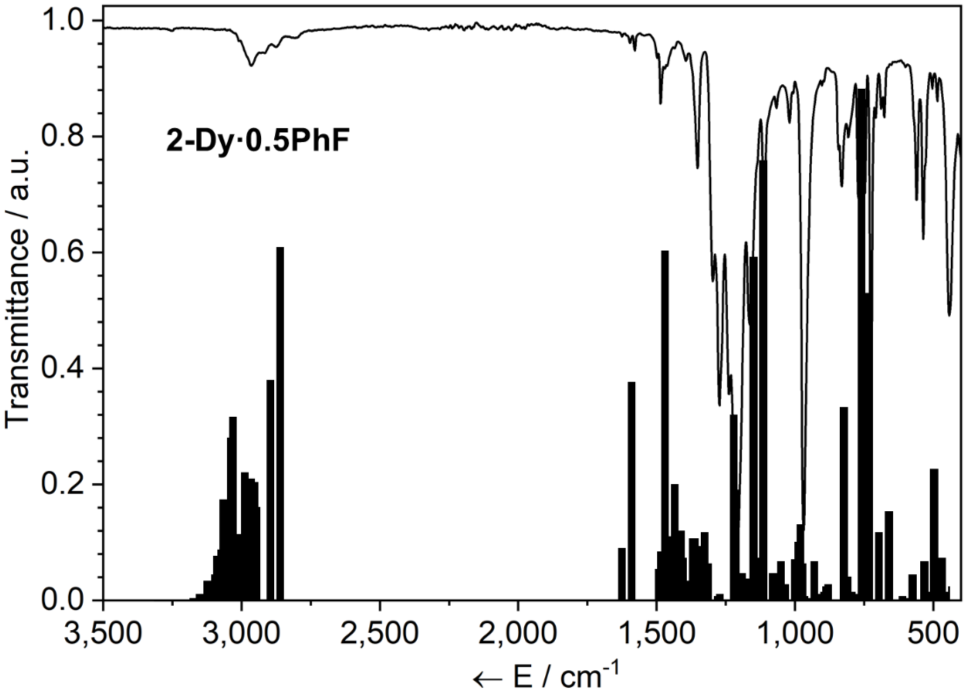

Figure S53. Overlay of the simulated IR spectrum of **2′-Y** (0.99 scale) and the measured ATR-IR spectrum of **2-Dy**.

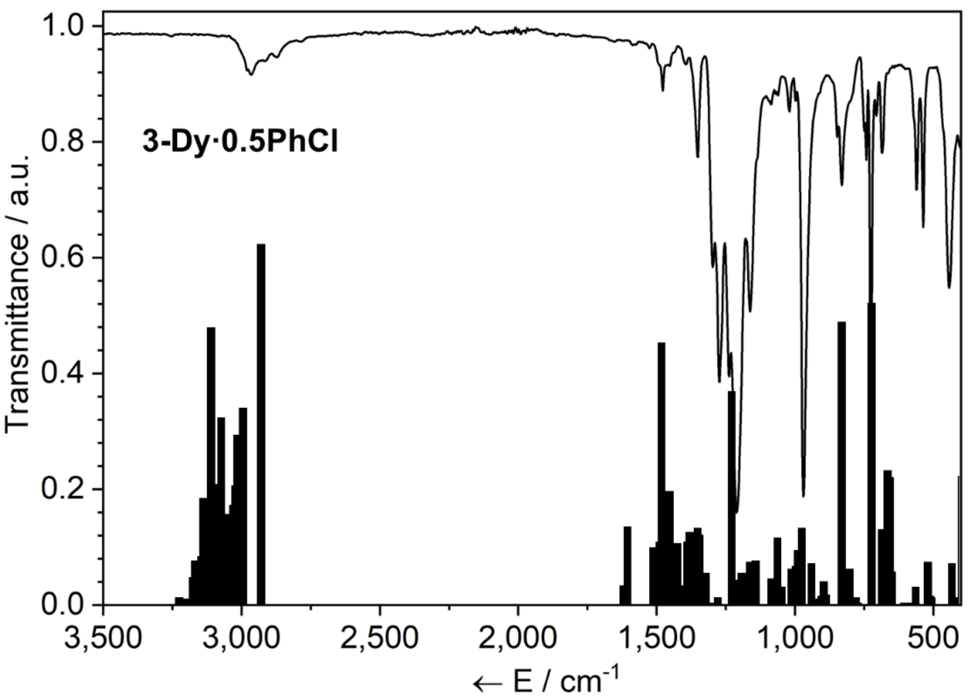

Figure S54. Overlay of the simulated IR spectrum of **3′-Y** (0.99 scale) and the measured ATR-IR spectrum of **3-Dy**.

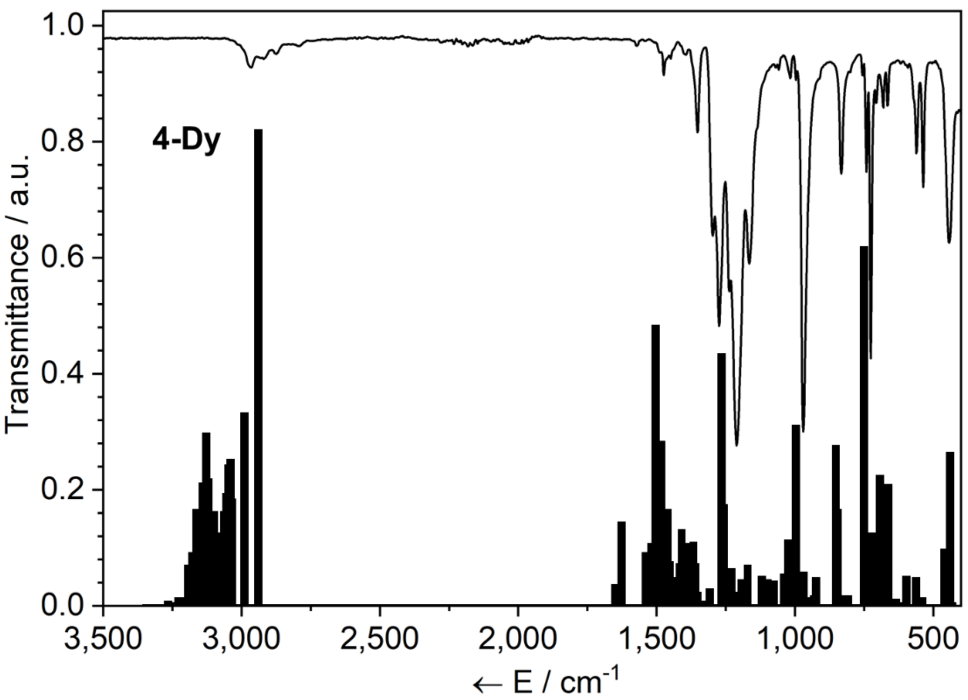

Figure S55. Overlay of the simulated IR spectrum of **4′-Y** (100 scale) and the measured ATR-IR spectrum of **4-Dy**.

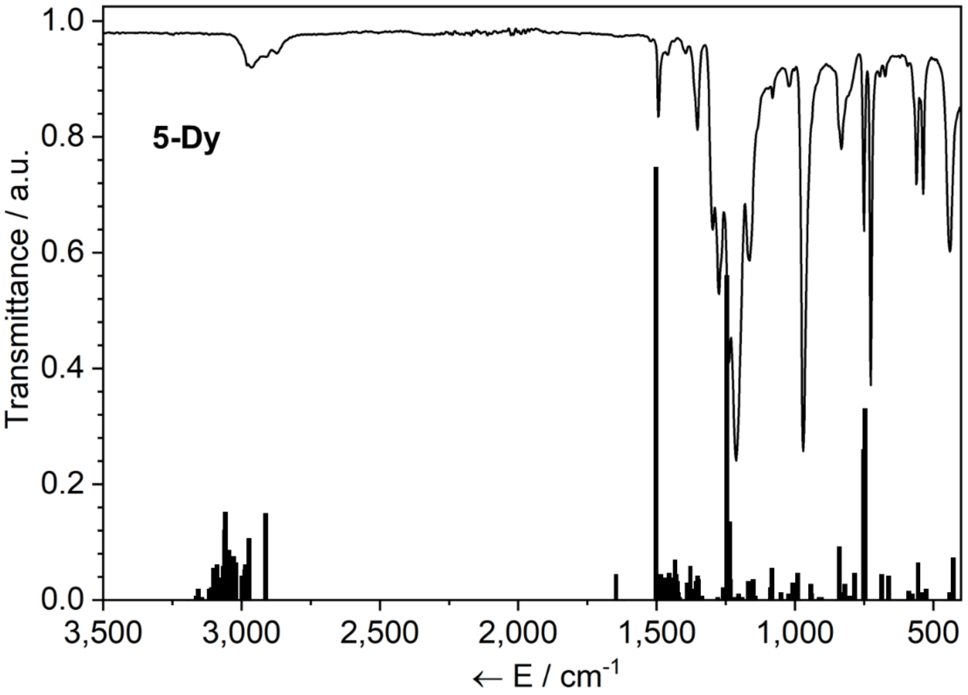

Figure S56. Overlay of the simulated IR spectrum of **5′-Y** (0.98 scale) and the measured ATR-IR spectrum of **5-Dy**.

8. Magnetic Measurements

Magnetic measurements were performed using a Quantum Design MPMS3 superconducting quantum interference device (SQUID) magnetometer. All samples were crushed with a mortar and pestle under an inert atmosphere, and then loaded into a borosilicate glass NMR tube along with eicosane, which was then evacuated and flame-sealed to give an ampoule with a length of *ca.* 3 cm. The eicosane was melted by heating the tube gently with a low-power heat gun to immobilize the crystallites. The ampoule was then mounted in the center of a drinking straw using friction by wrapping it with Kapton tape, and the straw was then fixed to the end of the sample rod. Samples of **2-Dy** (20.5 mg, 21.0 mg or 18.8 mg), **3-Dy** (20.6 mg), **4-Dy** (20.7 mg), **5-Dy** (22.9 mg) and **5%Dy@2-Y** (32.9 mg) were prepared with 13.3 mg (12.9 or 14.2 mg), 14.9 mg, 16.0 mg, 14.4 mg and 18.6 mg of eicosane, respectively. The measurements were corrected for the diamagnetism of the straw, borosilicate tube and eicosane using calibrated blanks, for the shape of the sample using Quantum Design Geometry Simulator, and for the intrinsic diamagnetism of the sample estimated as the molecular weight (g mol^{-1}) multiplied by -0.5×10^{-6} cm^3 K mol^{-1}. For measurements on **5%Dy@2-Y**, the saturation magnetization at 1.8 K and 5 T was normalized to pure **2-Dy**, and this scaling factor (approx. 0.814, corresponding to a molar percent ratio Dy:Y of 6.1:93.9) was used to correct all measurements.

All dc magnetic measurements on pure samples were performed in dc scan mode with a scan length of 40 mm and a scan time of 6 s unless specified otherwise. The equilibrium magnetic susceptibility was measured under a 0.1 T field on cooling in temperature settle mode, at 5 K min^{-1} from 300–100 K and 1 K min^{-1} from 100–1.8 K. The doped sample **5%Dy@2-Y** had a much weaker signal and so was measured under a 0.5 T field with VSM mode using 5 mm vibrational amplitude and 2 s averaging time with the same temperature settings. Zero-field cooled and field-cooled measurements were performed between 2 and 80 K under 0.1 T field in continuous temperature sweep mode with a constant sweep rate of 0.5 K min^{-1}. The

zero-field cooled sample was prepared by holding the sample at 150 K for 10 min, cooling at 10 K min^{-1} to 20 K, holding for 5 min, cooling to 2 K at 0.5 K min^{-1} and holding for 40 min before switching on the field. The field-cooled sample measured on warming was prepared by stabilizing in 0.1 T field at 2 K for 40 min, after approaching from above at 0.5 K min^{-1}. Hysteresis measurements were performed between ± 5 T at temperatures of 1.8–12 K on samples that had been magnetized at 5 T. Above 14 K, until closing of the hysteresis loop, hysteresis measurements were performed between ± 3 T on a sample that had been magnetized at 3 T. For **5-Dy**, hysteresis measurements were only performed up to 6 K. Measurements on **5%Dy@2-Y** were performed in VSM mode using 5 mm vibrational amplitude and 2 s averaging time, all between ± 5 T. All hysteresis measurements were performed in continuous sweep mode with a sweep rate of 22 Oe s^{-1} across the entire field range.

Alternating frequency (AC) susceptibility measurements were recorded for 8 frequencies per decade between 0.1–1000 Hz with a 5 Oe oscillating field. Due to instrumental limitations, 750 and 1000 Hz frequencies were measured with a 2 Oe oscillating field. Averages were performed for 2 s or for 10 cycles, whichever was longer; for the doped sample **5%Dy@2-Y**, measurements were generally averaged over 10 s or 50 cycles. Measurements were performed at 44–86 K (**2-Dy**), 36–93 K (**3-Dy**), 46–97 K (**4-Dy**), 2–73.5 K (**5-Dy**) and 8–89 K (**5%Dy@2-Y**). The high frequency end of the data was trimmed at low temperatures for **2-Dy** ($T \leq 65$ K), **3-Dy** ($T \leq 72$ K), **4-Dy** ($T \leq 70$ K) and **5-Dy** ($T \leq 33$ K). AC data were fit to the Generalised Debye model (Equation S1) in CC-FIT2 to extract relaxation rates and distributions.[22,23]

$$\chi(\omega) = \chi_S + (\chi_T - \chi_S)\frac{1}{1+(i\omega\tau_{Debye})^{1-\alpha}} \qquad \textbf{Equation S1}$$

For **5%Dy@2-Y**, three frequency-dependent peaks were observed that overlap in temperature and frequency ranges. We attempted to fit as many of the peaks as possible at once, if a peak lay significantly outside observed frequency window and could not be reliably fit, we trimmed

the data to only include contributions from the observed peak(s). We only consider relaxation times of peaks that lie within the frequency window to be reliable. For 80–86 K peak A was fit to the generalized Debye model; for 62–77 K, peaks A and B were fit to the double generalized Debye model (Equation S2); for 23–59 K, peaks A, B and C were fit to the triple generalized Debye model (Equation S3); for 8–20 K, peaks B and C were fit to the double generalized Debye model.

$$\chi(\omega) = \chi_{total} + \frac{\Delta_{\chi,B}}{1+(i\omega\tau_{Debye,B})^{1-\alpha_B}} + \frac{\Delta_{\chi,A/C}}{1+(i\omega\tau_{Debye,A/C})^{1-\alpha_{A/C}}} \qquad \textbf{Equation S2}$$

$$\chi(\omega) = \chi_{total} + \frac{\Delta_{\chi,A}}{1+(i\omega\tau_{Debye,A})^{1-\alpha_A}} + \frac{\Delta_{\chi,B}}{1+(i\omega\tau_{Debye,B})^{1-\alpha_B}} + \frac{\Delta_{\chi,C}}{1+(i\omega\tau_{Debye,C})^{1-\alpha_C}} \qquad \textbf{Equation S3}$$

All fitting was performed in CC-FIT2,[22,23] with customized scripts for the triple generalized Debye model. Consistently negative values of χ' are observed at high frequencies between approx. 20–50 K for **5%Dy@2-Y**, indicating the sum of the adiabatic susceptibilities of each component (χ_{total}) is negative because of a large diamagnetic contribution from the doped sample. Freely refined values of χ_{total} (8–38 K) obey a Curie law (χ_{total} = –0.03794 + 0.60981/T, R^2 = 0.989, weighted by uncertainties). Above 38 K, χ_{total} cannot be reliably fit as not all of peak C is observed and χ_{total} is highly correlated with $\Delta_{\chi,C}$, so we used the Curie Law to fix the value of χ_{total} for the 41–59 K datasets. Note that above 60 K the definition of χ_{total} includes the isothermal contribution of component C. Therefore this value is positive, and is restrained to be so.

DC magnetisation decay measurements were performed on **2-Dy** (2–14 K), **3-Dy** (2–20 K), **4-Dy** (2–24 K) and **5%Dy@2-Y** (2–16 K). The sample was saturated in a 30 kOe magnetic field for a minimum of 5 minutes (magnetic moment M_{sat}), then the field was rapidly removed (700 Oe s^{-1}) to the calibrated target of +22.5 Oe and the magnetic moment was measured as a function of time as soon as the field stabilized. The target field corresponds

to a zero actual field, as determined using an identical charging sequence with a palladium reference sample at 298 K. Measurements were performed with DC scan mode with 40 mm scan length and 6 s scan time. All compounds exhibited non-stretched exponential behaviour at very long timescales in zero field once most of the relaxation had elapsed; this was observed for all temperatures for **4-Dy** and **5%Dy@2-Y**, and at 6 K and above for **2-Dy** and **3-Dy**. The very small fraction of a slower relaxing impurity has been observed for similar compounds and is not representative of the entire sample,[23] so decay curves were trimmed after 99% of the decay (1% of M_0),[23] where M_0 is the initial magnetisation in zero field. Exceptions were 14–20 K for **3-Dy**, which showed the onset of non-stretched exponential behavior earlier, and datapoints with $M < 10^{-4}$ emu were discarded. Additionally, 2 K datasets for **2-Dy** were measured until 14.6% of M_0 or 24.4 h, and **3-Dy**, measured until 3.5% of M_0 or 18.7 h due to the long times involved. High temperature datasets with $M_0/M_{sat} < 0.07$ were excluded[1] as they are at the limit of what can be measured by the technique and may not be representative of the sample. Datapoints with incorrectly reported timestamps were removed.[1]

Processed decay data were fit to the stretched exponential model (Equation S4) in OriginPro:[24]

$$M(t) = M_0 e^{-\left(\frac{t}{\tau*}\right)^{\beta}} \qquad \textbf{Equation S4}$$

where τ* is the "characteristic" relaxation time and β is a stretching parameter. M_0 was fixed to the magnetic moment of the first point measured in the target field ($t = 0$), and the equilibrium value was assumed to be zero (in calibrated zero field). The representative value of the relaxation time for AC and magnetisation decay measurements is given by $e^{\langle \ln \tau \rangle}$,[23] which is equal to τ_{Debye} in AC measurements. The one standard deviation limits of the relaxation time ($\tau_{\pm}$) were defined using the variance in $\ln \tau$ ($\sigma^2_{\ln \tau}$) for both ac and magnetization decay data.[23] The distribution values are derived using equations reported by Zorn.[25]

For a generalized Debye model:

$$\tau_{\pm} = e^{\langle \ln\tau \rangle \pm \sqrt{\sigma_{\ln\tau}^2}} \qquad \textbf{Equation S5}$$

where

$$\sigma_{\ln\tau}^2 = \frac{\pi^2}{3}\left(\frac{1}{(1-\alpha)^2} - 1\right)$$

For a stretched exponential function:

$$\langle \ln\tau \rangle = \ln\tau^* + \left(1 - \frac{1}{\beta}\right)\mathrm{Eu} \qquad \textbf{Equation S6}$$

where Eu is Euler's constant (0.5772…) and

$$\sigma_{\ln\tau}^2 = \frac{\pi^2}{6}\left(\frac{1}{\beta^2} - 1\right)$$

For **5-Dy** and **5%Dy@2-Y** (peak A slowest ac component and magnetization decay rates) the temperature-dependence of the rates in zero-field was fit to:

$$\log_{10}[\tau^{-1}] = \log_{10}\left[10^{-A}e^{-\left(\frac{U_{\mathrm{eff}}}{k_{\mathrm{B}}T}\right)} + 10^{R}T^{n} + 10^{-Q}\right] \qquad \textbf{Equation S7}$$

where 10^{-A} s^{-1} ($\tau_0{}^{-1}$) is the Orbach prefactor, U_{eff} is the effective energy barrier for the Orbach process, 10^{R} s^{-1} K^{-n} (C) and n are phenomenological parameters that describe the Raman process, and 10^{-Q} s^{-1} ($\tau_{\mathrm{QTM}}{}^{-1}$) is the quantum tunnelling of magnetisation (QTM) rate. Rates for **2-Dy** cannot be fit to Equation S7 as no plateau is observed, fitting to only the Orbach and Raman process (Equation S8) is shown for comparison (Figure S119, Table S26) but cannot accurately describe the relaxation behaviour. For **3-Dy** rates, fits were performed with the QTM term (Equation S7) and without it (Equation S8).

$$\log_{10}[\tau^{-1}] = \log_{10}\left[10^{-A}e^{-\left(\frac{U_{\mathrm{eff}}}{k_{\mathrm{B}}T}\right)} + 10^{R}T^{n}\right] \qquad \textbf{Equation S8}$$

The QTM rate obtained for **3-Dy** is much slower than expected, so the fit without QTM is preferred (Figures S120–S121, Table S27).

The rates for **2-Dy** and **4-Dy** were fit considering a phonon bottleneck process. For **4-Dy** the phonon bottleneck was considered to limit the Raman and QTM processes according to Equation S9.

$$\log_{10}[\tau^{-1}] = \log_{10}\left[10^{-A}e^{-\left(\frac{U_{\text{eff}}}{k_{\text{B}}T}\right)} + \frac{1}{10^{B}T^{-m}+[10^{R}T^{n}+10^{-Q}]^{-1}}\right] \quad \textbf{Equation S9}$$

We derive Equation S9 by reference to Rousset *et al*[26]. including the QTM and Raman processes in the phonon bottleneck, while any phonon bottleneck effect on the Orbach process renormalizes the characteristic relaxation time, τ_0:[27]

$$\tau^{-1} = \tau_{\text{Orbach}}^{-1} + \tau_{\text{SB,bottleneck}}^{-1}$$

Where the bottlenecked spin-bath relaxation time, $\tau_{\text{SB,bottleneck}}$ is the sum of the lattice-bath (τ_{LB}) relaxation time, weighted by the ratio of spin and lattice heat capacities, and the spin-lattice (τ_{SL}) relaxation time:[28]

$$\tau_{\text{SB,bottleneck}} = \left(\frac{C_S}{C_L}\right)\tau_{\text{LB}} + \tau_{\text{SL}}$$

The first term is assumed to have a power-law temperature dependence:

$$\left(\frac{C_S}{C_L}\right)\tau_{\text{LB}} = 10^{B}T^{-m}$$

And the spin-lattice relaxation rate is given by either the sum of Raman and QTM processes (**4-Dy**) or the Raman process only (**2-Dy**):

$$\tau_{\text{SL}}^{-1} = 10^{R}T^{n} + 10^{-Q}$$

Fitting of the relaxation rates was performed in CC-FIT2,[22,23] where fits were performed with points weighted by the reciprocal of the logarithmic variance ($\sigma_{\ln\tau}^{2}$).

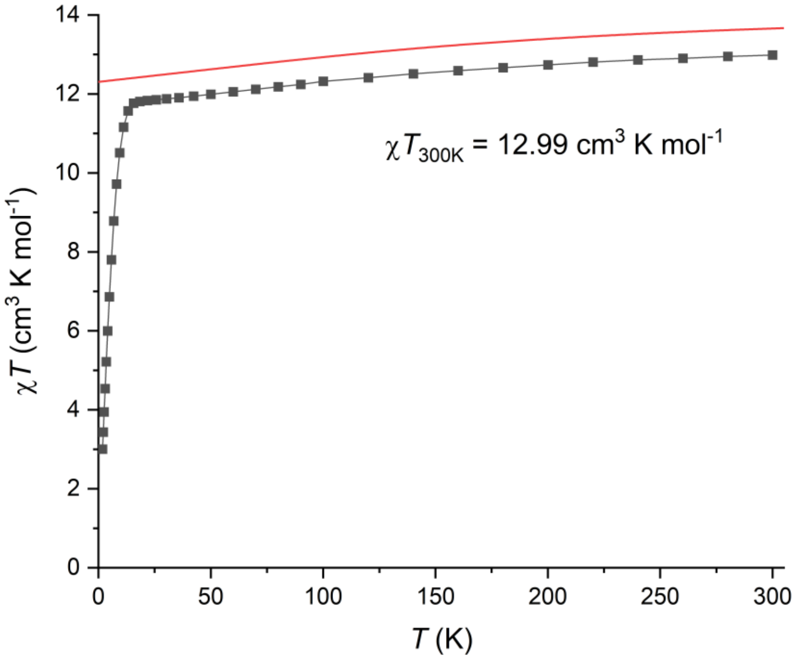

Figure S57. Temperature dependence of the molar magnetic susceptibility $\chi_M T$ product for powdered **2-Dy** measured under a 0.1 T applied magnetic field (black) and predicted values from CASSCF (red).

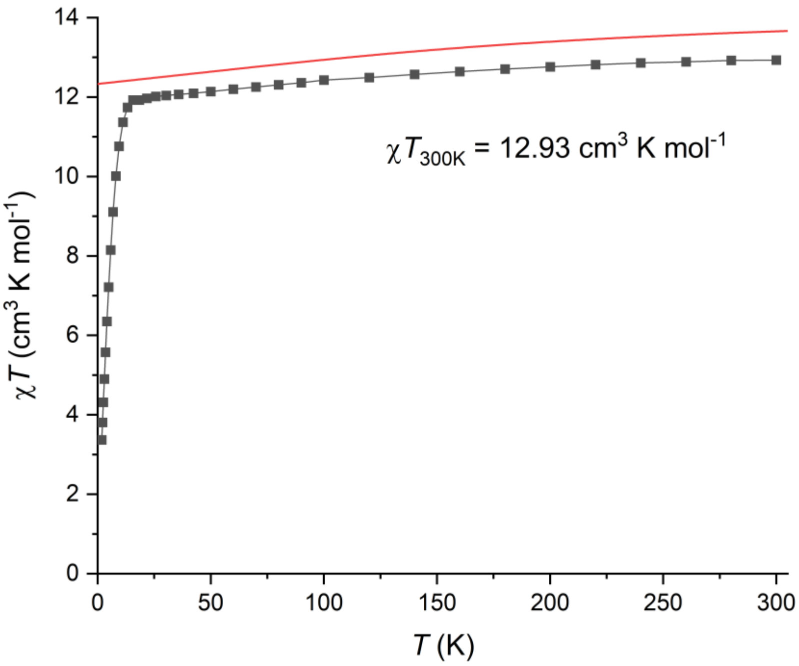

Figure S58. Temperature dependence of the molar magnetic susceptibility $\chi_M T$ product for powdered **3-Dy** measured under a 0.1 T applied magnetic field (black) and predicted values from CASSCF (red).

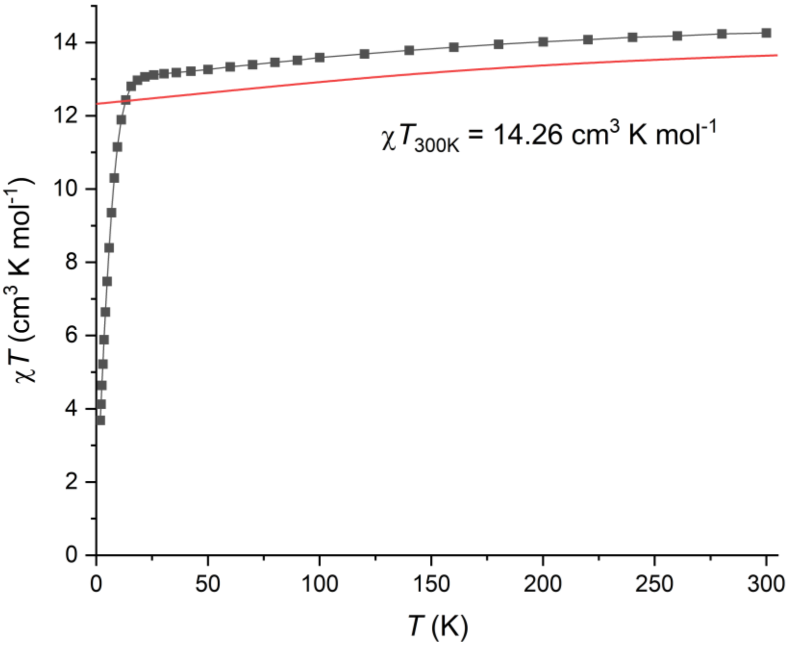

Figure S59. Temperature dependence of the molar magnetic susceptibility χ_MT product for powdered **4-Dy** measured under a 0.1 T applied magnetic field (black) and predicted values from CASSCF (red).

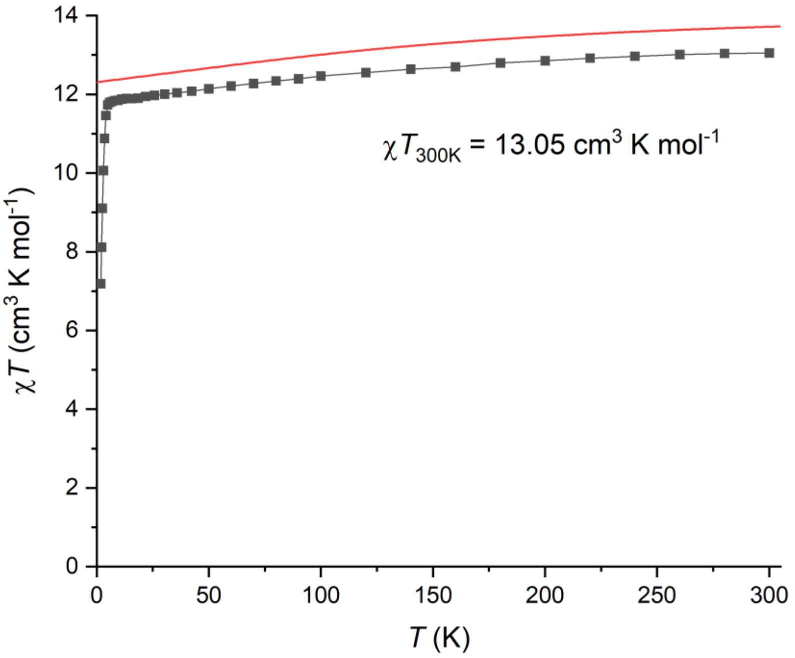

Figure S60. Temperature dependence of the molar magnetic susceptibility χ_MT product for powdered **5-Dy** measured under a 0.1 T applied magnetic field (black) and predicted values from CASSCF (red).

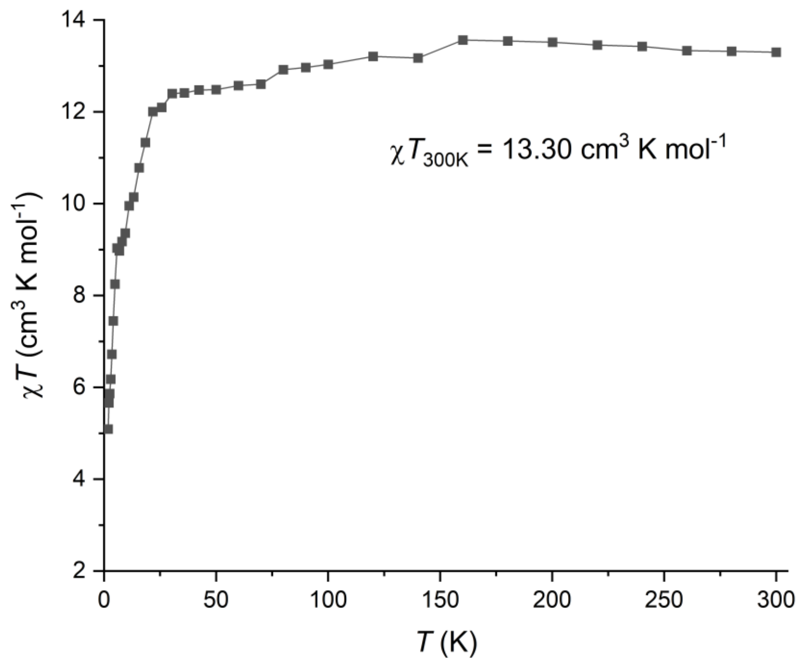

Figure S61. Temperature dependence of the molar magnetic susceptibility $\chi_M T$ product for powdered **5%Dy@2-Y** measured under a 0.5 T applied magnetic field (black).

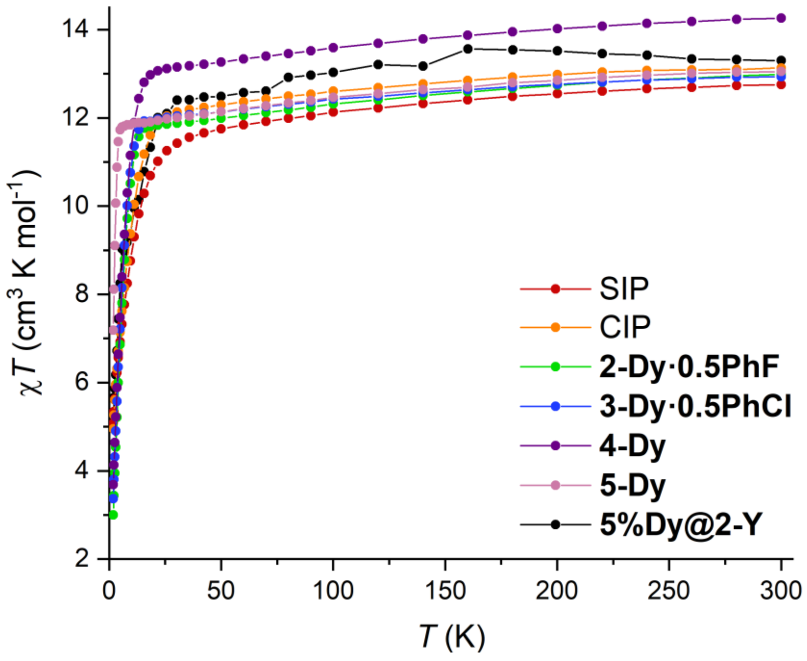

Figure S62. Combined temperature dependence of the molar magnetic susceptibility $\chi_M T$ products under a 0.1 T applied magnetic field (0.5 T for **5%Dy@2-Y**).

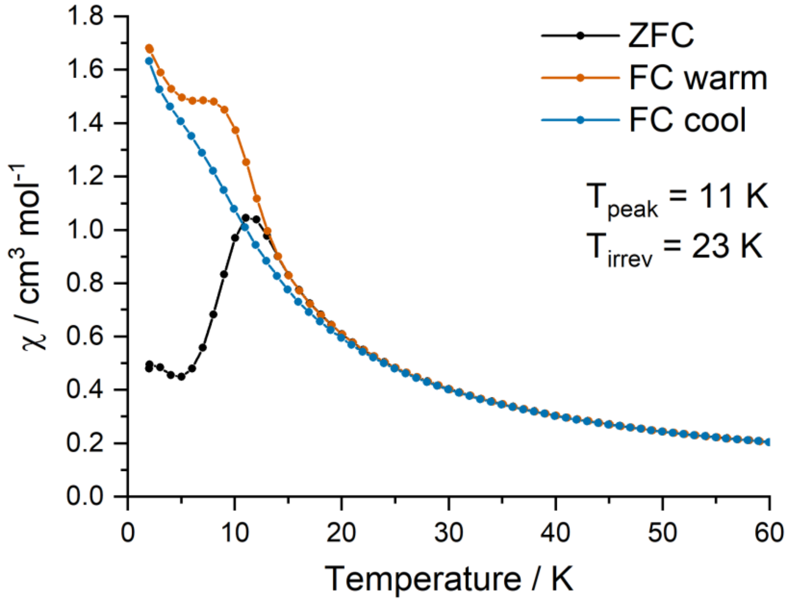

Figure S63. Magnetic susceptibility (χ) *vs*. temperature (K) for **2-Dy** measured on warming after cooling in zero field (ZFC, gray) and measured on cooling in field (FC cool, blue) and warming in field (FC warm, red).

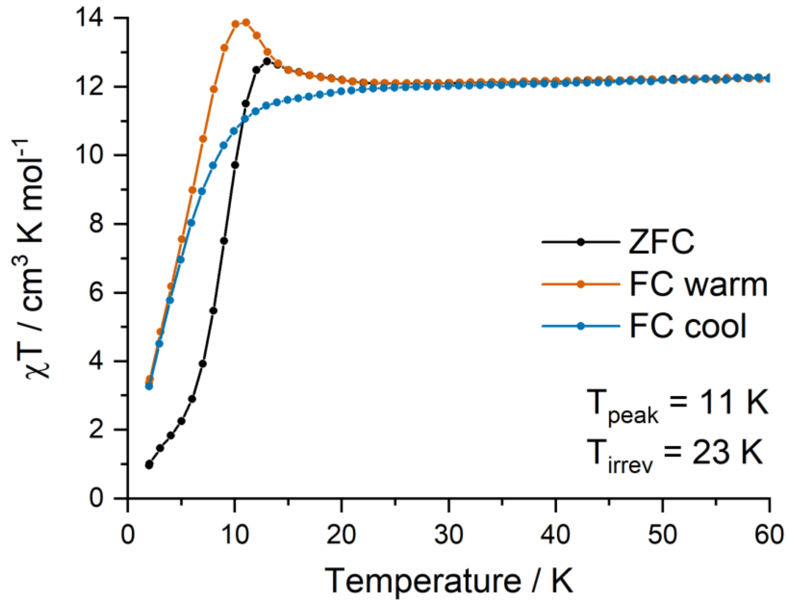

Figure S64. Magnetic susceptibility temperature product (χT) *vs*. temperature (K) for **2-Dy** measured on warming after cooling in zero field (ZFC, gray) and measured on cooling in field (FC cool, blue) and warming in field (FC warm, red).

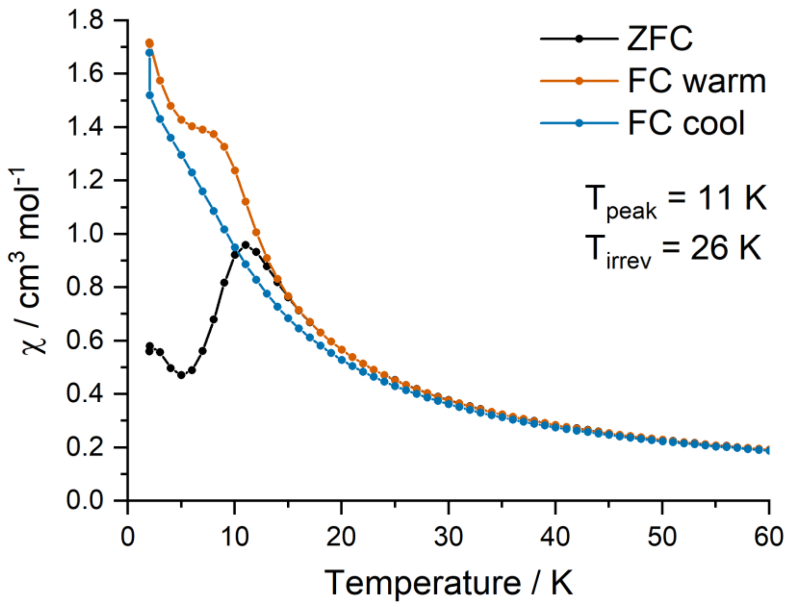

Figure S65. Magnetic susceptibility (χ) *vs*. temperature (K) for **3-Dy** measured on warming after cooling in zero field (ZFC, gray) and measured on cooling in field (FC cool, blue) and warming in field (FC warm, red).

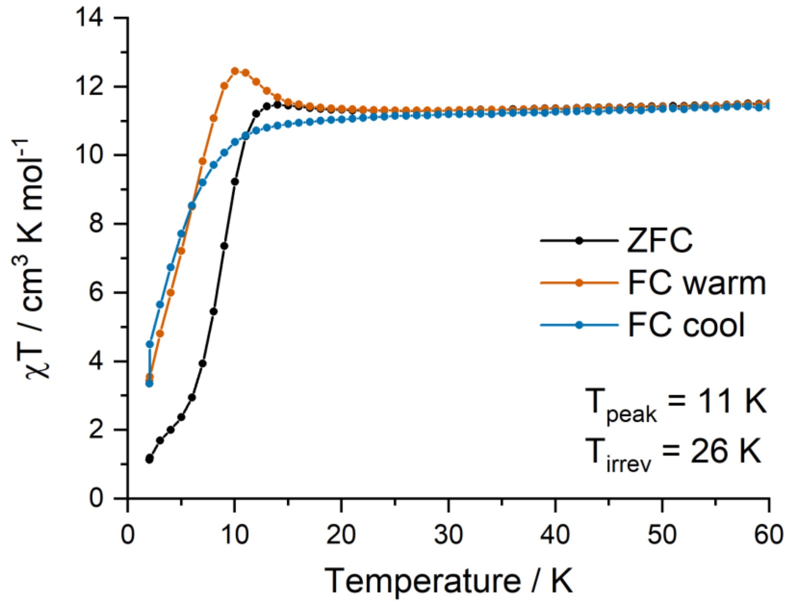

Figure S66. Magnetic susceptibility temperature product (χT) *vs*. temperature (K) for **3-Dy** measured on warming after cooling in zero field (ZFC, gray) and measured on cooling in field (FC cool, blue) and warming in field (FC warm, red).

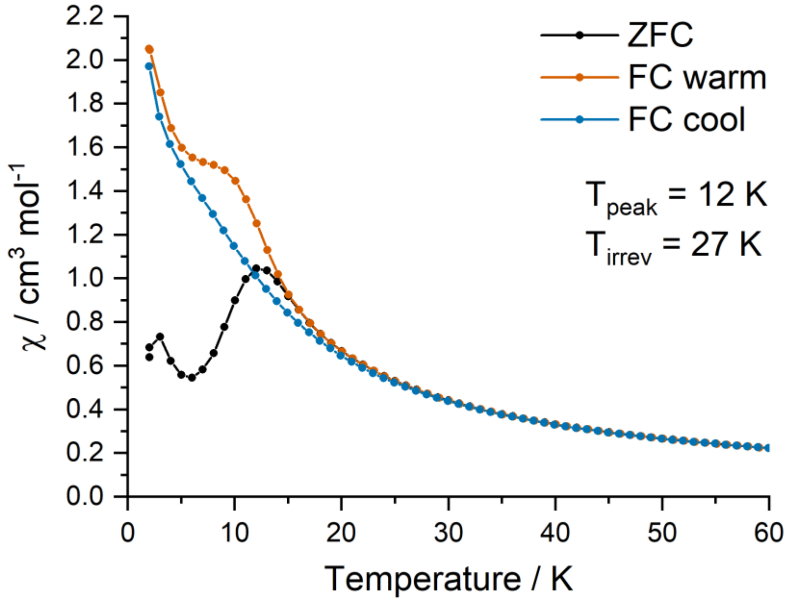

Figure S67. Magnetic susceptibility (χ) *vs*. temperature (K) for **4-Dy** measured on warming after cooling in zero field (ZFC, gray) and measured on cooling in field (FC cool, blue) and warming in field (FC warm, red).

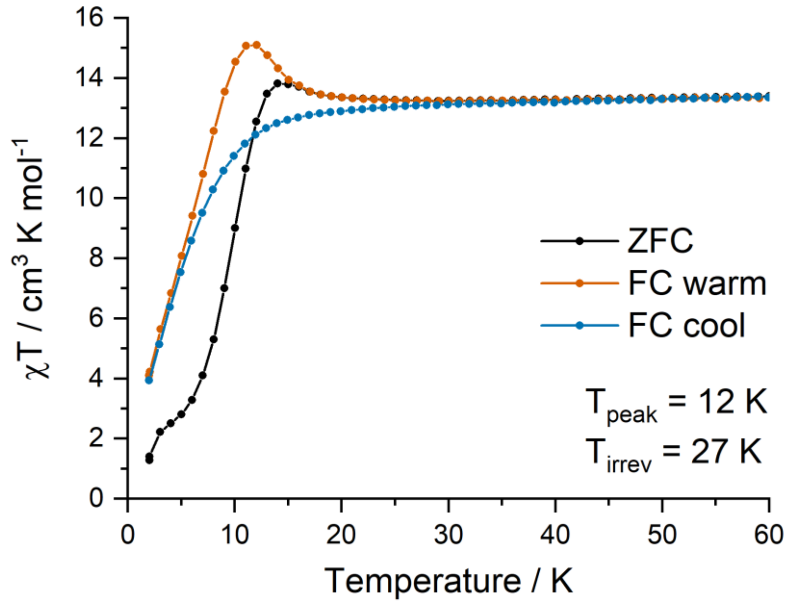

Figure S68. Magnetic susceptibility temperature product (χT) *vs*. temperature (K) for **4-Dy** measured on warming after cooling in zero field (ZFC, gray) and measured on cooling in field (FC cool, blue) and warming in field (FC warm, red).

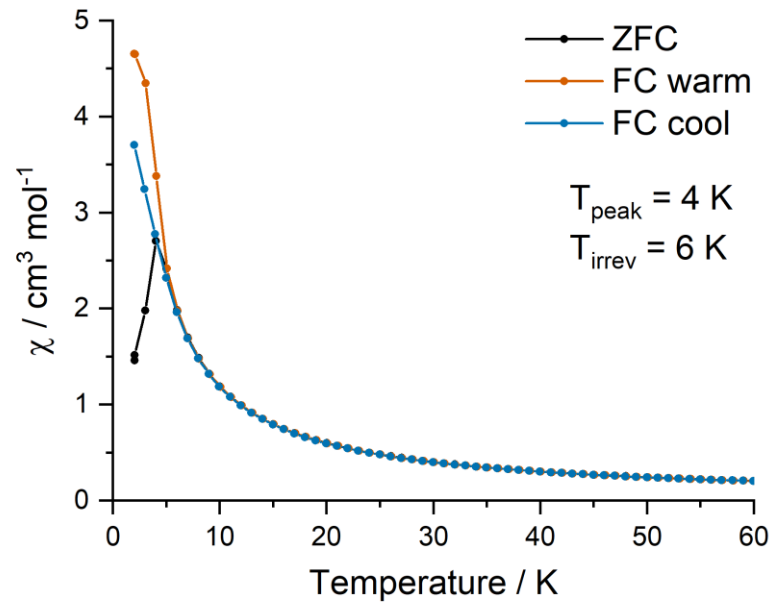

Figure S69. Magnetic susceptibility (χ) *vs*. temperature (K) for **5-Dy** measured on warming after cooling in zero field (ZFC, gray) and measured on cooling in field (FC cool, blue) and warming in field (FC warm, red).

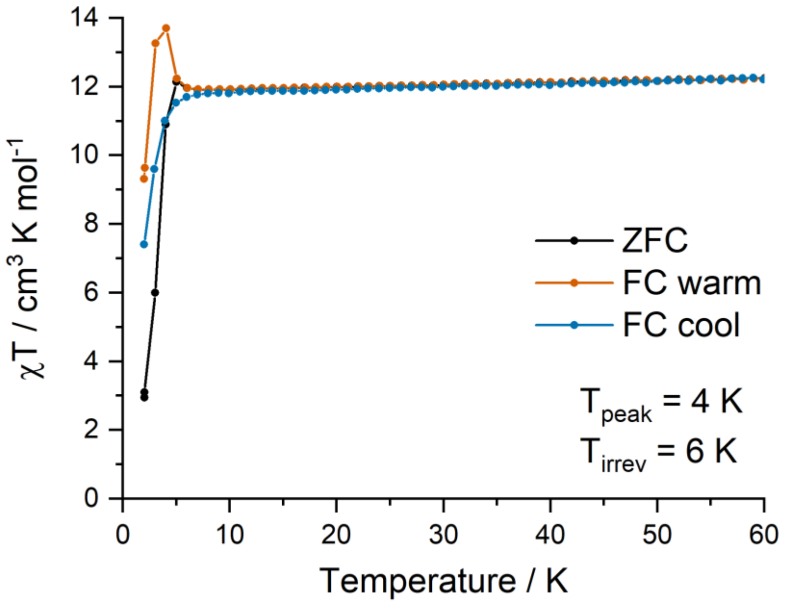

Figure S70. Magnetic susceptibility temperature product (χT) *vs*. temperature (K) for **5-Dy** measured on warming after cooling in zero field (ZFC, gray) and measured on cooling in field (FC cool, blue) and warming in field (FC warm, red).

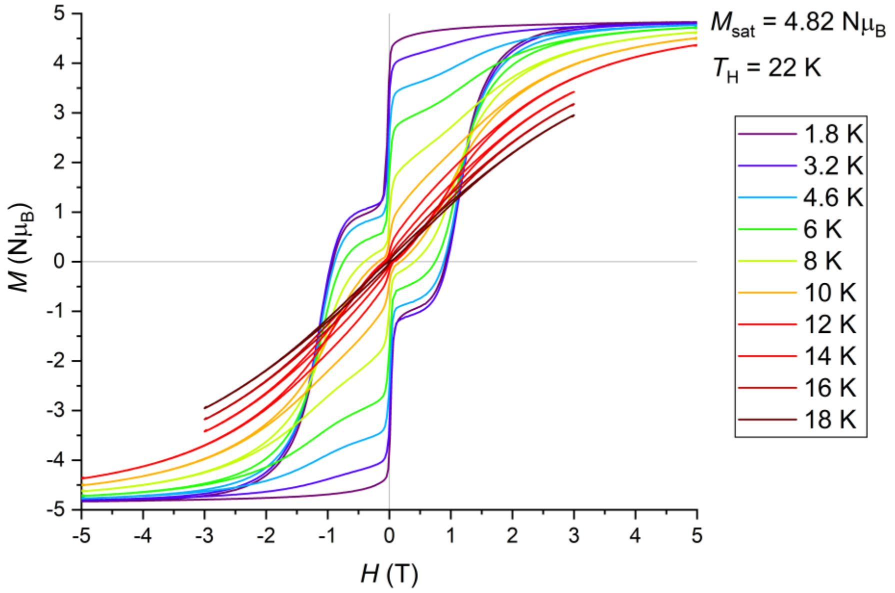

Figure S71. Hysteresis loops of **2-Dy** from –5 T to +5 T (1.8–12 K) and –3 T to +3 T (14–18 K). Sweep rate is 22 Oe s^{-1}.

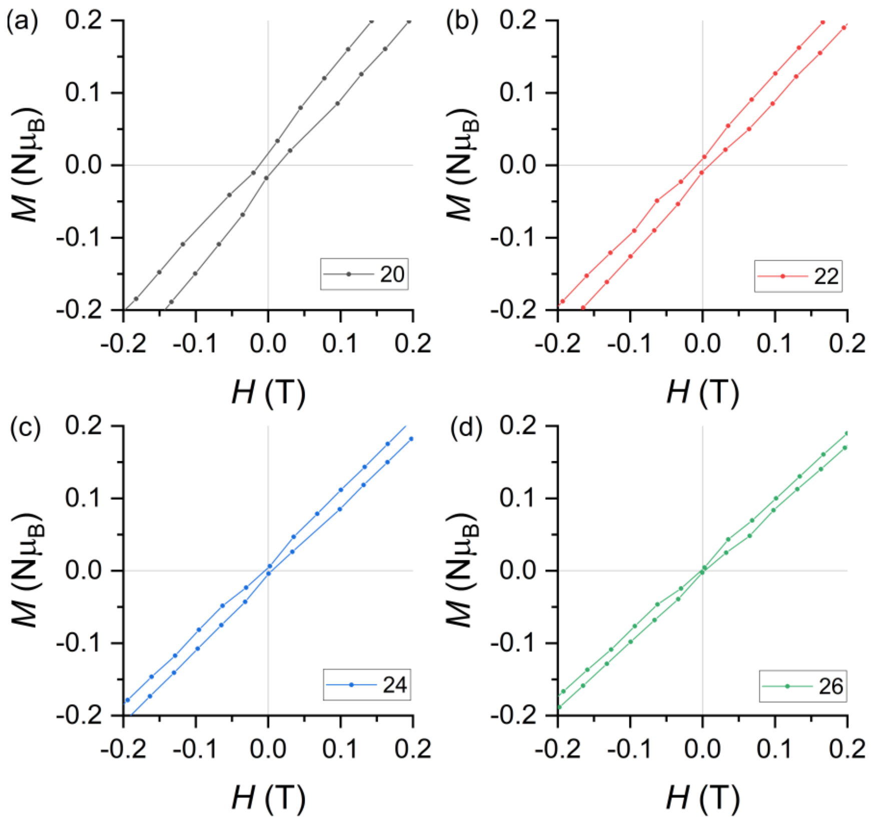

Figure S72. Hysteresis loops of **2-Dy** at (a) 20 K, (b) 22 K, (c) 24 K and (d) 26 K from –3 T to +3 T, zoomed in between –0.2 and +0.2 T. Sweep rate is 22 Oe s^{-1}.

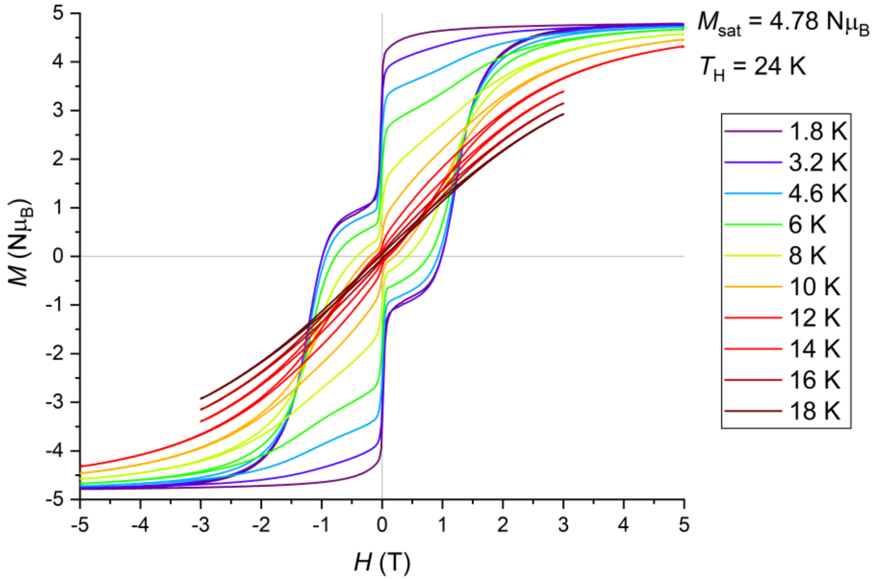

Figure S73. Hysteresis loops of **3-Dy** from –5 T to +5 T (1.8–12 K) and –3 T to +3 T (14–18 K). Sweep rate is 22 Oe s^{-1}.

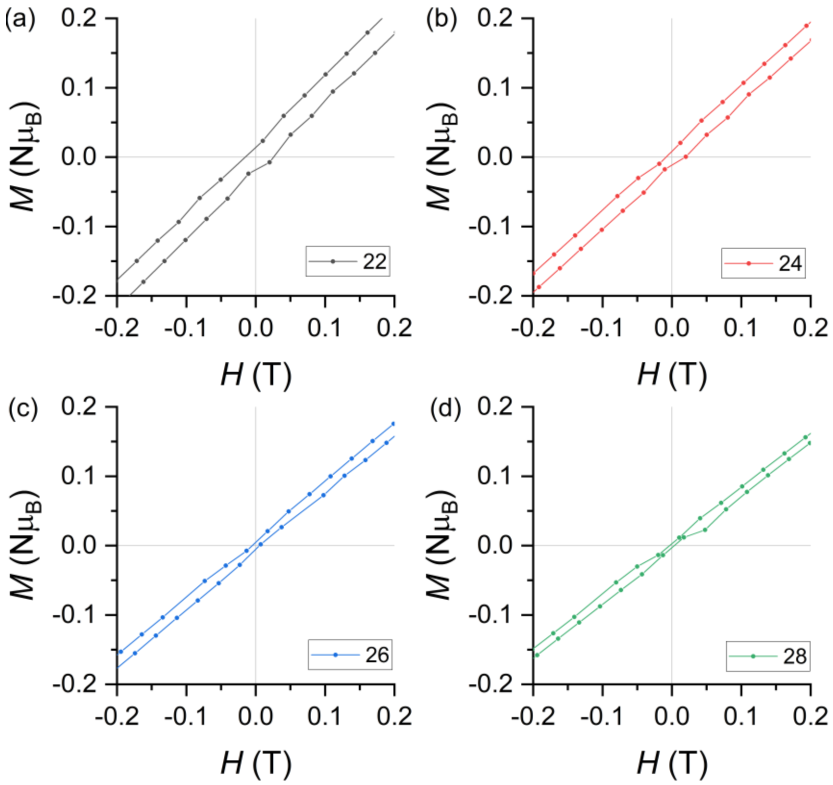

Figure S74. Hysteresis loops of **3-Dy** at (a) 22 K, (b) 24 K, (c) 26 K and (d) 28 K from –3 T to +3 T, zoomed in between –0.2 and +0.2 T. Sweep rate is 22 Oe s^{-1}.

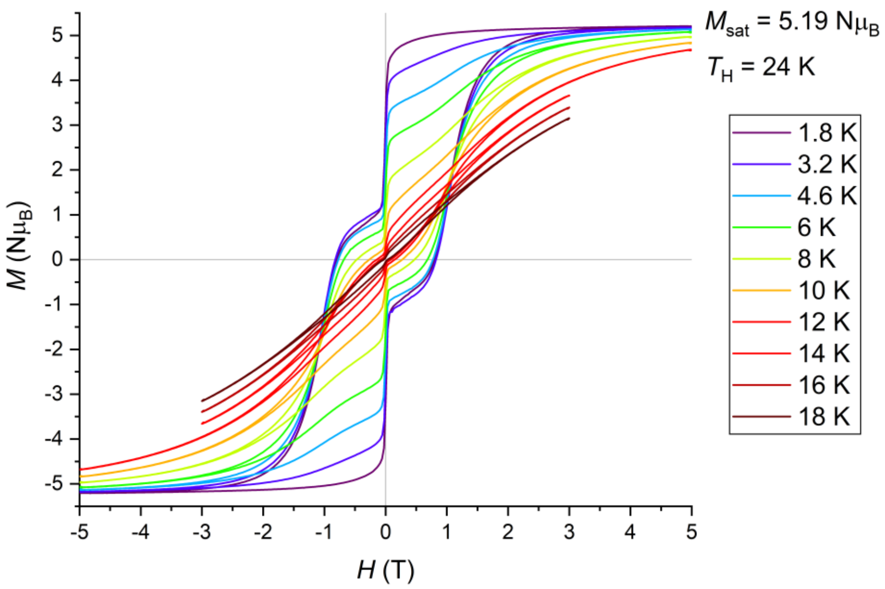

Figure S75. Hysteresis loops of **4-Dy** from –5 T to +5 T (1.8–12 K) and –3 T to +3 T (14–18 K). Sweep rate is 22 Oe s^{-1}.

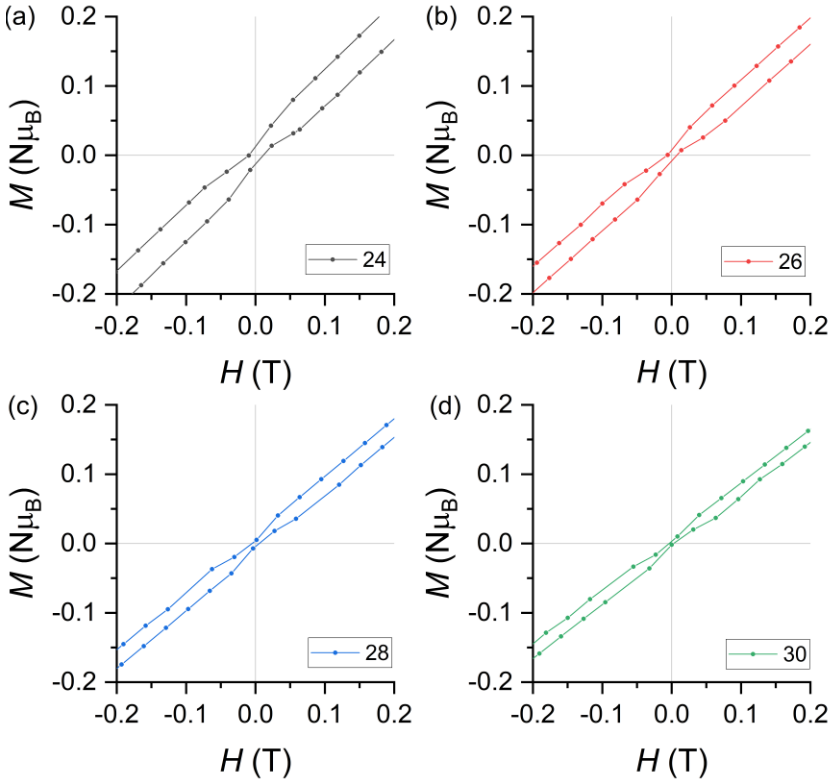

Figure S76. Hysteresis loops of **4-Dy** at (a) 24 K, (b) 26 K, (c) 28 K and (d) 30 K from –3 T to +3 T, zoomed in between –0.2 and +0.2 T. Sweep rate is 22 Oe s^{-1}.

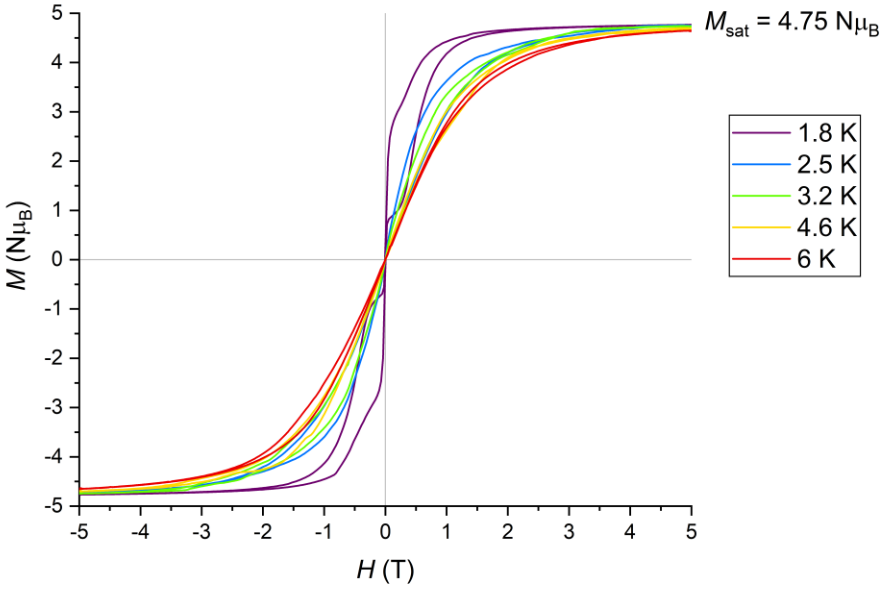

Figure S77. Hysteresis loops of **5-Dy** from 1.8 to 6 K and –5 T to +5 T. Sweep rate is 22 Oe s^{-1}.

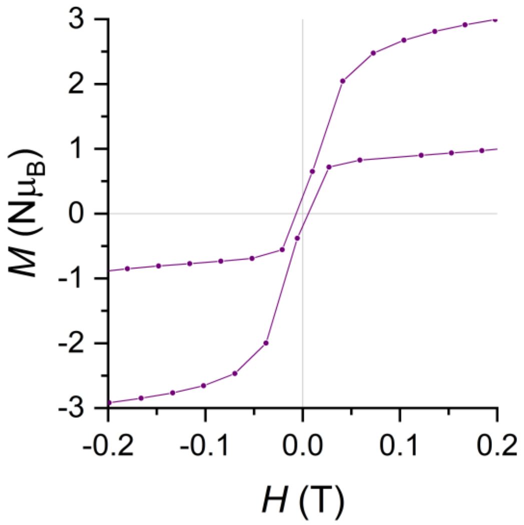

Figure S78. Hysteresis loop of **5-Dy** at 1.8 K from –3 T to +3 T, zoomed in between –0.2 and +0.2 T. Sweep rate is 22 Oe s^{-1}.

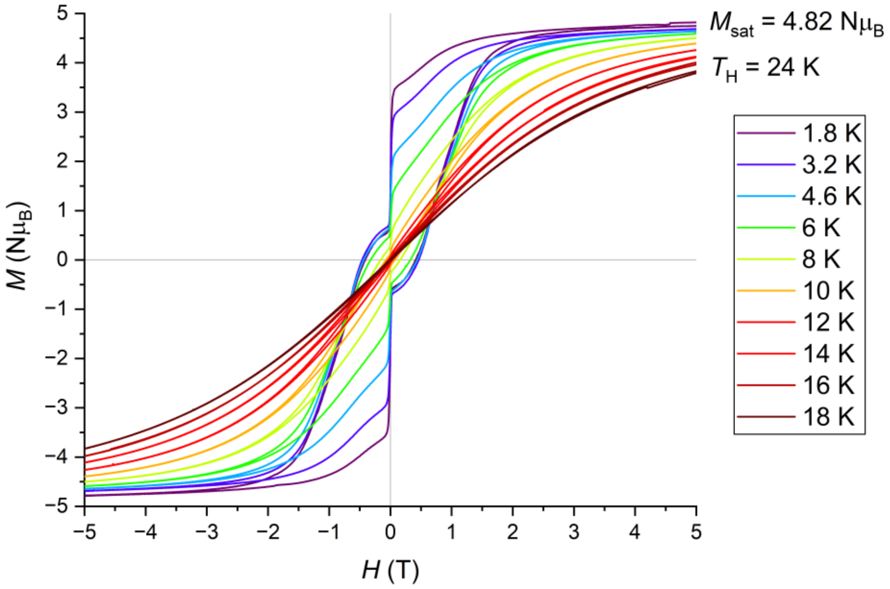

Figure S79. Hysteresis loops of **5%Dy@2-Y** from –5 T to +5 T (1.8–18 K). Sweep rate is 22 Oe s^{-1}. Scaled so M_{sat} at 1.8 K, 5 T matches **2-Dy** (scaling factor 0.814).

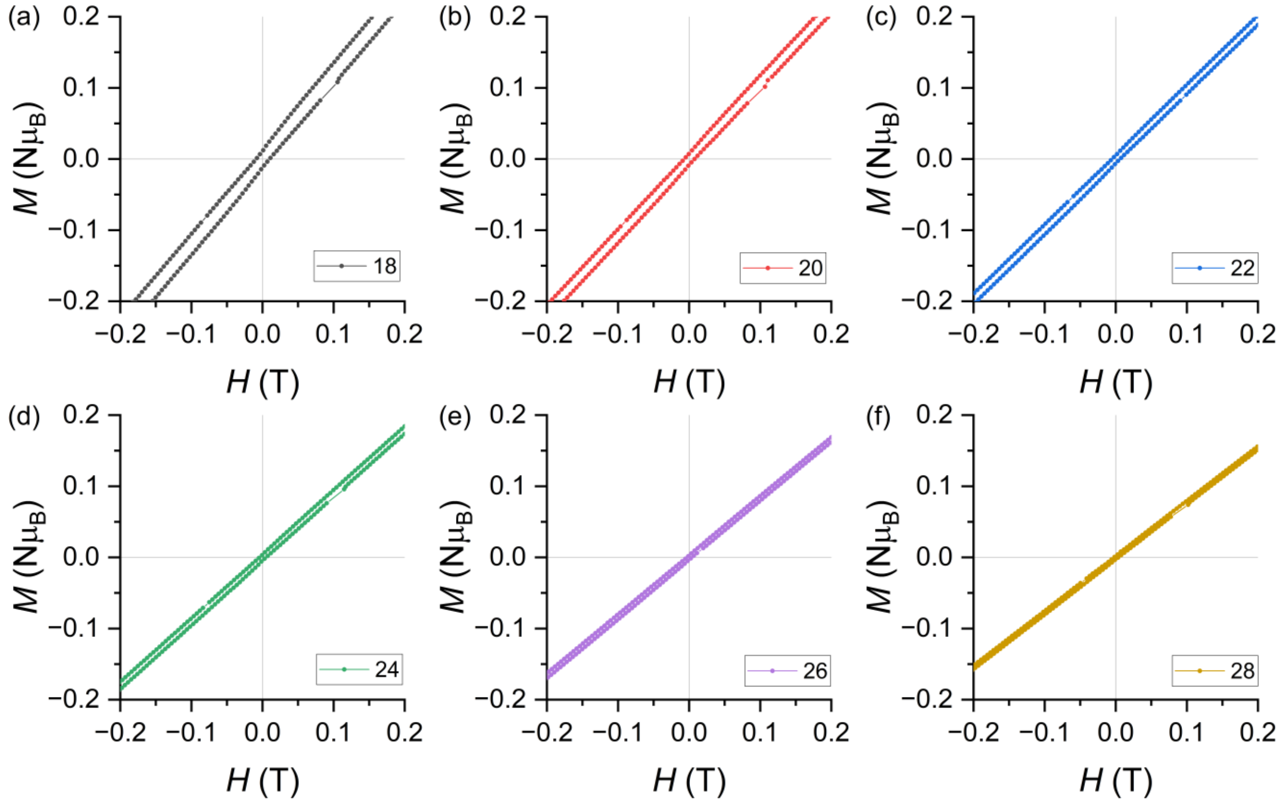

Figure S80. Hysteresis loops of **5%Dy@2-Y** at (a) 18 K, (b) 20 K, (c) 22 K, (d) 24 K, (e) 26 K and (f) 28 K from –5 T to +5 T, zoomed in between –0.2 and +0.2 T. Sweep rate is 22 Oe s^{-1}. Scaled so M_{sat} at 1.8 K, 5 T matches **2-Dy** (scaling factor 0.814).

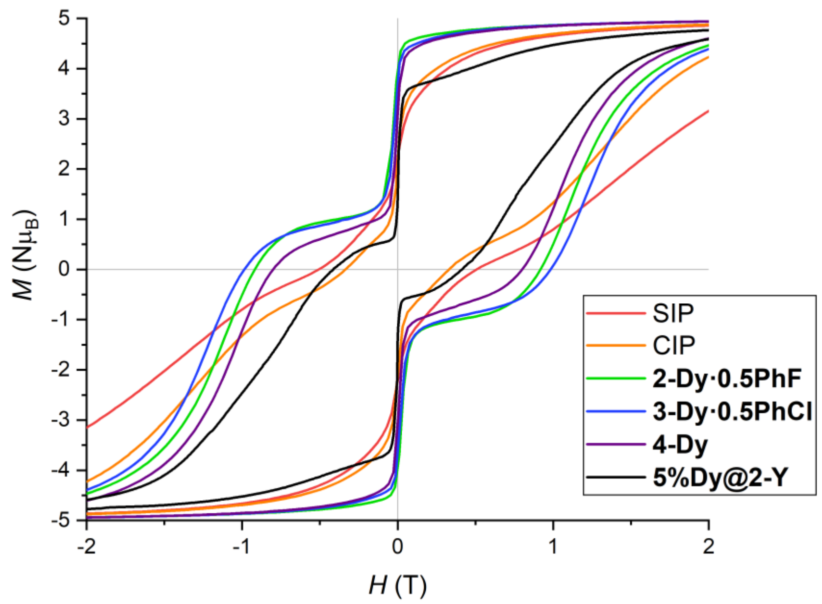

Figure S81. Comparison of the hysteresis loops at 1.8 K for SIP, CIP, **2-4-Dy** and **5%Dy@2-Y**, zoomed in between –2 and +2 T and normalized so the magnetization value at 5 T is 5.00 $N\mu_B$.

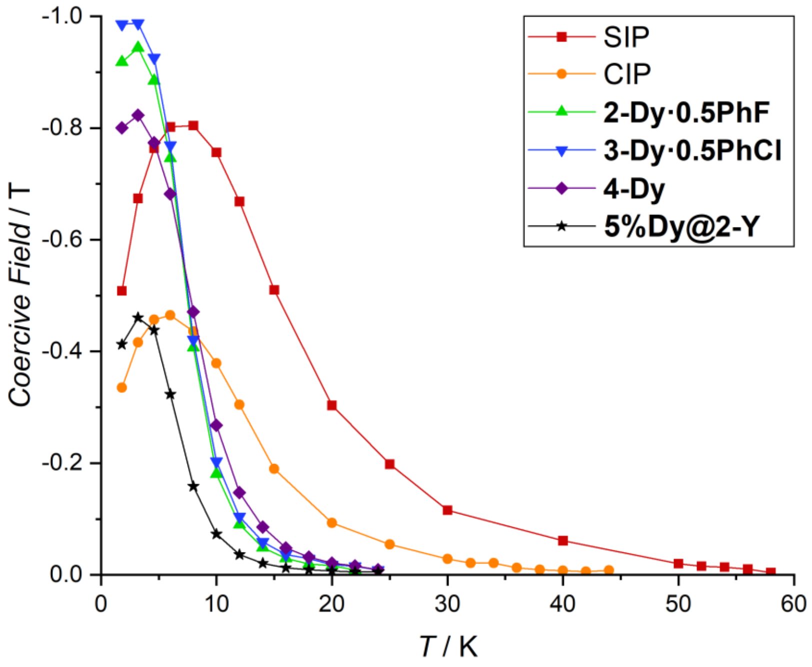

Figure S82. Comparison of the temperature dependence of coercive fields for SIP, CIP, **2-4-Dy** and **5%Dy@2-Y**; from interpolation between points on the sweep from positive to negative field.

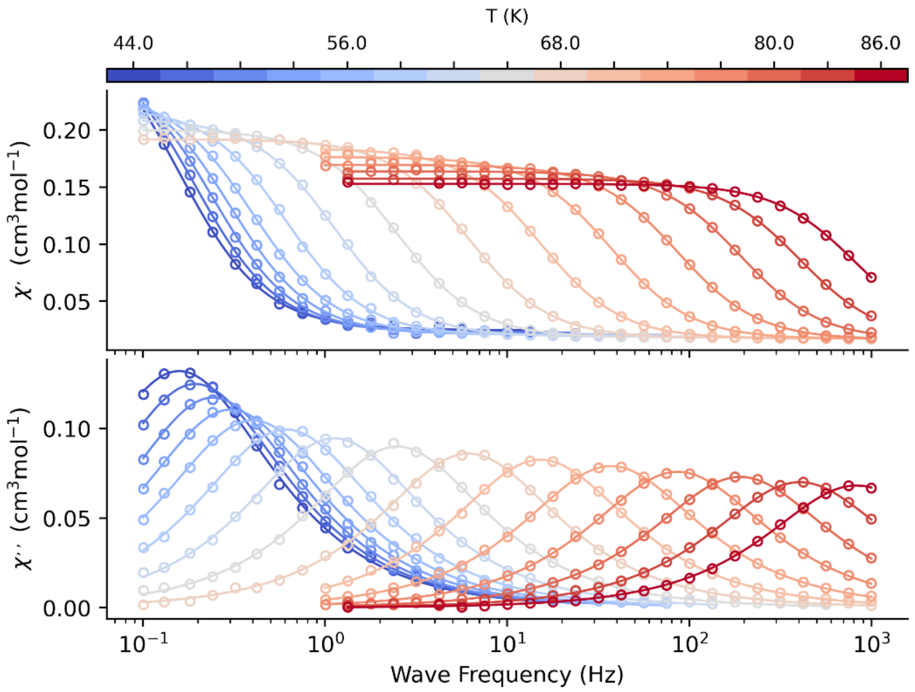

Figure S83. In-phase (top) and out-of-phase (bottom) ac susceptibilities of **2-Dy** in a zero field. Solid lines are fits to the generalized Debye model in CC-FIT2,[22,23] giving $0.00569 \leq \alpha \leq 0.0525$.

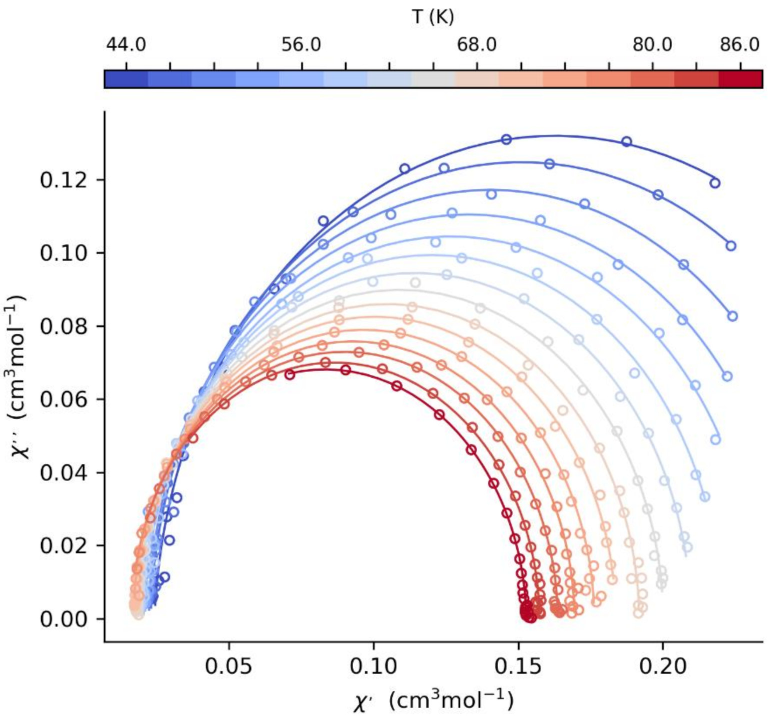

Figure S84. Cole-Cole plot showing fitting of ac data for **2-Dy** in a zero dc field. Solid lines are fits to the generalized Debye model in CC-FIT2,[22,23] giving $0.00569 \leq \alpha \leq 0.0525$.

Table S14. Best fit parameters to the generalized Debye model for **2-Dy** in zero dc field.

T	τ_{debye}	τ_{Debye}^{err}	χ_S	χ_S^{err}	χ_T	χ_T^{err}	α	α^{err}	$\langle \ln \tau \rangle$	$\sigma_{\langle \ln \tau \rangle}$
(K)	(s)		(emu/mol)		(emu/mol)				ln (s)	
44.00	1.03	2.09E-2	2.11E-2	4.82E-4	3.07E-1	4.11E-3	5.25E-2	8.07E-3	0.0089	0.4578
47.00	8.28E-1	1.17E-2	2.04E-2	4.04E-4	2.82E-1	2.68E-3	3.30E-2	6.55E-3	-0.1959	0.3327
50.00	6.60E-1	7.27E-3	2.02E-2	3.64E-4	2.61E-1	1.92E-3	1.81E-2	5.77E-3	-0.4173	0.1778
53.00	5.27E-1	4.56E-3	1.95E-2	3.13E-4	2.46E-1	1.37E-3	1.68E-2	4.84E-3	-0.6406	0.2245
56.00	3.97E-1	3.26E-3	1.93E-2	3.23E-4	2.34E-1	1.16E-3	1.78E-2	4.81E-3	-0.9209	0.2655
59.00	2.60E-1	1.70E-3	1.92E-2	2.87E-4	2.21E-1	7.93E-4	9.77E-3	4.05E-3	-1.3447	0.1665
62.00	1.42E-1	6.65E-4	1.85E-2	2.24E-4	2.10E-1	4.63E-4	8.74E-3	2.93E-3	-1.9525	0.2019
65.00	6.40E-2	2.41E-4	1.82E-2	1.93E-4	2.00E-1	2.97E-4	8.15E-3	2.34E-3	-2.7485	0.2263
68.00	2.60E-2	1.16E-4	1.78E-2	2.47E-4	1.92E-1	2.86E-4	7.63E-3	2.75E-3	-3.6507	0.2254
71.00	1.04E-2	3.17E-5	1.73E-2	1.76E-4	1.84E-1	2.41E-4	5.69E-3	1.97E-3	-4.5621	0.1944
74.00	4.29E-3	1.92E-5	1.70E-2	2.98E-4	1.76E-1	2.79E-4	5.84E-3	2.90E-3	-5.4513	0.1970
77.00	1.84E-3	9.39E-6	1.64E-2	3.98E-4	1.69E-1	2.56E-4	6.56E-3	3.32E-3	-6.2977	0.2088
80.00	8.35E-4	3.66E-6	1.62E-2	4.06E-4	1.64E-1	1.77E-4	7.53E-3	2.81E-3	-7.0877	0.2238
83.00	3.94E-4	2.79E-6	1.58E-2	7.49E-4	1.57E-1	2.01E-4	7.30E-3	4.07E-3	-7.8401	0.2203
86.00	1.92E-4	1.99E-6	1.38E-2	1.11E-3	1.53E-1	1.39E-4	1.31E-2	4.10E-3	-8.5568	0.2966

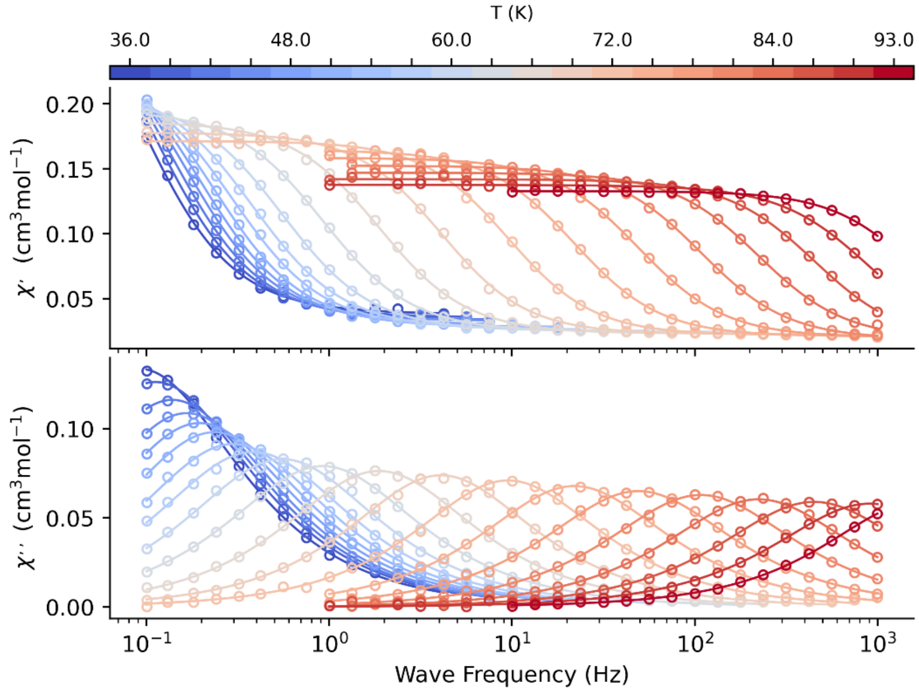

Figure S85. In-phase (top) and out-of-phase (bottom) ac susceptibilities of **3-Dy** in a zero field. Solid lines are fits to the generalized Debye model in CC-FIT2,[22,23] giving $0.0324 \leq \alpha \leq 0.169$.

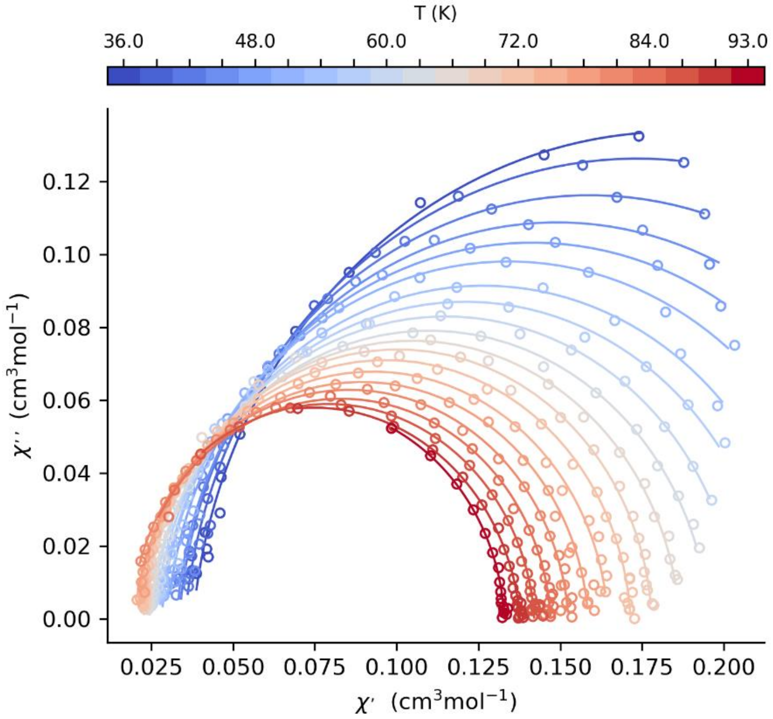

Figure S86. Cole-Cole plot showing fitting of ac data for **3-Dy** in a zero dc field. Solid lines are fits to the generalized Debye model in CC-FIT2,[22,23] giving $0.0323 \leq \alpha \leq 0.169$.

Table S15. Best fit parameters to the generalized Debye model for **3-Dy** in zero dc field.

T	τ_{Debye}	$\tau_{\mathrm{Debye}}^{\mathrm{err}}$	χ_S	χ_S^{err}	χ_T	χ_T^{err}	α	α^{err}	$\langle \ln \tau \rangle$	$\sigma_{\langle \ln \tau \rangle}$
(K)	(s)		(emu/mol)		(emu/mol)				ln (s)	
36.00	2.29	2.57E-1	2.78E-2	9.69E-4	3.93E-1	2.39E-2	1.69E-1	2.06E-2	0.5468	0.6670
39.00	1.64	1.19E-1	2.71E-2	8.74E-4	3.43E-1	1.40E-2	1.36E-1	1.76E-2	0.3514	0.6451
42.00	1.25	6.59E-2	2.65E-2	8.16E-4	3.03E-1	9.25E-3	1.12E-1	1.60E-2	0.1402	0.5854
45.00	1.01	4.20E-2	2.60E-2	7.62E-4	2.79E-1	6.75E-3	1.00E-1	1.46E-2	-0.0421	0.5459
48.00	8.37E-1	2.60E-2	2.48E-2	6.54E-4	2.61E-1	4.74E-3	9.18E-2	1.22E-2	-0.2061	0.5909
51.00	6.87E-1	1.66E-2	2.43E-2	5.85E-4	2.45E-1	3.47E-3	8.03E-2	1.06E-2	-0.3880	0.5984
54.00	5.39E-1	1.07E-2	2.41E-2	5.33E-4	2.29E-1	2.58E-3	7.73E-2	9.51E-3	-0.6212	0.6077
57.00	4.16E-1	6.33E-3	2.39E-2	4.54E-4	2.18E-1	1.80E-3	7.26E-2	7.82E-3	-0.8764	0.6350
60.00	2.87E-1	3.33E-3	2.37E-2	3.91E-4	2.06E-1	1.21E-3	6.08E-2	6.43E-3	-1.2451	0.6028
63.00	1.72E-1	1.46E-3	2.37E-2	3.16E-4	1.96E-1	7.45E-4	5.56E-2	4.88E-3	-1.7574	0.6003
66.00	8.49E-2	6.07E-4	2.29E-2	2.96E-4	1.87E-1	5.15E-4	4.51E-2	4.21E-3	-2.4656	0.5536
69.00	3.89E-2	2.25E-4	2.26E-2	2.61E-4	1.78E-1	3.45E-4	3.23E-2	3.46E-3	-3.2464	0.4662
72.00	1.67E-2	9.40E-5	2.18E-2	2.76E-4	1.71E-1	2.80E-4	3.42E-2	3.34E-3	-4.0903	0.4803
75.00	7.26E-3	4.08E-5	2.09E-2	2.90E-4	1.65E-1	3.41E-4	3.66E-2	3.50E-3	-4.9253	0.5047
78.00	3.24E-3	2.38E-5	2.01E-2	4.35E-4	1.59E-1	3.57E-4	4.03E-2	4.55E-3	-5.7329	0.5314
81.00	1.47E-3	8.48E-6	1.87E-2	3.99E-4	1.53E-1	2.36E-4	4.00E-2	3.58E-3	-6.5208	0.5291
84.00	7.12E-4	5.48E-6	1.90E-2	6.18E-4	1.47E-1	2.37E-4	3.76E-2	4.60E-3	-7.2471	0.5118
87.00	3.53E-4	4.98E-6	1.63E-2	1.26E-3	1.42E-1	2.73E-4	4.12E-2	7.07E-3	-7.9483	0.5374
90.00	1.75E-4	3.10E-6	1.16E-2	1.60E-3	1.38E-1	1.65E-4	5.38E-2	5.95E-3	-8.6489	0.6201
93.00	9.50E-5	2.90E-6	9.42E-3	2.81E-3	1.33E-1	1.41E-4	3.24E-2	6.97E-3	-9.2611	0.4734

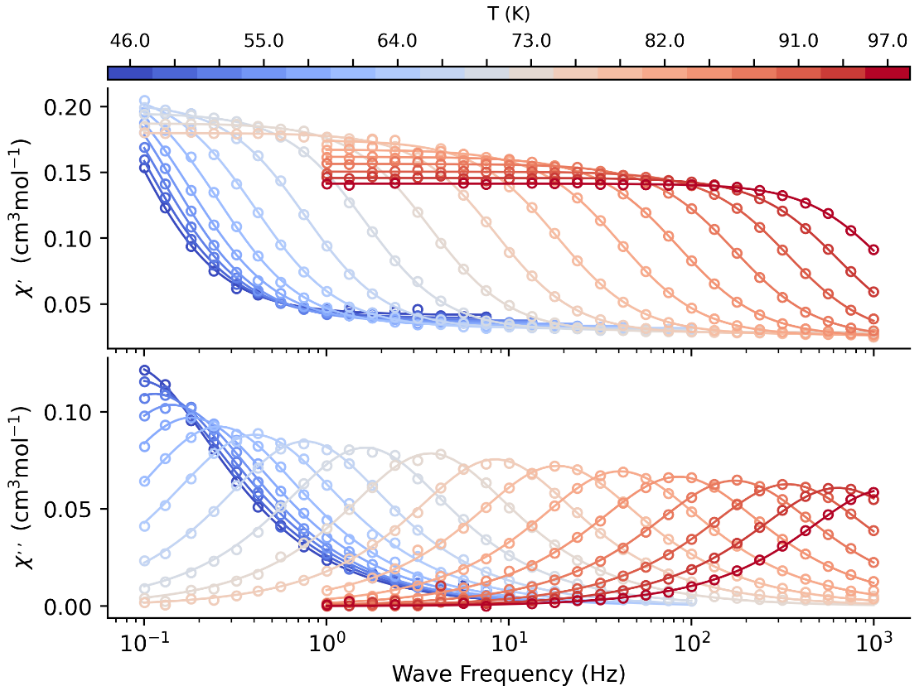

Figure S87. In-phase (top) and out-of-phase (bottom) ac susceptibilities of **4-Dy** in a zero field. Solid lines are fits to the generalized Debye model in CC-FIT2,[22,23] giving 0.00417 ≤ α ≤ 0.0545.

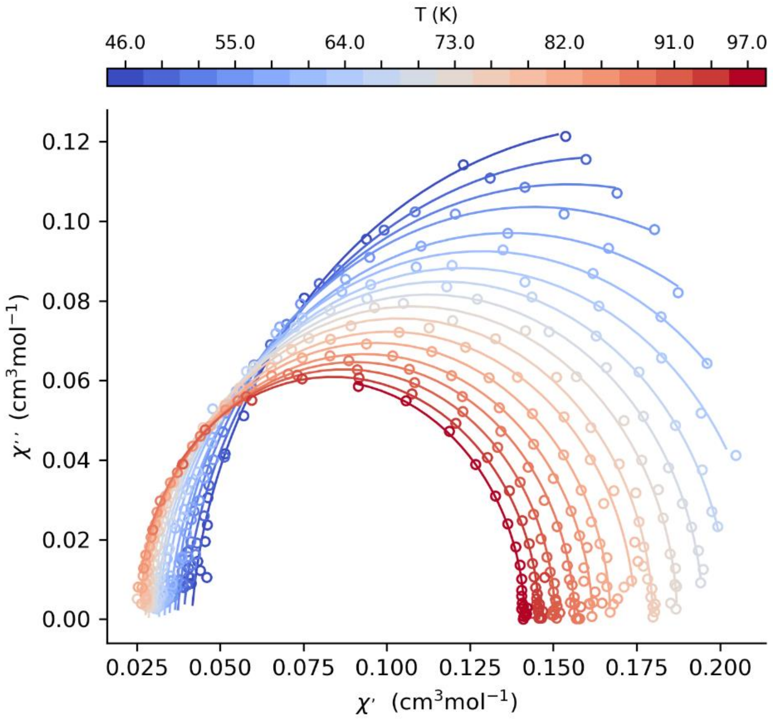

Figure S88. Cole-Cole plot showing fitting of ac data for **4-Dy** at in a zero dc field. Solid lines are fits to the generalized Debye model in CC-FIT2,[22,23] giving 0.00417 ≤ α ≤ 0.0545.

Table S16. Best fit parameters to the generalized Debye model for **4-Dy** in zero dc field.

T	τ_{Debye}	$\tau_{\mathrm{Debye}}^{\mathrm{err}}$	χ_{S}	$\chi_{\mathrm{S}}^{\mathrm{err}}$	χ_{T}	$\chi_{\mathrm{T}}^{\mathrm{err}}$	α	α^{err}	$\langle \ln \tau \rangle$	$\sigma_{\ln \tau}$
(K)	(s)		(emu/mol)		(emu/mol)				ln (s)	
46.00	1.95	8.74E–02	4.15E–02	7.68E–04	3.09E–01	8.67E–03	4.93E–02	1.30E–02	0.6696	0.5919
49.00	1.69	4.64E–02	3.90E–02	5.44E–04	2.92E–01	4.97E–03	5.45E–02	8.76E–03	0.5271	0.6245
52.00	1.39	3.62E–02	3.72E–02	5.81E–04	2.72E–01	4.46E–03	4.47E–02	9.54E–03	0.3291	0.5613
55.00	1.12	1.86E–02	3.64E–02	4.73E–04	2.52E–01	2.72E–03	2.53E–02	7.33E–03	0.1125	0.4162
58.00	8.83E–01	1.13E–02	3.48E–02	4.02E–04	2.39E–01	1.93E–03	3.19E–02	6.21E–03	-0.1246	0.4697
61.00	6.22E–01	5.82E–03	3.34E–02	3.54E–04	2.24E–01	1.29E–03	1.87E–02	5.28E–03	-0.4753	0.3560
64.00	3.93E–01	3.39E–03	3.17E–02	3.63E–04	2.14E–01	1.04E–03	2.13E–02	5.23E–03	-0.9347	0.3808
67.00	2.09E–01	1.09E–03	3.11E–02	2.66E–04	2.03E–01	5.10E–04	7.12E–03	3.39E–03	-1.5674	0.2176
70.00	9.76E–02	4.61E–04	3.00E–02	2.75E–04	1.95E–01	3.65E–04	7.05E–03	3.07E–03	-2.3269	0.2165
73.00	4.30E–02	2.69E–04	2.86E–02	2.96E–04	1.87E–01	4.00E–04	5.23E–03	3.89E–03	-3.1461	0.1862
76.00	1.89E–02	1.06E–04	2.77E–02	2.87E–04	1.80E–01	3.02E–04	4.17E–03	3.49E–03	-3.9698	0.1661
79.00	8.62E–03	6.76E–05	2.67E–02	4.14E–04	1.75E–01	5.23E–04	1.50E–02	5.04E–03	-4.7533	0.3173
82.00	3.91E–03	1.92E–05	2.59E–02	2.94E–04	1.67E–01	2.64E–04	1.10E–02	3.17E–03	-5.5438	0.2716
85.00	1.87E–03	1.12E–05	2.50E–02	4.12E–04	1.62E–01	2.65E–04	1.70E–02	3.85E–03	-6.2835	0.3390
88.00	9.20E–04	5.26E–06	2.45E–02	4.57E–04	1.56E–01	2.03E–04	1.33E–02	3.62E–03	-6.9907	0.2983
91.00	4.70E–04	2.60E–06	2.37E–02	5.07E–04	1.51E–01	1.47E–04	6.22E–03	3.29E–03	-7.6627	0.2032
94.00	2.49E–04	6.99E–06	2.21E–02	2.68E–03	1.46E–01	4.04E–04	1.01E–02	1.28E–02	-8.2981	0.2593
97.00	1.35E–04	3.32E–06	1.97E–02	2.34E–03	1.41E–01	1.74E–04	8.13E–03	7.57E–03	-8.9139	0.2327

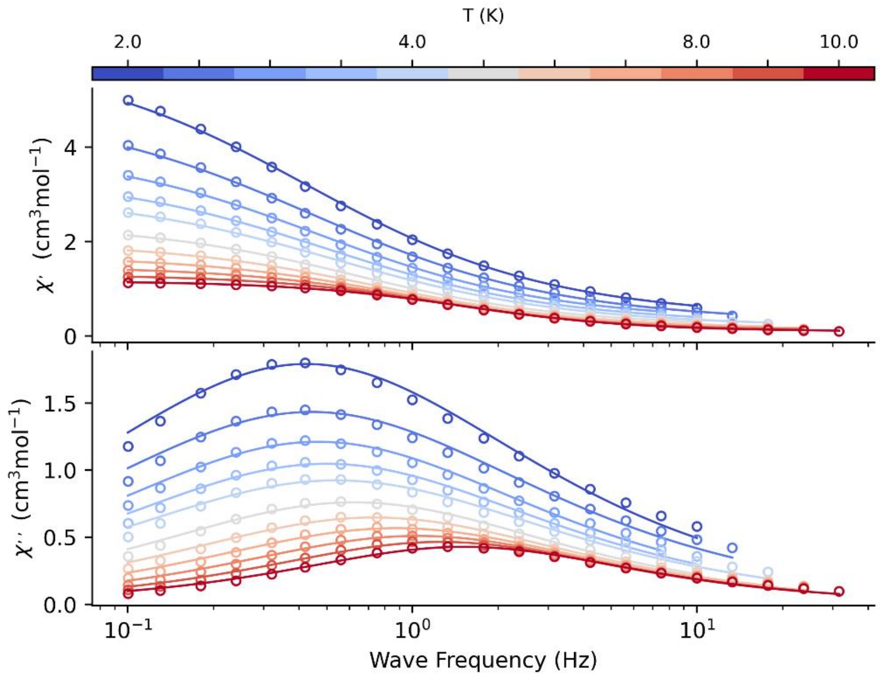

Figure S89. In-phase (top) and out-of-phase (bottom) ac susceptibilities of **5-Dy** in a zero field between 2 and 10 K. Solid lines are fits to the generalized Debye model in CC-FIT2,[22,23] giving $0.147 \leq \alpha \leq 0.297$.

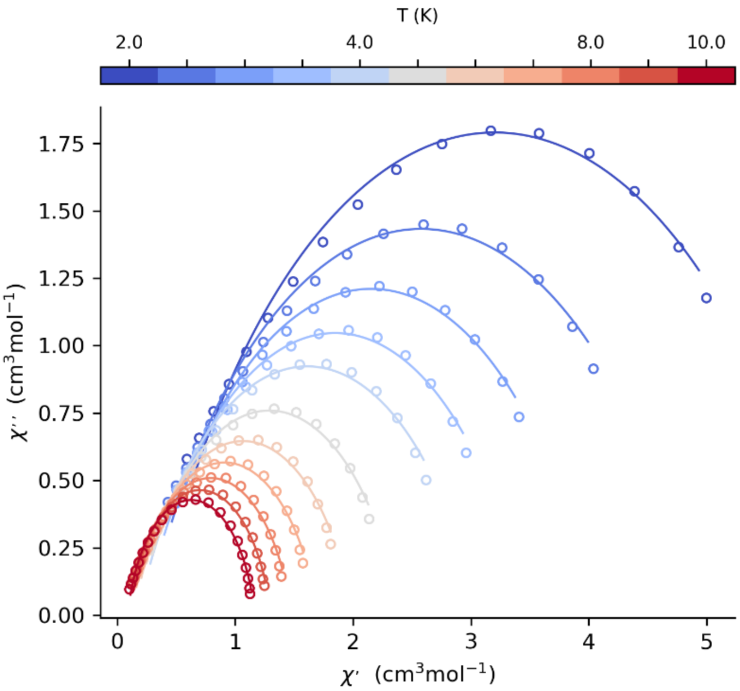

Figure S90. Cole-Cole plot showing fitting of ac data for **5-Dy** in a zero dc field between 2 and 10 K. Solid lines are fits to the generalized Debye model in CC-FIT2,[22,23] giving $0.18 \leq \alpha \leq 0.349$.

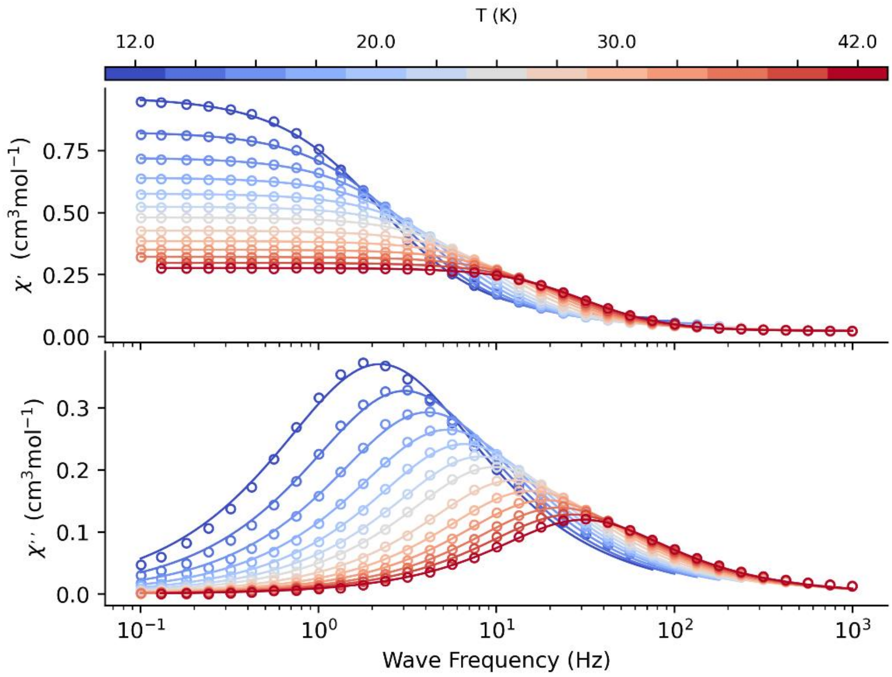

Figure S91. In-phase (top) and out-of-phase (bottom) ac susceptibilities of **5-Dy** in a zero field between 12 and 42 K. Solid lines are fits to the generalized Debye model in CC-FIT2,[22,23] giving 0.0374 ≤ α ≤ 0.125.

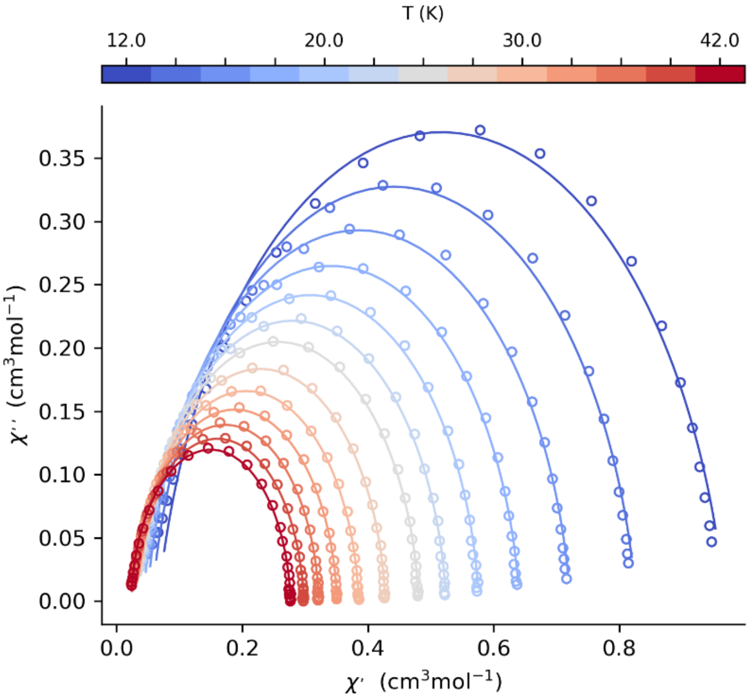

Figure S92. Cole-Cole plot showing fitting of ac data for **5-Dy** in a zero dc field between 12 and 42 K. Solid lines are fits to the generalized Debye model in CC-FIT2,[22,23] giving 0.0373 ≤ α ≤ 0.297.

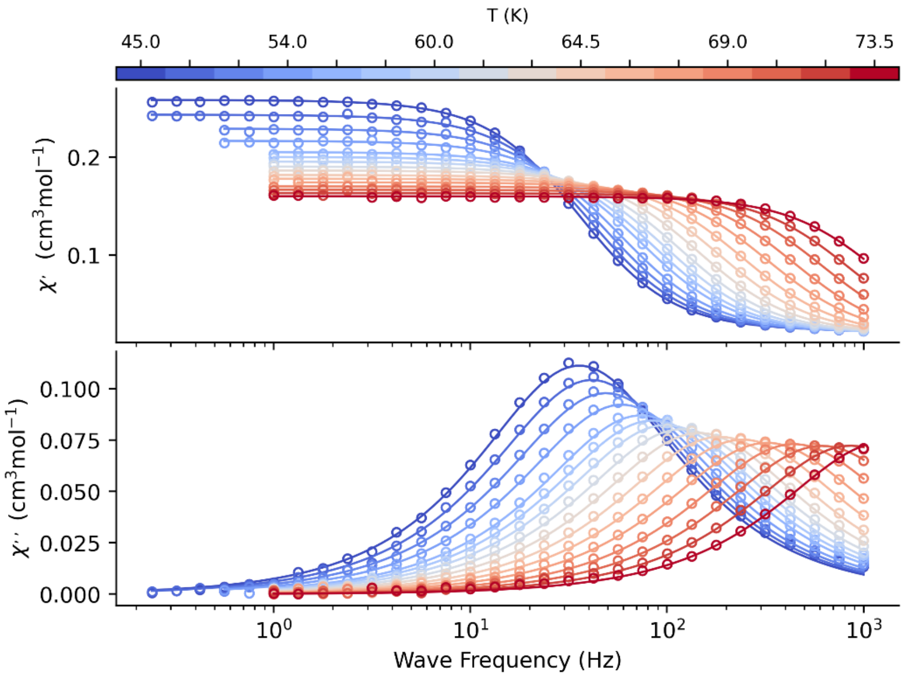

Figure S93. In-phase (top) and out-of-phase (bottom) ac susceptibilities of **5-Dy** in a zero field between 45 and 73.5 K. Solid lines are fits to the generalized Debye model in CC-FIT2,[22,23] giving $0.0373 \leq \alpha \leq 0.297$.

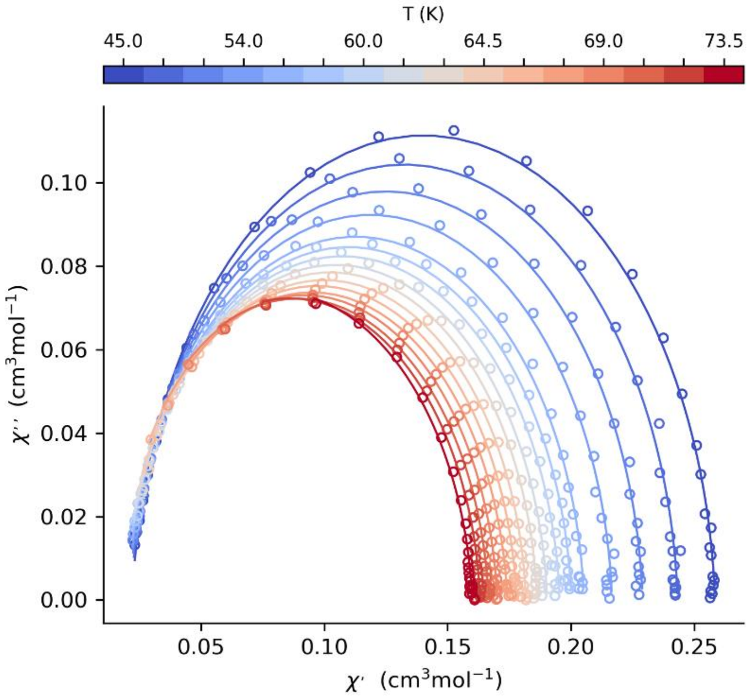

Figure S94. Cole-Cole plot showing fitting of ac data for **5-Dy** in a zero dc field between 45 and 73.5 K. Solid lines are fits to the generalized Debye model in CC-FIT2,[22,23] giving 0.0373 $\leq \alpha \leq 0.297$.

Table S17. Best fit parameters to the generalized Debye model for **5-Dy** in zero dc field.

T	τ_{Debye}	$\tau_{\text{Debye}}^{\text{err}}$	χ_S	χ_S^{err}	χ_T	χ_T^{err}	α	α^{err}	$\langle \ln \tau \rangle$	$\sigma_{\ln \tau}$
(K)	(s)		(emu/mol)		(emu/mol)				ln (s)	
2.00	3.75E-1	9.72E-3	3.35E-1	3.44E-2	6.07	7.02E-2	2.89E-1	1.05E-2	-0.9808	1.7956
2.50	3.59E-1	1.02E-2	2.53E-1	2.82E-2	4.90	6.07E-2	2.97E-1	1.10E-2	-1.0233	1.8340
3.00	3.41E-1	8.29E-3	2.77E-1	2.61E-2	4.03	4.44E-2	2.71E-1	1.11E-2	-1.0764	1.7013
3.50	3.19E-1	8.11E-3	2.28E-1	2.22E-2	3.47	3.93E-2	2.69E-1	1.13E-2	-1.1441	1.6943
4.00	2.95E-1	8.09E-3	1.77E-1	1.82E-2	3.05	3.61E-2	2.72E-1	1.14E-2	-1.2207	1.7101
5.00	2.55E-1	5.75E-3	1.82E-1	1.62E-2	2.40	2.37E-2	2.35E-1	1.13E-2	-1.3663	1.5249
6.00	2.16E-1	4.83E-3	1.40E-1	1.23E-2	2.00	1.90E-2	2.26E-1	1.09E-2	-1.5330	1.4839
7.00	1.81E-1	3.78E-3	1.18E-1	9.95E-3	1.70	1.47E-2	2.09E-1	1.04E-2	-1.7066	1.4045
8.00	1.52E-1	2.88E-3	1.09E-1	8.65E-3	1.48	1.12E-2	1.86E-1	1.00E-2	-1.8839	1.2942
9.00	1.26E-1	2.10E-3	9.96E-2	7.33E-3	1.30	8.36E-3	1.64E-1	9.15E-3	-2.0690	1.1909
10.00	1.05E-1	1.61E-3	8.77E-2	6.09E-3	1.17	6.70E-3	1.47E-1	8.55E-3	-2.2578	1.1115
12.00	7.21E-2	8.91E-4	6.62E-2	3.80E-3	9.69E-1	4.25E-3	1.25E-1	6.85E-3	-2.6291	1.0025
14.00	5.22E-2	5.33E-4	5.64E-2	2.80E-3	8.27E-1	2.86E-3	1.04E-1	5.79E-3	-2.9524	0.8969
16.00	3.89E-2	3.29E-4	4.86E-2	2.08E-3	7.22E-1	1.98E-3	8.87E-2	4.86E-3	-3.2480	0.8194
18.00	3.00E-2	2.15E-4	4.28E-2	1.59E-3	6.41E-1	1.44E-3	7.79E-2	4.14E-3	-3.5054	0.7609
20.00	2.41E-2	1.60E-4	3.94E-2	1.41E-3	5.77E-1	1.17E-3	6.73E-2	3.91E-3	-3.7274	0.7013
22.00	1.96E-2	1.20E-4	3.57E-2	1.16E-3	5.25E-1	9.51E-4	6.26E-2	3.59E-3	-3.9319	0.6736
24.00	1.63E-2	8.97E-5	3.35E-2	1.00E-3	4.81E-1	7.62E-4	5.63E-2	3.26E-3	-4.1155	0.6360
27.00	1.27E-2	6.62E-5	3.02E-2	8.43E-4	4.28E-1	6.25E-4	5.09E-2	3.09E-3	-4.3640	0.6017

30.00	1.02E-2	5.06E-5	2.78E-2	7.16E-4	3.85E-1	5.24E-4	4.74E-2	2.94E-3	-4.5830	0.5792
33.00	8.44E-3	4.29E-5	2.58E-2	6.59E-4	3.51E-1	4.80E-4	4.55E-2	3.01E-3	-4.7744	0.5669
36.00	7.07E-3	3.93E-5	2.43E-2	6.16E-4	3.22E-1	4.78E-4	4.23E-2	3.27E-3	-4.9523	0.5448
39.00	6.03E-3	3.27E-5	2.31E-2	5.70E-4	2.98E-1	4.35E-4	4.38E-2	3.20E-3	-5.1108	0.5556
42.00	5.17E-3	2.89E-5	2.30E-2	5.63E-4	2.77E-1	4.09E-4	3.74E-2	3.34E-3	-5.2654	0.5104
45.00	4.44E-3	2.44E-5	2.22E-2	5.28E-4	2.58E-1	3.92E-4	3.73E-2	3.31E-3	-5.4163	0.5099
48.00	3.83E-3	2.34E-5	2.17E-2	5.67E-4	2.43E-1	3.98E-4	3.80E-2	3.68E-3	-5.5643	0.5151
51.00	3.25E-3	1.77E-5	2.12E-2	4.89E-4	2.29E-1	3.65E-4	3.80E-2	3.35E-3	-5.7292	0.5151
54.00	2.70E-3	1.73E-5	2.07E-2	5.62E-4	2.17E-1	3.88E-4	3.81E-2	3.91E-3	-5.9134	0.5152
57.00	2.16E-3	1.52E-5	2.02E-2	6.15E-4	2.05E-1	4.19E-4	3.88E-2	4.37E-3	-6.1379	0.5209
58.50	1.88E-3	1.13E-5	1.95E-2	5.27E-4	2.00E-1	3.36E-4	4.22E-2	3.70E-3	-6.2744	0.5445
60.00	1.61E-3	1.02E-5	1.95E-2	5.62E-4	1.95E-1	3.31E-4	4.20E-2	3.89E-3	-6.4299	0.5432
61.50	1.34E-3	1.07E-5	1.91E-2	7.23E-4	1.90E-1	3.88E-4	4.09E-2	4.89E-3	-6.6129	0.5354
63.00	1.09E-3	6.61E-6	1.87E-2	5.69E-4	1.86E-1	2.72E-4	4.14E-2	3.70E-3	-6.8202	0.5389
64.50	8.61E-4	7.35E-6	1.82E-2	8.32E-4	1.82E-1	3.46E-4	4.10E-2	5.13E-3	-7.0579	0.5363
66.00	6.56E-4	5.07E-6	1.63E-2	7.90E-4	1.78E-1	2.73E-4	4.55E-2	4.47E-3	-7.3298	0.5670
67.50	4.93E-4	4.92E-6	1.56E-2	1.06E-3	1.74E-1	3.06E-4	4.68E-2	5.48E-3	-7.6150	0.5755
69.00	3.60E-4	3.58E-6	1.38E-2	1.10E-3	1.70E-1	2.42E-4	4.28E-2	5.00E-3	-7.9286	0.5482
70.50	2.59E-4	4.11E-6	1.17E-2	1.77E-3	1.67E-1	2.69E-4	4.46E-2	6.80E-3	-8.2569	0.5606
72.00	1.86E-4	4.10E-6	8.65E-3	2.50E-3	1.63E-1	2.71E-4	4.21E-2	7.89E-3	-8.5915	0.5437
73.50	1.27E-4	4.71E-6	1.88E-4	4.29E-3	1.60E-1	2.70E-4	5.02E-2	9.94E-3	-8.9744	0.5974

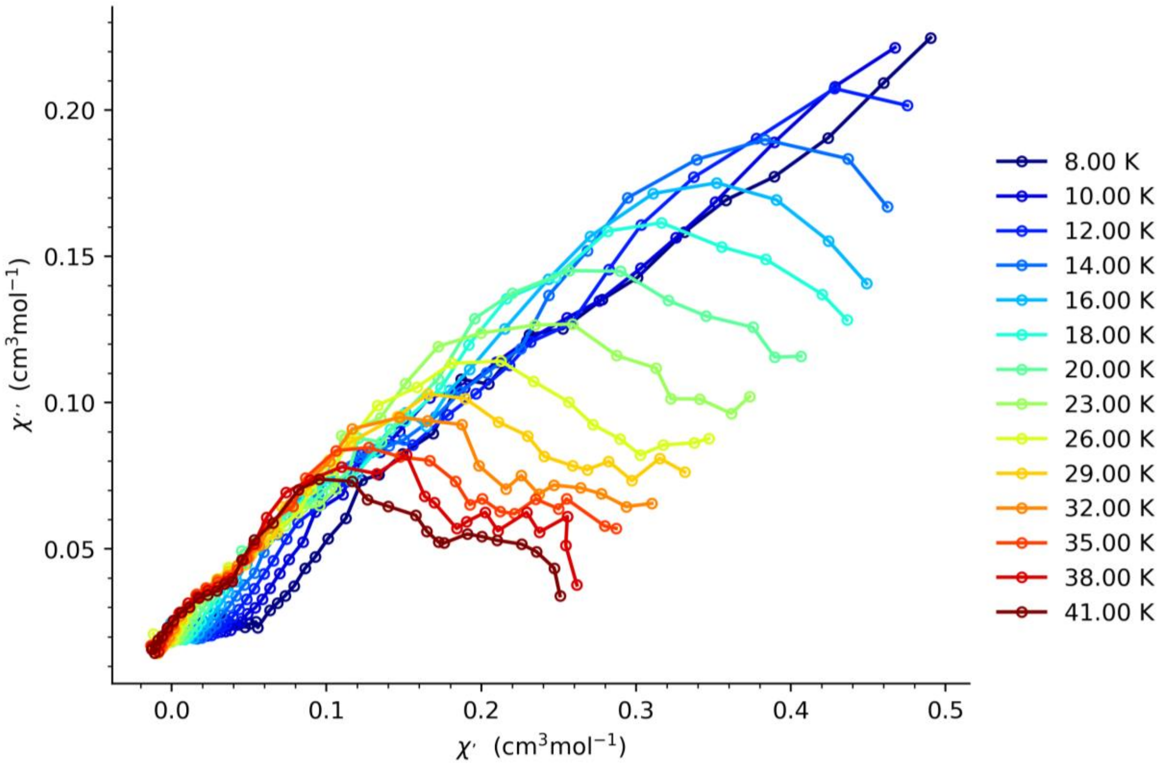

Figure S95. Cole-Cole plot showing raw ac data for **5%Dy@2-Y** in a zero dc field, 8–41 K.

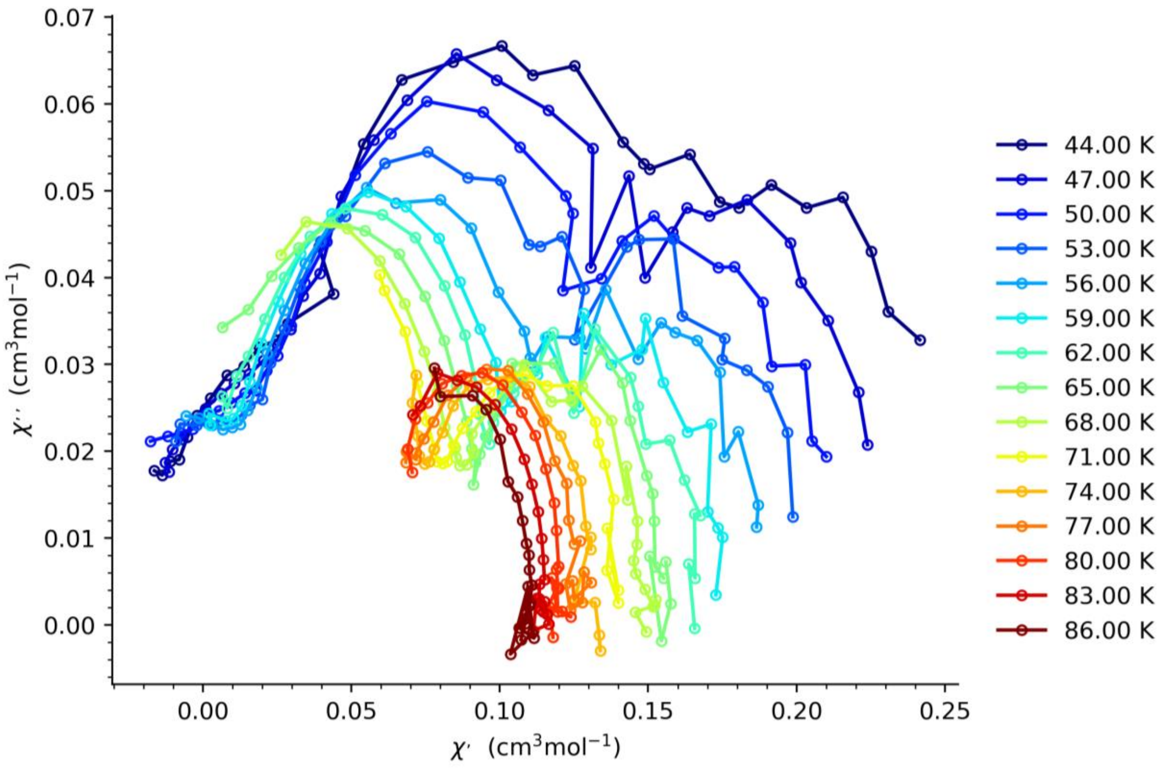

Figure S96. Cole-Cole plot showing raw ac data for **5%Dy@2-Y** in a zero dc field, 44–86 K.

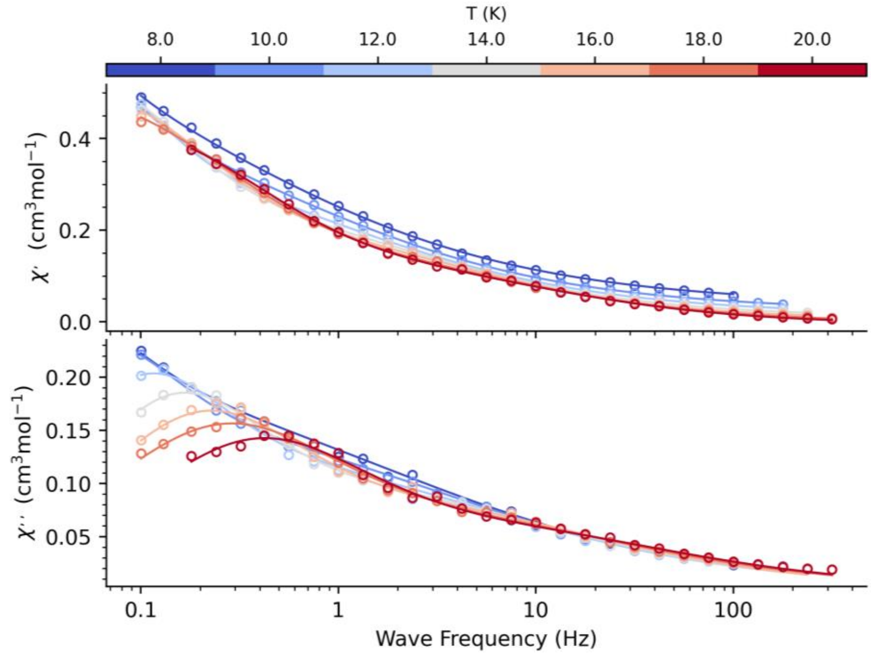

Figure S97. In-phase (top) and out-of-phase (bottom) ac susceptibilities of **5%Dy@2-Y** in a zero field, between 8 and 20 K. Solid lines are fits of peaks B and C to the double generalized Debye model in CC-FIT2, with χ_{total} fitted freely (negative values allowed).[22,23]

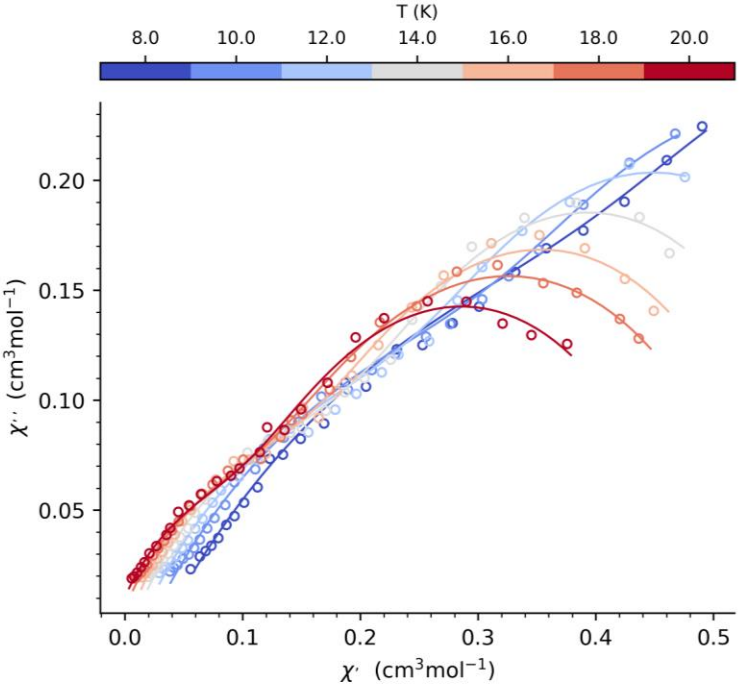

Figure S98. Cole-Cole plot showing fitting of ac data for **5%Dy@2-Y** in a zero dc field, between 8 and 20 K. Solid lines are fits of peaks B and C to the double generalized Debye model in CC-FIT2, with χ_{total} fitted freely (negative values allowed).[22,23]

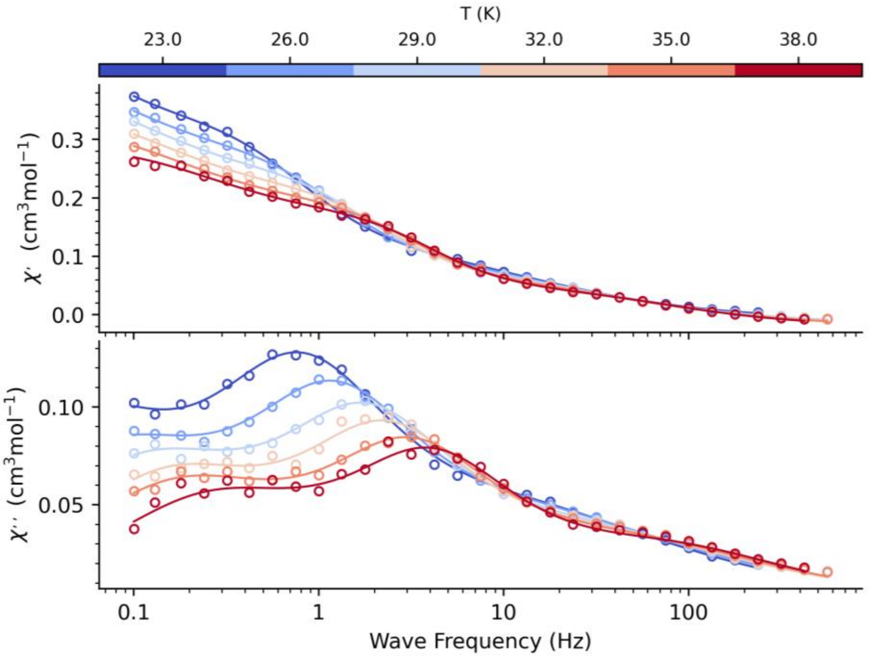

Figure S99. In-phase (top) and out-of-phase (bottom) ac susceptibilities of **5%Dy@2-Y** in a zero field, between 23 and 38 K. Solid lines are fits of peaks A, B and C to the triple generalized Debye model in CC-FIT2, with χ_{total} fitted freely (negative values allowed).[22,23]

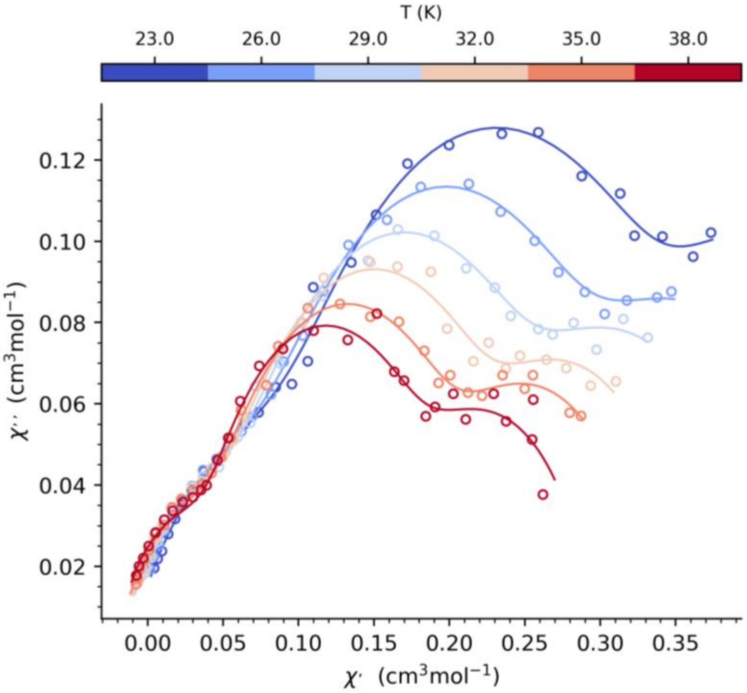

Figure S100. Cole-Cole plot showing fitting of ac data for **5%Dy@2-Y** in a zero dc field, between 23 and 38 K. Solid lines are fits of peaks A, B and C to the triple generalized Debye model in CC-FIT2, with χ_{total} fitted freely (negative values allowed).[22,23]

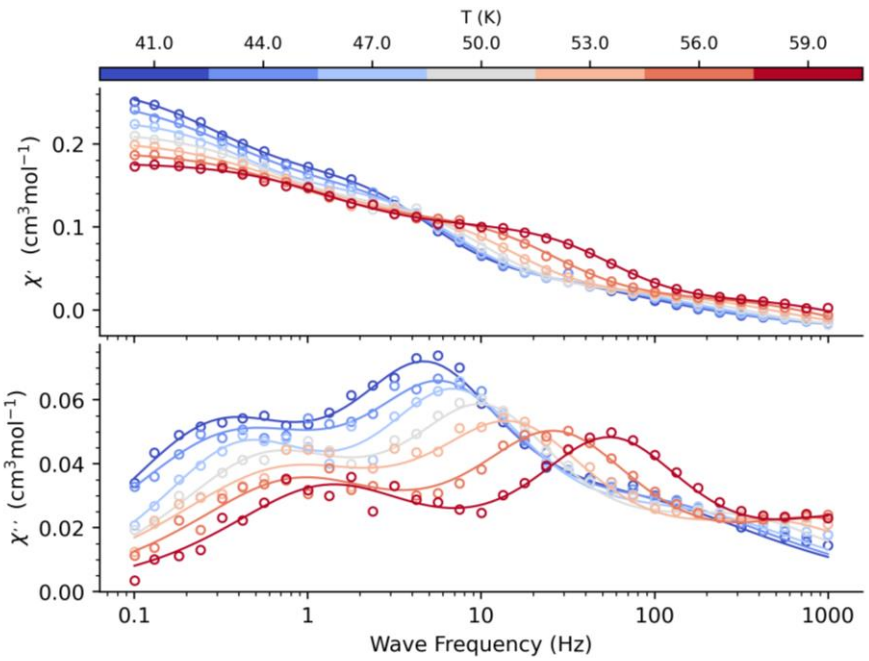

Figure S101. In-phase (top) and out-of-phase (bottom) ac susceptibilities of **5%Dy@2-Y** in a zero field, between 41 and 59 K. Solid lines are fits of peaks A, B and C to the triple generalized Debye model in CC-FIT2, with χ_{total} restrained by the Curie law.[22,23]

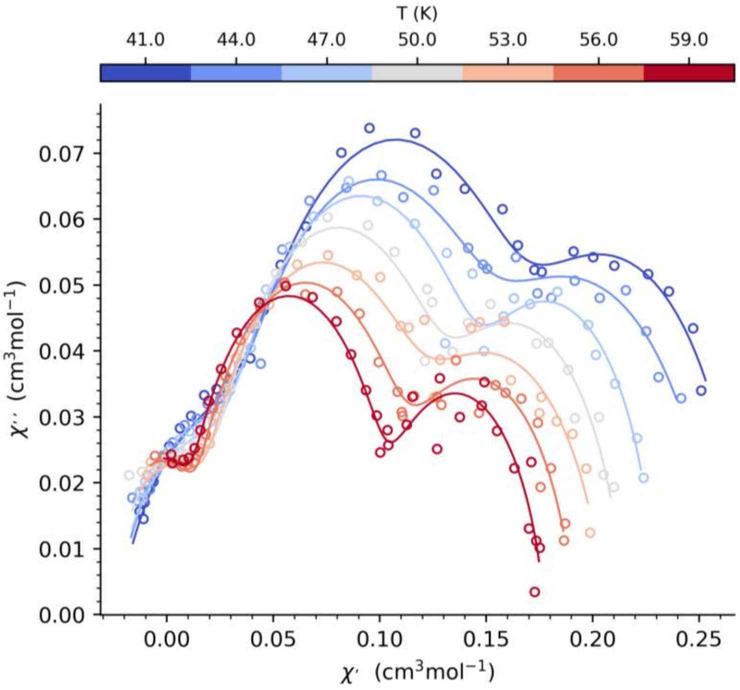

Figure S102. Cole-Cole plot showing fitting of ac data for **5%Dy@2-Y** in a zero dc field, between 41 and 59 K. Solid lines are fits of peaks A, B and C to the triple generalized Debye model in CC-FIT2, with χ_{total} restrained by the Curie Law.[22,23]

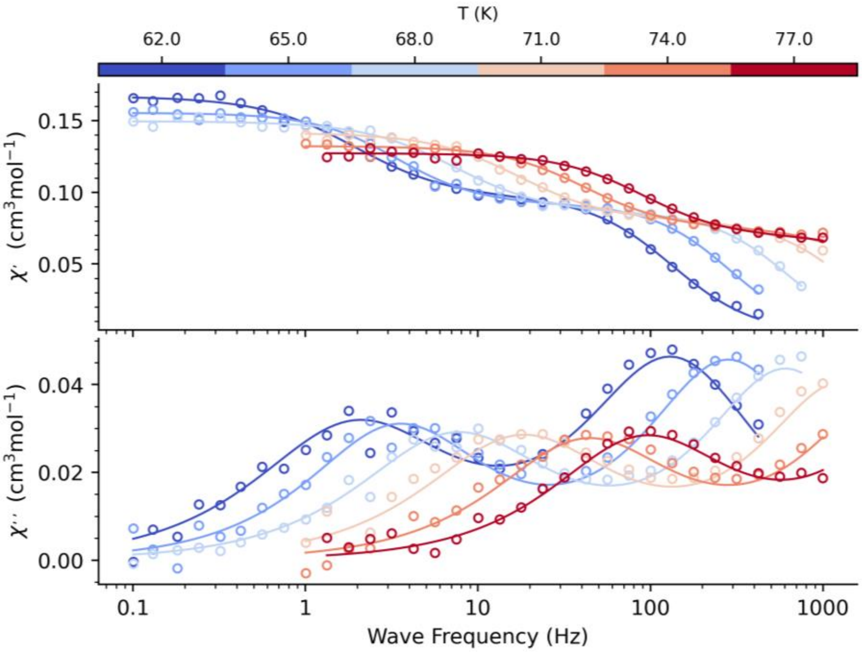

Figure S103. In-phase (top) and out-of-phase (bottom) ac susceptibilities of **5%Dy@2-Y** in a zero field, between 62 and 77 K. Solid lines are fits of peaks A and B to the double generalized Debye model in CC-FIT2, with χ_{total} restrained to be positive.[22,23]

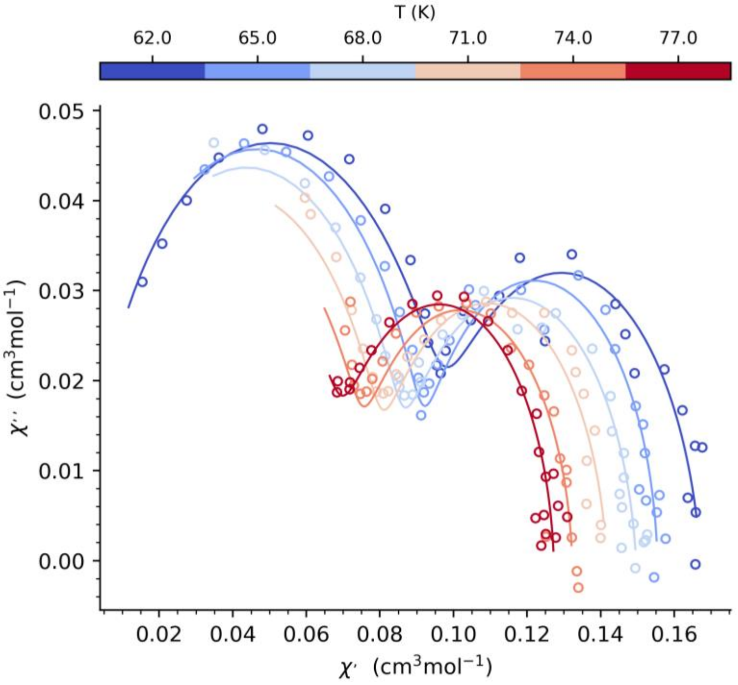

Figure S104. Cole-Cole plot showing fitting of ac data for **5%Dy@2-Y** in a zero dc field, between 62 and 77 K. Solid lines are fits of peaks A and B to the double generalized Debye model in CC-FIT2, with χ_{total} restrained to be positive.[22,23]

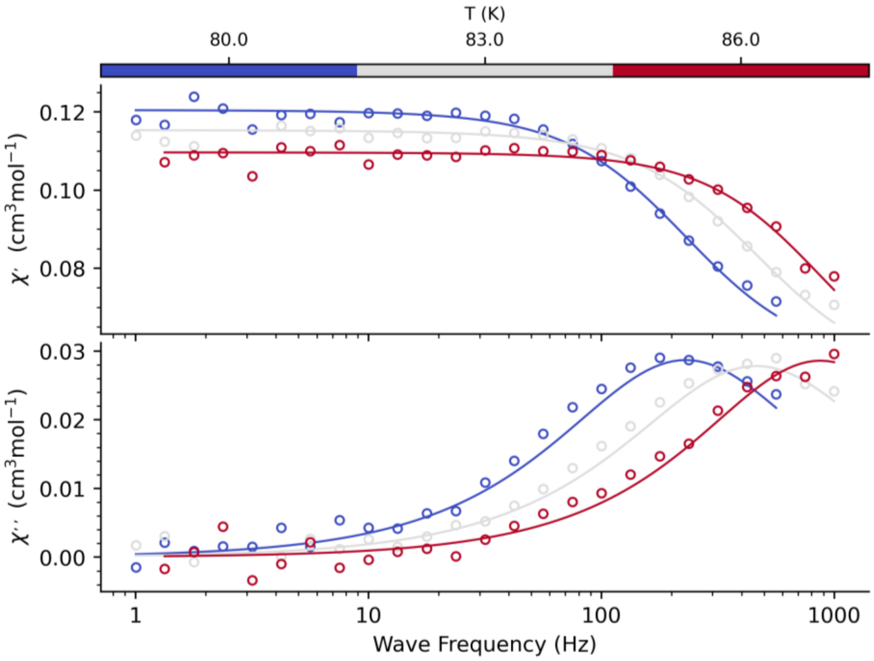

Figure S105. In-phase (top) and out-of-phase (bottom) ac susceptibilities of **5%Dy@2-Y** in a zero field, between 80 and 86 K. Solid lines are fits of peak A to the generalized Debye model in CC-FIT2, with χ_S restrained to be positive.[22,23]

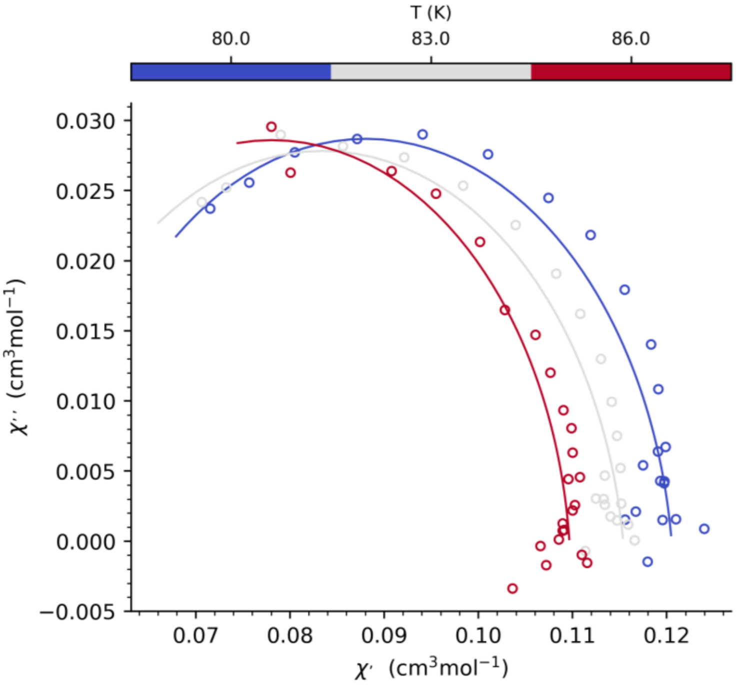

Figure S106. Cole-Cole plot showing fitting of ac data for **5%Dy@2-Y** in a zero dc field, between 80 and 86 K. Solid lines are fits of peak A to the generalized Debye model in CC-FIT2, with χ_S restrained to be positive.[22,23]

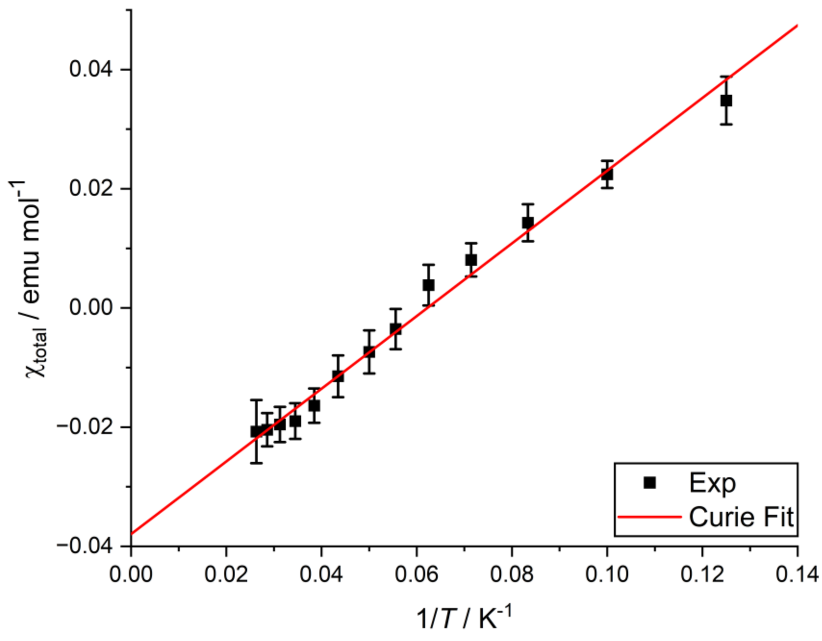

Figure S107. Curie fit of χ_{total} for **5%Dy@2-Y** between 8–38 K.

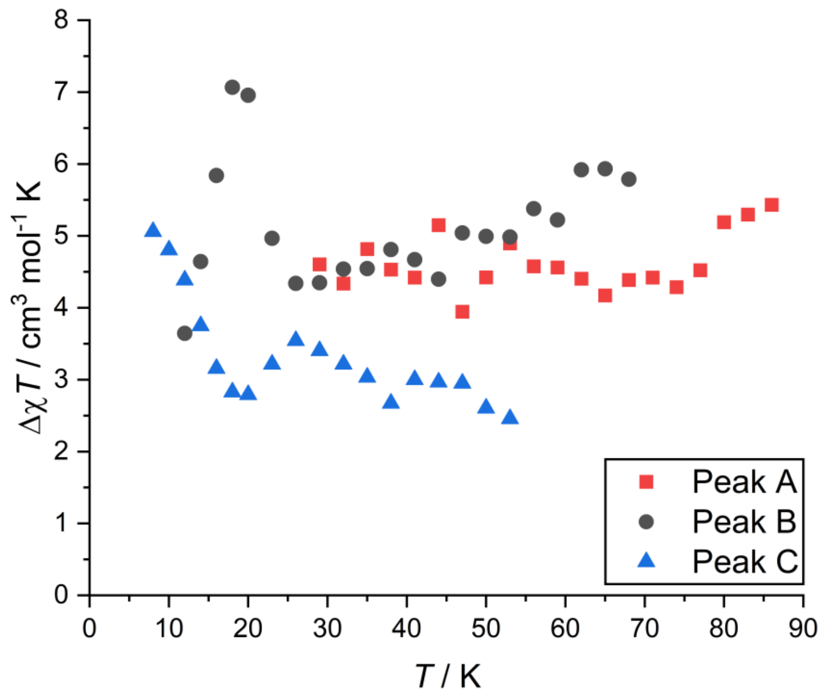

Figure S108. Temperature-dependence of the contribution of the three peaks A, B and C to the out-of-phase susceptibility of **5%Dy@2-Y**.

Table S18. Best fit parameters to the generalized Debye model for peaks B and C in **5%Dy@2-Y** in zero dc field, between 8–20 K.

T	τ_B	τ_B^{err}	α_B	α_B^{err}	$\Delta\chi_B$	$\Delta\chi_B^{err}$	τ_C	τ_C^{err}	α_C	α_C^{err}	$\Delta\chi_C$	$\Delta\chi_C^{err}$	$\Delta\chi_{total}$	$\Delta\chi_{total}^{err}$	$\langle \ln \tau_B \rangle$	$\sigma_{\langle \ln \tau_B \rangle}$	$\langle \ln \tau_C \rangle$	$\sigma_{\langle \ln \tau_C \rangle}$
(K)	(s)				(emu/mol)						(emu/mol)		(emu/mol)		ln (s)		ln (s)	
8.00	4.57	2.61	1.24E-2	2.53E-1	3.35E-1	2.70E-1	4.94E-1	3.25E-1	5.00E-1	2.67E-2	6.32E-1	1.64E-1	3.48E-2	4.01E-3	1.52[a]	0.29[a]	-0.71	3.14
10.00	2.76	3.29E-1	1.39E-9	8.03E-2	3.23E-1	7.63E-2	2.48E-1	7.31E-2	4.69E-1	1.79E-2	4.80E-1	5.71E-2	2.24E-2	2.27E-3	1.02[a]	0.00[a]	-1.39	2.90
12.00	1.56	6.69E-2	1.20E-17	5.37E-2	3.04E-1	5.00E-2	1.28E-1	4.09E-2	4.45E-1	2.73E-2	3.65E-1	4.85E-2	1.43E-2	3.11E-3	0.44	0.00	-2.05	2.72
14.00	1.07	2.97E-2	4.82E-2	3.41E-2	3.32E-1	3.21E-2	5.86E-2	1.40E-2	4.05E-1	2.90E-2	2.68E-1	2.98E-2	8.06E-3	2.79E-3	0.06	0.58	-2.84	2.45
16.00	7.79E-1	2.86E-2	1.25E-1	3.28E-2	3.65E-1	3.17E-2	3.01E-2	7.75E-3	3.64E-1	4.20E-2	1.97E-1	2.81E-2	3.83E-3	3.41E-3	-0.25	1.00	-3.50	2.20
18.00	6.11E-1	2.45E-2	1.98E-1	2.74E-2	3.93E-1	3.00E-2	1.85E-2	5.60E-3	3.82E-1	4.83E-2	1.57E-1	2.70E-2	-3.54E-3	3.38E-3	-0.49	1.35	-3.99	2.31
20.00	4.13E-1	1.66E-2	1.79E-1	3.17E-2	3.48E-1	2.93E-2	1.27E-2	3.92E-3	3.68E-1	5.50E-2	1.40E-1	2.57E-2	-7.37E-3	3.61E-3	-0.88	1.26	-4.36	2.22

[a] Relaxation times are unreliable as they lie outside the observable frequency range

Table S19. Best fit parameters to the generalized Debye model for peaks A, B and C in **5%Dy@2-Y** in zero dc field, between 23–59 K.

T	τ_A	τ_A^{err}	α_A	α_A^{err}	$\Delta\chi_A$	$\Delta\chi_A^{err}$	τ_B	τ_B^{err}	α_B	α_B^{err}	$\Delta\chi_B$	$\Delta\chi_B^{err}$
(K)	(s)				(emu/mol)		(s)				(emu/mol)	
23.00	3.28	1.33	4.92E-2	2.29E-1	1.78E-1	8.96E-2	2.01E-1	1.32E-2	4.50E-2	5.31E-2	2.16E-1	4.38E-2
26.00	2.23	2.92E-1	1.38E-1	1.04E-1	1.74E-1	3.63E-2	1.32E-1	4.93E-3	8.66E-3	3.98E-2	1.67E-1	2.61E-2
29.00	1.45	1.21E-1	1.32E-1	8.69E-2	1.59E-1	2.69E-2	8.93E-2	4.04E-3	2.31E-3	4.99E-2	1.50E-1	2.70E-2
32.00	1.09	7.02E-2	9.91E-2	6.65E-2	1.36E-1	1.76E-2	6.93E-2	2.93E-3	1.12E-22	4.67E-2	1.42E-1	2.18E-2
35.00	9.38E-1	5.58E-2	1.38E-1	5.49E-2	1.38E-1	1.45E-2	5.11E-2	1.99E-3	8.66E-21	4.26E-2	1.30E-1	1.75E-2
38.00	6.35E-1	7.14E-2	1.31E-1	7.98E-2	1.19E-1	1.95E-2	3.92E-2	2.48E-3	8.79E-16	6.55E-2	1.27E-1	2.40E-2
41.00	5.62E-1	3.37E-2	1.03E-1	4.36E-2	1.08E-1	9.24E-3	3.25E-2	1.27E-3	1.78E-3	3.91E-2	1.14E-1	1.21E-2
44.00	4.69E-1	4.60E-2	1.81E-1	5.69E-2	1.17E-1	1.36E-2	2.59E-2	1.42E-3	3.94E-16	5.37E-2	1.00E-1	1.51E-2

47.00	4.03E-1	2.36E-2	2.69E-2	4.32E-2	8.39E-2	6.81E-3	2.26E-2	9.53E-4	3.37E-13	4.04E-2	1.07E-1	9.96E-3
50.00	2.87E-1	2.09E-2	9.62E-2	4.28E-2	8.84E-2	7.30E-3	1.52E-2	6.75E-4	1.02E-15	3.93E-2	9.99E-2	8.59E-3
53.00	2.39E-1	2.63E-2	1.86E-1	5.25E-2	9.24E-2	9.78E-3	1.04E-2	6.02E-4	3.13E-2	4.86E-2	9.40E-2	1.06E-2
56.00	1.99E-1	1.16E-2	1.50E-1	3.24E-2	8.17E-2	4.60E-3	5.85E-3	2.07E-4	3.95E-2	2.96E-2	9.61E-2	5.45E-3
59.00	1.26E-1	7.26E-3	1.32E-1	3.22E-2	7.73E-2	3.78E-3	2.85E-3	1.21E-4	3.75E-3	3.57E-2	8.85E-2	6.02E-3

T	τ_C	τ_C^{err}	α_C	α_C^{err}	$\Delta\chi_C$	$\Delta\chi_C^{err}$	$\Delta\chi_{total}$	$\Delta\chi_{total}^{err}$	$\langle\ln\tau_A\rangle$	$\sigma_{\langle\ln\tau_A\rangle}$	$\langle\ln\tau_B\rangle$	$\sigma_{\langle\ln\tau_B\rangle}$	$\langle\ln\tau_C\rangle$	$\sigma_{\langle\ln\tau_C\rangle}$
(K)	(s)				(emu/mol)		(emu/mol)		ln (s)		ln (s)		ln (s)	
23.00	1.05E-2	3.02E-3	3.53E-1	5.29E-2	1.40E-1	2.38E-2	-1.15E-2	3.50E-3	1.19[a]	0.59[a]	-1.61	0.56	-4.56	2.14
26.00	8.81E-3	2.12E-3	3.70E-1	4.34E-2	1.36E-1	1.85E-2	-1.64E-2	2.87E-3	0.80[a]	1.07[a]	-2.02	0.24	-4.73	2.24
29.00	5.74E-3	1.87E-3	3.69E-1	5.63E-2	1.17E-1	2.07E-2	-1.90E-2	2.99E-3	0.37	1.04	-2.42	0.12	-5.16	2.23
32.00	4.15E-3	1.17E-3	3.32E-1	6.08E-2	1.00E-1	1.73E-2	-1.95E-2	2.95E-3	0.08	0.87	-2.67	0.00	-5.49	2.02
35.00	2.89E-3	6.61E-4	2.98E-1	6.16E-2	8.67E-2	1.37E-2	-2.04E-2	2.79E-3	-0.06	1.07	-2.97	0.00	-5.85	1.84
38.00	1.88E-3	4.89E-4	2.26E-1	1.18E-1	7.03E-2	1.76E-2	-2.07E-2	5.29E-3	-0.45	1.03	-3.24	0.00	-6.28	1.49
41.00	1.70E-3	3.42E-4	2.91E-1	3.22E-2	7.31E-2	7.26E-3	-2.31E-2	n/a	-0.58	0.89	-3.43	0.11	-6.38	1.80
44.00	1.26E-3	2.64E-4	2.57E-1	4.05E-2	6.74E-2	7.33E-3	-2.41E-2	n/a	-0.76	1.27	-3.65	0.00	-6.68	1.63
47.00	8.97E-4	1.72E-4	2.53E-1	4.31E-2	6.28E-2	6.13E-3	-2.50E-2	n/a	-0.91	0.43	-3.79	0.00	-7.02	1.61
50.00	4.94E-4	6.18E-5	1.52E-1	4.62E-2	5.21E-2	3.96E-3	-2.57E-2	n/a	-1.25	0.86	-4.19	0.00	-7.61	1.13
53.00	2.87E-4	4.03E-5	1.30E-1	6.47E-2	4.63E-2	4.26E-3	-2.64E-2	n/a	-1.43	1.29	-4.57	0.46	-8.16	1.03
56.00	1.70E-4	1.33E-5	2.48E-2	5.93E-2	4.07E-2	2.68E-3	-2.71E-2	n/a	-1.62	1.13	-5.14	0.53	-8.68[a]	0.41[a]
59.00	1.13E-4	1.22E-5	3.64E-11	1.07E-1	3.92E-2	3.96E-3	-2.76E-2	n/a	-2.07	1.04	-5.86	0.16	-9.09[a]	0.00[a]

[a] Relaxation times are unreliable as they lie outside the observable frequency range.

Table S20. Best fit parameters to the generalized Debye model for peaks A and B in **5%Dy@2-Y** in zero dc field, between 62–77 K.

T	τ_A	τ_A^{err}	α_A	α_A^{err}	$\Delta\chi_A$	$\Delta\chi_A^{err}$	τ_B	τ_B^{err}	α_B	α_B^{err}	$\Delta\chi_B$	$\Delta\chi_B^{err}$	$\Delta\chi_{total}$	$\Delta\chi_{total}^{err}$	$\langle\ln\tau_A\rangle$	$\sigma_{\langle\ln\tau_A\rangle}$	$\langle\ln\tau_B\rangle$	$\sigma_{\langle\ln\tau_B\rangle}$
(K)	(s)		(s)		(emu/mol)						(emu/mol)		(emu/mol)		ln (s)		ln (s)	
62.00	8.22E-2	4.19E-3	1.01E-1	2.99E-2	7.10E-2	2.83E-3	1.18E-3	4.38E-5	4.01E-2	2.93E-2	9.55E-2	4.08E-3	6.23E-4	2.60E-3	-2.50	0.88	-6.74	0.53
65.00	4.68E-2	1.87E-3	4.40E-2	2.48E-2	6.42E-2	1.91E-3	5.52E-4	3.08E-5	1.20E-2	3.42E-2	9.13E-2	5.29E-3	3.97E-10	4.35E-3	-3.06	0.56	-7.50	0.28
68.00	2.16E-2	8.47E-4	8.63E-2	2.14E-2	6.45E-2	1.74E-3	2.56E-4	1.62E-5	5.86E-15	3.57E-2	8.51E-2	5.55E-3	3.57E-21	4.66E-3	-3.83	0.81	-8.27	0.00
71.00	9.05E-3	5.45E-4	7.81E-2	3.58E-2	6.23E-2	3.20E-3	1.17E-4	2.51E-5	1.33E-18	8.47E-2	7.94E-2	1.56E-2	1.96E-19	1.39E-2	-4.71	0.76	-9.05[a]	0.00[a]
74.00	3.79E-3	2.80E-4	5.50E-2	3.70E-2	5.79E-2	4.09E-3	6.20E-5	4.87E-5	1.75E-19	1.93E-1	7.43E-2	4.82E-2	1.08E-14	4.55E-2	-5.58	0.63	-9.69[a]	0.00[a]
77.00	1.76E-3	1.69E-4	5.30E-2	3.64E-2	5.87E-2	6.53E-3	3.58E-5	9.63E-5	2.99E-20	4.76E-1	6.85E-2	1.47E-1	3.68E-15	1.42E-1	-6.34	0.62	-10.24[a]	0.00[a]

[a] Relaxation times are unreliable as they lie outside the observable frequency range

Table S21. Best fit parameters to the generalized Debye model for peak A in **5%Dy@2-Y** in zero dc field, between 80–86 K.

T	τ_A	τ_A^{err}	χ_S	χ_S^{err}	χ_T	χ_T^{err}	α_A	α_A^{err}	$\langle\ln\tau_A\rangle$	$\sigma_{\langle\ln\tau_A\rangle}$
(K)	(s)		(emu/mol)		(emu/mol)				ln (s)	
80.00	6.91E-4	3.79E-5	5.56E-2	2.34E-3	1.21E-1	5.54E-4	7.79E-2	2.62E-2	-7.28	0.76
83.00	3.44E-4	1.87E-5	5.16E-2	2.28E-3	1.15E-1	4.74E-4	8.68E-2	2.42E-2	-7.98	0.81
86.00	1.82E-4	2.07E-5	4.65E-2	5.04E-3	1.10E-1	5.10E-4	6.31E-2	3.79E-2	-8.61	0.68

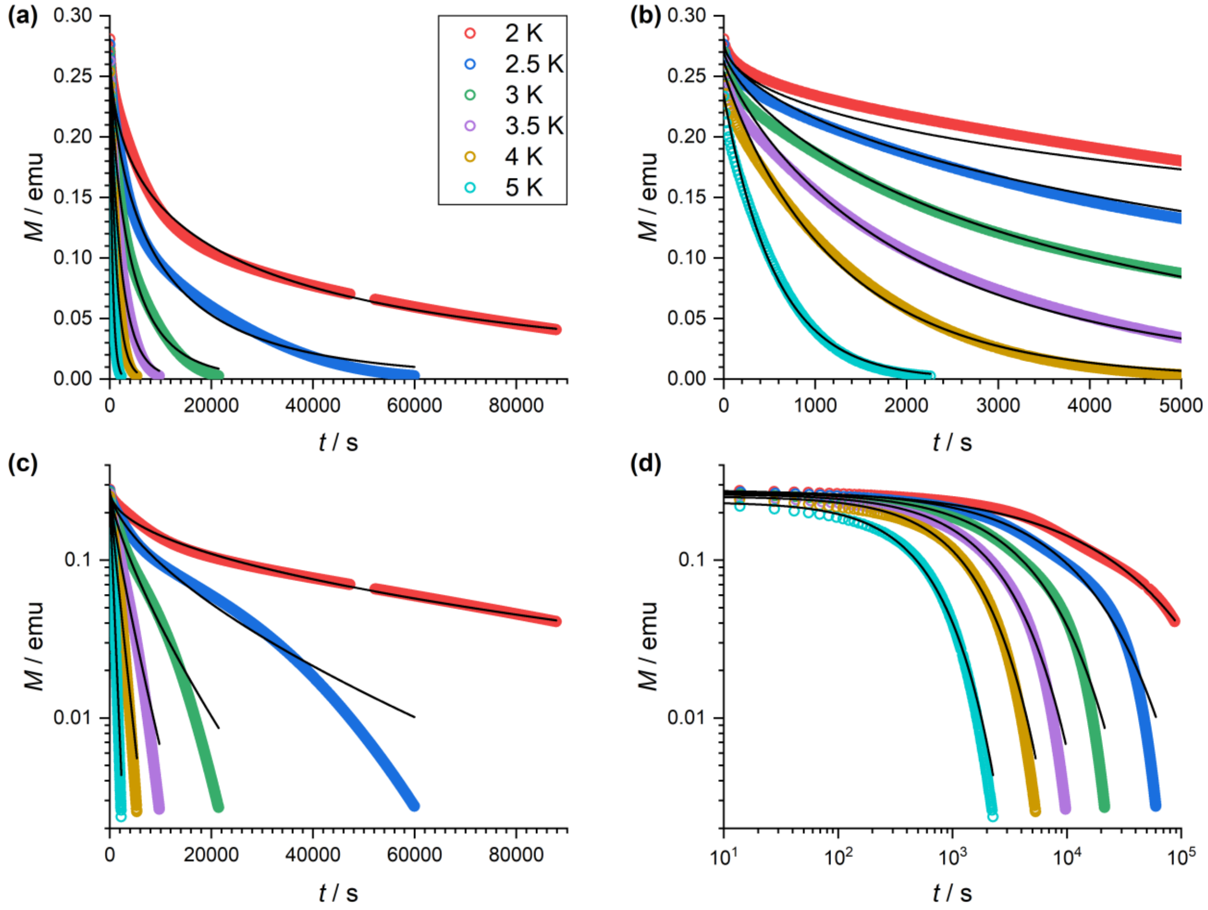

Figure S109. Magnetization decays for **2-Dy** at 2–5 K, fitted to a stretched exponential model with parameters given in Table S22.

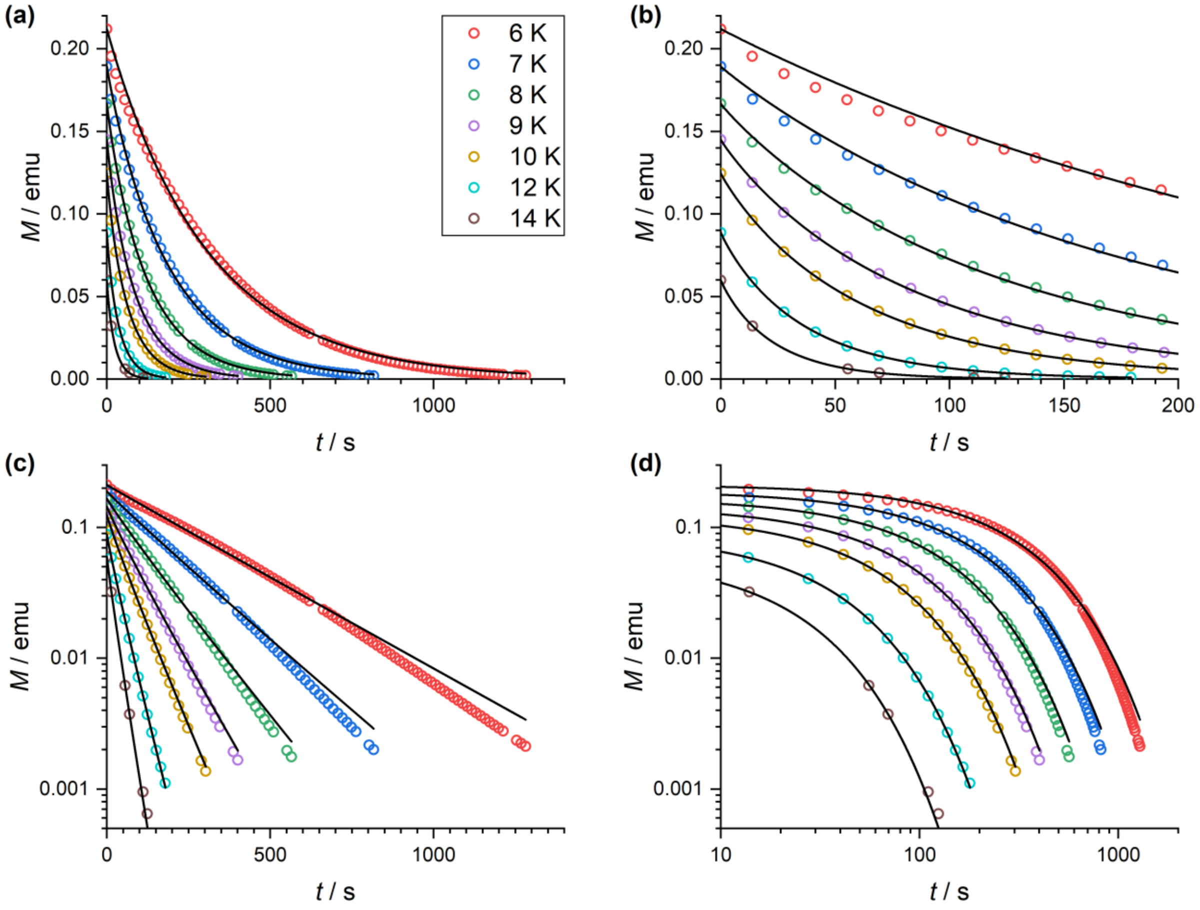

Figure S110. Magnetization decays for **2-Dy** at 6–14 K, fitted to a stretched exponential model with parameters given in Table S22.

Table S22. Best fit parameters of magnetization decays to stretched exponential model for **2-Dy** in calibrated zero dc field.

T	M_0	M_{sat}	M_0/M_{sat}	τ*	τ*^{err}	β	β^{err}	$\langle \ln \tau \rangle$	$\sigma_{\langle \ln \tau \rangle}$
(K)	(emu)	(emu)		(s)				ln (s)	
2.00	0.2810	0.3302	0.8509	22769	27	0.47932	6.3E-4	9.4062	2.3484
2.50	0.2764	0.3292	0.8398	9021	19	0.6313	1.3E-3	8.7702	1.5757
3.00	0.2703	0.3267	0.8271	4090	10	0.7468	2.2E-3	8.1205	1.1422
3.50	0.2625	0.3244	0.8091	2155.3	6.4	0.8567	3.4E-3	7.5791	0.7722
4.00	0.2537	0.3217	0.7886	1279.1	4.9	0.9403	5.3E-3	7.1173	0.4642
5.00	0.2337	0.3153	0.7414	567.2	3.0	1.0000	8.3E-3	6.3407	0.0000[a]
6.00	0.2120	0.3078	0.6888	306.1	1.7	0.9912	8.6E-3	5.7189	0.1715
7.00	0.1893	0.2995	0.6321	185.5	1.0	0.9646	7.8E-3	5.2018	0.3505
8.00	0.1668	0.2905	0.5741	121.46	0.59	0.9459	6.7E-3	4.7666	0.4401
9.00	0.1450	0.2811	0.5159	84.37	0.32	0.9357	5.2E-3	4.3956	0.4834
10.00	0.1246	0.2715	0.4590	61.24	0.17	0.9311	3.7E-3	4.0721	0.5026
12.00	0.0888	0.2525	0.3516	35.967	2.8E-2	0.9305	1.1E-3	3.5395	0.5047
14.00	0.0599	0.2339	0.2561	23.191	9.7E-2	0.9311	5.1E-3	3.1010	0.5026

[a] Best fit is a monoexponential model. To avoid overbiasing this datapoint in fitting of the relaxation profile, the $\sigma_{\langle \ln \tau \rangle}$ value for 6 K was used for 5 K data when fitting the relaxation profile.

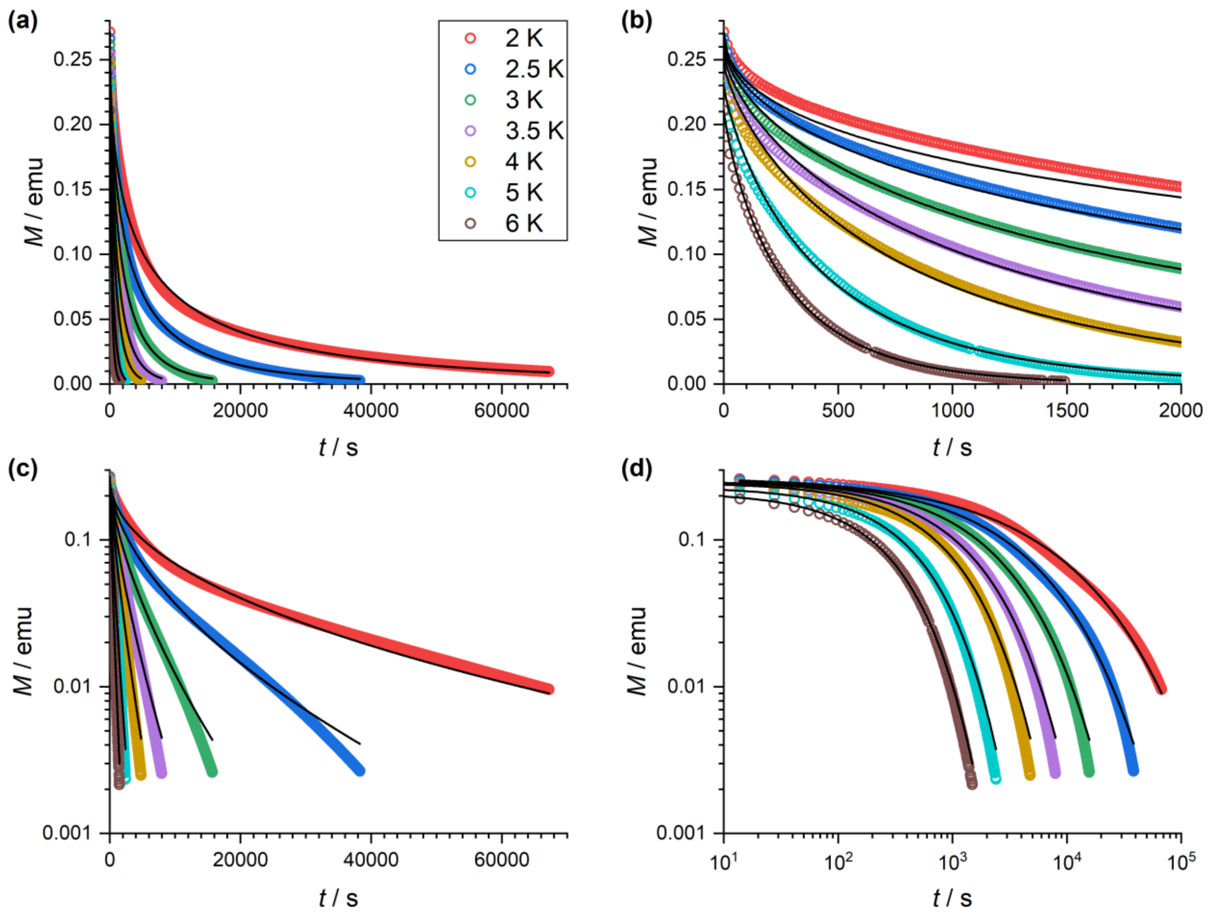

Figure S111. Magnetization decays for **3-Dy** at 2–6 K, fitted to a stretched exponential model with parameters given in Table S23.

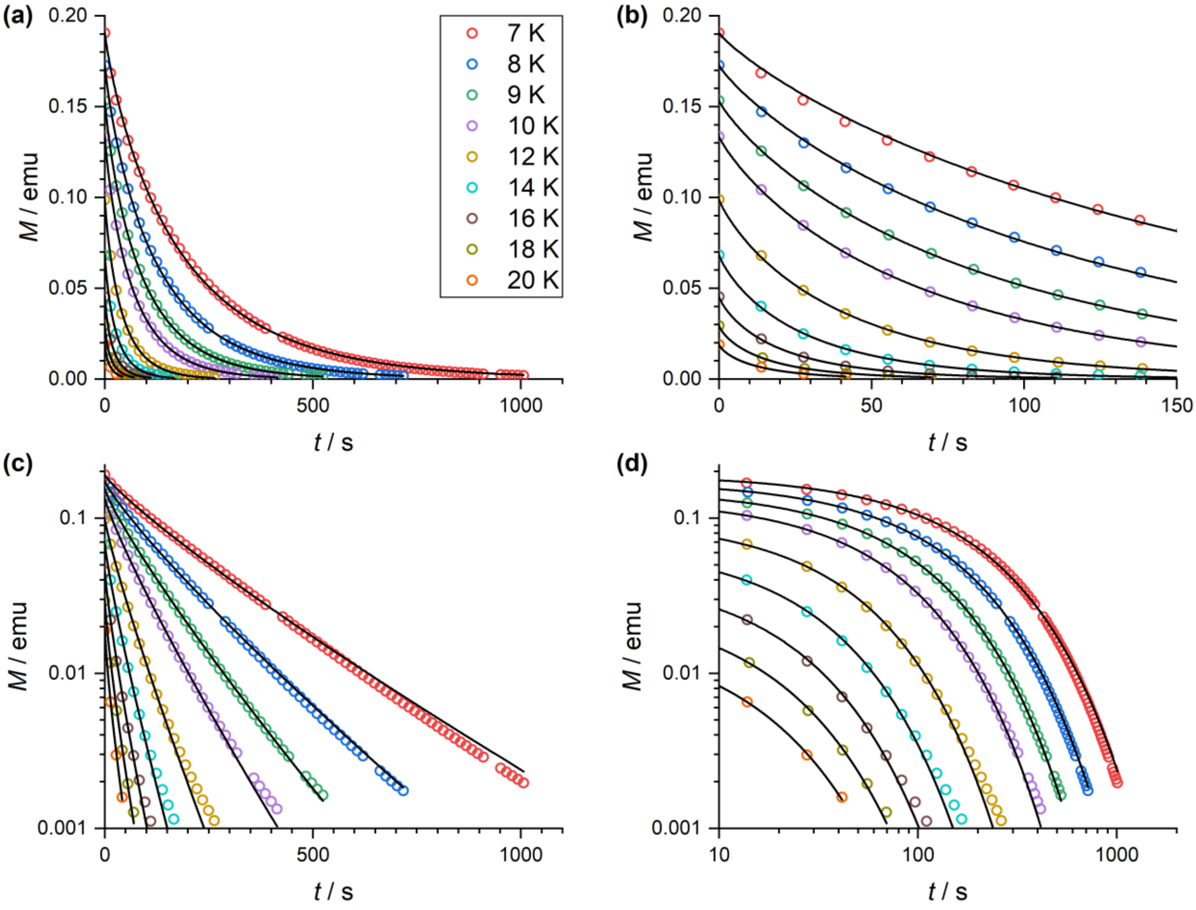

Figure S112. Magnetization decays for **3-Dy** at 7–20 K, fitted to a stretched exponential model with parameters given in Table S23.

Table S23. Best fit parameters of magnetization decays to stretched exponential model for **3-Dy** in calibrated zero dc field.

T	M_0	M_{sat}	M_0/M_{sat}	τ*	$τ*^{err}$	B	$β^{err}$	$\langle \ln \tau \rangle$	$\sigma_{\langle \ln \tau \rangle}$
	(emu)	(emu)		(s)				ln (s)	
2.00	0.2714	0.3351	0.8100	5187	10	0.47827	6.5E-4	7.9242	2.3551
2.50	0.2664	0.3336	0.7987	2959.0	3.6	0.55859	5.4E-4	7.5365	1.9045
3.00	0.2608	0.3317	0.7862	1778.1	1.9	0.64736	6.7E-4	7.1689	1.5101
3.50	0.2542	0.3294	0.7716	1158.3	2.9	0.7244	2.0E-3	6.8350	1.2206
4.00	0.2469	0.3267	0.7557	804.3	3.0	0.7805	3.5E-3	6.5276	1.0274
5.00	0.2295	0.3202	0.7167	442.0	2.0	0.8361	4.9E-3	5.9780	0.8417
6.00	0.2110	0.3129	0.6745	272.1	1.1	0.8525	4.6E-3	5.5062	0.7866
7.00	0.1907	0.3045	0.6262	181.12	0.57	0.8643	3.6E-3	5.1086	0.7462
8.00	0.1728	0.2955	0.5847	124.70	0.29	0.8654	2.6E-3	4.7362	0.7425
9.00	0.1532	0.2860	0.5358	89.943	9.2E-2	0.8687	1.2E-3	4.4119	0.7314
10.00	0.1335	0.2764	0.4832	67.558	9.8E-2	0.8730	1.7E-3	4.1290	0.7167
12.00	0.0988	0.2573	0.3840	41.29	0.23	0.8688	6.5E-3	3.6336	0.7309
14.00	0.0683	0.2384	0.2865	27.77	0.28	0.854	1.2E-2	3.2252	0.7816
16.00	0.0453	0.2210	0.2050	20.19	0.29	0.830	1.7E-2	2.8868	0.8627
18.00	0.0294	0.2053	0.1431	15.60	0.25	0.800	1.8E-2	2.6032	0.9614
20.00	0.0189	0.1913	0.0990	12.77	0.15	0.783	1.4E-2	2.3871	1.0193

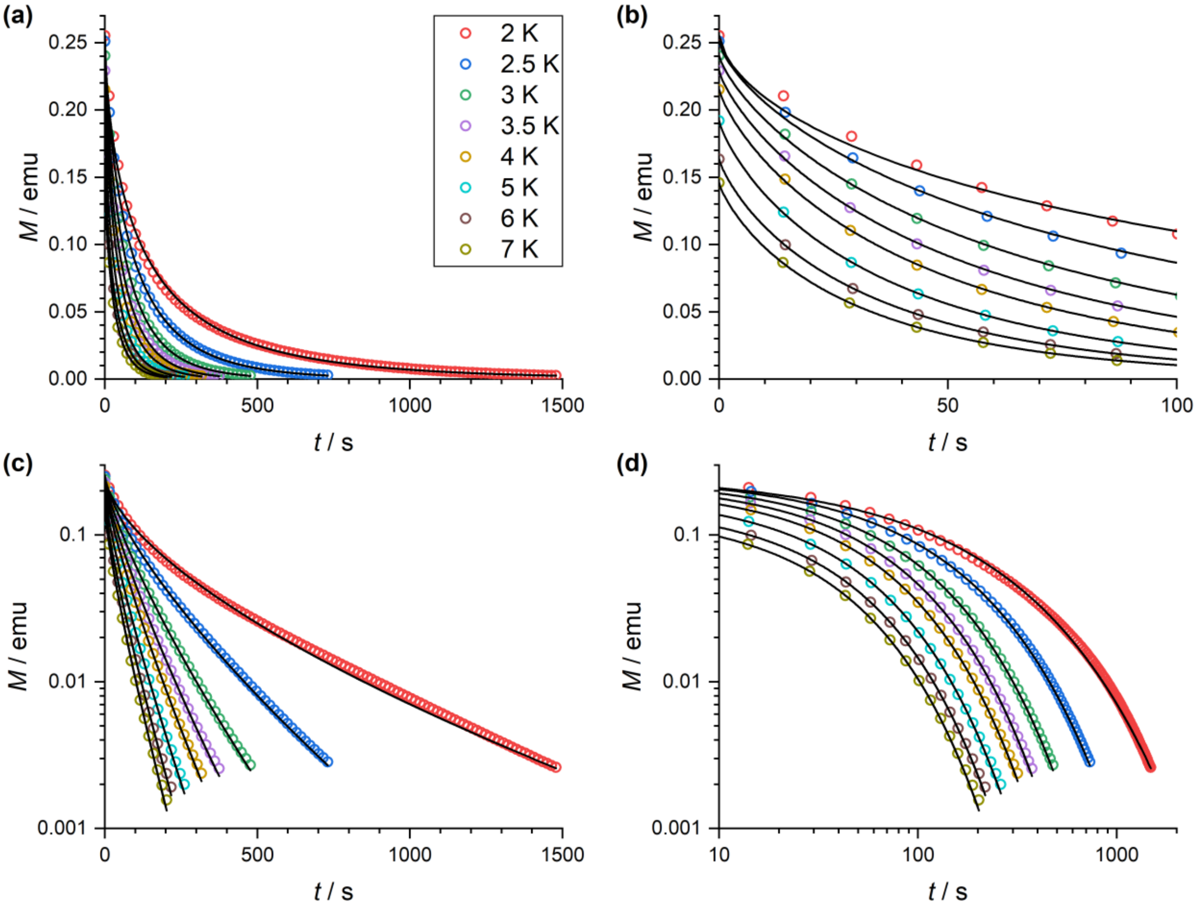

Figure S113. Magnetization decays for **4-Dy** at 2–7 K, fitted to a stretched exponential model with parameters given in Table S24.

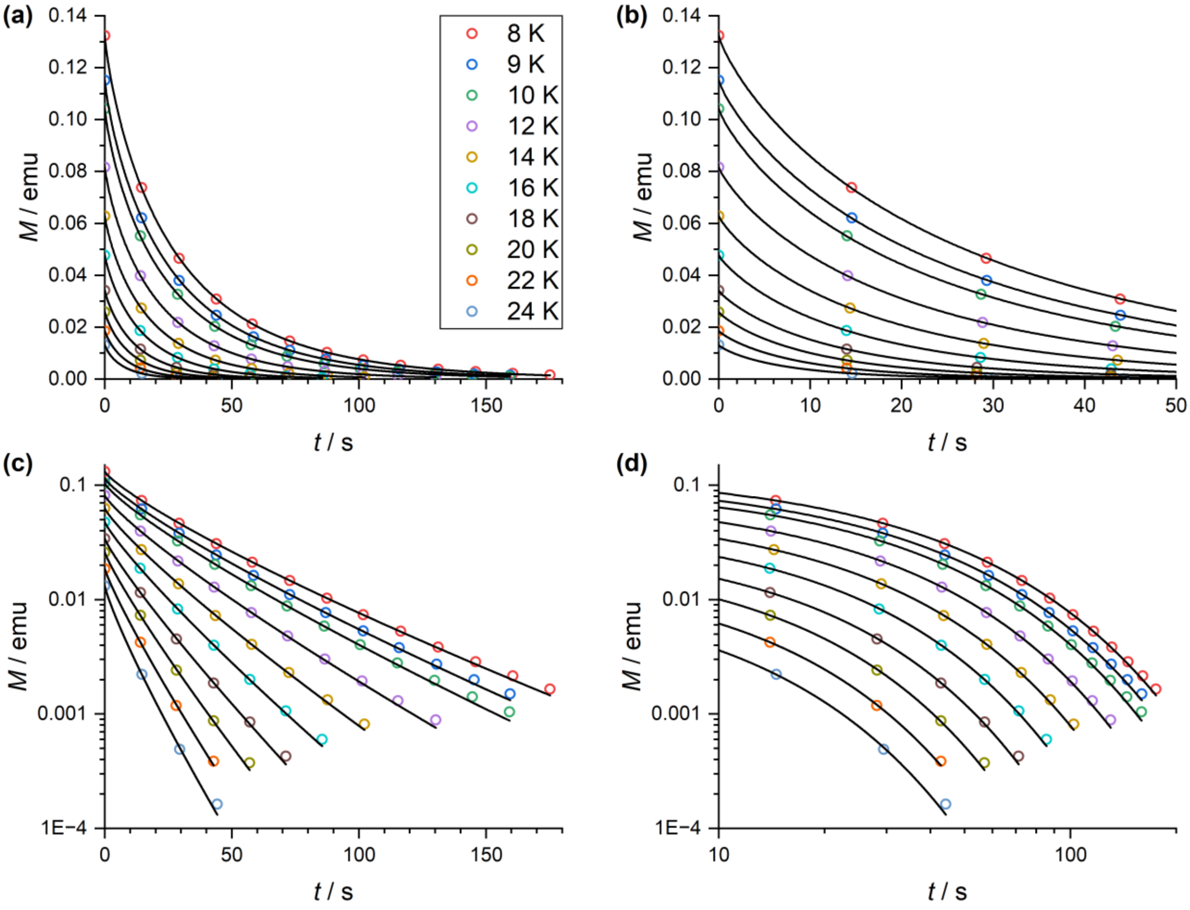

Figure S114. Magnetization decays for **4-Dy** at 8–24 K, fitted to a stretched exponential model with parameters given in Table S24.

Table S24. Best fit parameters of magnetization decays to stretched exponential model for **4-Dy** in calibrated zero dc field.

T	M_0	M_{sat}	M_0/M_{sat}	τ*	$τ^{*err}$	Β	$β^{err}$	$\langle \ln \tau \rangle$	$\sigma_{\langle \ln \tau \rangle}$
(K)	(emu)	(emu)		(s)				ln (s)	
2.00	0.2552	0.3681	0.6933	131.42	0.87	0.6301	3.8E-3	4.5396	1.5806
2.50	0.2511	0.3664	0.6852	91.65	0.37	0.7282	3.2E-3	4.3026	1.2071
3.00	0.2405	0.3642	0.6603	68.67	0.21	0.7845	2.9E-3	4.0707	1.0137
3.50	0.2292	0.3615	0.6339	55.73	0.17	0.8028	3.0E-3	3.8787	0.9526
4.00	0.2153	0.3585	0.6005	47.61	0.13	0.8085	2.7E-3	3.7264	0.9336
5.00	0.1920	0.3514	0.5463	38.572	8.1E-2	0.8108	2.1E-3	3.5178	0.9260
6.00	0.1636	0.3431	0.4768	33.949	5.5E-2	0.8181	1.7E-3	3.3965	0.9015
7.00	0.1459	0.3337	0.4373	30.637	6.1E-2	0.8196	2.1E-3	3.2951	0.8966
8.00	0.1324	0.3236	0.4091	27.833	4.8E-2	0.8185	1.8E-3	3.1983	0.9001
9.00	0.1152	0.3130	0.3679	26.008	5.6E-2	0.8254	2.3E-3	3.1363	0.8772
10.00	0.1042	0.3022	0.3448	24.095	5.6E-2	0.8282	2.6E-3	3.0623	0.8677
12.00	0.0817	0.2808	0.2908	20.825	5.4E-2	0.8416	3.0E-3	2.9275	0.8231
14.00	0.0629	0.2600	0.2417	17.791	4.4E-2	0.8552	3.0E-3	2.7809	0.7773
16.00	0.0477	0.2410	0.1981	15.089	4.6E-2	0.8686	3.8E-3	2.6266	0.7316
18.00	0.0342	0.2236	0.1527	12.769	5.6E-2	0.8800	5.7E-3	2.4682	0.6924
20.00	0.0259	0.2081	0.1245	10.687	6.8E-2	0.8825	8.2E-3	2.2922	0.6833
22.00	0.0186	0.1942	0.0958	8.976	7.9E-2	0.880	1.1E-2	2.1162	0.6905
24.00	0.0132	0.1818	0.0723	7.42	0.15	0.855	2.1E-2	1.9060	0.7782

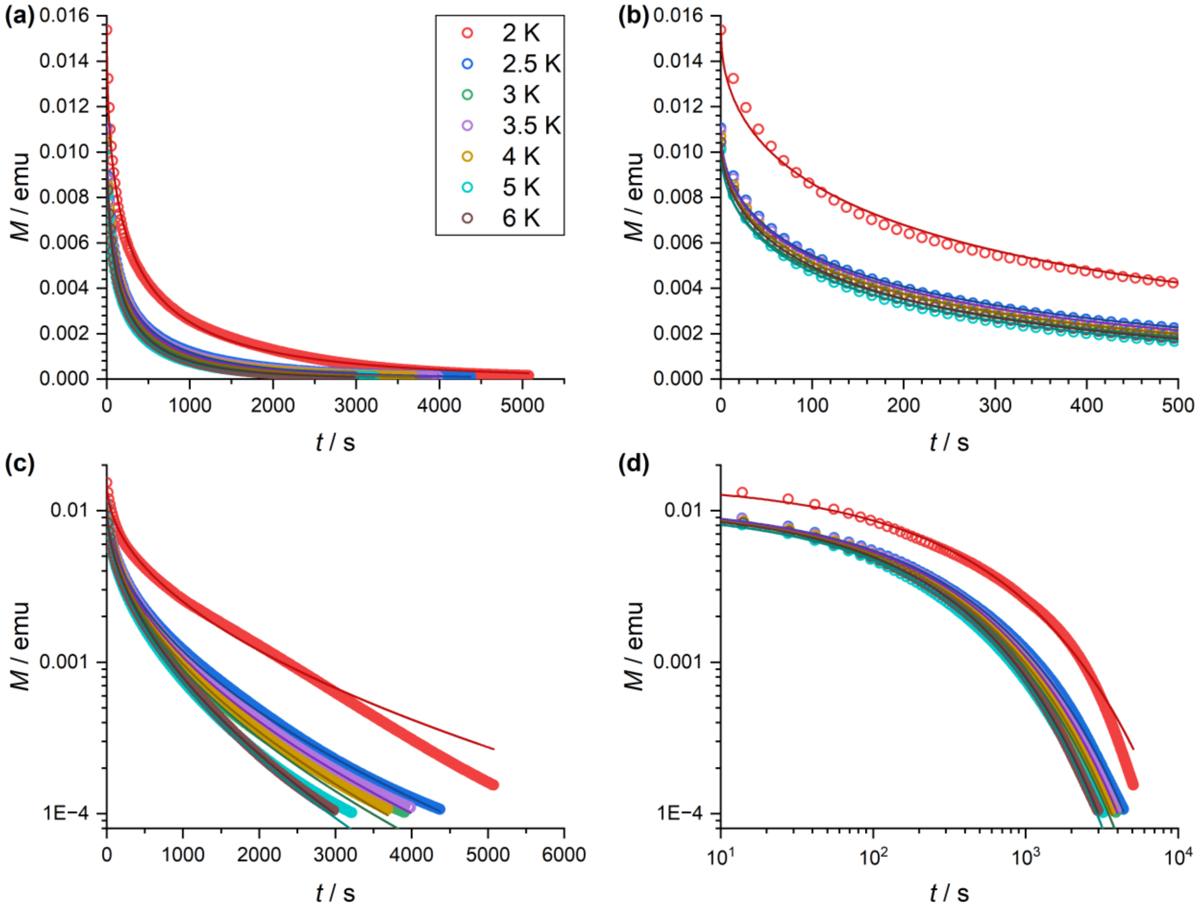

Figure S115. Magnetization decays for **2%Dy@2-Y** at 2–6 K, fitted to a stretched exponential model with parameters given in Table S25.

.

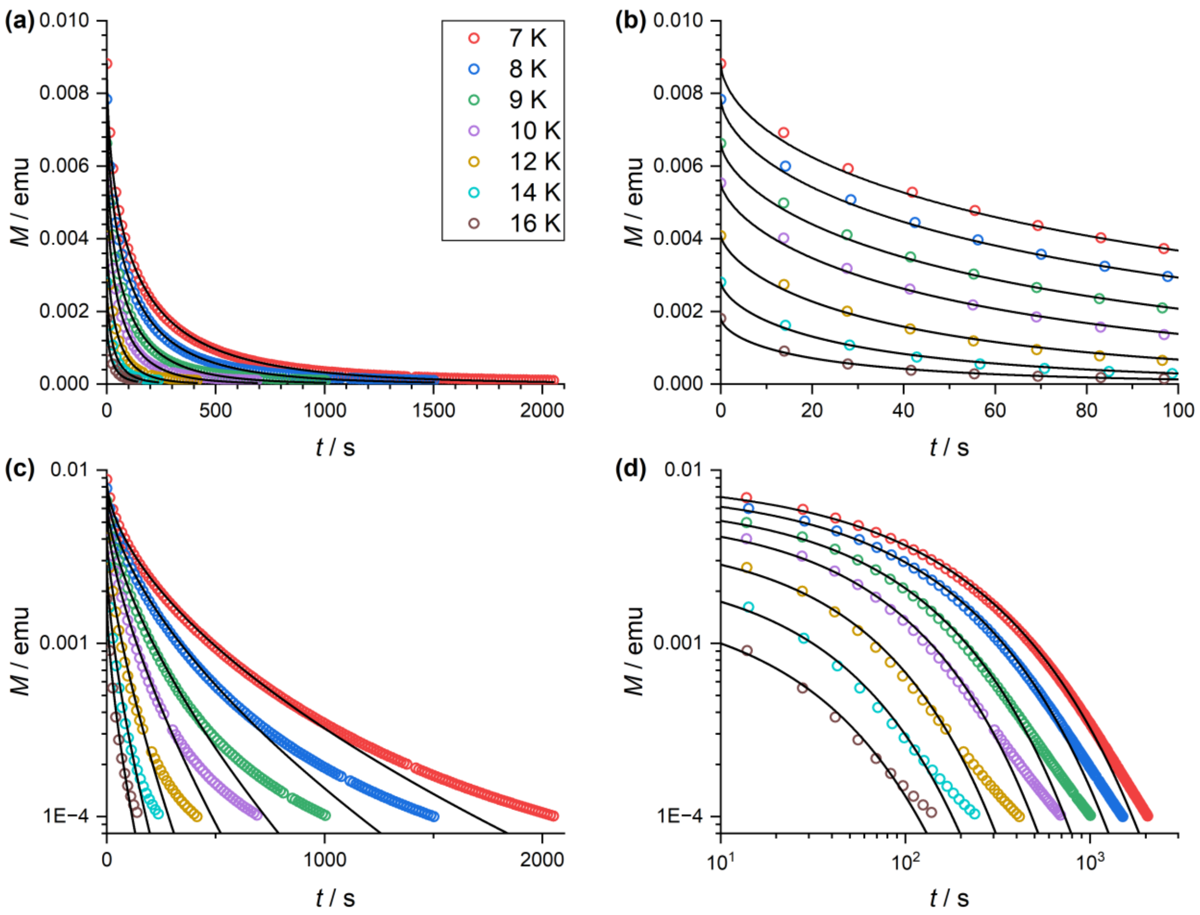

Figure S116. Magnetization decays for **2%Dy@2-Y** at 7–16 K, fitted to a stretched exponential model with parameters given in Table S25.

Table S25. Best fit parameters of magnetization decays to stretched exponential model for **5%Dy@-2-Y** in calibrated zero dc field.

T	M_0	M_{sat}	M_0/M_{sat}	τ*	$τ*^{err}$	B	$β^{err}$	$\langle \ln \tau \rangle$	$\sigma_{\langle \ln \tau \rangle}$
(K)	(emu)	(emu)		(s)					(s)
2.00	1.538E-2	3.805E-2	0.4041	301.0	1.8	0.4955	2.0E-3	5.119	2.249
2.50	1.107E-2	3.583E-2	0.3088	200.38	0.60	0.4998	1.0E-3	4.723	2.222
3.00	1.019E-2	3.526E-2	0.2888	178.36	0.71	0.5152	1.4E-3	4.641	2.134
3.50	1.096E-2	3.550E-2	0.3086	192.39	0.51	0.50937	9.3E-4	4.704	2.167
4.00	1.069E-2	3.527E-2	0.3031	183.28	0.50	0.51541	9.7E-4	4.668	2.132
5.00	1.010E-2	3.460E-2	0.2920	165.77	0.54	0.5331	1.3E-3	4.605	2.036
6.00	1.045E-2	3.454E-2	0.3024	172.49	0.37	0.53861	8.6E-4	4.656	2.006
7.00	8.823E-3	3.324E-2	0.2654	126.04	0.57	0.5774	2.1E-3	4.414	1.813
8.00	7.838E-3	3.232E-2	0.2425	102.91	0.66	0.6081	3.4E-3	4.262	1.674
9.00	6.627E-3	3.118E-2	0.2126	79.52	0.79	0.6473	6.0E-3	4.062	1.510
10.00	5.535E-3	3.009E-2	0.1840	61.70	0.90	0.6755	9.9E-3	3.845	1.400
12.00	4.088E-3	2.829E-2	0.1445	43.09	0.99	0.696	1.7E-2	3.511	1.323
14.00	2.804E-3	2.634E-2	0.1065	30.03	0.92	0.674	2.1E-2	3.123	1.405
16.00	1.806E-3	2.450E-2	0.0737	22.54	0.86	0.648	2.6E-2	2.802	1.509

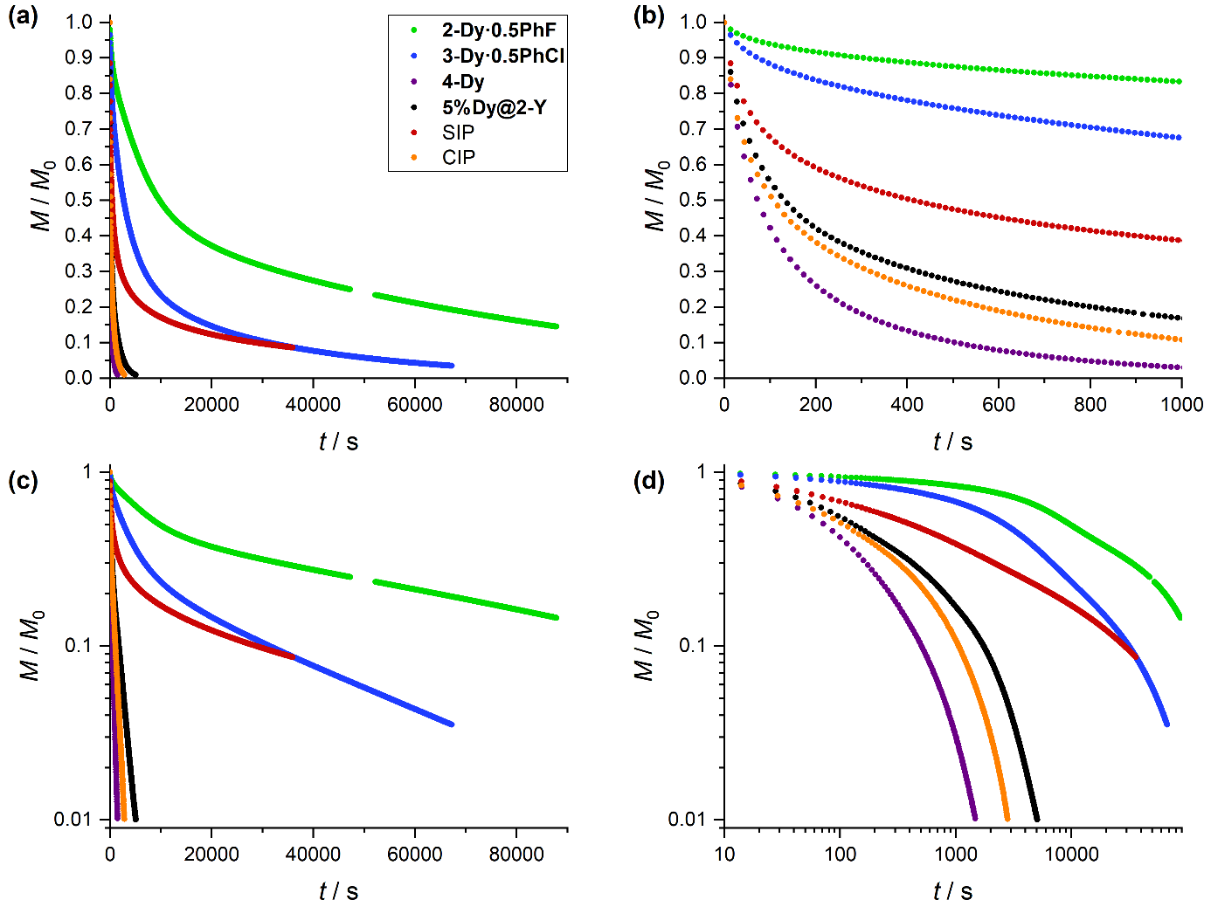

Figure S117. Comparison of magnetization decays at 2 K, normalized to M_0.

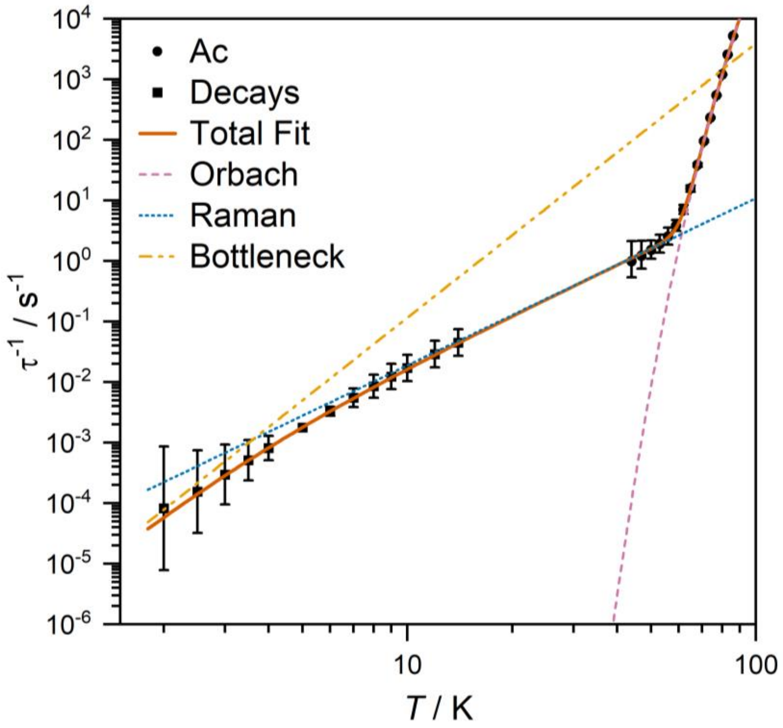

Figure S118. Fit of **2-Dy** relaxation profile with Orbach and Raman processes and phonon bottleneck limit. Error bars represent one ESD in the distribution of rates.

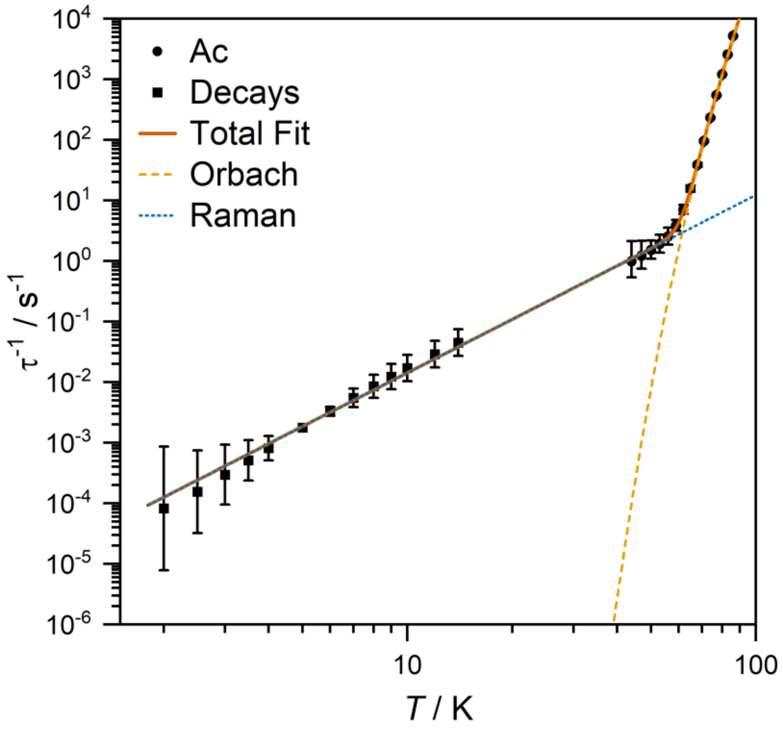

Figure S119. Alternative fit of **2-Dy** relaxation profile with Orbach and Raman processes only. Error bars represent one ESD in the distribution of rates.

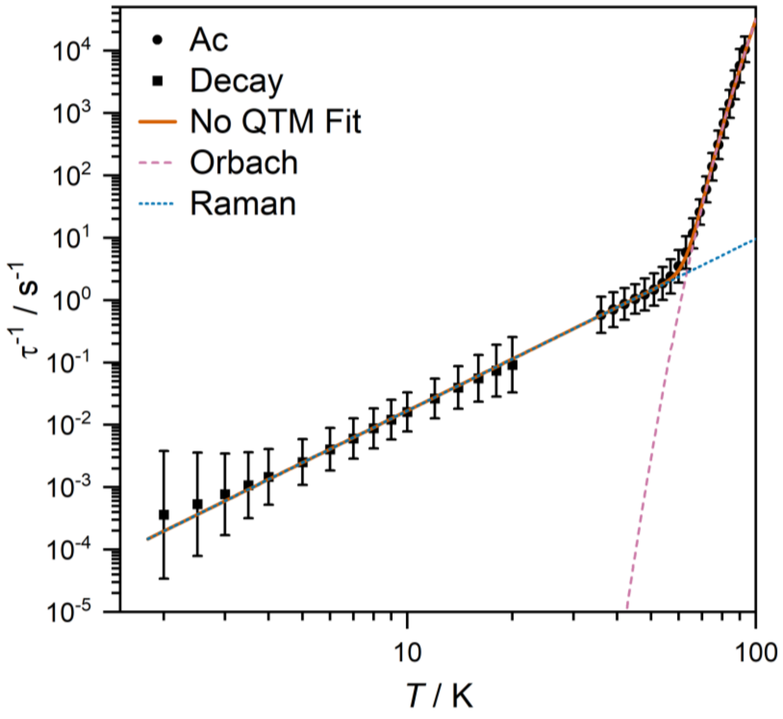

Figure S120. Fit of **3-Dy** relaxation profile without QTM, showing Orbach and Raman components. Error bars represent one ESD in the distribution of rates.

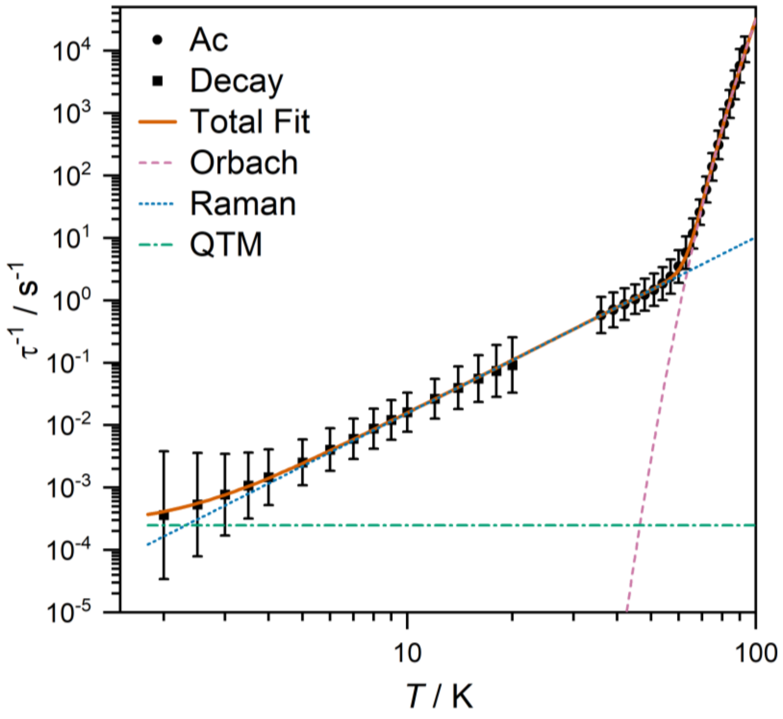

Figure S121. Alternative fit of **3-Dy** relaxation profile, showing Orbach, Raman and QTM components. Error bars represent one ESD in the distribution of rates.

Table S26. Comparison of relaxation profile fitting parameters with and without phonon bottleneck for **2-Dy**.

	Without phonon bottleneck	With phonon bottleneck
U_{eff} / cm^{-1}	1106(13)	1100(9)
τ_0 / s	$10^{-11.7(1)}$	$10^{-11.66(8)}$
C / s^{-1} K^{-n}	$10^{-4.78(2)}$	$10^{-4.5(2)}$
n	2.94(2)	2.8(1)
B / s K^{m}	n/a	$10^{5.5(3)}$
m	n/a	4.5(7)

Table S27. Comparison of relaxation profile fitting parameters with and without QTM term for **3-Dy**.

	Without QTM	With QTM
U_{eff} / cm^{-1}	1125(12)	1128(9)
τ_0 / s	$10^{-11.53(9)}$	$10^{-11.56(7)}$
C / s^{-1} K^{-n}	$10^{-4.53(3)}$	$10^{-4.63(3)}$
n	2.76(2)	2.82(2)
τ_{QTM} / s	n/a	$10^{3.6(1)}$

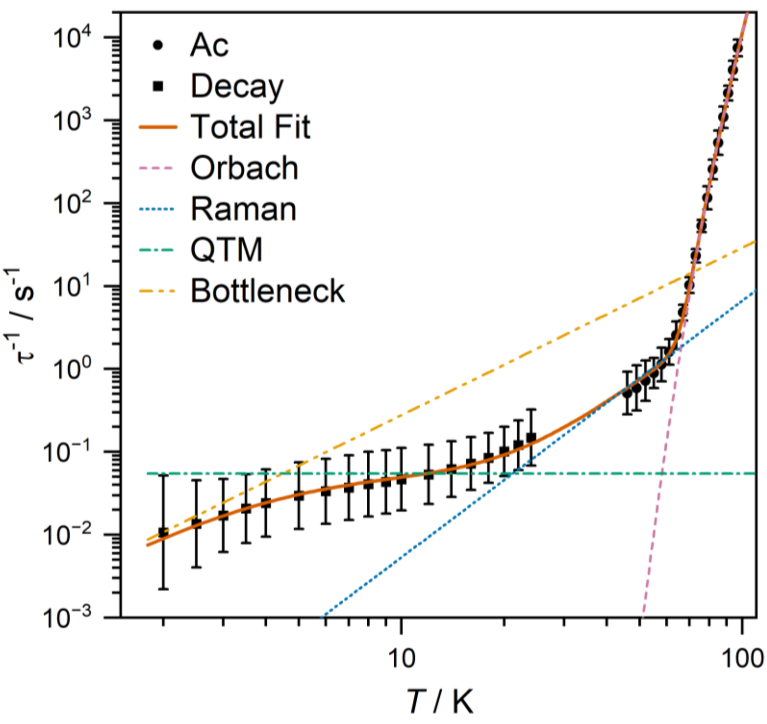

Figure S122. Fitting of **4-Dy** relaxation profile, showing Orbach, Raman and QTM components and phonon bottleneck limit on Raman and QTM rates. Error bars represent one ESD in the distribution of rates.

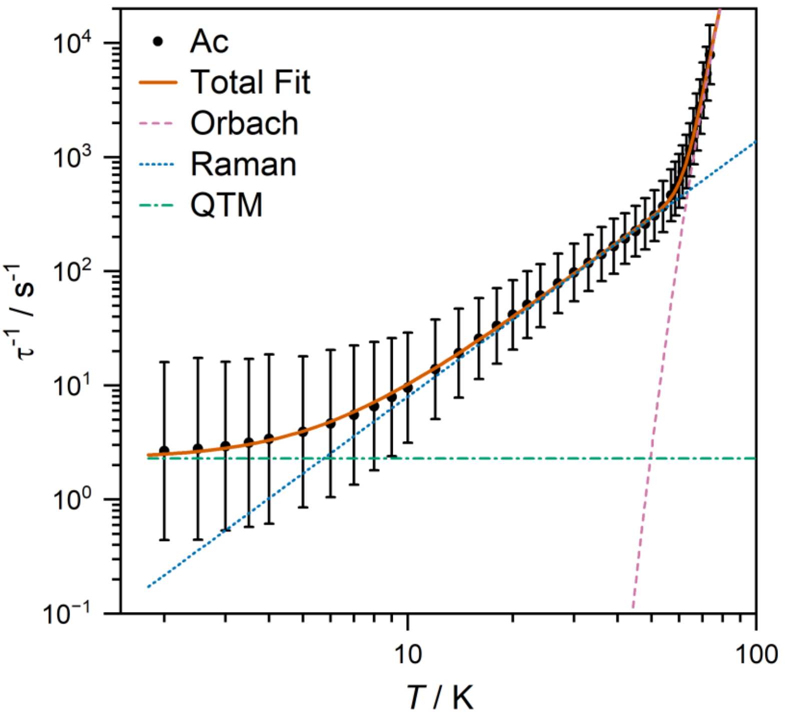

Figure S123. Fitting of **5-Dy** relaxation profile, showing Orbach, Raman and QTM components. Error bars represent one ESD in the distribution of rates.

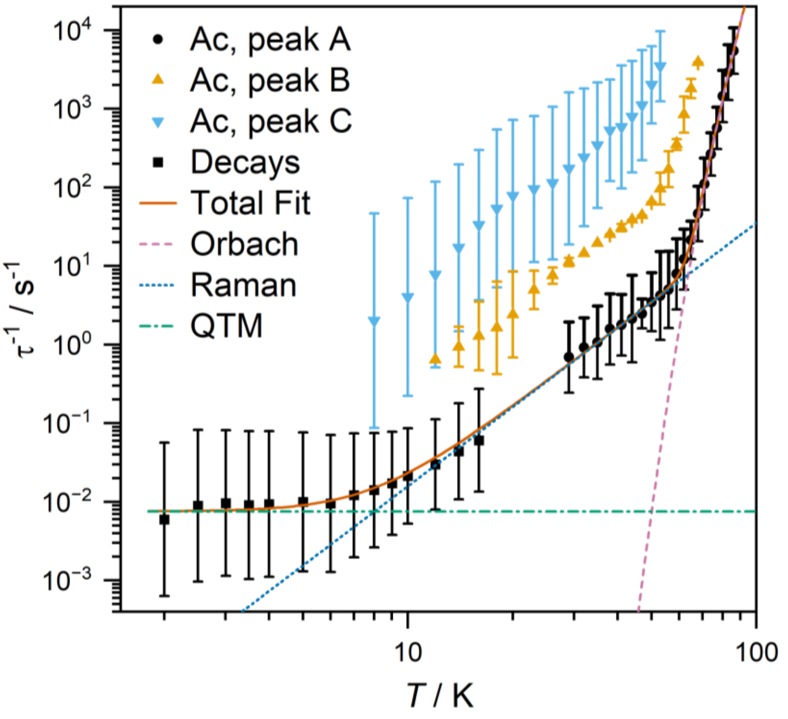

Figure S124. Fitting of **5%Dy@2-Y** relaxation profile, showing Orbach, Raman and QTM components. Error bars represent one ESD in the distribution of rates.

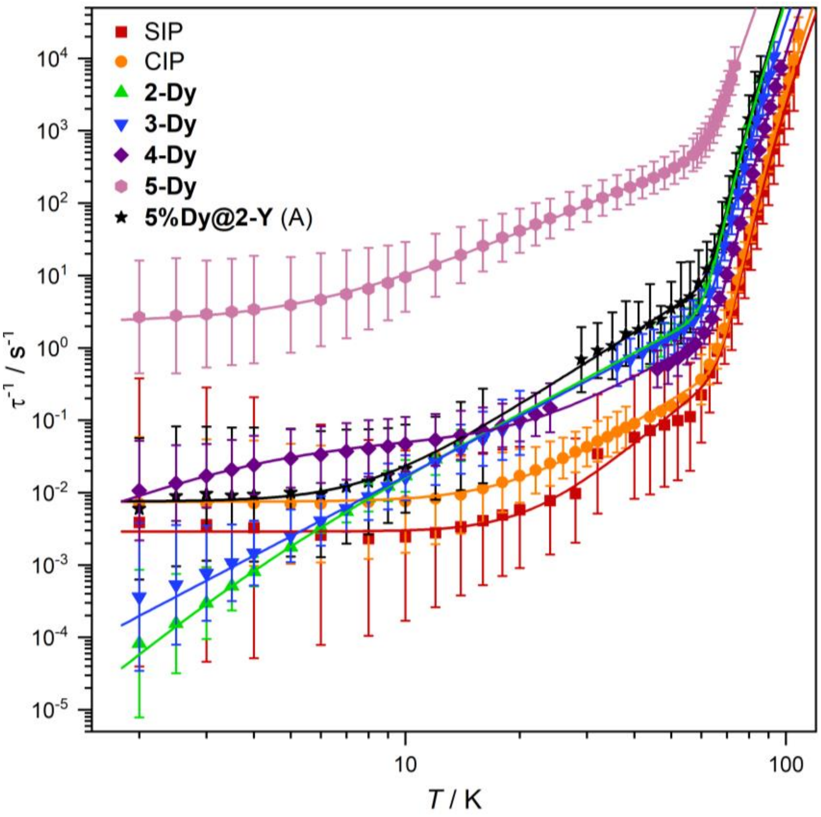

Figure S125. Combined relaxation profiles of SIP, CIP, **2-Dy**, **3-Dy**, **4-Dy**, **5-Dy** and **5%Dy@2-Y** (peak A).

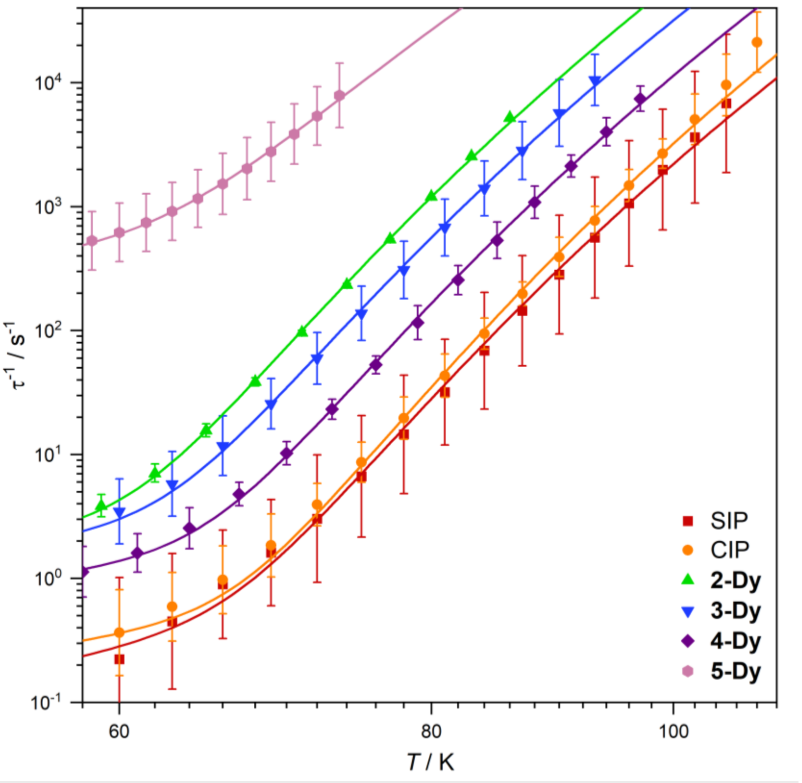

Figure S126. Comparison of high-temperature relaxation profiles of SIP, CIP, **2-Dy**, **3-Dy**, **4-Dy** and **5-Dy**.

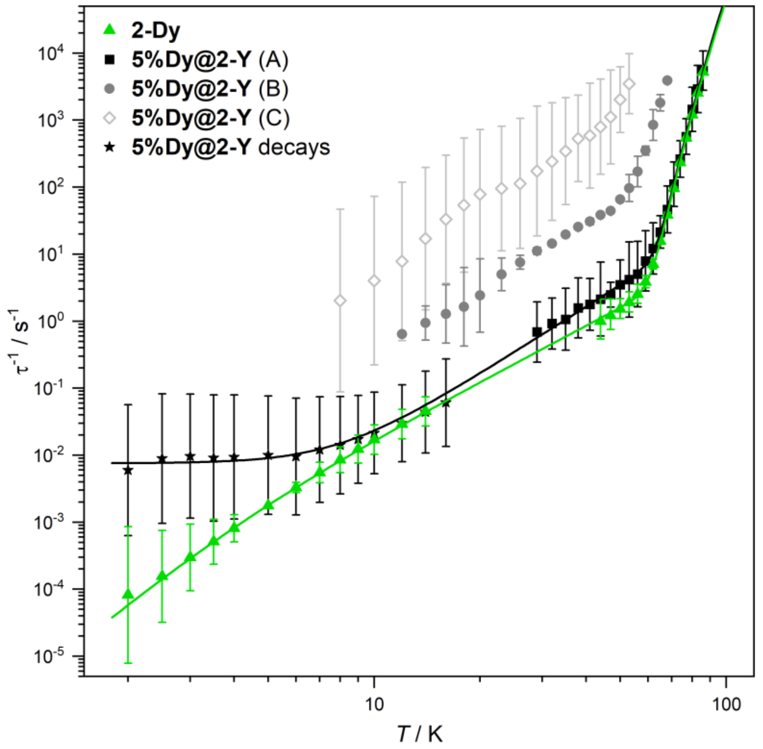

Figure S127. Combined relaxation profiles of **2-Dy** and **5%Dy@2-Y** (peaks A, B and C).

9. CASSCF-SO Calculations

OpenMolcas[29] was used to perform CASSCF-SO calculations on $[Dy(Cp^{ttt})(Cp^*)(PhF\text{-}\kappa\text{-}F)]^+$ (**2′-Dy**), $[Dy(Cp^{ttt})(Cp^*)(PhCl\text{-}\kappa\text{-}Cl)]^+$ (**3′-Dy**), $[Dy(Cp^{ttt})(Cp^*)(PhBr\text{-}\kappa\text{-}Br)]^+$ (**4′-Dy**) and $[Dy(Cp^{ttt})(Cp^*)(C_6H_4F_2\text{-}\kappa^2\text{-}F,F)]^+$ (**5′-Dy**) to determine their electronic structures. The molecular geometries from the single crystal XRD structures were used with no optimization, taking the largest disorder component only. Integrals were performed in the SEWARD module using basis sets from ANO-RCC library[30–33] with VTZP quality for Dy atoms, VDZP quality for the cyclopentadienyl C atoms the fluorobenzene F atom (**2′-Dy**), the *ortho*-difluorobenzene F atoms (**5′-Dy**), the chlorobenzene Cl atom (**3′-Dy**), bromobenzene Br atom (**4′-Dy**) and VDZ quality for all remaining atoms, employing the second-order DKH transformation. Cholesky decomposition of the two-electron integrals with a threshold of 10^{-8} was performed to save disk space and reduce computational demand. The molecular orbitals (MOs) were optimised in state-averaged CASSCF calculations in the RASSCF module, where the active space was defined by the nine electrons in the seven 4f orbitals of Dy(III). Three such calculations were performed independently for each possible spin state, where 21 roots were included for S = 5/2, 224 roots were included for S = 3/2, and 490 roots were included for S = 1/2. The wavefunctions obtained from these CASSCF calculations were then mixed by spin orbit coupling in the RASSI module, where all 21 S = 5/2 states, 128 of the S = 3/2 states, and 130 of the S = 1/2 states were included**.** SINGLE_ANISO was used to decompose the resulting spin-orbit wave functions into the CF Hamiltonian formalism.[34] Diamond was employed for molecular graphics.[35]

Table S28. Electronic structure of **2′-Dy** calculated with the crystal field parameters obtained from CASSCF-SO using the solid state geometry of **2′-Dy** in zero-field. Each row corresponds to a Kramers doublet.

Energy (cm^{-1})	Energy (K)	g_x	g_y	g_z	Angle[a] (deg)	Wavefunction	$<J_z>$
0.00	0.00	0.0001	0.0001	19.84	--	98.9% |± 15/2⟩	±7.478
396.67	570.81	0.003	0.004	17.0	0.4	98% |± 13/2⟩	±6.467
648.92	933.79	0.03	0.03	14.4	0.3	97% |± 11/2⟩	±5.482
810.87	1166.84	0.07	0.2	11.6	1.1	94% |± 9/2⟩	±4.426
924.61	1330.51	3.2	4.5	7.7	3.7	77% |± 7/2⟩ + 15% |± 3/2⟩ + 5% |∓ 1/2⟩	±2.950
1004.56	1445.56	2.8	5.7	10.0	89.8	52% |± 5/2⟩ + 25% |± 1/2⟩ + 12% |∓ 7/2⟩ + 7% |∓ 3/2⟩	±1.086
1139.53	1639.79	0.1	0.3	15.6	89.5	42% |± 3/2⟩ + 30% |∓ 5/2⟩ + 9% |∓ 1/2⟩ + 8% |± 7/2⟩ + 5% |∓ 3/2⟩	±0.085
1341.41	1930.29	0.004	0.01	19.2	89.7	30% |± 1/2⟩ + 30% |∓ 1/2⟩ + 17% |± 3/2⟩ + 15% |∓ 3/2⟩	±0.043

[a] The angle between the g_z value of the excited Kramers doublet and the ground Kramers doublet.

Table S29. Electronic structure of **3′-Dy** calculated with the crystal field parameters obtained from CASSCF-SO using the solid state geometry of **3′-Dy** in zero-field. Each row corresponds to a Kramers doublet.

Energy (cm^{-1})	Energy (K)	g_x	g_y	g_z	Angle[a] (deg)	Wavefunction	$<J_z>$
0.00	0.00	0.0000	0.0000	19.86	--	99.2% |± 15/2⟩	±7.484
405.25	583.15	0.002	0.002	17.0	0.6	99.0% |± 13/2⟩	±6.479
657.46	946.09	0.03	0.03	14.4	1.2	98% |± 11/2⟩	±5.494
823.96	1185.69	0.1	0.2	11.7	1.5	96% |± 9/2⟩	±4.471
953.74	1372.44	1.2	1.7	8.6	2.4	90% |± 7/2⟩ + 8% |± 3/2⟩	±3.296
1048.67	1509.04	4.2	7.0	7.8	89.9	65% |± 5/2⟩ + 23% |± 1/2⟩ + 7% |∓ 3/2⟩	±1.604
1159.22	1668.11	0.7	1.5	14.7	89.2	53% |± 3/2⟩ + 25% |∓ 5/2⟩ + 14% |∓ 1/2⟩ + 6% |± 7/2⟩	±0.298
1348.07	1939.88	0.02	0.04	19.1	89.6	37% |± 1/2⟩ + 25% |∓ 1/2⟩ + 17% |∓ 3/2⟩ + 14% |± 3/2⟩	±0.054

[a] The angle between the g_z value of the excited Kramers doublet and the ground Kramers doublet.

Table S30. Electronic structure of **4′-Dy** calculated with the crystal field parameters obtained from CASSCF-SO using the solid state geometry of **4′-Dy** in zero-field. Each row corresponds to a Kramers doublet.

Energy (cm^{-1})	Energy (K)	g_x	g_y	g_z	Angle[a] (deg)	Wavefunction	$<J_z>$
0.00	0.00	0.0000	0.0000	19.86	--	99.2% |± 15/2⟩	±7.483
416.55	599.41	0.002	0.002	17.0	0.8	98.7% |± 13/2⟩	±6.474
668.74	962.31	0.02	0.03	14.4	1.7	98% |± 11/2⟩	±5.485
832.02	1197.28	0.07	0.1	11.7	1.1	95% |± 9/2⟩	±4.456
960.25	1381.79	1.6	2.2	8.5	2.7	87% |± 7/2⟩ + 9% |± 3/2⟩	±3.246
1056.40	1520.15	4.0	6.8	8.4	89.2	62% |± 5/2⟩ + 22% |± 1/2⟩ + 8% |∓ 3/2⟩	±1.526
1179.58	1697.41	0.6	1.2	14.9	89.3	52% |± 3/2⟩ + 27% |∓ 5/2⟩ + 13% |∓ 1/2⟩ + 7% |± 7/2⟩	±0.262
1370.41	1972.02	0.02	0.05	19.0	89.7	40% |± 1/2⟩ + 22% |∓ 1/2⟩ + 19% |∓ 3/2⟩ + 12% |± 3/2⟩ + 5% |± 5/2⟩	±0.038

[a] The angle between the g_z value of the excited Kramers doublet and the ground Kramers doublet.

Table S31. Electronic structure of **5′-Dy** calculated with the crystal field parameters obtained from CASSCF-SO using the solid state geometry of **5′-Dy** in zero-field. Each row corresponds to a Kramers doublet.

Energy (cm^{-1})	Energy (K)	g_x	g_y	g_z	Angle[a] (deg)	Wavefunction	$\langle J_z \rangle$
0.00	0.00	0.0004	0.0005	19.84	--	98.9% |± 15/2⟩	±7.478
348.13	500.96	0.005	0.005	17.0	0.9	99.2% |± 13/2⟩	±6.484
581.61	836.94	0.02	0.03	14.4	0.8	98% |± 11/2⟩	±5.509
736.62	1060.00	0.7	0.9	11.7	0.5	98% |± 9/2⟩	±4.456
849.00	1221.72	4.4	5.2	7.7	4.5	85% |± 7/2⟩ + 7% |± 3/2⟩	±2.971
928.77	1336.50	2.8	4.3	10.4	88.9	56% |± 5/2⟩ + 27% |± 1/2⟩ + 10% |∓ 7/2⟩ + 7% |∓ 3/2⟩	±1.108
1005.89	1447.48	0.04	0.1	14.4	89.8	53% |± 3/2⟩ + 30% |∓ 5/2⟩ + 12% |∓ 1/2⟩	±0.081
1193.04	1716.79	0.03	0.07	19.3	89.6	34% |± 1/2⟩ + 24% |∓ 1/2⟩ + 19% |∓ 3/2⟩ + 12% |± 3/2⟩ + 5% |± 5/2⟩	±0.053

[a] The angle between the g_z value of the excited Kramers doublet and the ground Kramers doublet.

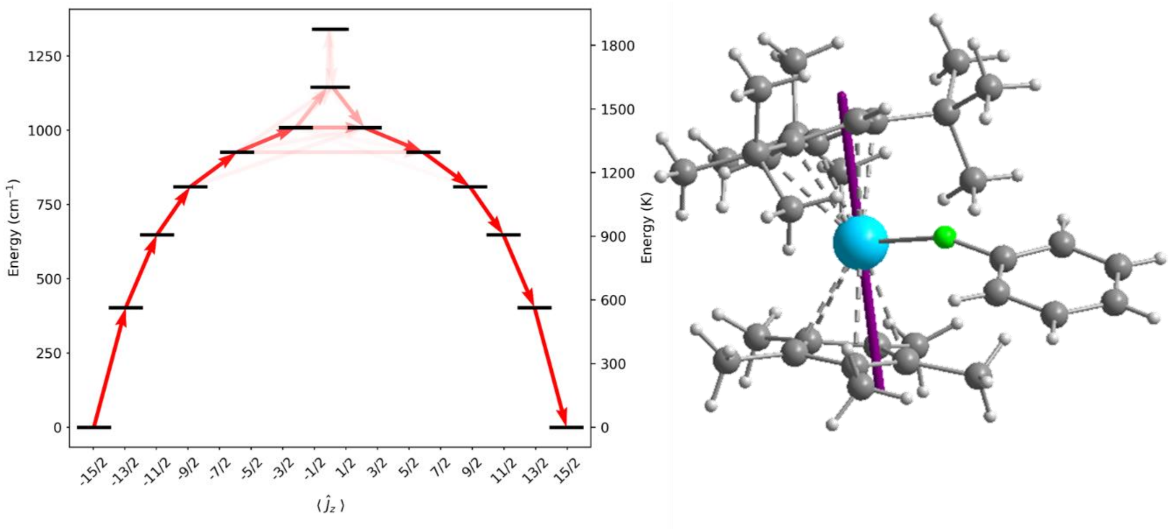

Figure S128. Energy barrier to magnetic relaxation for a model of **2′-Dy**. Electronic states from CASSCF-SO calculations, labelled with their dominant m_J composition in the J = 15/2 basis. Arrows represent the Orbach relaxation pathway, where the opacity of the arrows is proportional to the transition probability approximated with the average matrix elements of magnetic moment connecting the states, $\gamma_{ij} = (1/3)[|\langle i|\mu_x|j\rangle|^2 + |\langle i|\mu_y|j\rangle|^2 + |\langle i|\mu_z|j\rangle|^2]$, normalized from each departing state and commencing from |–15/2> (left). Denotation of the g_z axis (purple) within the solid state structure at the ground state (right).

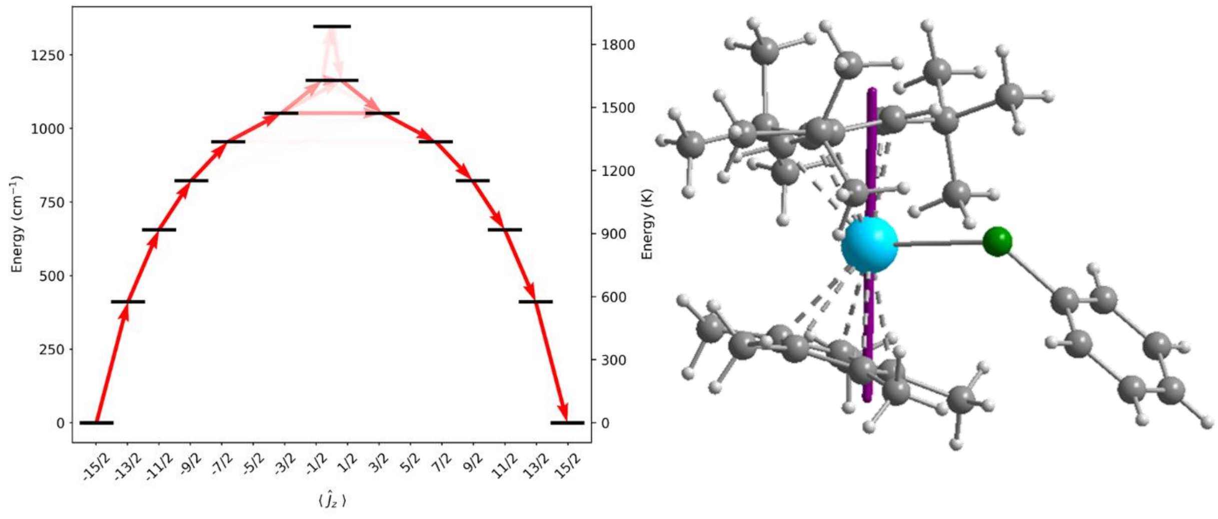

Figure S129. Energy barrier to magnetic relaxation for a model of **3′-Dy**. Electronic states from CASSCF-SO calculations, labelled with their dominant m_J composition in the J = 15/2 basis. Arrows represent the Orbach relaxation pathway, where the opacity of the arrows is proportional to the transition probability approximated with the average matrix elements of magnetic moment connecting the states, $\gamma_{ij} = (1/3)[|\langle i|\mu_x|j\rangle|^2 + |\langle i|\mu_y|j\rangle|^2 + |\langle i|\mu_z|j\rangle|^2]$, normalized from each departing state and commencing from |–15/2> (left). Denotation of the g_z axis (purple) within the solid state structure at the ground state (right).

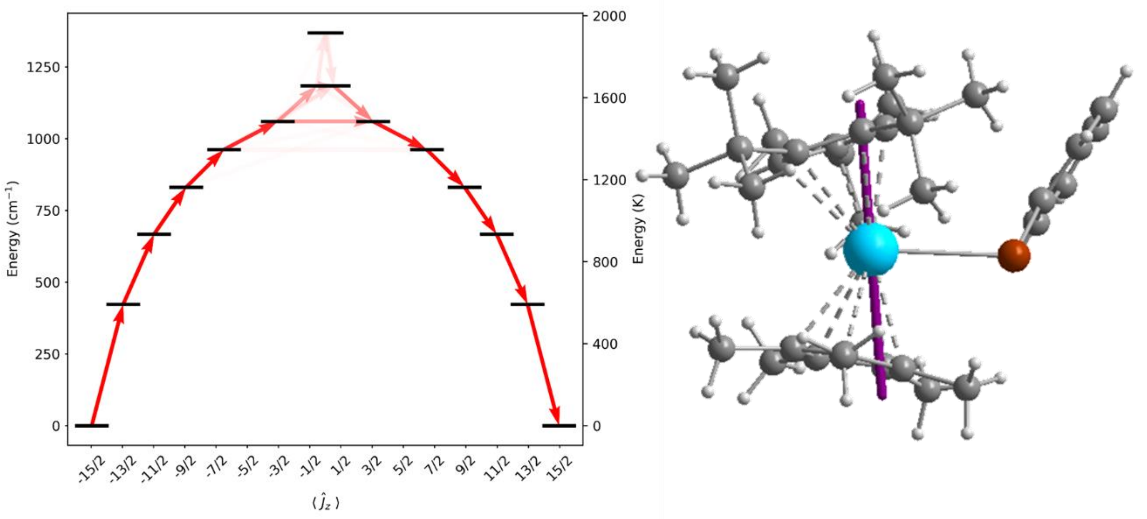

Figure S130. Energy barrier to magnetic relaxation for a model of **4′-Dy**. Electronic states from CASSCF-SO calculations, labelled with their dominant m_J composition in the J = 15/2 basis. Arrows represent the Orbach relaxation pathway, where the opacity of the arrows is proportional to the transition probability approximated with the average matrix elements of magnetic moment connecting the states, $\gamma_{ij} = (1/3)[|\langle i|\mu_x|j\rangle|^2 + |\langle i|\mu_y|j\rangle|^2 + |\langle i|\mu_z|j\rangle|^2]$, normalized from each departing state and commencing from $|-15/2\rangle$ (left). Denotation of the g_z axis (purple) within the solid state structure at the ground state (right).

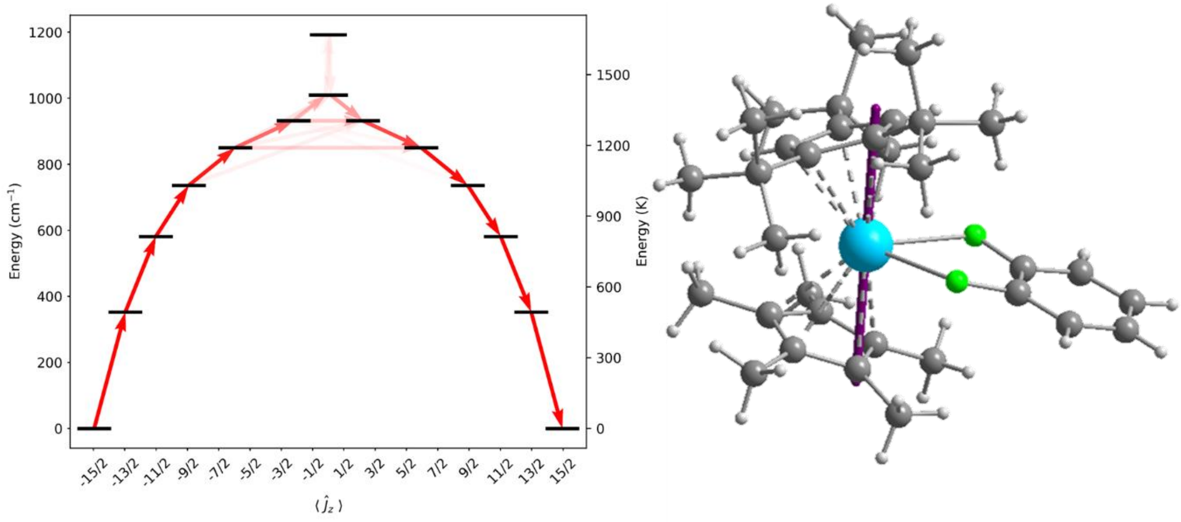

Figure S131. Energy barrier to magnetic relaxation for a model of **5′-Dy**. Electronic states from CASSCF-SO calculations, labelled with their dominant m_J composition in the J = 15/2 basis. Arrows represent the Orbach relaxation pathway, where the opacity of the arrows is proportional to the transition probability approximated with the average matrix elements of magnetic moment connecting the states, $\gamma_{ij} = (1/3)[|<i|\mu_x|j>|^2 + |<i|\mu_y|j>|^2 + |<i|\mu_z|j>|^2]$, normalized from each departing state and commencing from |–15/2> (left). Denotation of the g_z axis (purple) within the solid state structure at the ground state (right).

10. Exchange studies

General procedures

The complex (*ca.* 20 mg for **2-Dy** and **3-Dy**, 10 mg for **4-Dy**) was dissolved in the halobenzene (0.5 mL), layered with *n*-hexane (3 mL) and stored at –35 °C overnight. The solvent was decanted and the crystals dried *in-vacuo*. The identity of crystalline material was confirmed by SCXRD, visual inspection of the crystals, and ATR-IR spectroscopy on microcrystalline solid.

Results

For **2-Dy** in PhCl, SCXRD showed that **3-Dy** had formed, this is in accord with an absorption in the ATR-IR spectrum at 684 cm^{-1}, which is consistent with the authentic sample (see above).

For **2-Dy** in PhBr, SCXRD showed that **4-Dy** had formed, this is in accord with an absorption in the ATR-IR spectrum at 664 cm^{-1}, which is consistent with the authentic sample. However, absorptions at 1112 and 770 cm^{-1}, characteristic of **2-Dy**, indicate that only partial exchange has occurred.

For **2-Dy** in $C_6H_4F_2$, SCXRD showed that **5-Dy** had formed, this is in accord with absorptions in the ATR-IR spectrum at 1494 and 750 cm^{-1}, which is consistent with the authentic sample.

For **3-Dy** in PhF, SCXRD showed that **2-Dy** had formed, this is in accord with absorptions in the ATR-IR spectrum at 1112 and 770 cm^{-1}, which is consistent with the authentic sample.

For **3-Dy** in PhBr, SCXRD showed that **4-Dy** had formed, this is in accord with an absorption in the ATR-IR spectrum at 664 cm^{-1}, which is consistent with the authentic sample.

For **4-Dy** in PhCl, SCXRD showed that **3-Dy** had formed, this is in accord with an absorption in the ATR-IR spectrum at 684 cm^{-1}, which is consistent with the authentic sample.

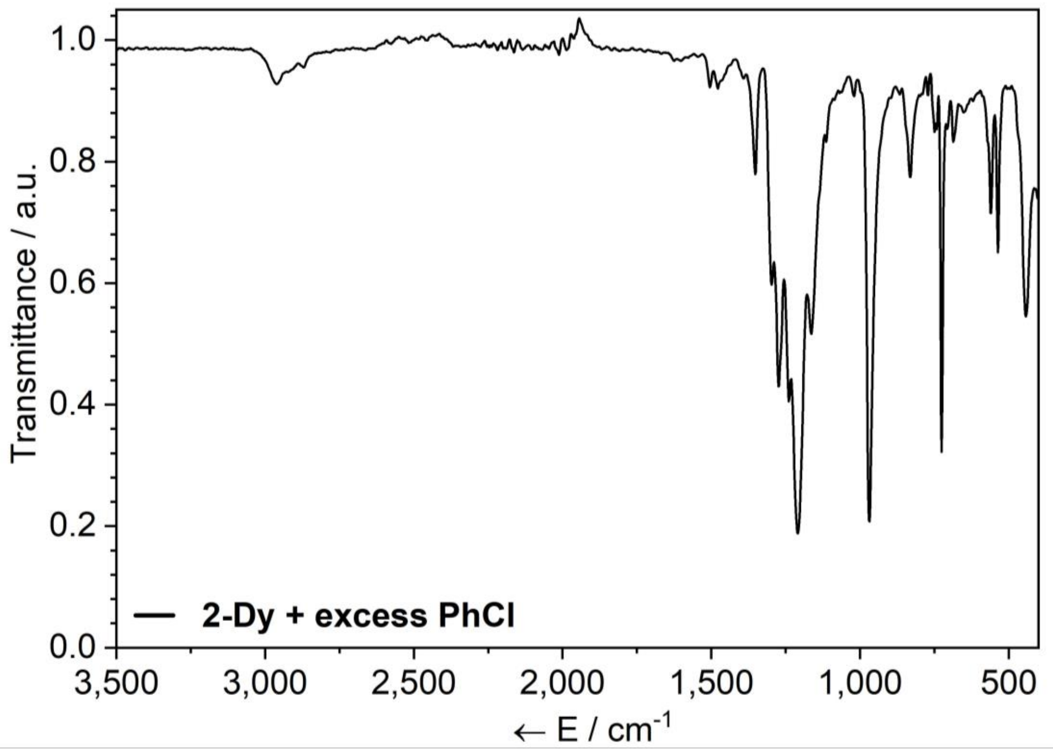

Figure S132. ATR-IR spectrum of **2-Dy**, recrystallized from PhCl layered with *n*-hexane recorded as a microcrystalline powder. The results obtained are consistent with those of pure **3-Dy**.

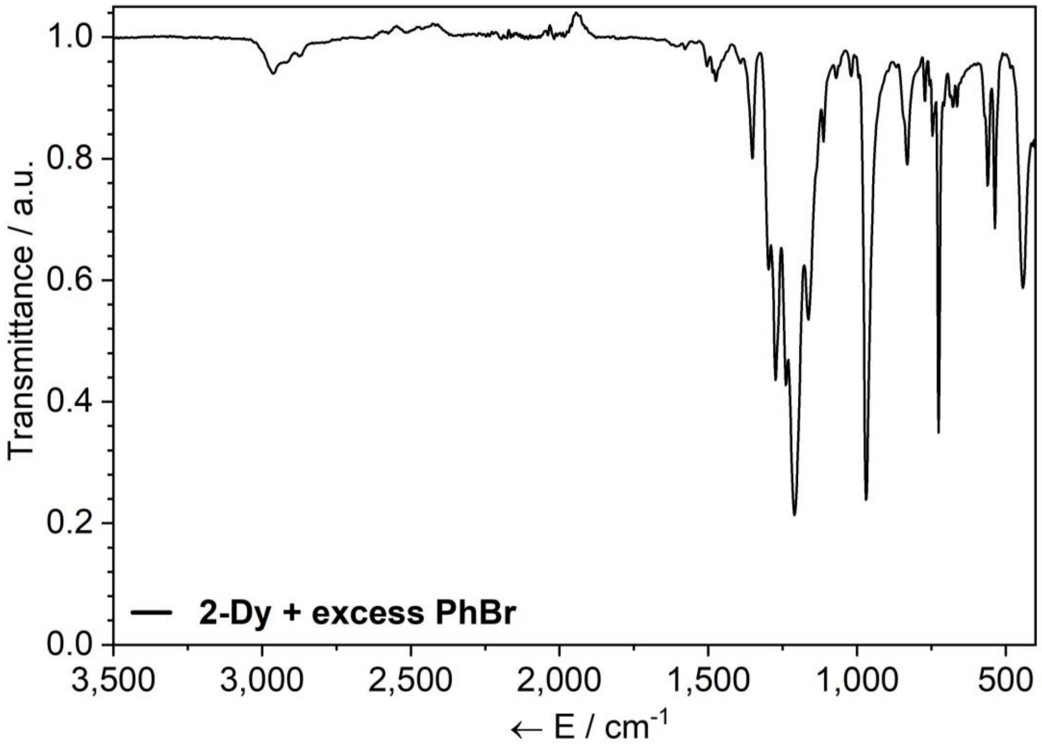

Figure S133. ATR-IR spectrum of **2-Dy**, recrystallized from PhBr layered with *n*-hexane recorded as a microcrystalline powder. The results obtained are consistent with a mix of **2-Dy** and **4-Dy** existing in the sample.

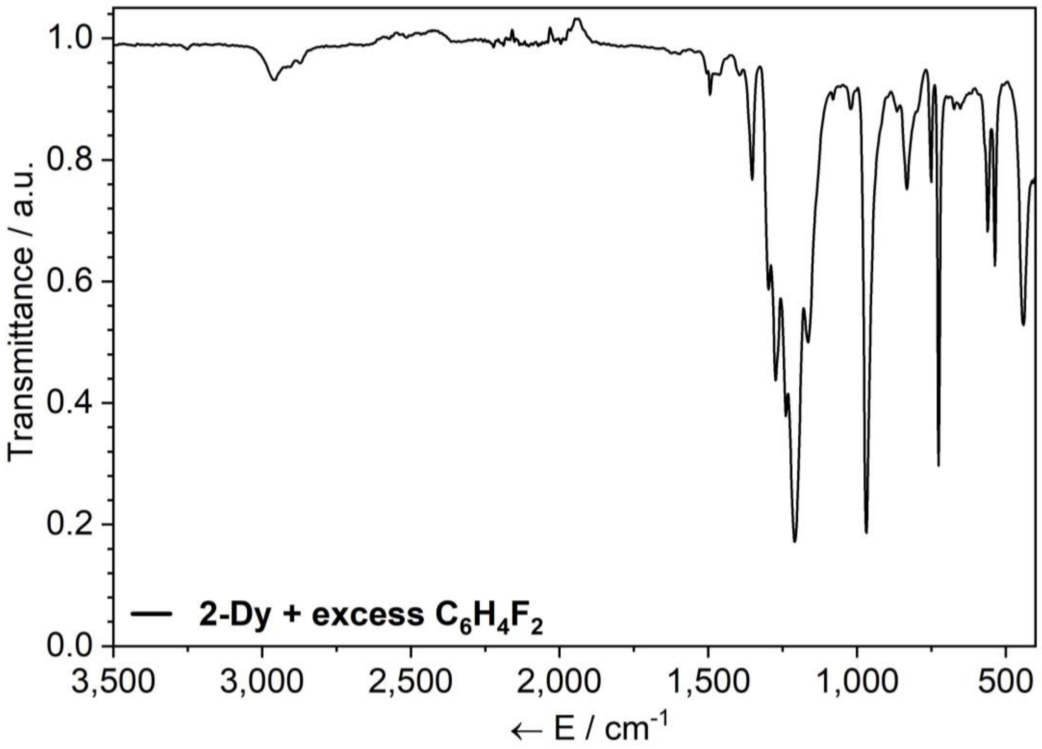

Figure S134. ATR-IR spectrum of **2-Dy**, recrystallized from $C_6H_4F_2$ layered with *n*-hexane recorded as a microcrystalline powder. The results obtained are consistent with those of pure **5-Dy**.

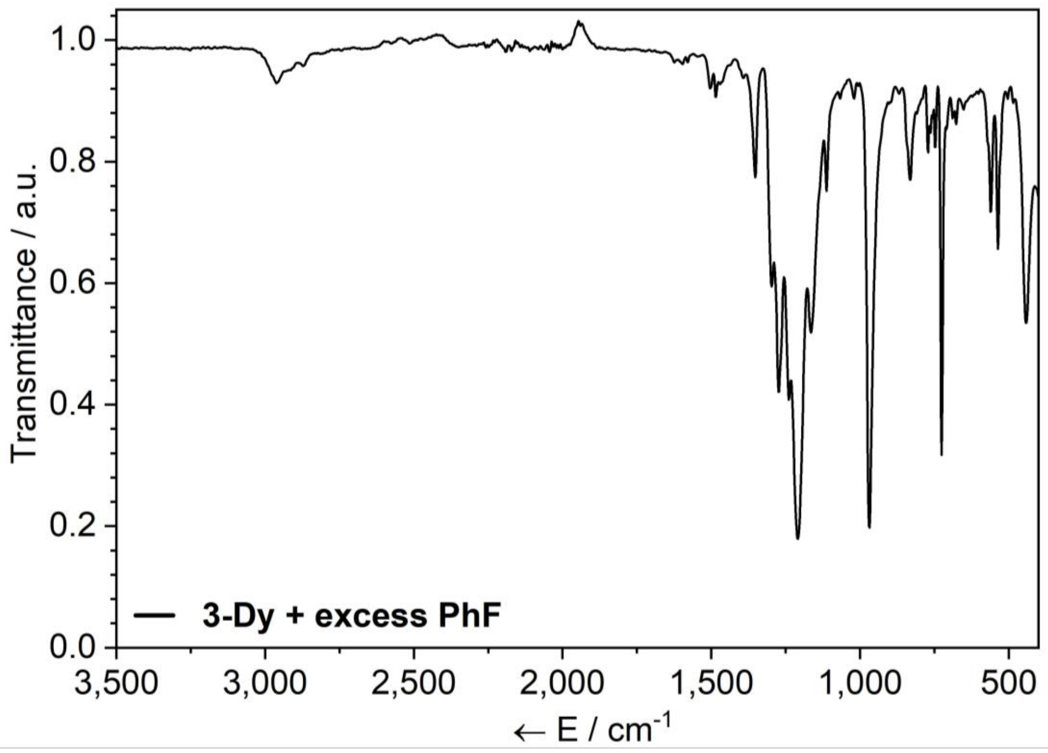

Figure S135. ATR-IR spectrum of **3-Dy**, recrystallized from PhF layered with *n*-hexane recorded as a microcrystalline powder. The results obtained are consistent with those of pure **2-Dy**.

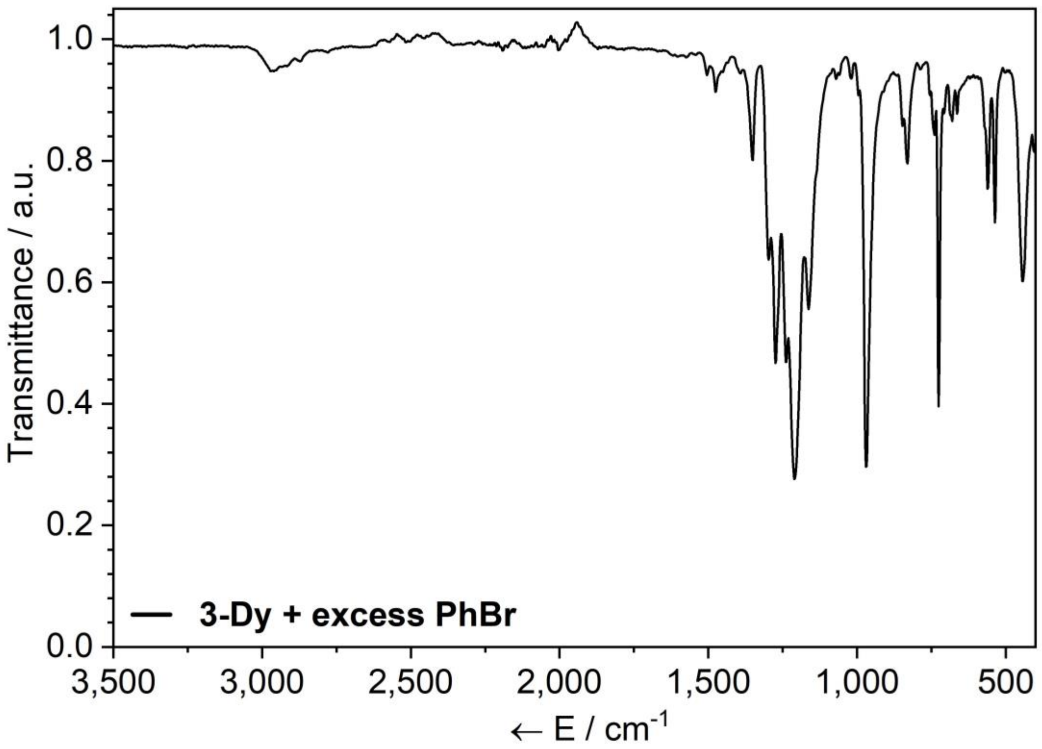

Figure S136. ATR-IR spectrum of **3-Dy**, recrystallized from PhBr layered with *n*-hexane recorded as a microcrystalline powder. The results obtained are consistent with those of pure **4-Dy**.

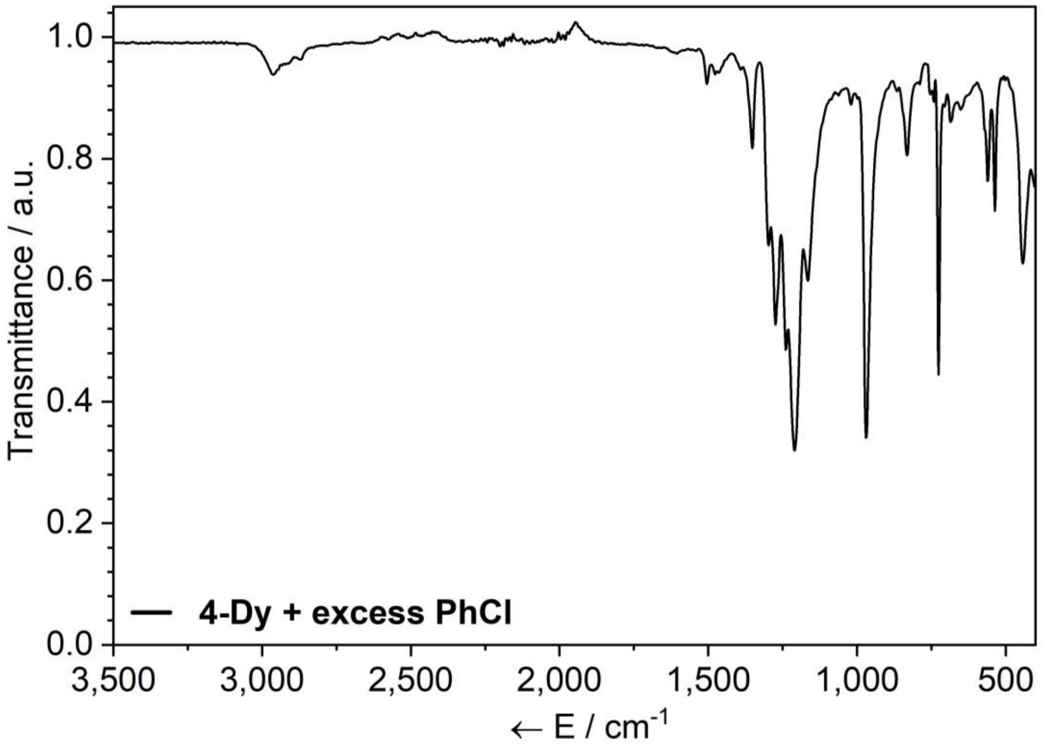

Figure S137. ATR-IR spectrum of **4-Dy**, recrystallized from PhCl layered with *n*-hexane recorded as a microcrystalline powder. The results obtained are consistent with those of pure **3-Dy**.

11. References

(1) Gransbury, G. K.; Corner, S. C.; Kragskow, J. G. C.; Evans, P.; Yeung, H. M.; Blackmore, W. J. A.; Whitehead, G. F. S.; Vitorica-Yrezabal, I. J.; Oakley, M. S.; Chilton, N. F.; Mills, D. P. *AtomAccess*: A Predictive Tool for Molecular Design and Its Application to the Targeted Synthesis of Dysprosium Single-Molecule Magnets. *J. Am. Chem. Soc.* **2023**, *145* (41), 22814–22825. https://doi.org/10.1021/jacs.3c08841.

(2) Krossing, I.; Brands, H.; Feuerhake, R.; Koenig, S. New Reagents to Introduce Weakly Coordinating Anions of Type $Al(OR_F)_4^-$: Synthesis, Structure and Characterization of Cs and Trityl Salts. *J. Fluor. Chem.* **2001**, *112* (1), 83–90. https://doi.org/10.1016/S0022-1139(01)00490-0.

(3) Gabbaï, F. P.; Chirik, P. J.; Fogg, D. E.; Meyer, K.; Mindiola, D. J.; Schafer, L. L.; You, S.-L. An Editorial About Elemental Analysis. *Organometallics* **2016**, *35* (19), 3255–3256. https://doi.org/10.1021/acs.organomet.6b00720.

(4) Fadeeva, V. P.; Tikhova, V. D.; Nikulicheva, O. N. Elemental Analysis of Organic Compounds with the Use of Automated CHNS Analyzers. *J. Anal. Chem.* **2008**, *63* (11), 1094–1106. https://doi.org/10.1134/S1061934808110142.

(5) Kabova, E. A.; Blundell, C. D.; Muryn, C. A.; Whitehead, G. F. S.; Vitorica-Yrezabal, I. J.; Ross, M. J.; Shankland, K. SDPD-SX: Combining a Single Crystal X-Ray Diffraction Setup with Advanced Powder Data Structure Determination for Use in Early Stage Drug Discovery. *CrystEngComm* **2022**, *24* (24), 4337–4340. https://doi.org/10.1039/D2CE00387B.

(6) *CrysAlis PRO*; Agilent Technologies Ltd: Yarnton, Oxfordfordshire, England 2014.

(7) Pawley, G. S. Unit-Cell Refinement from Powder Diffraction Scans. *J. Appl. Crystallogr.* **1981**, *14* (6), 357–361. https://doi.org/10.1107/S0021889881009618.

(8) Coelho, A. A. An Indexing Algorithm Independent of Peak Position Extraction for X-

Ray Powder Diffraction Patterns. *J. Appl. Crystallogr.* **2017**, *50* (5), 1323–1330. https://doi.org/10.1107/S1600576717011359.

(9) Nowell, H.; Barnett, S. A.; Christensen, K. E.; Teat, S. J.; Allan, D. R. I19, the Small-Molecule Single-Crystal Diffraction Beamline at Diamond Light Source. *J. Synchrotron Radiat.* **2012**, *19* (3), 435–441. https://doi.org/10.1107/S0909049512008801.

(10) Sheldrick, G. M. Crystal Structure Refinement with SHELXL. *Acta Crystallogr. Sect. C Struct. Chem.* **2015**, *71* (1), 3–8. https://doi.org/10.1107/S2053229614024218.

(11) Dolomanov, O. V.; Bourhis, L. J.; Gildea, R. J.; Howard, J. A. K.; Puschmann, H. OLEX2 : A Complete Structure Solution, Refinement and Analysis Program. *J. Appl. Crystallogr.* **2009**, *42* (2), 339–341. https://doi.org/10.1107/S0021889808042726.

(12) Farrugia, L. J. WinGX and ORTEP for Windows : An Update. *J. Appl. Crystallogr.* **2012**, *45* (4), 849–854. https://doi.org/10.1107/S0021889812029111.

(13) *Persistence of Vision Raytracer.* Persistence of Vision Raytracer Pty. Ltd., v.3.7, 2013. Retrieved from http://www.povray.org/download/.

(14) Neese, F. The ORCA Program System. *WIREs Comput. Mol. Sci.* **2012**, *2* (1), 73–78. https://doi.org/10.1002/wcms.81.

(15) Neese, F. Software Update: The ORCA Program System, Version 4.0. *WIREs Comput. Mol. Sci.* **2018**, *8* (1), e1327. https://doi.org/10.1002/wcms.1327.

(16) Lehtola, S.; Steigemann, C.; Oliveira, M. J. T.; Marques, M. A. L. Recent Developments in LIBXC — A Comprehensive Library of Functionals for Density Functional Theory. *SoftwareX* **2018**, *7*, 1–5. https://doi.org/10.1016/j.softx.2017.11.002.

(17) Caldeweyher, E.; Bannwarth, C.; Grimme, S. Extension of the D3 Dispersion Coefficient Model. *J. Chem. Phys.* **2017**, *147* (3), 034112. https://doi.org/10.1063/1.4993215.

(18) Caldeweyher, E.; Ehlert, S.; Hansen, A.; Neugebauer, H.; Spicher, S.; Bannwarth, C.;

Grimme, S. A Generally Applicable Atomic-Charge Dependent London Dispersion Correction. *J. Chem. Phys.* **2019**, *150* (15), 154122. https://doi.org/10.1063/1.5090222.

(19) Weigend, F.; Ahlrichs, R. Balanced Basis Sets of Split Valence, Triple Zeta Valence and Quadruple Zeta Valence Quality for H to Rn: Design and Assessment of Accuracy. *Phys. Chem. Chem. Phys.* **2005**, *7* (18), 3297–3305. https://doi.org/10.1039/b508541a.

(20) Weigend, F. Accurate Coulomb-Fitting Basis Sets for H to Rn. *Phys. Chem. Chem. Phys.* **2006**, *8* (9), 1057. https://doi.org/10.1039/b515623h.

(21) Andrae, D.; Häußermann, U.; Dolg, M.; Stoll, H.; Preuß, H. Energy-Adjusted *Ab Initio* Pseudopotentials for the Second and Third Row Transition Elements. *Theor. Chim. Acta* **1990**, *77* (2), 123–141. https://doi.org/10.1007/BF01114537.

(22) Reta, D.; Chilton, N. F. Uncertainty Estimates for Magnetic Relaxation Times and Magnetic Relaxation Parameters. *Phys. Chem. Chem. Phys.* **2019**, *21* (42), 23567–23575. https://doi.org/10.1039/C9CP04301B.

(23) Blackmore, W. J. A.; Gransbury, G. K.; Evans, P.; Kragskow, J. G. C.; Mills, D. P.; Chilton, N. F. Characterisation of Magnetic Relaxation on Extremely Long Timescales. *Phys. Chem. Chem. Phys.* **2023**, *25* (25), 16735–16744. https://doi.org/10.1039/D3CP01278F.

(24) *OriginPro*; OriginLab Corporation: Northampton, England, 2022.

(25) Zorn, R. Logarithmic Moments of Relaxation Time Distributions. *J. Chem. Phys.* **2002**, *116* (8), 3204–3209. https://doi.org/10.1063/1.1446035.

(26) Rousset, E.; Piccardo, M.; Boulon, M.; Gable, R. W.; Soncini, A.; Sorace, L.; Boskovic, C. Slow Magnetic Relaxation in Lanthanoid Crown Ether Complexes: Interplay of Raman and Anomalous Phonon Bottleneck Processes. *Chem. Eur. J.* **2018**, *24* (55), 14768–14785. https://doi.org/10.1002/chem.201802779.

(27) Arauzo, A.; Bartolomé, E.; Benniston, A. C.; Melnic, S.; Shova, S.; Luzón, J.; Alonso,

P. J.; Barra, A.-L.; Bartolomé, J. Slow Magnetic Relaxation in a Dimeric Mn_2 Ca_2 Complex Enabled by the Large Mn(III) Rhombicity. *Dalton Trans.* **2017**, *46* (3), 720–732. https://doi.org/10.1039/C6DT02509A.

(28) Stoneham, A. M. The Phonon Bottleneck in Paramagnetic Crystals. *Proc. Phys. Soc.* **1965**, *86* (6), 1163–1177. https://doi.org/10.1088/0370-1328/86/6/302.

(29) Fdez. Galván, I.; Vacher, M.; Alavi, A.; Angeli, C.; Aquilante, F.; Autschbach, J.; Bao, J. J.; Bokarev, S. I.; Bogdanov, N. A.; Carlson, R. K.; Chibotaru, L. F.; Creutzberg, J.; Dattani, N.; Delcey, M. G.; Dong, S. S.; Dreuw, A.; Freitag, L.; Frutos, L. M.; Gagliardi, L.; Gendron, F.; Giussani, A.; González, L.; Grell, G.; Guo, M.; Hoyer, C. E.; Johansson, M.; Keller, S.; Knecht, S.; Kovačević, G.; Källman, E.; Li Manni, G.; Lundberg, M.; Ma, Y.; Mai, S.; Malhado, J. P.; Malmqvist, P. Å.; Marquetand, P.; Mewes, S. A.; Norell, J.; Olivucci, M.; Oppel, M.; Phung, Q. M.; Pierloot, K.; Plasser, F.; Reiher, M.; Sand, A. M.; Schapiro, I.; Sharma, P.; Stein, C. J.; Sørensen, L. K.; Truhlar, D. G.; Ugandi, M.; Ungur, L.; Valentini, A.; Vancoillie, S.; Veryazov, V.; Weser, O.; Wesołowski, T. A.; Widmark, P. O.; Wouters, S.; Zech, A.; Zobel, J. P.; Lindh, R. OpenMolcas: From Source Code to Insight. *J. Chem. Theory Comput.* **2019**, *15* (11), 5925–5964. https://doi.org/10.1021/acs.jctc.9b00532.

(30) Roos, B. O.; Veryazov, V.; Widmark, P.-O. Relativistic Atomic Natural Orbital Type Basis Sets for the Alkaline and Alkaline-Earth Atoms Applied to the Ground-State Potentials for the Corresponding Dimers. *Theor. Chem. Acc.* **2004**, *111* (2–6), 345–351. https://doi.org/10.1007/s00214-003-0537-0.

(31) Roos, B. O.; Lindh, R.; Malmqvist, P. Å.; Veryazov, V.; Widmark, P. O. Main Group Atoms and Dimers Studied with a New Relativistic ANO Basis Set. *J. Phys. Chem. A* **2004**, *108* (15), 2851–2858. https://doi.org/10.1021/jp031064+.

(32) Roos, B. O.; Lindh, R.; Malmqvist, P.-Å.; Veryazov, V.; Widmark, P.-O. New

Relativistic ANO Basis Sets for Transition Metal Atoms. *J. Phys. Chem. A* **2005**, *109* (29), 6575–6579. https://doi.org/10.1021/jp0581126.

(33) Roos, B. O.; Lindh, R.; Malmqvist, P.-Å.; Veryazov, V.; Widmark, P.-O.; Borin, A. C. New Relativistic Atomic Natural Orbital Basis Sets for Lanthanide Atoms with Applications to the Ce Diatom and LuF_3. *J. Phys. Chem. A* **2008**, *112* (45), 11431–11435. https://doi.org/10.1021/jp803213j.

(34) Chibotaru, L. F.; Ungur, L. Ab Initio Calculation of Anisotropic Magnetic Properties of Complexes. I. Unique Definition of Pseudospin Hamiltonians and Their Derivation. *J. Chem. Phys.* **2012**, *137* (6), 064112. https://doi.org/10.1063/1.4739763.

(35) *Diamond-Crystal and Molecular Structure Visualization, v.4.6.6.;* Putz, H.; Brandenburg, K.; Crystal Impact GbR, Bonn, Germany, 2021.
